# Epidemiological trends of early-onset gastrointestinal cancers from 1990 to 2021 and predictions for 2036: analysis from the global burden of disease study 2021

**DOI:** 10.1080/07853890.2025.2555518

**Published:** 2025-09-05

**Authors:** Yu-Qing Gong, Chun-Jing Lu, Yan-Ru Xiao, Jin-Yan Zhang, Zhong Xu, Ji Li, Wei-Feng Huang

**Affiliations:** a Department of Gastroenterology and Hepatology, The First Affiliated Hospital of Xiamen University, School of Medicine, Xiamen University, Xiamen, China; b Department of Blood Transfusion, Women and Children’s Hospital, School of Medicine, Xiamen University, Xiamen, China; c Department of Medical Oncology, The First Affiliated Hospital of Xiamen University, School of Medicine, Xiamen University, Xiamen, China; d The School of Clinical Medicine, Fujian Medical University, Fuzhou, China

**Keywords:** Disability-adjusted life years, early-onset gastrointestinal cancer, global burden of disease study, incidence, mortality

## Abstract

**Background:**

Gastrointestinal cancers account for 39.0% of global cancer-related deaths. The rising incidence of early-onset gastrointestinal cancers poses a substantial public health challenge due to their aggressive nature and poor prognosis.

**Methods:**

Using the Global Burden of Disease (GBD) 2021 database, we analyzed temporal trends (1990-2021) in age-standardized incidence (ASIR), mortality (ASMR), and disability-adjusted life years (ASDR) rates for early-onset gastrointestinal cancers among individuals aged 15-49 years. Analyses included burden by age, sex, region, and socio-demographic index (SDI). Joinpoint regression estimated average annual percentage changes (AAPC), and future trends (2022-2036) were projected using the Bayesian Age-Period-Cohort model.

**Results:**

In 2021, there were 499,800 new cases of early-onset gastrointestinal cancers globally. Colorectal and stomach cancers were most prevalent. From 1990-2021, ASIR increased only for colorectal cancer (AAPC: 0.37, 95%CI: 0.24-0.50), while declines occurred in esophageal, stomach, liver, pancreatic, and gallbladder/biliary tract cancers. Males bore a higher burden except for gallbladder/biliary tract cancer. Significant disparities existed across regions, genders, and SDI levels. Smoking and high body-mass index were leading risk factors. Projections indicate rising ASIRs for early-onset colorectal and esophageal cancers, stable liver cancer incidence, and diminishing incidence, mortality, and DALYs rates for stomach, pancreatic, and gallbladder and biliary tract cancers.

**Conclusions:**

Early-onset gastrointestinal cancers represent a substantial and evolving global burden, with notable variations by cancer type, age, sex, region, and SDI. Targeted prevention and healthcare strategies are urgently needed to address modifiable risk factors and reduce disparities.

## KEY MESSAGES

Early-onset gastrointestinal cancers pose a significant impact on public health, but current research on these diseases remain rare. This study represents the first comprehensive analysis of the global burden of early-onset gastrointestinal cancers from 1990 to 2021. We confirmed the substantial burden of early-onset gastrointestinal cancers and identify an upward trend in age-standardized incidence rate for colorectal cancer. High-middle socio-demographic index region showed great burden of early-onset gastrointestinal cancers. Smoking and high body mass index were major contributors to mortality.

## 1. Introduction

With cancer remaining a foremost contributor to global mortality (10 million deaths in 2019) [1], the gastrointestinal cancer burden warrants particular attention. Epidemiological evidence reveals that one in twelve individuals will develop gastrointestinal cancers, and one in sixteen will die from these conditions [2]. Gastrointestinal cancers include esophageal, stomach, colorectal, liver, pancreatic, and gallbladder and biliary tract cancer [3]. Although cancers predominantly affect individuals over the age of 65, there has been a growing incidence of cancer among adolescents and young adults (AYAs) [4–6], including gastrointestinal cancers [7]. Cancer in AYAs differs significantly from that in older individuals, with distinct biological, epidemiological, and clinical features [8]. Early-onset cancers are particularly concerning, as they tend to be more aggressive and are associated with lower five-year survival rates [8,9]. Moreover, these cancers have a profound impact on the quality of life, often leading to long-term health complications, socioeconomic burdens, and psychological distress, all of which intensify the societal impact [10,11]. Despite the growing importance of early-onset gastrointestinal cancers, research in this area has been limited, particularly concerning survival rates, which have shown less improvement compared to those in older patient populations [12–14].

Epidemiological data documented a sustained increase in colorectal cancer incidence among young individuals over the past three decades [15]. This concerning pattern extends beyond colorectal cancers, as evidenced by data from the Surveillance, Epidemiology, and End Results (SEER) and multinational registry data, which demonstrate a rising trend of early-onset gastrointestinal cancers [16]. The magnitude and direction of this trend vary by cancer site and may be related to earlier diagnosis resulting from advances in diagnostic techniques, improvements in public health awareness, and progress in screening technologies [17,18]. In addition, diet, lifestyle, obesity, environmental exposures, microbiota, and other factors also influence cancer incidence [16]. More than half of gastrointestinal cancers are attributed to preventable risk factors, including alcohol consumption, metabolic syndrome, infections, and dietary factors [19]. The changes in early-onset cancer trends may reflect increased exposure to risk factors during early life and/or young adulthood [15].

Although early-onset gastrointestinal cancers pose a significant impact on public health, current research has predominantly concentrated on early-onset colorectal and early-onset pancreatic cancer. To fill this epidemiological gap, our research aims to conduct the first comprehensive analysis of the incidence, mortality, and disability-adjusted life years (DALYs) associated with early-onset gastrointestinal cancers from 1990 to 2021, using data from the Global Burden of Disease (GBD) 2021 Study. Additionally, this study will explore the role of risk factors and forecast trends up to 2036, providing actionable insights for preventive strategies.

## 2. Methods

### 2.1. Data sources and definition

The GBD 2021 provides comprehensive estimates of health loss from 371 diseases and injuries and 88 risk factors across 204 countries and territories between 1990 and 2021 [20]. The Global Health Data Exchange (GHDx) query tool, provided by the Institute for Health Metrics and Evaluation (IHME), serves as an online resource for collecting data on the incidence, mortality, and DALYs of early-onset gastrointestinal cancers (https://ghdx.healthdata.org/gbd-2021/sources).

In the GBD 2021, gastrointestinal cancers were systematically categorized using International Classification of Diseases (ICD) topography codes: ICD-9 and ICD-10 (Table S1). In this study, early-onset gastrointestinal cancers are defined as cancers affecting adolescents and young adults aged 15–49 years [18,21,22]. We quantified the burden of early-onset gastrointestinal cancers (esophageal, stomach, colorectal, liver, pancreatic, and gallbladder and biliary tract cancers) in individuals aged 15–49 years between 1990 and 2021, examined the role of associated risk factors, and predicted trends from 2022 to 2036. The 15–49 age group is subdivided into seven age groups at five-year intervals. To address potential selection bias and assess result stability, sensitivity analyses were conducted within the 15–49 age cohort employing alternative stratification schemes: comparative evaluation of quinquennial (5-year) intervals versus three-tier categorization (15–29, 30–44, and 45–49 years). Given the use of publicly available, anonymized aggregate data, no ethical approval or informed consent was required.

### 2.2. Overview of disease burden estimates

The GBD 2021 employed the Bayesian meta-regression modeling tool (DisMod-MR, version 2.1) to leverage all available epidemiological data and produce internally consistent disease burden. Cause of Death Ensemble model (CODEm) was employed to assess mortality based on various factors, including age, sex, location, and year. This model applies Bayesian geospatial regression analysis, accounting for spatial relationships among data points. Mortality is defined as the probability of death before age 50 years conditional on survival to age 15 years, consistent with the GBD mortality methodology [23]. Detailed explanations of the underlying principles of GBD 2021 are available in previous studies [24].

The study also included the Socio-demographic Index (SDI) across 21 regions to evaluate the level of development. Tables S2 and S3 presents detailed SDI values for different regions. SDI is a composite index based on three indicators: the total fertility rates among females under 25, average years of education for individuals aged 15 and above, and per capita income [4]. The SDI ranges from 0 to 1, with countries and regions classified into five levels of development: low (0 < SDI ≤ 0.570), lower-middle (0.570 < SDI ≤ 0.670), middle (0.670 < SDI ≤ 0.812), high-middle (0.812 < SDI ≤ 0.858), and high (0.858 < SDI ≤ 1) [5].

### 2.3. Statistical analysis

Age-standardized rates (ASRs) for every 1,00,000 individuals were extracted from the GBD 2021 database. The ASR is calculated as follows:

$$\text{ASR}=\frac{\sum_{i=1}^{N}a_{i}W_{i}}{\sum_{i=1}^{N}W_{i}}$$

In the equation, *a_i_* is the specific age rate for the *i*th age group, *w_i_* represents the population in the same age group from the GBD standard population (or the weight) and *N* is the number of age groups. The 95% uncertainty interval (UI) is represented by the 2.5th and 97.5th percentile values across the draws.

Temporal trends in age-standardized incidence rate (ASIR), mortality rate (ASMR), and disability-adjusted life years rate (ASDR) were analyzed using Joinpoint Regression software (version 5.0.2). This segmented regression approach quantified average annual percentage changes (AAPC) with 95% confidence intervals (CIs) by identifying optimal inflection points in log-transformed temporal series. Model specification was subsequently determined through iterative comparison of weighted Bayesian Information Criterion (BIC) values, with parameter constraints limiting the maximum number of segments to seven (six joinpoints) [25]. The AAPC is calculated as:

$$\text{AAPC}=\left\{\text{exp}\left(\frac{\sum w\text{ibi}}{\sum wi}\right)-1\right\}\;\times \;100$$

where *w_i_* is the length of each segment in the year range, and b_i_ represents the slope coefficient for each segment. If the AAPC and its 95% CI lower bound are both greater than 0, an increasing trend is identified. If the AAPC and its 95% CI upper bound are both less than 0, a decreasing trend is confirmed. Otherwise, the rate remains stable over time. The standard error (SE) is calculated as: SE = (*upper*-*lower*)/(1.96 × 2), where the upper and lower bounds represent the two CI boundaries obtained from the GBD.

Further subgroup analysis, risk factors evaluation, and future trends forecasting were systematically conducted. Risk factors were identified based on the World Cancer Research Fund’s risk-outcome associations and the Comparative Risk Assessment Framework [26]. The GBD database employs hierarchical regression models to derive spatially resolved, age-sex-year stratified risk exposure estimates. For each validated risk-outcome association, population attributable fractions (PAFs) were computed through systematic integration of exposure distributions and meta-analyzed relative risks. Attributable burden metrics, including DALYs and mortality rates, were subsequently calculated by applying these PAFs to total burden estimates [27]. Mortality rates attributable to the most detailed risk factors was estimated. For forecasting early-onset gastrointestinal cancer burden from 2022 to 2036, Bayesian Age-Period-Cohort (BAPC) models were used with R software (BAPC 0.0.36 and INLA 24.05.10) to predict ASIR, ASMR, and ASDR. The BAPC model tests the combined effects of age, period, and cohort [28]: *η*_ij_ = *µ* + *α*_i_ + *β*_j_ + *γ*_k_. Where *η*_ij_ represents ASR, µ is the intercept, and *α*_i_, *β*_j,_ and *γ*_k_ represent the age, period, and cohort effects, respectively. This approach operates under the assumption that temporally adjacent age, period, and cohort effects exhibit similarity. Second-order random walk priors were employed to smooth estimated effects and project posterior mortality rates. Integrated Nested Laplace Approximation (INLA) was utilized to approximate marginal posterior distributions, circumventing the mixing and convergence issues inherent in traditional Markov chain Monte Carlo sampling techniques for Bayesian inference [29,30]. All analyses were conducted using R (version 4.4.0), with a two-tailed *p*-value of < 0.05 considered statistically significant.

## 3. Results

### 3.1. Overview of gastrointestinal cancers in 2021

Global surveillance data for 2021 documented 499,800 incident cases of early-onset gastrointestinal cancers, revealing a striking male predominance (males: 328,368 cases, 65.70%; females: 171,432 cases, 34.30%; Table 1). The disease burden was further evidenced by an estimated 285,896 deaths worldwide, of which 66.67% (190,738) occurred in males and 33.33% (95,157) in females. DALYs totaled 14,008,900 globally, distributed as 9,288,747 (66.31%) male and 4,720,152 (33.69%) female cases. Among the various types of early-onset gastrointestinal cancers, colorectal cancer exhibited the highest ASIR at 5.15 per 100,000 population (95%UI: 4.68 to 5.67), ASMR at 1.93 (95%UI: 1.76–2.11) and ASDR at 97.68 (95%UI: 88.78 to 106.85) (Table 1).

**Table 1.** The incidence, mortality, and DALYs numbers and rates of early-onset gastrointestinal cancers in 2021 globally.

	Incidence		Mortality		DALYs	
	Number (95%UI)	ASR (95%UI)	Number (95%UI)	ASR (95%UI)	Number(95%UI)	ASR (95%UI)
Gastrointestinal cancers						
Both sex	499800 (443833,561965)	12.13 (10.71,13.72)	285896 (253659,322678)	6.94 (6.11,7.86)	14008900 (12436230,15773980)	341.10 (300.66,386.00)
Female	171432 (153939,191208)	4.17 (3.72,4.68)	95157 (85115,106019)	2.31 (2.06,2.60)	4720152 (4221962,5265722)	115.25 (102.34,129.71)
Male	328368 (278771,387697)	7.96 (4.12,9.41)	190738 (162464,224497)	4.62 (3.90,5.45)	9288747 (7920317,10908345)	228.01 (182.48,261.97)
Esophageal cancer						
Both sex	42698 (38138,47972)	1.03 (0.91,1.16)	32922 (29481,36953)	0.79 (0.70,0.90)	1551915 (1388751,1740151)	37.52 (33.33,42.37)
Female	9911 (8365,11799)	0.48 (0.41,0.58)	7620 (6455,9120)	0.37 (0.31,0.45)	367041 (310728,441180)	17.97 (15.2,21.88)
Male	32787 (28342,37780)	1.57 (1.35,1.82)	25302 (22061,28917)	1.21 (1.05,1.40)	1184874 (1033997,1350955)	56.81 (49.22,65.40)
Stomach cancer						
Both sex	125121 (107274,144783)	3.04 (2.61,3.52)	78871 (68704,90836)	1.92 (1.66,2.20)	3859036 (3356411,4428942)	94.03 (81.42,107.52)
Female	43336 (38788,48813)	2.13 (1.89,2.41)	29057 (26115,32185)	1.43 (1.28,1.59)	1447833 (1301321,1605517)	71.46 (63.78,79.53)
Male	81785 (66841,101136)	3.94 (3.17,4.83)	49814 (41140,61050)	2.40 (1.94,2.90)	2411203 (1989622,2940774)	116.31 (94.17,140.25)
Colorectal cancer						
Both sex	211890 (193832,231272)	5.15 (4.68,5.67)	79504 (72699,86539)	1.93 (1.76,2.11)	4002756 (3666300,4350565)	97.68 (88.78,106.85)
Female	85224 (77873,93407)	4.17 (3.80,4.61)	32145 (29391,35098)	1.57 (1.43,1.73)	1615410 (1475902,1767273)	79.49 (72.11,87.75)
Male	126666 (110037,144645)	6.11 (5.30,7.00)	47359 (41411,53364)	2.29 (2.00,2.59)	2387346 (2093226,2687482)	115.58 (100.78,131)
Liver cancer						
Both sex	74948 (65248,87630)	1.82 (1.57,2.16)	58825 (51340,68517)	1.43 (1.23,1.69)	2889492 (2528098,3356476)	70.53 (60.87,83.05)
Female	15911 (14229,17834)	0.78 (0.69,0.89)	12895 (11582,14375)	0.64 (0.56,0.72)	647956 (582243,722083)	32.12 (28.43,36.46)
Male	59037 (49998,72069)	2.84 (2.36,3.49)	45930 (38909,55822)	2.21 (1.85,2.70)	2241535 (1901989,2722914)	108.37 (90.59,132.24)
Pancreatic cancer						
Both sex	31531 (28671,34517)	0.76 (0.69,0.84)	26996 (24493,29598)	0.65 (0.59,0.72)	1285174 (1164116,1407685)	31.16 (28.24,34.29)
Female	10360 (9483,11205)	0.50 (0.46,0.55)	8744 (7986,9486)	0.43 (0.39,0.47)	417145 (380540,452563)	20.39 (18.52,22.31)
Male	21171 (18931,23781)	1.02 (0.90,1.14)	18251 (16265,20502)	0.88 (0.78,0.99)	868029 (773603,974430)	41.77 (37.11,47)
Gallbladder and biliary tract cancer						
Both sex	13612 (10670,15791)	0.33 (0.26,0.38)	8778 (6942,10235)	0.21 (0.17,0.25)	420527 (332554,490161)	10.20 (8.04,11.95)
Female	6690 (5201,8150)	0.33 (0.25,0.40)	4696 (3586,5755)	0.23 (0.17,0.28)	224767 (171228,277106)	10.99 (8.33,13.65)
Male	6922 (4622,8286)	0.33 (0.22,0.40)	4082 (2678,4842)	0.20 (0.13,0.24)	195760 (127880,231790)	9.43 (6.14,11.33)

ASR, age-standardized rate. DALYs, disability-adjusted life years. UI, uncertainty interval.

**Figure 1.**
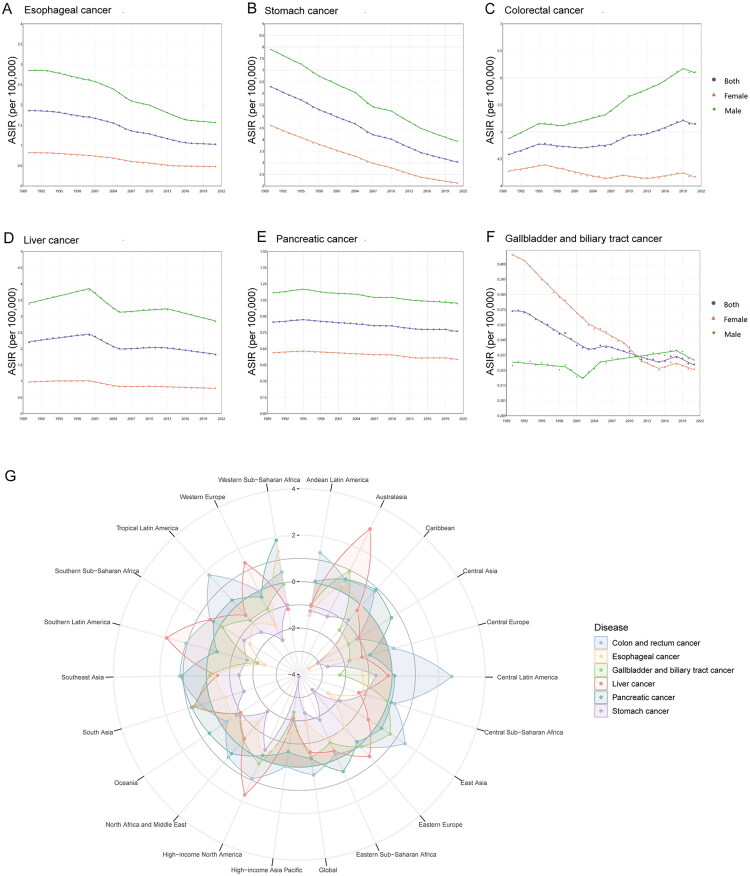
Temporal trends of ASIR in burden of early-onset gastrointestinal cancers from 1990 to 2021. (A-F) Temporal trend of ASIR in burden of early-onset gastrointestinal cancers, globally, 1990–2021. (G) AAPC of ASIR in burden of early-onset gastrointestinal cancers, regionally, 1990–2021. ASIR, age-standardized incidence rate. AAPC, average annual percentage changes.

Temporal trends analysis identified colorectal cancer as the only cancer with increasing ASIR (AAPC: 0.37, 95%CI: 0.24–0.50), whereas stomach cancer had the largest decrease of ASIR (AAPC: −2.32, 95%CI: −2.41 to −2.22) (Figure 1 and Table S4). All gastrointestinal cancers showed decreasing ASMR and ASDR trends, with stomach cancer demonstrated the most pronounced decline (ASMR: AAPC: −2.87, 95%CI: −3.04 to −2.71; ASDR: AAPC: −2.87, 95%CI: −3.06 to −2.68) (Figure S1, S2, Tables S5 and S6).

**Figure 2.**
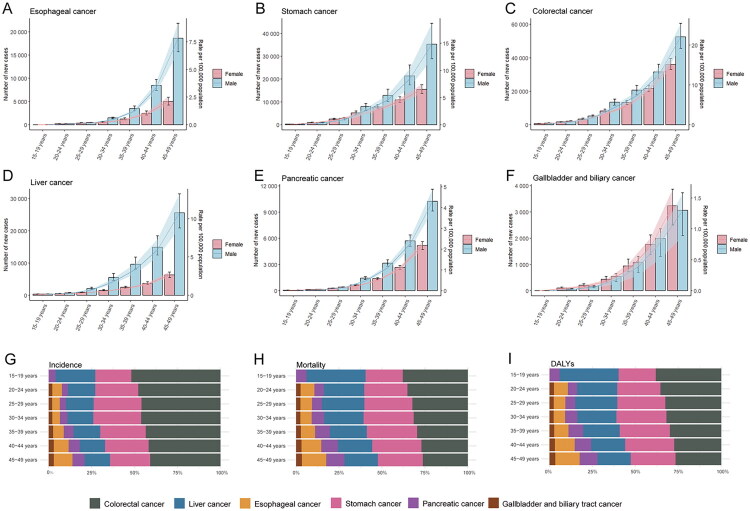
Global burden of age-specific early-onset gastrointestinal cancers in 2021. (A–F) Global age-specific counts and rates of incident numbers and rates by sex. (G–I) Global proportion of age-specific rates.

The ASIR, ASMR, and ASDR of early-onset gastrointestinal cancers across five-year age groups in 2021 are shown in Figures 2 and S3. Analysis revealed progressively increasing incidence, mortality, and DALY rates with advancing age in both sexes. Notably, the disease burden for gallbladder and biliary tract cancers increased more significantly in females than in males, while for other cancer types, the burden was more pronounced in males. The distribution of these cancers varies across different age groups is depicted in Figure 2. Early-onset colorectal cancer was the most common cancer across all age groups, though its relative burden decreased with age. Temporal trends from 1990 to 2021 demonstrated consistent patterns in incidence, mortality, and DALY rates across age groups without significant intergroup differences (Figure S4). Sensitivity analyses with alternative age stratification (15–29 years, 30–44 years, 45–49 years) demonstrated the highest incidence, mortality, and DALY rates in the 45–49-year age group. Early-onset esophageal cancer specifically exhibited the highest incidence, deaths, and DALYs numbers in the 45–49 age group, whereas the other early-onset gastrointestinal cancers displayed the maximal cases in the 30–44 age stratum (Figure S5). Temporal analysis from 1990 to 2021 revealed no significant differences in trends of rates and counts for incidence, mortality, and DALYs across redefined age subgroups (Figure S6).

### 3.2. Early-onset esophageal cancer

In 2021, the global ASIR, ASMR, and ASDR for early-onset esophageal cancer were 1.03 (95%UI: 0.91–1.16), 0.79 (95%UI: 0.70–0.90), and 37.52 (95%UI: 33.33–42.37), respectively (Table 1). From 1990 to 2021, significant declines were observed in ASIR (AAPC: −1.90, 95%CI: −2.09 to −1.72), ASMR (AAPC: −2.32, 95%CI: −2.55 to −2.10), and ASDR (AAPC: −2.31, 95%CI: −2.53 to −2.09) (Figure 1, Figures S1, S2, and Tables S4–S6).

**Figure 3.**
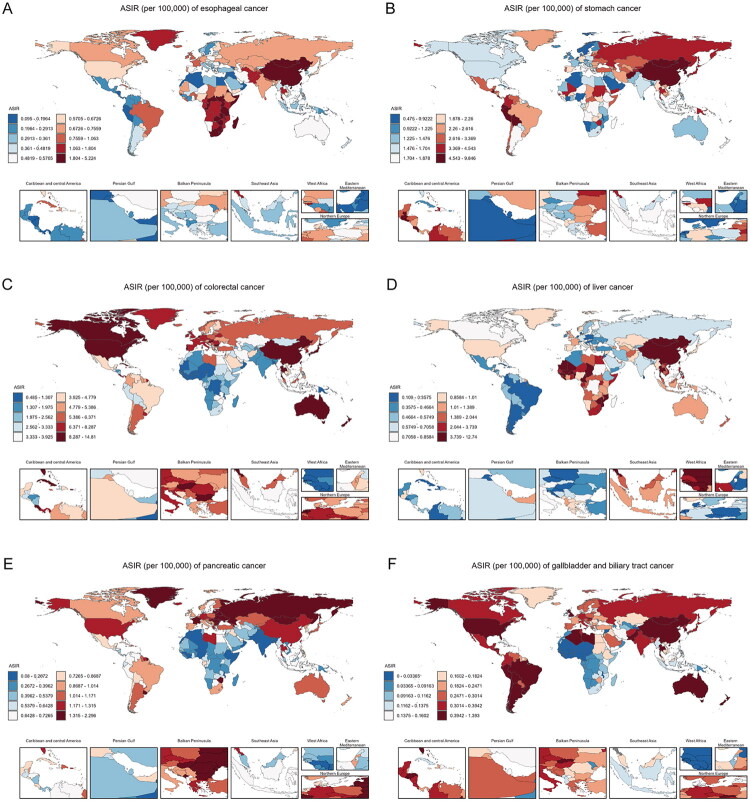
ASIR of early-onset gastrointestinal cancers in both sexes in 204 countries and territories in 2021. ASIR, age-standardized incidence rate.

**Figure 4.**
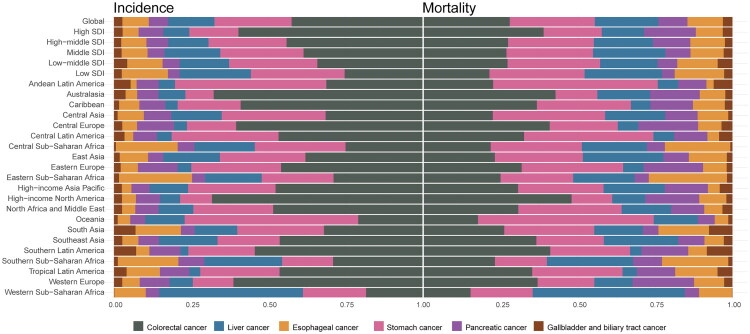
Region-specific proportion of ASIR and ASMR in 2021. ASIR: age-standardized incidence rate. ASMR, age-standardized mortality rate.

At the national level, Malawi reported the highest ASIR (5.22, 95%UI: 3.49–7.85) and ASMR (4.80, 95%UI: 3.21–7.03), while Mongolia had the highest ASDR (91.80, 95%UI: 65.04–123.51) in 2021 (Figures 3, S7 and S8, Tables S7–9). The countries with the most pronounced percentage change in ASIR, ASMR, and ASDR were Thailand (1.85, 95% UI: 0.75–3.56), Liberia (1.52, 95% UI: 0.59–2.99), and Liberia (1.49, 95% UI: 0.58–2.92), respectively (Tables S10–S12). Regionally, early-onset esophageal cancer was most common in Eastern Sub-Saharan Africa, followed by Central Sub-Saharan Africa and Southern Sub-Saharan Africa (Figures 4, S9, and Table S13). From 1990 to 2021, Western Sub-Saharan Africa experienced the most pronounced increases in ASIR (AAPC: 1.38, 95%CI: 1.21–1.55), ASMR (AAPC: 1.36, 95%CI: 1.20–1.53), and ASDR (AAPC: 1.38, 95%CI: 1.21–1.55). Stable trends were observed in Australasia (ASIR: AAPC: 0.31, 95%CI: −0.12 to 0.74; ASMR: AAPC: −0.04, 95%CI: −0.48 to 0.39; ASDR: AAPC: −0.06, 95%CI: −0.49 to 0.38), Caribbean (ASIR: AAPC: −0.02, 95%CI: −0.46 to 0.43; ASMR: AAPC: −0.09, 95%CI: −0.54 to 0.36; ASDR: AAPC: −0.12, 95%CI: −0.56 to 0.32), and High-income North America (ASIR: AAPC: −0.16, 95%CI: −0.35 to 0.04; ASMR: AAPC: −0.51, 95%CI: −1.04 to 0.02; ASDR: AAPC: −0.44, 95%CI: −0.98 to 0.11). Notably, Central Asia exhibited significant declines of ASIR (AAPC: −3.51, 95%CI: −4.01 to −3.01), ASMR (AAPC: −3.55, 95%CI: −4.05 to −3.05), and ASDR (AAPC: −3.52, 95%CI: −4.02 to −3.02) (Tables S4–6).

Among regions categorized by SDI, the high-middle SDI region reported the highest ASIR (1.43, 95%UI: 1.15–1.81), ASMR (0.99, 95%UI: 0.80–1.25), and ASDR (46.71, 95%UI: 37.49–58.63) (Table S14). Correlation analysis revealed a non-linear, inverse relationship between SDI and disease burden, with ASIR, ASMR, and ASDR showing a fluctuating decline as SDI increased, indicating a complex relationship between socio-demographic development and disease burden (Figure S10).

### 3.3. Early-onset stomach cancer

In 2021, the global ASIR, ASMR, and ASDR for early-onset stomach cancer were quantified as 3.04 (95%UI: 2.61–3.52), 1.92 (95%UI: 1.66–2.20), and 94.03 (95%UI: 81.42–107.52), respectively (Table 1). From 1990 to 2021, the ASIR (AAPC: −2.32, 95%CI: −2.41 to −2.22), ASMR (AAPC: −2.87, 95%CI: −3.04 to −2.71), and ASDR (AAPC: −2.87, 95%CI: −3.06 to −2.68) all showed a decline trend (Figures 1, S^1^, S2, and Tables S4–6).

Afghanistan had the highest ASIR (9.85, 95%UI: 4.81 to 15.50), ASMR (8.90, 95%UI: 4.30–13.90), and ASDR (437.21, 95%UI: 210.54 to 683.55) (Figures 3, S7, 8, and Tables S7–9). From 1990 to 2021, Zimbabwe recorded the most pronounced percentage change in ASIR (0.96, 95% UI: 0.24–1.91), ASMR (0.99, 95% UI: 0.25–1.96), and ASDR (0.98, 95% UI: 0.25–1.92) (Tables S10–12). Significant geographical disparities were observed across the 21 global regions. Oceania had relatively higher ASRs for early-onset stomach cancer (Figure 4, Figure S9, Table S13). From 1990 to 2021, all regions saw reductions in ASIR, ASMR, and ASDR, with High-income Asia-Pacific exhibiting particularly notable declines (ASIR: AAPC: −3.91, 95%CI: −4.20 to −3.62; ASMR: AAPC: −4.85, 95%CI: −5.09 to −4.61; ASDR: AAPC: −4.85, 95%CI: −5.08 to −4.62) (Tables S4–6).

SDI subgroups analysis revealed the high-middle SDI region had the highest rates of ASIR (4.63, 95%UI: 3.74–5.62), ASMR (2.55, 95%UI: 2.09–3.04), and ASDR (124.82, 95%UI: 102.43–148.52) (Table S14). The trends in ASIR differed from those of ASMR and ASDR based on SDI: regions with SDI below 0.7 exhibited positive correlations between SDI increases and ASRs, whereas regions above SDI 0.7 showed stabilized ASIR accompanied by continued ASMR and ASDR (Figure S10).

### 3.4. Early-onset colorectal cancer

Among all early-onset gastrointestinal cancers, colorectal cancer demonstrated the most substantial disease burden in 2021, with ASIR, ASMR, and ASDR reaching 5.15 (95%UI: 4.68–5.67), 1.93 (95%UI: 1.76–2.11), and 97.68 (95%UI: 88.78–106.85), respectively (Table 1). Between 1990 and 2021, the ASIR exhibited a gradual increase (AAPC: 0.37, 95%CI: 0.24–0.50), while both ASMR (AAPC: −0.86, 95%CI: −1.00 to −0.72) and ASDR (AAPC: −0.84, 95%CI: −0.98 to −0.70) showed consistent declines (Figure 1, Figures S1, S2, and Tables S4–S6).

Nationally, Monaco displayed the highest ASIR (14.81, 95%UI: 9.05–22.49), whereas Seychelles recorded peak ASMR (3.90, 95%UI: 2.87–5.17) and ASDR (196.11, 95%UI: 144.11–260.82) (Figures 3, S7, S8, and Tables S7–S9). Costa Rica demonstrated the most substantial percentage change in ASIR (2.85, 95% UI: 2.06–3.86), ASMR (1.71, 95% UI: 1.20–2.38), and ASDR (1.69, 95% UI: 1.18–2.35) during the past 30 years (Tables S10–S12). Early-onset colorectal cancer predominated as the most frequent gastrointestinal cancer across most regions, particularly in High-income North America and Australasia (Figures 4, S9, and Table S13). From 1990 to 2021, Central Latin America saw the most significant increases in ASIR (AAPC: 2.56, 95%CI: 2.28–2.85), ASMR (AAPC: 1.39, 95%CI: 1.07–1.72), and ASDR (AAPC: 1.39, 95%CI: 1.07–1.72). Conversely, Central Asia recorded the most pronounced reductions of ASIR (AAPC: −1.10, 95%CI: −1.60 to −0.59) and ASDR (AAPC: −1.67, 95%CI: −2.11 to −1.22), while Western Europe exhibited the largest ASMR decrease (AAPC: −1.67, 95%CI: −1.88 to −1.46) (Tables S4–S6).

In terms of the SDI, the high SDI region exhibited the highest ASIR at 8.43 (95%UI: 8.08–8.79), while the high-middle SDI region reported the highest ASMR at 2.53 (95%UI: 2.20–2.93) and ASDR at 129.34 (95%UI: 112.40–150.01) (Table S14). A positive correlation was observed between ASIR and SDI, indicating an upward trend as SDI increased. In regions with an SDI below 0.7, both ASMR and ASDR showed a positive association with SDI. However, in regions with an SDI above 0.7, these rates exhibited a decline as SDI increased (Figure S10).

### 3.5. Early-onset liver cancer

Globally, the ASIR, ASMR, and ASDR for early-onset liver cancer were 1.82 (95%UI: 1.57–2.16), 1.43 (95%UI: 1.23–1.69), and 70.53 (95%UI: 60.87–83.05), respectively (Table 1). Over the past three decades, significant downward trends were observed in ASIR (AAPC: −0.61, 95%CI: −0.68 to −0.54), ASMR (AAPC: −0.97, 95%CI: −1.16 to −0.79), and ASDR (AAPC: −1.01, 95%CI: −1.19 to −0.82) (Figures 1, S1, S2, and Tables S4–S6).

At the national level, Eswatini had the highest ASIR (8.54, 95%UI: 2.29–22.86) and ASMR (7.88, 95%UI: 2.09–21.06), while Mongolia showed the highest ASDR (569.50, 95%UI: 372.17–841.8) for early-onset liver cancer in 2021 (Figures 3, S7, S8, and Tables S7–S9). From 1990 to 2021, Lesotho exhibited the largest percentage change in ASIR (2.70, 95% UI: −0.04 to 17.26) and ASDR (2.67, 95% UI: −0.07 to 16.89), while Poland showed the highest increase in ASMR (2.35, 95% UI: 1.84–2.89) (Tables S10–S12). Among all regions, Western Sub-Saharan Africa stands out for having the highest proportion of ASRs of early-onset liver cancer (Figures 4, S9, and Table S13). Australasia had the most significant increases in ASIR (AAPC: 2.97, 95%CI: 2.87–3.07), ASMR (AAPC: 2.29, 95%CI: 1.74–2.84), and ASDR (AAPC: 2.27, 95%CI: 1.75–2.80). In contrast, the High-income Asia Pacific experienced the largest declines in ASIR (AAPC: −2.08, 95%CI: −2.19 to −1.96), ASMR (AAPC: −3.30, 95%CI: −3.76 to −2.85), and ASDR (AAPC: −3.31, 95%CI: −3.77 to −2.84) (Tables S4–S6).

Regarding SDI subgroups, the highest ASIR (2.39, 95%UI: 1.93–3.01), ASMR (1.86, 95%UI: 1.52–2.32), and ASDR (91.67, 95%UI: 75.10–113.85) were observed in the middle SDI region (Table S14). Correlation analysis revealed an inverse relationship between ASRs and SDI. However, in regions with the highest SDI, an upward trend in these metrics was observed (Figure S10).

### 3.6. Early-onset pancreatic cancer

In 2021, the global ASRs of early-onset pancreatic cancer were 0.76 (95%UI: 0.69–0.84), 0.65 (95%UI: 0.59–0.72), and 31.16 (95%UI: 28.24–34.29) in terms of incidence, mortality, and DALYs, respectively (Table 1). From 1990 to 2021, declining trends were observed in ASIR (AAPC: −0.35, 95%CI: −0.49 to −0.21), ASMR (AAPC: −0.44, 95%CI: −0.59 to −0.28), and ASDR (AAPC: −0.44, 95%CI: −0.60 to −0.28) (Figures 1, S^1^, S2, and Tables S4–S6).

Greenland reported the highest ASIR (2.30, 95%UI: 1.57–3.19), ASMR (2.02, 95%UI: 1.39–2.83), and ASDR (93.84, 95%UI: 64.02–131.26) for early-onset pancreatic cancer in 2021(Figures 3, S^7^, S8, and Tables S7–S9). Between 1990 and 2021, Turkmenistan exhibited the largest percentage change in ASIR (23.19, 95%UI: 16.26–32.27), ASMR (23.09, 95%UI: 16.21–32.19), and ASDR (22.26, 95%UI: 15.70–31.02) (Tables S10–12). Despite these national variations, early-onset pancreatic cancer remains relatively uncommon in most parts of the region (Figures 4, S^9^, and Table S13). Trends in disease burden varied significantly across regions between 1990 and 2021. Western Sub-Saharan Africa experienced the most significant increase in ASIR (AAPC: 1.87, 95%CI: 1.72–2.01), ASMR (AAPC: 1.84, 95%CI: 1.69–1.98), and ASDR (AAPC: 1.85, 95%CI: 1.69–2.01), while Central Europe saw the largest decline in ASIR (AAPC: −0.85, 95%CI: −1.01 to −0.69), ASMR (AAPC: −0.91, 95%CI: −1.07 to −0.75), and ASDR (AAPC: −0.93, 95%CI: −1.09 to −0.77) (Tables S4–S6).

The highest ASIR (1.26, 95%UI: 1.10–1.45), ASMR (1.09, 95%UI: 0.94–1.25), and ASDR (52.10, 95%UI: 45.23–59.78) for early-onset pancreatic cancer were observed in high-middle SDI region (Table S14). Correlation analysis between ASRs and SDI demonstrated that ASIR, ASMR, and ASDR initially increased with rising SDI, peaking at an SDI of 0.7, followed by a decline as SDI further increased (Figure S10).

### 3.7. Early-onset gallbladder and biliary tract cancer

In 2021, early-onset gallbladder and biliary tract cancer recorded an ASIR of 0.33 (95%UI: 0.26–0.38), an ASMR of 0.21 (95%UI: 0.17–0.25), and an ASDR of 10.20 (95%UI: 8.04–11.95) (Table 1). Over the past three decades, significant declines were observed in ASIR (AAPC: −0.39, 95%CI: −0.57 to −0.22), ASMR (AAPC: −1.05, 95%CI: −1.10 to −1.00), and ASDR (AAPC: −1.03, 95%CI: −1.08 to −0.98) (Figures 1, S1, S2, and Tables S4–6).

Nationally, Thailand reported the highest ASIR (1.39, 95%UI: 0.68 to 2.09), ASMR (0.89, 95%UI: 0.44–1.34), and ASDR (42.66, 95%UI: 20.96 to 63.68) in 2021 (Figures 3, S7, S8, and Tables S7–9). Over the period from 1990 to 2021, the most substantial percentage change in ASIR was observed in Iran (Islamic Republic of) (1.58, 95%UI: 0.45–2.66), while Uzbekistan experienced the largest percentage rises in ASMR (1.41, 95%UI: 0.58–2.59) and ASDR (1.37, 95%UI: 0.56–2.52) (Tables S10–12). Across regions, early-onset gallbladder and biliary tract cancer contributed the least to the overall disease burden (Figures 4, S9, and Table S13). Australasia experienced the most pronounced increase in ASIR (AAPC: 0.95, 95%CI: −0.38 to 2.30), while South Asia was the only region with increases in ASMR (AAPC: 0.45, 95%CI: 0.38–0.53) and ASDR (AAPC: 0.46, 95%CI: 0.32–0.62). Conversely, High-income Asia Pacific exhibited the most notable declines in ASIR (AAPC: −2.35, 95%CI: −2.63 to −2.08), ASMR (AAPC: −3.24, 95%CI: −3.50 to −2.99), ASDR (AAPC: −3.21, 95%CI: −3.46 to −2.97) (Tables S4–S6).

When assessed by SDI levels, the high-middle SDI region showed the highest ASIR (0.42, 95%UI: 0.29–0.51), while the low-middle SDI region had the highest ASMR (0.25, 95%UI: 0.20–0.32) and ASDR (12.23, 95%UI: 9.48–15.24) (Table S14). The relationship between region-level rates and SDI is illustrated in Figure S10. ASIR, ASMR, and ASDR increased with SDI up to a threshold of 0.7. Beyond this threshold, ASIR continued to rise gradually, while ASMR and ASDR began to decline.

### 3.8. Risk factors for early-onset gastrointestinal cancers

Comparative analysis of risk factor contributions to ASMR of early-onset gastrointestinal cancers revealed significant temporal shifts between 1990 and 2021 (Figure S11). Smoking was a contributor to mortality from most early-onset gastrointestinal cancers, affecting esophageal, stomach, colorectal, and liver cancers. However, its relative contribution has shown a decrease over the years. High body mass index (BMI) showed increases in its mortality impact, particularly for early-onset colorectal, pancreatic, liver, and gallbladder and biliary tract cancers. Dietary risk factors were the primary mortality determinant for early-onset colorectal cancer. Figure S12 provides further insight into the distribution of risk factors across different SDI regions in 2021, showing that smoking, high BMI, and alcohol have a higher impact in middle and high-middle SDI regions for most early-onset gastrointestinal cancers.

### 3.9. Predictions of early-onset gastrointestinal cancers from 2022 to 2036

**Figure 5.**
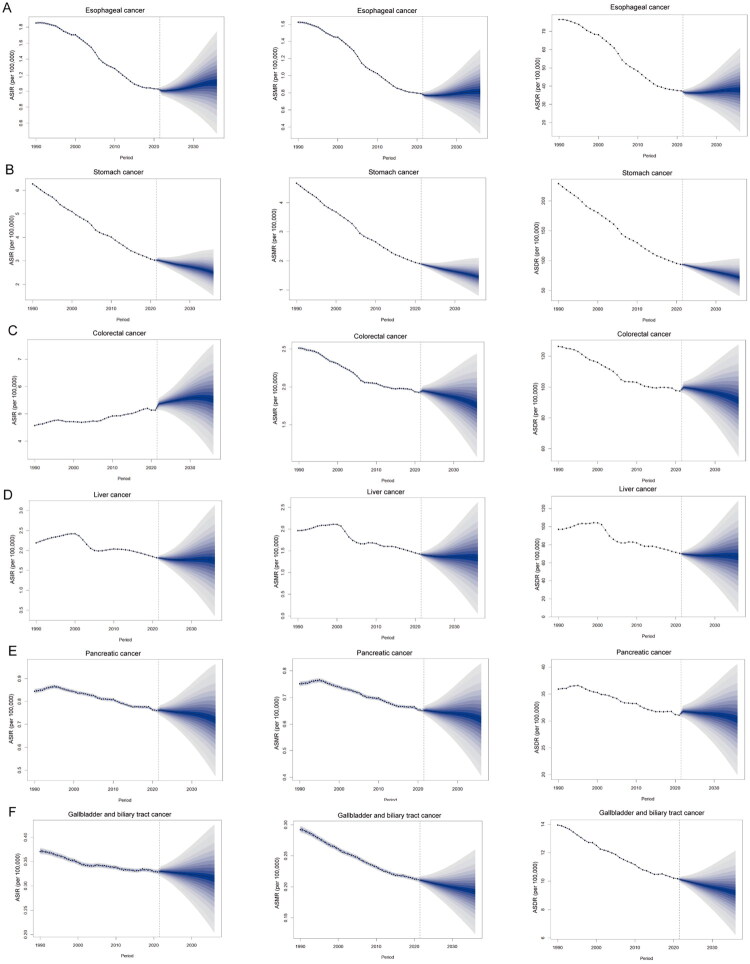
Forecasts of ASIR, ASMR, and ASDR of early-onset gastrointestinal cancers over the next 15 years using the BAPC model. ASIR, age-standardized incidence rate. ASMR, age-standardized mortality rate. ASDR, age-standardized disability-adjusted life years rate. BAPC, Bayesian age-period-cohort.

Projections indicate divergent epidemiological trajectories across early-onset gastrointestinal cancers during the 2022–2036 period. The incidence, mortality, and DALYs rates of early-onset stomach, pancreatic, and gallbladder and biliary tract cancers are projected to decline. In contrast, early-onset esophageal cancer is expected to exhibit increases in incidence, mortality, and DALY rates, with ASIR, ASMR, and ASDR projected to reach 1.33 (95%UI: 0.94–1.72), 0.98 (95%UI: 0.68–1.28), and 37.91 (95%UI: 26.16–49.66), respectively, by 2036. For early-onset colorectal cancer, ASIR is anticipated to continue rising, whereas ASMR and ASDR will maintain a downward trend. Nevertheless, early-onset colorectal cancer is expected to remain the heaviest disease burden by 2036, with ASIR, ASMR, and ASDR projected at 5.53 (95%UI: 4.49–6.57), 1.76 (95%UI: 1.41–2.11), and 91.18 (95%UI: 72.62–109.74), respectively (Figure 5 and Table S15).

## 4. Discussion

This study represents the first comprehensive analysis of the global burden of early-onset gastrointestinal cancers from 1990 to 2021. We confirmed the substantial burden of early-onset gastrointestinal cancer and identify an upward trend in ASIR for colorectal cancer alongside a decline in ASMR and ASDR for all types of cancers. Currently, early-onset colorectal cancer carries a significant impact on global health among all gastrointestinal cancers. The analysis further uncovers significant disparities across age, gender, region, and SDI level, highlighting the urgent need to address modifiable risk factors, particularly the rising prevalence of high BMI and persistent smoking behaviors.

Comparative analysis of ASIR and ASMR from GBD 2021 and GLOBOCAN 2022 revealed strong concordance for overlapping early-onset (15–49 years) gastrointestinal cancers, including esophageal, stomach, colorectal, liver, and pancreatic cancers (Table S16). Discrepancies in early-onset stomach, colorectal, and liver cancer rates likely stem from differences in data sources and processing methods across the databases [31]. SEER registry analyses demonstrated stable or potentially declining incidence trends of early-onset esophageal cancer in the 49–54 age group from 1990 to 2019 [32], with multiple studies consistently reporting decreasing liver cancer incidence among younger populations [33,34]. However, SEER data also revealed a significant rise in the proportion of stomach cancer patients under 50 years of age [9]. Similar trends have been observed in the United Kingdom, Belarus, Chile, the Netherlands, and Canada [35]. Anatomically, stomach cancers are divided into cardia and non-cardia subtypes with distinct clinical and epidemiological features [36]. Non-cardia gastric cancer is common in East Asia, Central Asia, and Eastern Europe and linked to (*H. pylori*) infection [37,38], whereas cardia cancer is more prevalent in North America and Western Europe and associated with obesity [38]. Although our study found an overall global decline in early-onset stomach cancer incidence, the increases in certain countries warrant further investigation to ascertain whether they are driven by rises in cardia, non-cardia, or both subtypes [18]. Sensitivity analyses identified the 45–49 age group as bearing the heaviest ASR burden of gastrointestinal cancers, consistent with SEER findings demonstrating a clustering of early-onset colorectal cancer within this stratum [39]. Screening strategies for early-onset gastrointestinal cancers vary internationally. In 2021, the US Preventive Services Task Force updated guidelines recommending colorectal cancer screening starting at age 45 for individuals at average risk [40]. In contrast, the European Commission’s 2022 guidelines advocate annual fecal immunochemical testing (FIT) for those aged 50–74, reserving colonoscopy for patients with positive fecal tests [41]. Considering the persistent rise in early-onset colorectal cancer, several studies recommend lowering the screening age to 45 years [42]. Others emphasize the need for precise strategies to identify individuals under 45 for early screening, implement systematic population-level screening for ages 45 to 49, allocate substantial resources to boost participation, and ensure appropriate follow-up for individuals aged 50–75 who have not undergone timely colorectal cancer screening [43]. Our findings provide actionable epidemiological evidence to optimize region-specific screening programs for gastrointestinal cancers.

From 1990 to 2021, significant disparities were observed in the disease burden of early-onset gastrointestinal cancers. The observed decline in esophageal and gastric cancer burden aligns with previous epidemiological studies [36,44,45]. This decline may be attributed to reduced prevalence of *H. pylori* infection, improvements in food preservation, and advancements in diagnostic and therapeutic modalities [46]. In contrast, the ASIR of early-onset colorectal cancer has shown a marked increase, which is primarily driven by increase in male, emerging as a leading contributor to the gastrointestinal cancer burden among younger populations. These changes cannot be fully accounted for by screening practices alone and may be related to increased exposure to risk factors during early life and/or adolescence [18], including childhood nutritional deficiencies, radiation exposure, Westernized dietary patterns, adolescent obesity, and physical inactivity [15,47], compounded by birth cohort effects [48]. Furthermore, recent evidence suggests that improved early detection—due to greater public awareness and incidental findings on imaging techniques like ultrasound and CT—plays an important role in this increasing trend [17]. Furthermore, hereditary conditions (known cancer predisposition syndromes, de novo germline mutations, familial colorectal cancer) demonstrate significant associations with early-onset colorectal cancer [49]. Elevated rates of pathogenic variants in cancer-predisposition genes, including MSH2, MSH6, NF1, PTEN, TSC1/TSC2, and BRCA2, are mechanistically associations with early-onset colorectal cancer [48]. Concurrently, microbial dysbiosis characterized by enriched Flavonifractor plauti and increased tryptophan, bile acid, and choline metabolic constitutes established risk factors [50]. The decreasing trend in ASMR and ASDR of early-onset colorectal cancer, as confirmed by data from *CA: A Cancer Journal for Clinicians* [51], can be partially explained by prolonged survival due to enhanced treatment options, widespread implementation of early colonoscopy screenings, and increased public health awareness. For gallbladder and biliary tract cancer, the reduced burden may be attributed to improved sanitation, reduced parasitic infections in some regions [46], and therapeutic advancements [52,53]. Over the past 30 years, the burden of early-onset pancreatic cancer had decreased, potentially linked to treatment options such as precision medicine, targeted therapy, and immunotherapy, significantly increasing survival rate and life quality.

Our findings indicate significant regional disparities in gastrointestinal cancer burden linked to SDI, reflecting variations in dietary and lifestyle factors. The positive SDI-incidence correlation underscores the impact of lifestyle factors prevalent in high SDI regions, including obesity, processed meat consumption, and Western dietary patterns [54]. High-SDI countries generally benefit from robust cancer registries and healthcare systems. Additionally, countries like the United States recommend colorectal cancer screening from age 45, which may elevate early-onset colorectal cancer detection rates [55]. Such factors partially account for the higher ASIR and ASDR in these regions. These innovations facilitate the early detection of cancers, which is critical for improving prognosis and reducing mortality rates. Notably, while mortality and DALYs rates for early-onset stomach, colorectal, pancreatic, and gallbladder and bile tract cancers generally increase with rising SDI, the highest SDI regions demonstrate a decline in these rates. During the last 30 years, regions with higher SDI have experienced a more significant decrease in the incidence of most gastrointestinal cancers, likely due to improved control of risk factors. Public health initiatives focusing on lifestyle changes such as weight loss, enhanced physical activity, dietary improvements, limiting alcohol intake, and controlling viral hepatitis play a vital role in mitigating gastrointestinal cancer risk and its advancement [56]. Conversely, limited healthcare resources in lower SDI regions may lead to underestimation of disease burden, and delays in diagnosis and treatment could exacerbate mortality rates. These regional disparities in disease burden highlight the urgent need for targeted public health interventions and equitable resource allocation to address these inequities effectively.

The escalating impact of high BMI warrants particular attention, with obesity rates having tripled in recent decades [57]. Evidence from multiple murine models indicated that obesity not only promotes cancer progression but also accelerates the development of cancer at younger age groups [57,58]. While the mortality rate of early-onset gastrointestinal cancers attributable to smoking has declined, tobacco use remains a significant contributor to gastrointestinal cancer burden. More than half of gastrointestinal cancers are linked to high BMI, smoking, poor diet, alcohol consumption, and infections [59,60]. High BMI, smoking, and poor diet are major factors in the development of early-onset gastrointestinal cancers [61]. Lifestyle interventions, such as weight loss, increased physical activity, dietary changes and reduced alcohol consumption can effectively lower the risk of early-onset gastrointestinal cancers and slow disease progression [62,63].

Our projections suggest that the ASIR of early-onset colorectal cancer will continue to increase, while the ASMR and ASDR are expected to decline. In contrast, early-onset esophageal cancer is anticipated to exhibit rising trends in ASRs. The projection outcomes align with future trend forecasts for early-onset colorectal and liver cancers from other studies using GBD databases, validating the reliability of BAPC predictions [45,64]. For liver cancer, these rates are projected to remain stable over the same period. These trends highlight that, despite substantial advancements in healthcare, certain early-onset gastrointestinal cancers continue to impose a significant disease burden. The adoption of innovative screening technologies, such as artificial intelligence and metabolomics, offers considerable potential to enhance screening efficacy, facilitate earlier detection, and improve treatment outcomes for digestive cancers, thereby reducing the overall disease burden [65]. Prevention efforts should prioritize targeting modifiable risk factors, including elevated BMI, tobacco use, and unhealthy dietary patterns. In low-SDI regions, resource allocation strategies should emphasize improving access to healthcare services and promoting early diagnostic capabilities. Furthermore, strengthening cancer registries is essential for accurately tracking epidemiological trends, assessing the effectiveness of interventions, and optimizing the monitoring and management of gastrointestinal cancers.

There are several limitations in this study. First, the GBD2021 database’s primary reliance on population registration systems introduces potential data quality discrepancies. Particularly, inputs from regions with less robust registration infrastructure (notably in Asia) could introduce analytical inaccuracies, compounded by potential underdiagnosis and misclassification in low-income countries where limited healthcare capacity intersects with incomplete cancer registry data. Second, the absence of detailed classification and staging information constrains our ability to conduct nuanced analyses of specific cancer subtypes. For example, the inability to differentiate between cardia and non-cardia stomach cancers precludes quantification of subtype-specific burdens. Moreover, *H. pylori*, a key risk factor in stomach cancer, is not included in the GBD data as a risk factor for stomach cancer. Additionally, the GBD database’s aggregation of gallbladder cancer and biliary tract cancer into a single analytical entity may introduce confounding bias. Furthermore, despite emerging evidence indicating the predominant occurrence of early-onset colorectal cancer in the left colon [66], the GBD framework lacks subsite-specific stratification for colorectal malignancies, notably omitting differentiation between anatomically distinct segments. Thirdly, the COVID-19 pandemic significantly disrupted healthcare delivery systems, diagnostic practices, and disease reporting mechanisms. These disruptions may have caused diagnostic delays and distorted burden estimation accuracy during the study period, potentially introducing confounding effects that could bias our findings.

## 5. Conclusion

In conclusion, this study provides a comprehensive overview of global burden and attributable risk factors for early-onset gastrointestinal cancers. In this study, early-onset gastrointestinal cancers had shown varying trends from 1990 to 2021, with colorectal cancer continuing to be the most common and burdensome. The identification of high BMI and persistent smoking as principal modifiable risk factors underscores the critical need for targeted preventive strategies.

## Supplementary Material

Supplementary Material Clear version.docx

## Data Availability

The data were obtained through an online query tool from the website of GBD 2021 (https://ghdx.healthdata.org/gbd-2021/sources), and no permissions were required to access the data.
