# Supplementary material for: Epidemiological trends of early-onset gastrointestinal cancers from 1990 to 2021 and predictions for 2036: analysis from the global burden of disease study 2021
Source: Ann Med. 2025 Sep 5;57(1):2555518. doi: 10.1080/07853890.2025.2555518 (PMC12416015; doi:10.1080/07853890.2025.2555518)
Supplement: Supplementary Material Clear version.docx [file IANN_A_2555518_SM2824.docx]

Contents

[**Figure S1.** Temporal trend of ASMR in burden of early-onset gastrointestinal cancers from 1990 to 2021. 3](#_Toc205657812)

[**Figure S2.** Temporal trend of ASDR in burden of early-onset gastrointestinal cancers from 1990 to 2021. 4](#_Toc205657813)

[**Figure S3.** Global age-specific counts and rates of deaths and DALYs by sex in 2021. 5](#_Toc205657814)

[**Figure S4.** Temporal trend of age-specific counts and rates of incidence, mortality, and DALYs cases, globally, 1990 to 2021. 6](#_Toc205657815)

[**Figure S5.** Global age-specific counts and rates of incidence, deaths, and DALYs across non-uniform age groups (15-29, 30-44, 45-49 years) by sex in 2021. 7](#_Toc205657816)

[**Figure S6.** Temporal trend of age-specific counts and rates of incidence, mortality, and DALYs across non-uniform age groups (15-29, 30-44, 45-49 years), globally, 1990 to 2021. 8](#_Toc205657817)

[**Figure S7.** ASMR of early-onset gastrointestinal cancers in both sexes in 204 countries and territories in 2021. 9](#_Toc205657818)

[**Figure S8.** DALYs of early-onset gastrointestinal cancers in both sexes in 204 countries and territories in 2021. 10](#_Toc205657819)

[**Figure S9.** Region-specific proportion of ASDR in 2021. 11](#_Toc205657820)

[**Figure S10.** ASIR, ASMR, and ASDR for early-onset gastrointestinal cancer for 21 GBD regions by Socio-demographic Index, 1990–2021. 12](#_Toc205657821)

[**Figure S11.** Percentage contribution of risk factors to the mortality rates of early-onset gastrointestinal cancers globally, in 1990 and 2021. 13](#_Toc205657822)

[**Figure S12.** Rankings and rates of risk factors to the ASMR of early-onset gastrointestinal cancers across five SDI regions, in 2021. 14](#_Toc205657823)

[**Table S1.** List of International Classification of Diseases (ICD) codes mapped to gastrointestinal cancers in GBD 2021 15](#_Toc205657824)

[**Table S2.** The Socio-demographic index of 204 countries and territories from 1990 to 2005. 16](#_Toc205657825)

[Table S3. The Socio-demographic index of 204 countries and territories from 2006 to 2021. 17](#_Toc205657826)

[**Table S4.** AAPC of ASIR of early-onset gastrointestinal cancers from 1990 to 2021. 18](#_Toc205657827)

[Table S5. AAPC of ASMR of early-onset gastrointestinal cancers from 1990 to 2021. 20](#_Toc205657828)

[Table S6. AAPC of ASDR of early-onset gastrointestinal cancers from 1990 to 2021. 22](#_Toc205657829)

[**Table S7.** ASIR of early-onset gastrointestinal cancers in both sexes in 204 countries and territories in 2021. 24](#_Toc205657830)

[**Table S8.** ASMR of early-onset gastrointestinal cancers in both sexes in 204 countries and territories in 2021. 32](#_Toc205657831)

[**Table S9.** ASDR of early-onset gastrointestinal cancers in both sexes in 204 countries and territories in 2021. 40](#_Toc205657832)

[**Table S10.** Percentage rates of incidence early-onset gastrointestinal cancers from 1990 to 2021. 48](#_Toc205657833)

[**Table S11.** Percentage rates of death of early-onset gastrointestinal cancers from 1990 to 2021. 55](#_Toc205657834)

[**Table S12.** Percentage rates of DALYs of early-onset gastrointestinal cancers from 1990 to 2021. 62](#_Toc205657835)

[**Table S13.** Region-specific proportion of ASIR, ASMR, and ASDR in 2021. ASIR: age-standardized incidence rate. 69](#_Toc205657836)

[**Table S14.** Age-standardized rates and numbers of incidence, mortality, and DALYs of early-onset gastrointestinal cancers across five SDI regions in 2021. 71](#_Toc205657837)

[**Table S15.** Age-standardized rates of early-onset gastrointestinal cancers projected until 2036 globally. 73](#_Toc205657838)

[**Table S16.** Comparison of ASIR and ASMR of early-onset esophageal, stomach, colorectal, and pancreatic cancers between GBD 2021 and GLOBOCAN 2022 globally. 74](#_Toc205657839)

## **Figure S1.** Temporal trend of ASMR in burden of early-onset gastrointestinal cancers from 1990 to 2021. (A-F) Temporal trend of ASMR in burden of early-onset gastrointestinal cancers, globally, 1990 to 2021. (G) AAPC of ASMR in burden of early-onset gastrointestinal cancers, regionally, 1990 to 2021. ASMR, age-standardized mortality rate. AAPC, average annual percentage changes.


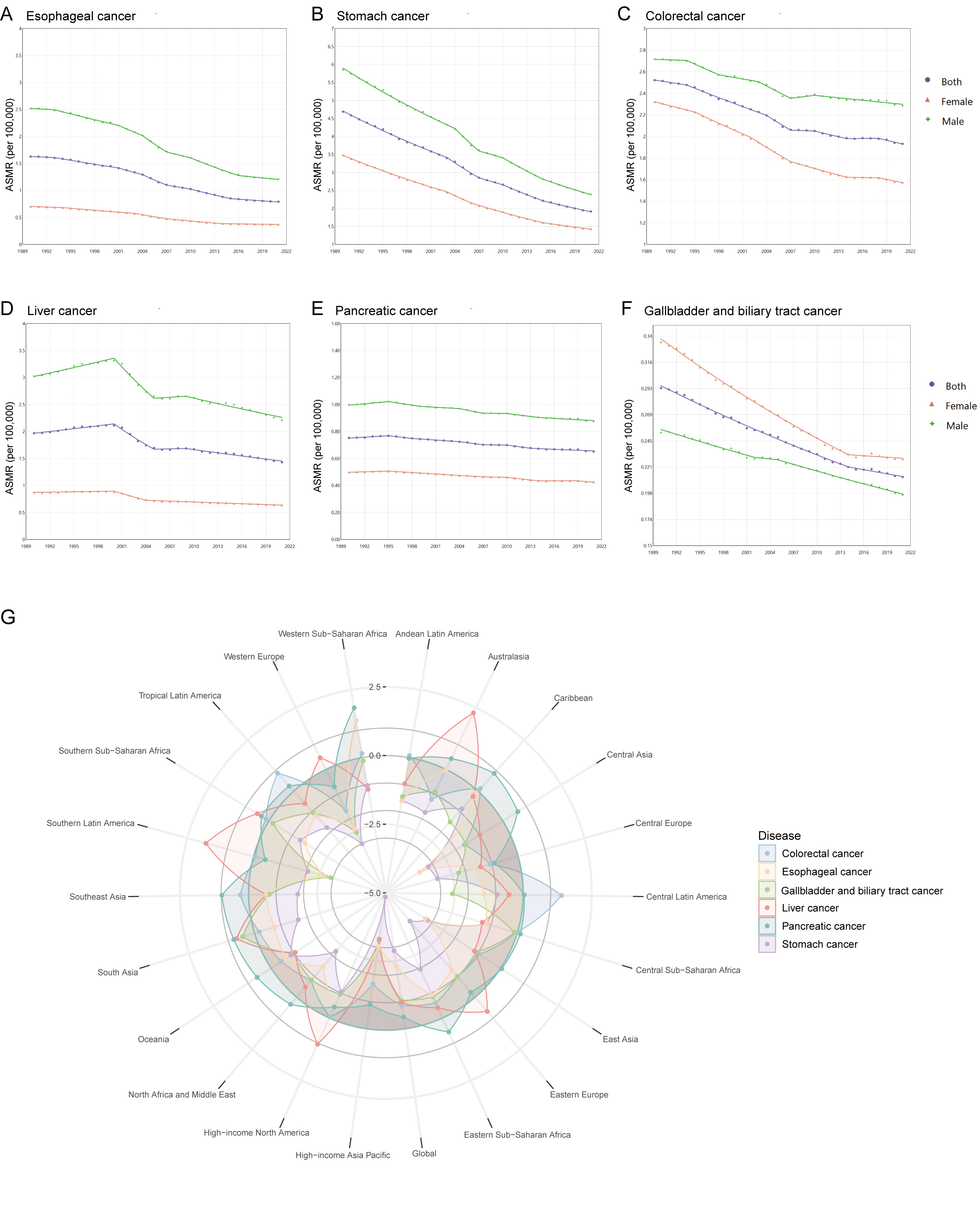


## **Figure S2.** Temporal trend of ASDR in burden of early-onset gastrointestinal cancers from 1990 to 2021. (A-F) Temporal trend of ASDR in burden of early-onset gastrointestinal cancers, globally, 1990 to 2021. (G) AAPC of ASDR in burden of early-onset gastrointestinal cancers, regionally, 1990 to 2021. ASDR, age-standardized disability-adjusted life years rate. AAPC, average annual percentage changes.


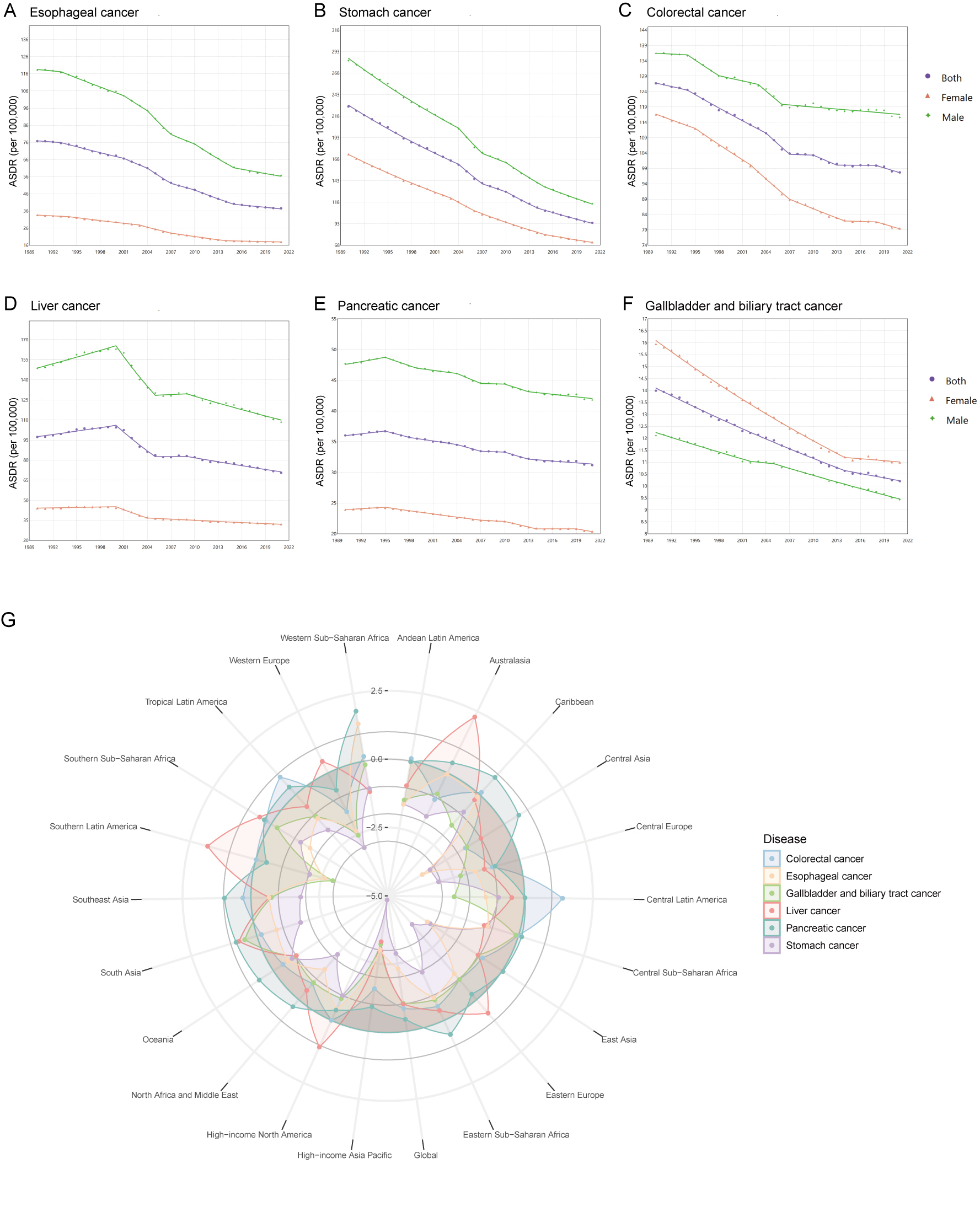


## **Figure S3.** Global age-specific counts and rates of deaths and DALYs by sex in 2021. DALYs, disability-adjusted life years.


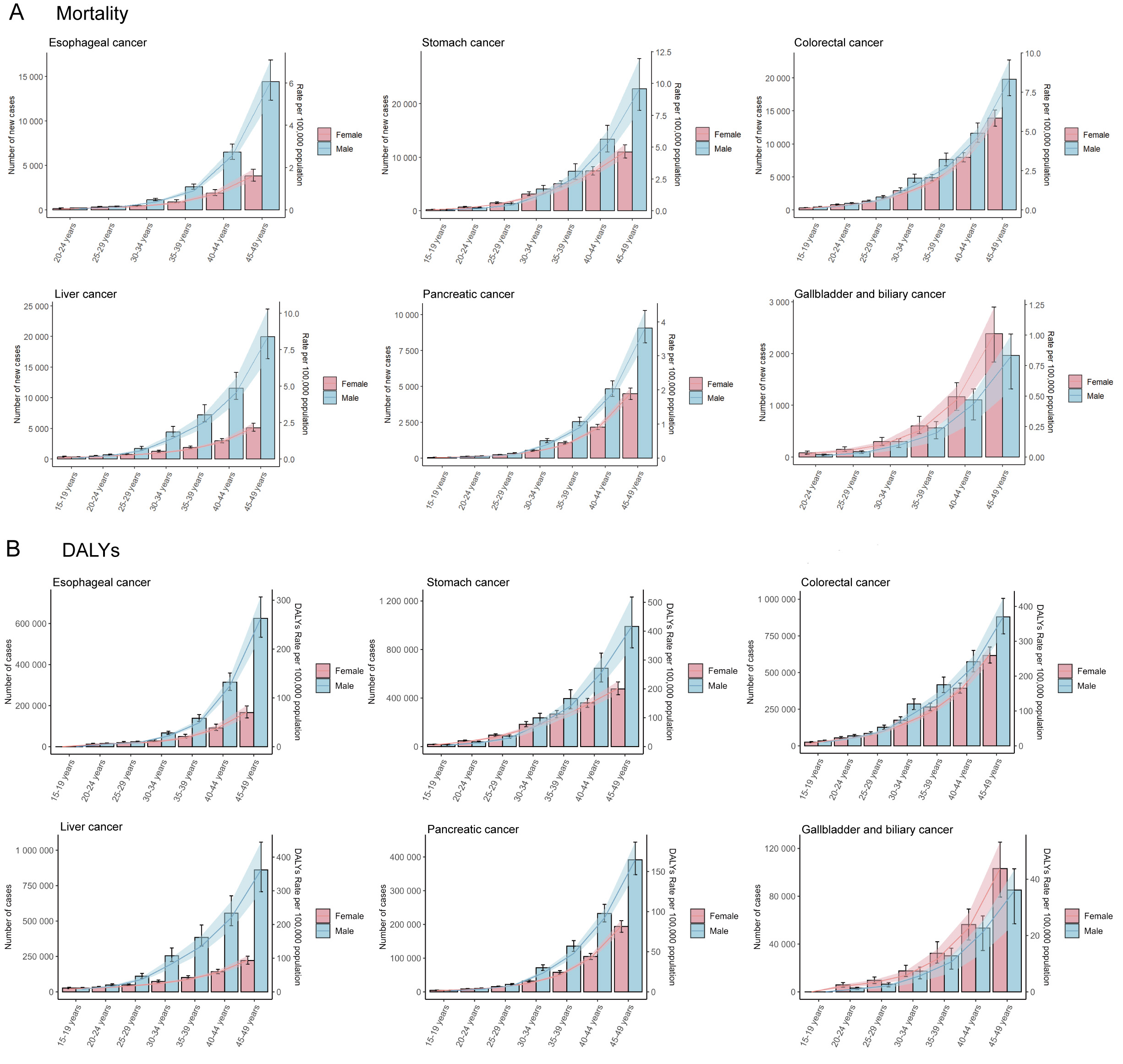


## **Figure S4.** Temporal trend of age-specific counts and rates of incidence, mortality, and DALYs cases, globally, 1990 to 2021. DALYs, disability-adjusted life years.


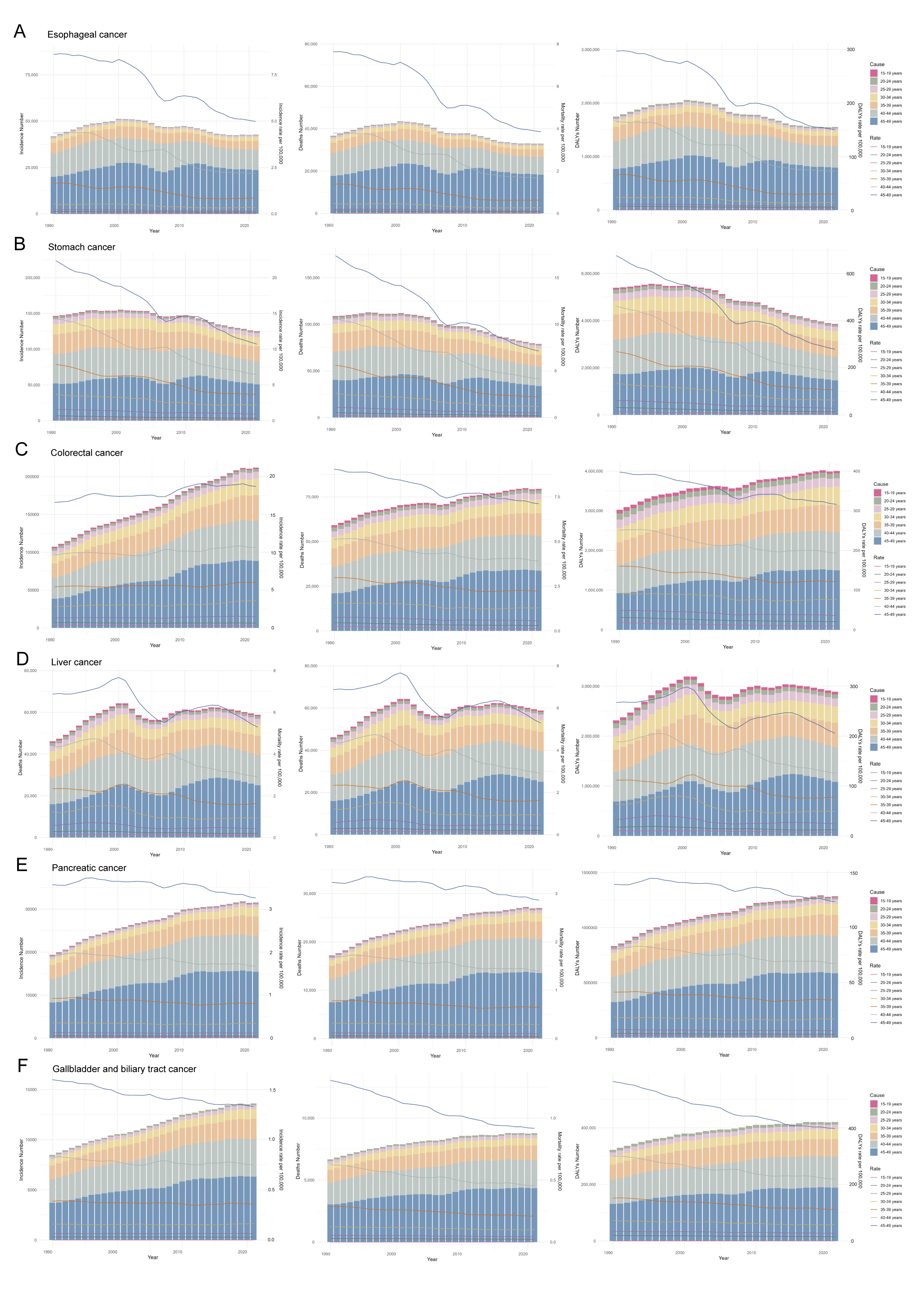


## **Figure S5.** Global age-specific counts and rates of incidence, deaths, and DALYs across non-uniform age groups (15-29, 30-44, 45-49 years) by sex in 2021. DALYs, disability-adjusted life years.


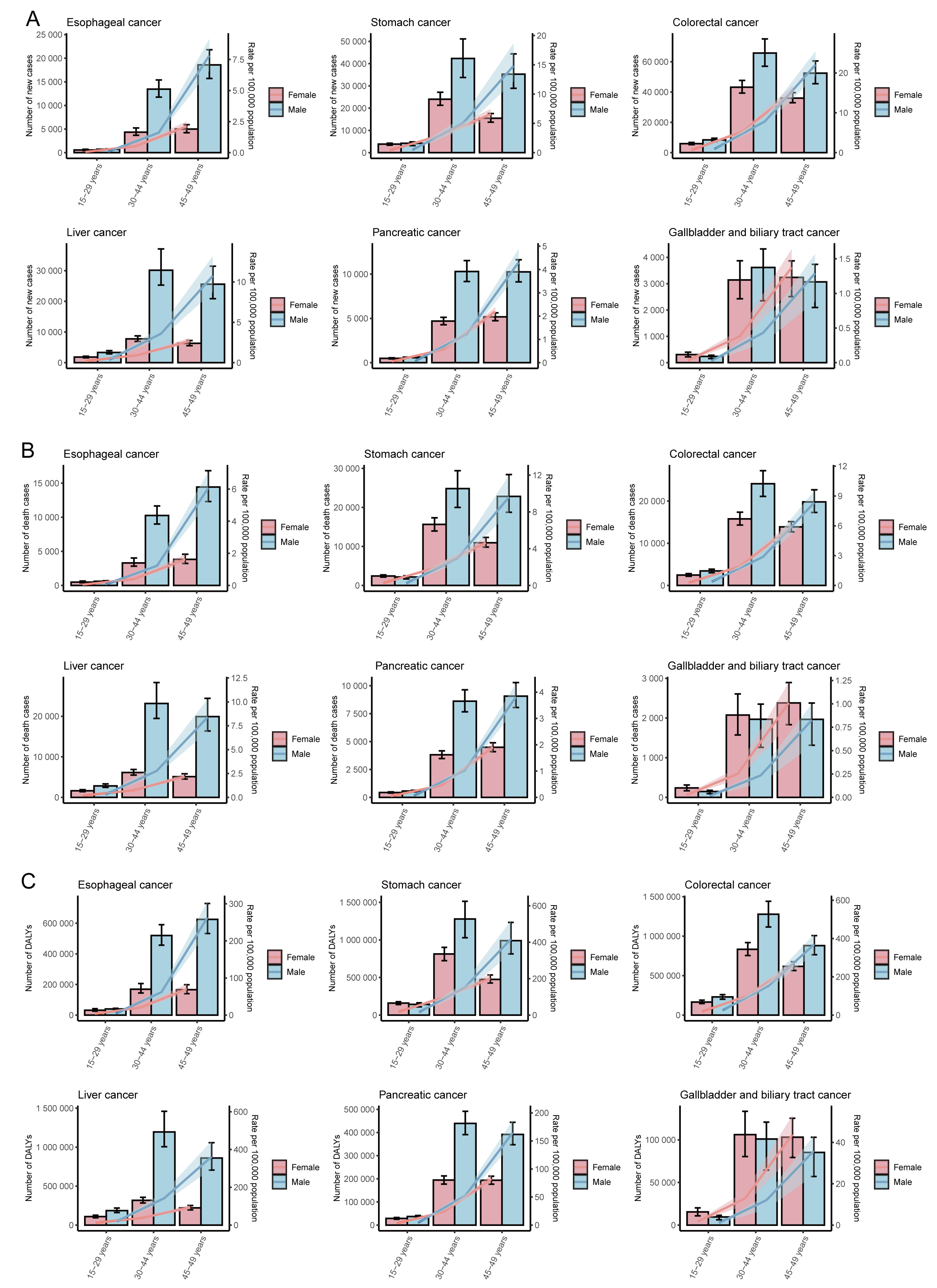


## **Figure S6.** Temporal trend of age-specific counts and rates of incidence, mortality, and DALYs across non-uniform age groups (15-29, 30-44, 45-49 years), globally, 1990 to 2021. DALYs, disability-adjusted life years.


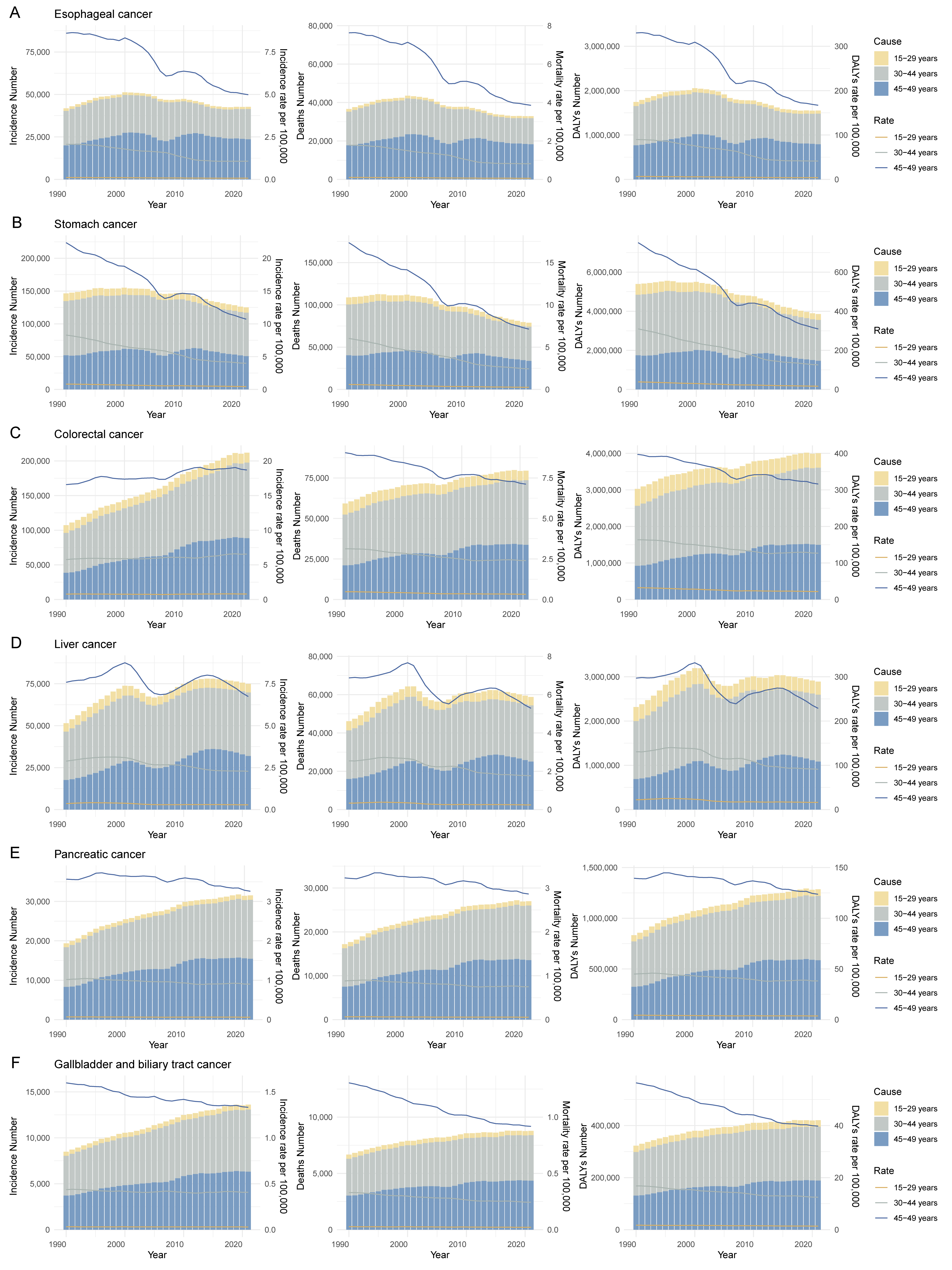


## **Figure S7.** ASMR of early-onset gastrointestinal cancers in both sexes in 204 countries and territories in 2021. ASMR, age-standardized mortality rate.


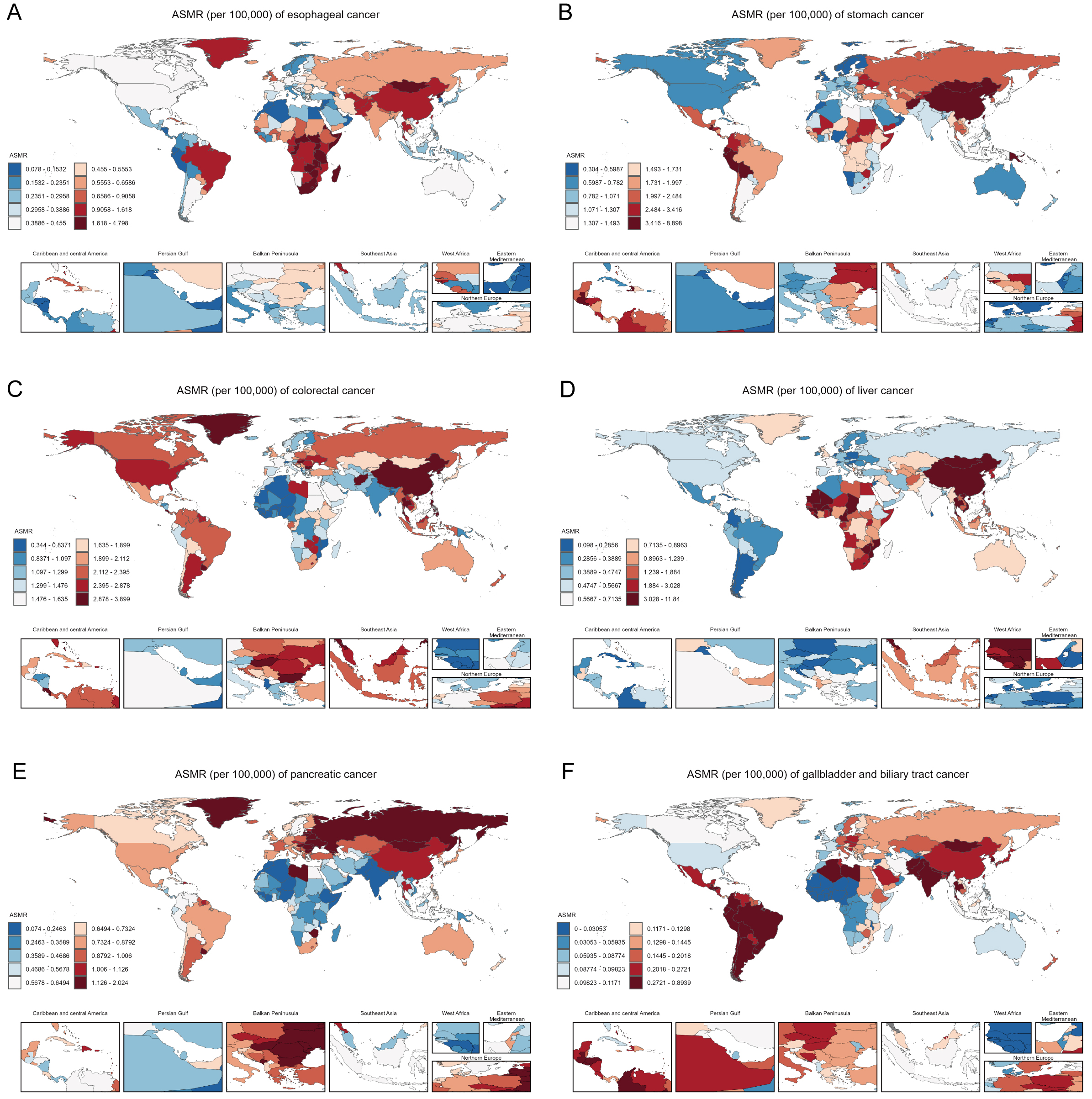


## **Figure S8.** DALYs of early-onset gastrointestinal cancers in both sexes in 204 countries and territories in 2021. ASDR, age-standardized disability-adjusted life years rate.


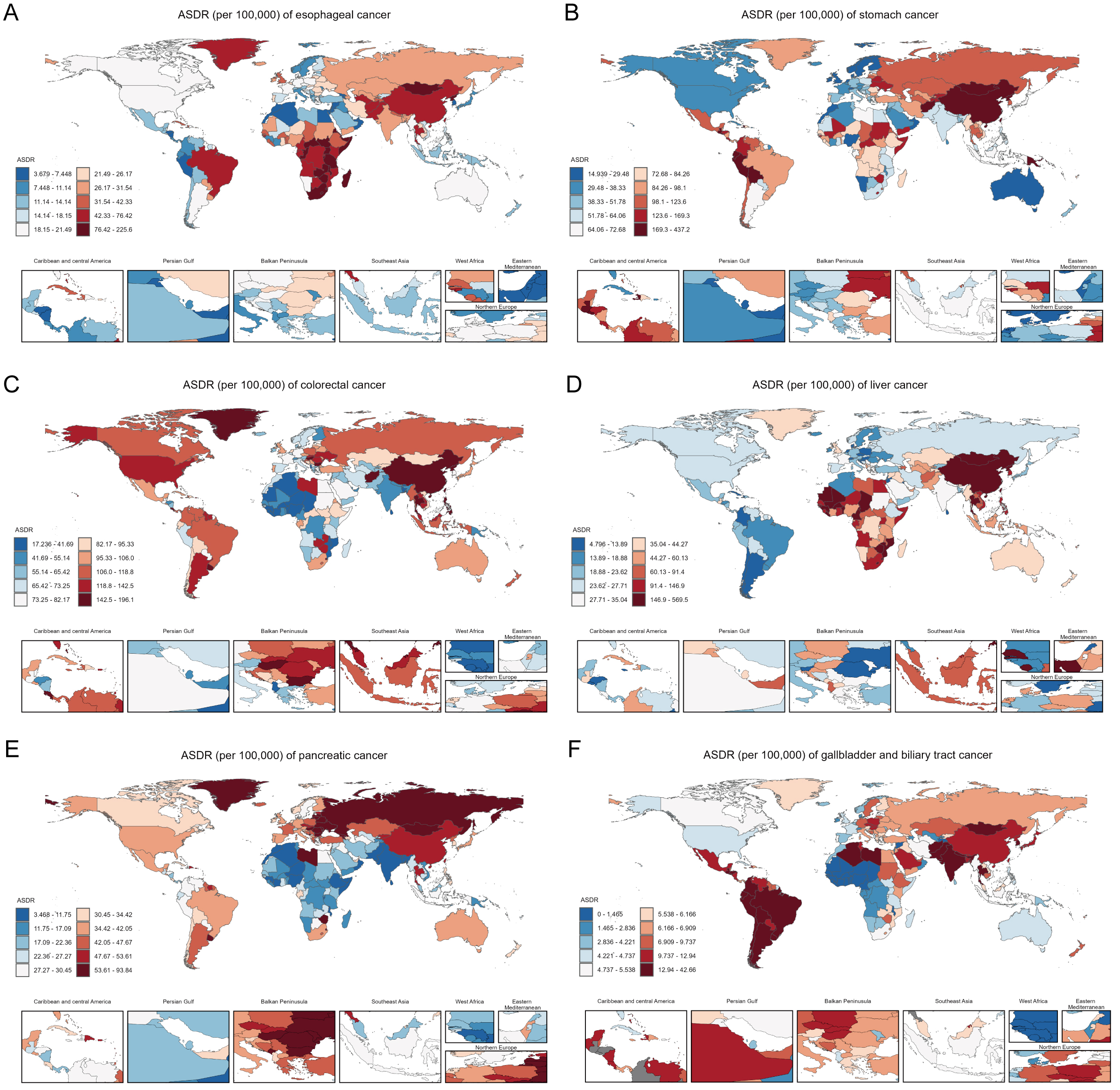


## **Figure S9.** Region-specific proportion of ASDR in 2021. ASDR, age-standardized disability-adjusted life years rate.


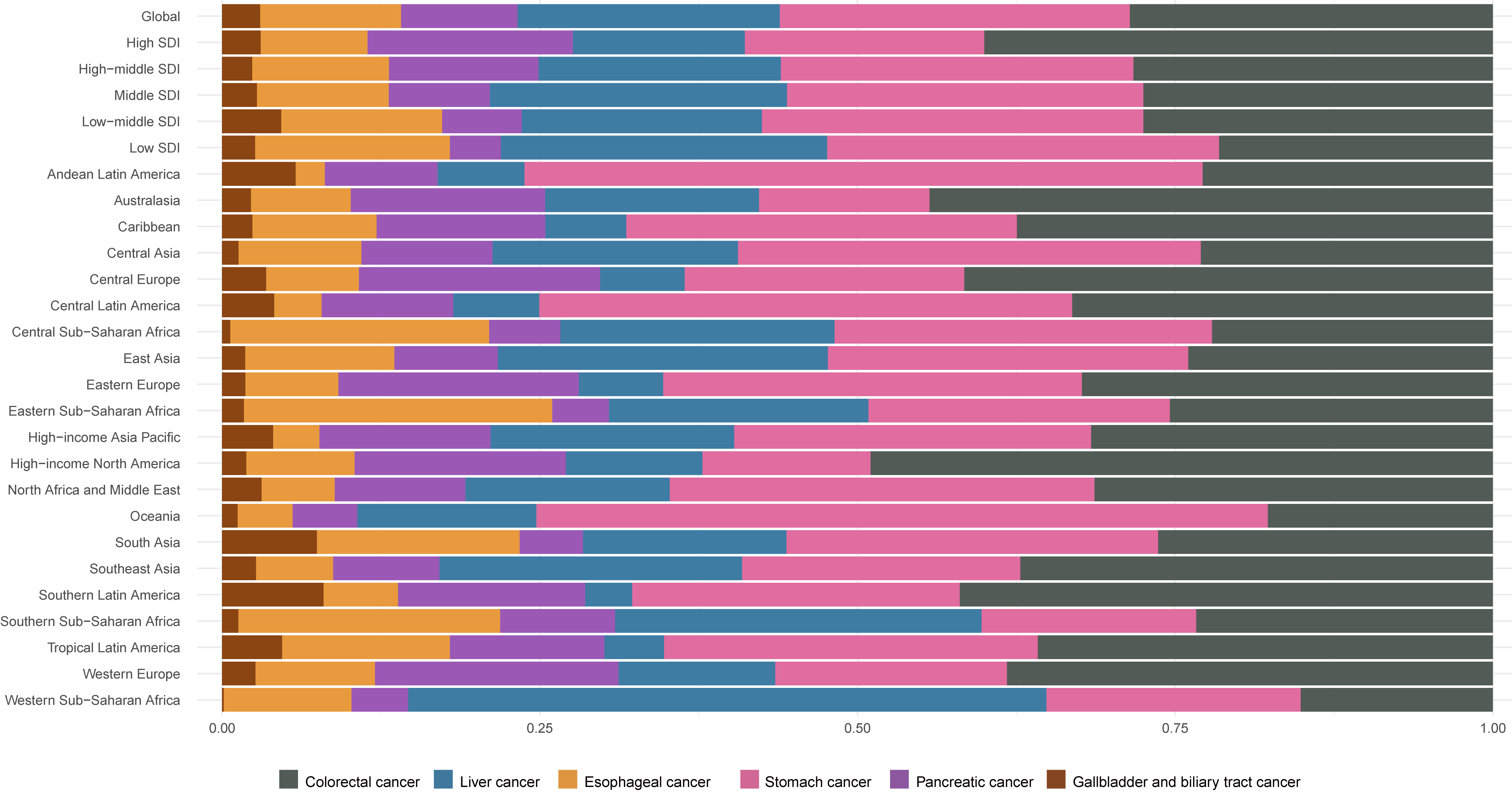


## **Figure S10.** ASIR, ASMR, and ASDR for early-onset gastrointestinal cancer for 21 GBD regions by Socio-demographic Index, 1990–2021. ASIR, age-standardized incidence rate; ASMR, age-standardized mortality rate; ASDR, age-standardized disability-adjusted life years rate.


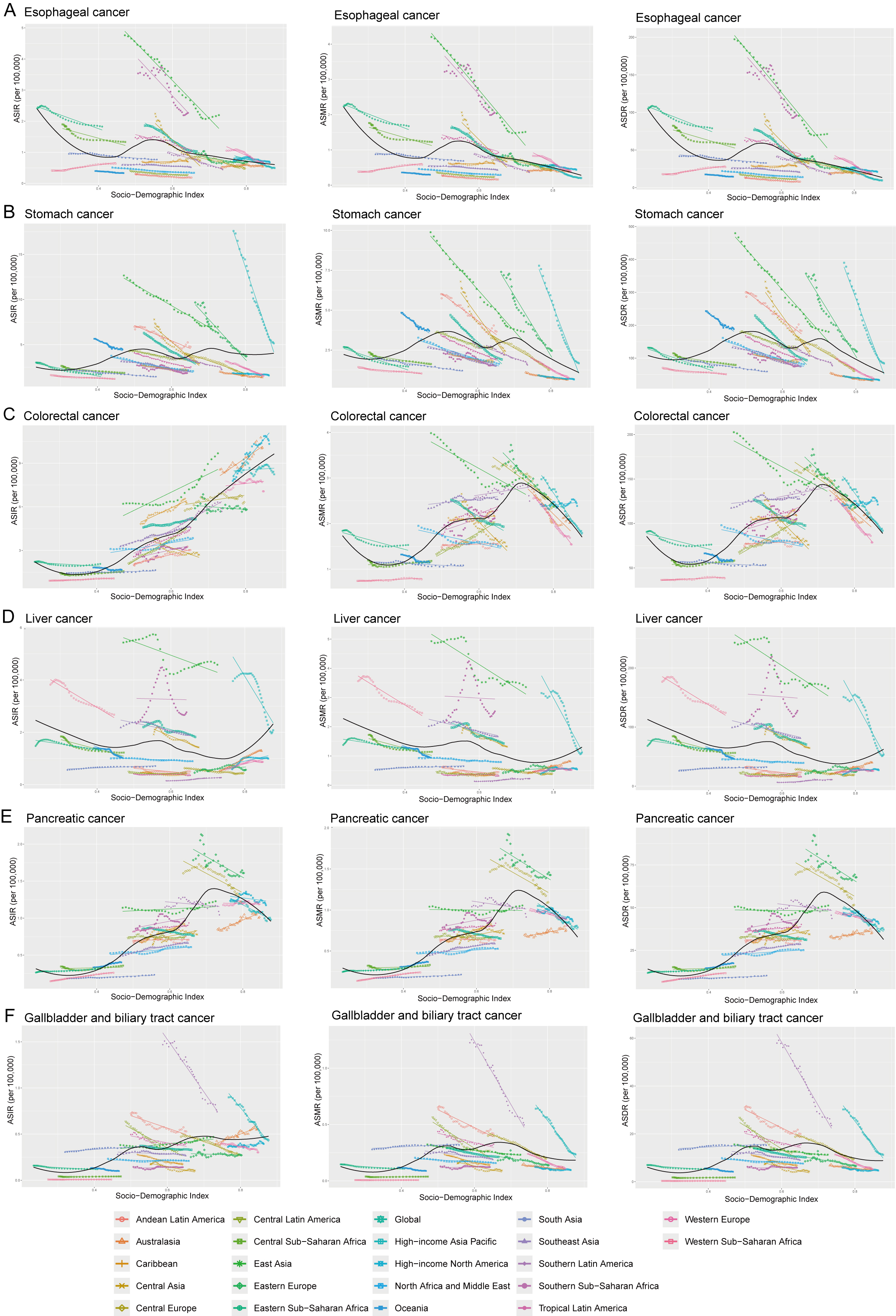


## **Figure S11.** Percentage contribution of risk factors to the mortality rates of early-onset gastrointestinal cancers globally, in 1990 and 2021.


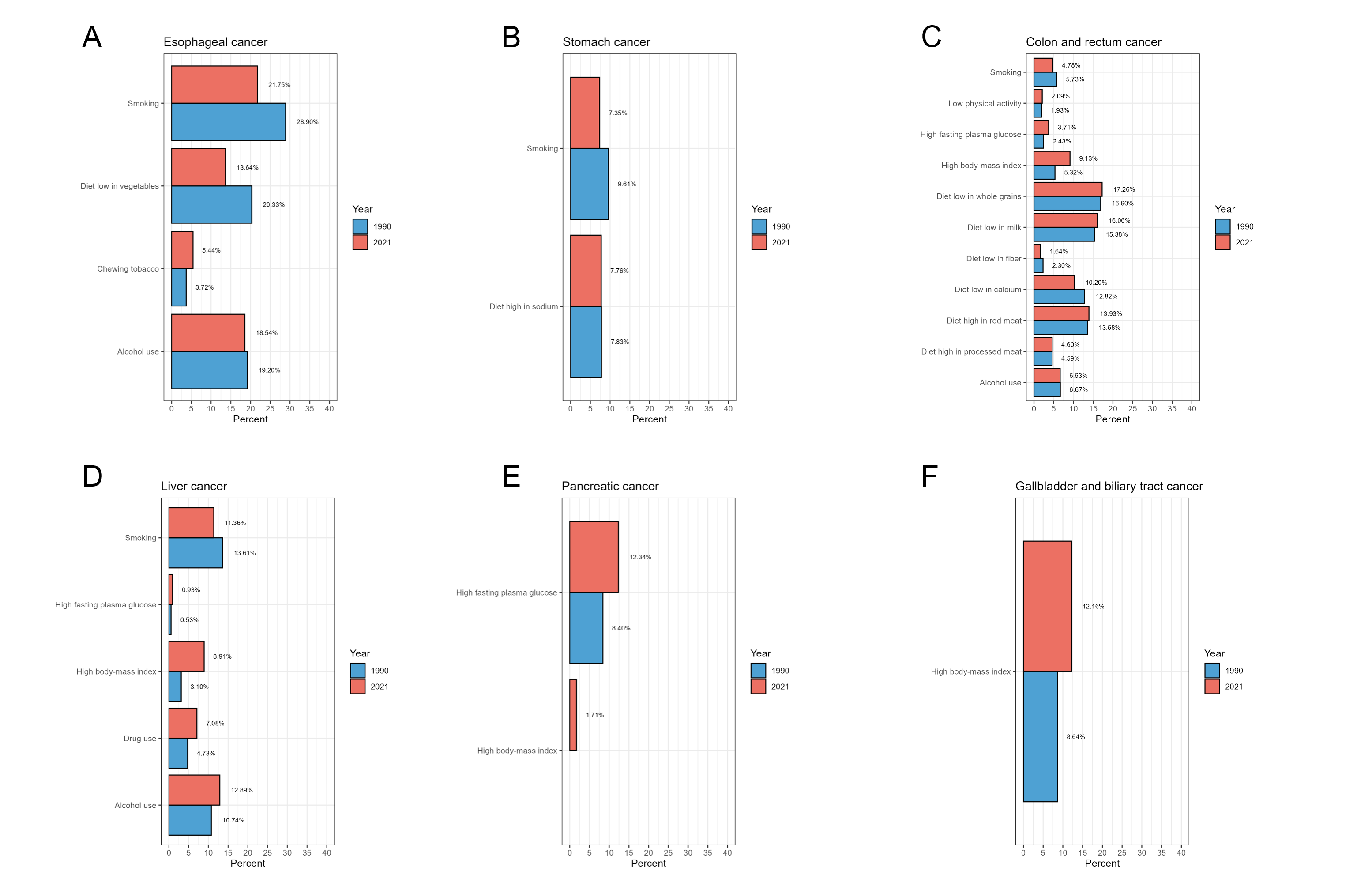


## **Figure S12.** Rankings and rates of risk factors to the ASMR of early-onset gastrointestinal cancers across five SDI regions, in 2021. ASMR, age-standardized mortality rate. SDI, socio-demographic index.


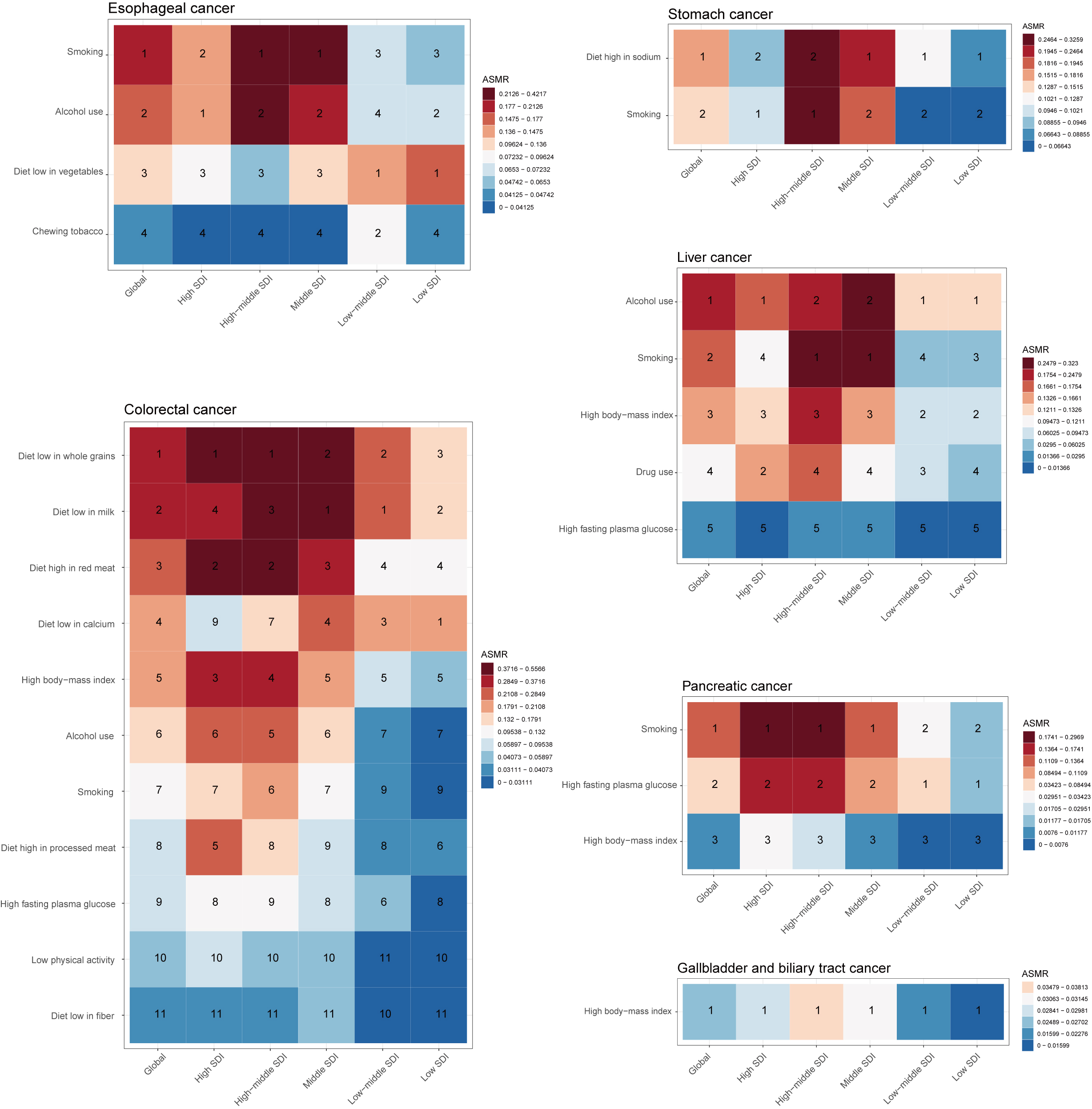


## **Table S1.** List of International Classification of Diseases (ICD) codes mapped to gastrointestinal cancers in GBD 2021

| **Cause** | **ICD10** | **ICD9** |
| --- | --- | --- |
| Esophageal cancer | C15-C15.9, D00.1, D13.0 | 150-150.9, 211.0, 230.1 |
| Stomach cancer | C16-C16.9, D00.2, D13.1, D37.1 | 151-151.9, 211.1, 230.2 |
| Colorectal cancer | C18-C21.9, D01.0-D01.3, D12-D12.9, D37.3-D37.5 | 153-154.9, 209.1, 209.5, 211.3-211.4, 230.3-230.6, 569.0 |
| Liver cancer | C22-C22.8, D13.4 | 155-155.1, 155.3-155.9, 211.5 |
| Pancreatic cancer | C25-C25.9, D13.6-D13.7 | 157-157.9, 211.6-211.7 |
| Gallbladder and biliary tract cancer | C23-C24.9, D13.5 | 156-156.9 |

## **Table S2.** The Socio-demographic index of 204 countries and territories from 1990 to 2005.

| **Location** | **1990** | **1991** | **1992** | **1993** | **1994** | **1995** | **1996** | **1997** | **1998** | **1999** | **2000** | **2001** | **2002** | **2003** | **2004** | **2005** |
| --- | --- | --- | --- | --- | --- | --- | --- | --- | --- | --- | --- | --- | --- | --- | --- | --- |
| Andean Latin America | 0.500011 | 0.501652 | 0.503656 | 0.506388 | 0.510306 | 0.514896 | 0.519376 | 0.524036 | 0.528444 | 0.532744 | 0.537561 | 0.542572 | 0.547911 | 0.553109 | 0.558235 | 0.563092 |
| Australasia | 0.731234 | 0.734915 | 0.739006 | 0.743426 | 0.747726 | 0.752015 | 0.756387 | 0.76099 | 0.7653 | 0.769521 | 0.773791 | 0.778074 | 0.78248 | 0.786285 | 0.789428 | 0.791722 |
| Caribbean | 0.518111 | 0.522789 | 0.526984 | 0.530507 | 0.533584 | 0.536651 | 0.539776 | 0.543303 | 0.547365 | 0.55206 | 0.557369 | 0.56311 | 0.569069 | 0.574889 | 0.580533 | 0.585758 |
| Central Asia | 0.553362 | 0.555148 | 0.55727 | 0.560083 | 0.5627 | 0.565586 | 0.568761 | 0.571766 | 0.574659 | 0.578121 | 0.582156 | 0.586646 | 0.591457 | 0.59653 | 0.601737 | 0.607294 |
| Central Europe | 0.637272 | 0.643083 | 0.648772 | 0.65447 | 0.661219 | 0.667955 | 0.674121 | 0.680037 | 0.685968 | 0.692056 | 0.69878 | 0.705657 | 0.712001 | 0.717824 | 0.723494 | 0.728674 |
| Central Latin America | 0.485787 | 0.489559 | 0.49411 | 0.499418 | 0.505061 | 0.509874 | 0.514693 | 0.520171 | 0.526132 | 0.531807 | 0.5373 | 0.54244 | 0.547134 | 0.551446 | 0.556011 | 0.56075 |
| Central sub-Saharan Africa | 0.302375 | 0.305221 | 0.307804 | 0.309082 | 0.309898 | 0.311059 | 0.312674 | 0.314502 | 0.316506 | 0.318569 | 0.320841 | 0.323749 | 0.327567 | 0.331935 | 0.33742 | 0.344099 |
| East Asia | 0.471179 | 0.479936 | 0.488431 | 0.496859 | 0.504886 | 0.51353 | 0.523758 | 0.533816 | 0.542653 | 0.551367 | 0.558816 | 0.564235 | 0.570798 | 0.578905 | 0.587683 | 0.597096 |
| Eastern Europe | 0.66425 | 0.671452 | 0.678753 | 0.683359 | 0.686012 | 0.689459 | 0.692791 | 0.695203 | 0.69694 | 0.698814 | 0.701011 | 0.703506 | 0.706727 | 0.712025 | 0.719215 | 0.726266 |
| Eastern sub-Saharan Africa | 0.233622 | 0.23634 | 0.238615 | 0.240817 | 0.242902 | 0.245424 | 0.248585 | 0.252145 | 0.255891 | 0.259957 | 0.264165 | 0.268772 | 0.273484 | 0.278434 | 0.283937 | 0.28997 |
| High-income Asia Pacific | 0.767804 | 0.773333 | 0.778834 | 0.783967 | 0.789028 | 0.794099 | 0.798863 | 0.803128 | 0.806831 | 0.810469 | 0.814089 | 0.817545 | 0.82111 | 0.824636 | 0.828074 | 0.831062 |
| High-income North America | 0.76566 | 0.76805 | 0.77155 | 0.774978 | 0.778207 | 0.781348 | 0.784105 | 0.786348 | 0.788569 | 0.791196 | 0.794997 | 0.799275 | 0.802961 | 0.805821 | 0.807991 | 0.808887 |
| North Africa and Middle East | 0.437421 | 0.445921 | 0.453971 | 0.462102 | 0.470394 | 0.478553 | 0.486463 | 0.49398 | 0.501445 | 0.508956 | 0.516824 | 0.524091 | 0.531149 | 0.538292 | 0.545678 | 0.553047 |
| Oceania | 0.391195 | 0.394419 | 0.397572 | 0.401113 | 0.404892 | 0.408507 | 0.412437 | 0.415893 | 0.419225 | 0.422521 | 0.42507 | 0.427004 | 0.428535 | 0.43006 | 0.431581 | 0.433152 |
| South Asia | 0.319797 | 0.325854 | 0.331941 | 0.338013 | 0.344323 | 0.350726 | 0.357287 | 0.363719 | 0.370288 | 0.376979 | 0.383388 | 0.389533 | 0.395194 | 0.400983 | 0.407181 | 0.413987 |
| Southeast Asia | 0.464104 | 0.47175 | 0.47949 | 0.487349 | 0.495348 | 0.503351 | 0.511233 | 0.518664 | 0.524439 | 0.529668 | 0.534567 | 0.53901 | 0.543479 | 0.548051 | 0.552707 | 0.557391 |
| Southern Latin America | 0.587308 | 0.592119 | 0.59808 | 0.603589 | 0.609392 | 0.614373 | 0.61926 | 0.624756 | 0.630349 | 0.635748 | 0.641237 | 0.64604 | 0.649826 | 0.652552 | 0.655719 | 0.660519 |
| Southern sub-Saharan Africa | 0.506947 | 0.512378 | 0.517569 | 0.522773 | 0.528063 | 0.53338 | 0.53878 | 0.544134 | 0.549142 | 0.553885 | 0.558491 | 0.562511 | 0.56605 | 0.569215 | 0.57239 | 0.576116 |
| Tropical Latin America | 0.499588 | 0.50429 | 0.508057 | 0.511622 | 0.51533 | 0.519291 | 0.523499 | 0.527841 | 0.532165 | 0.536659 | 0.541716 | 0.546932 | 0.552365 | 0.557691 | 0.563269 | 0.568897 |
| Western Europe | 0.7464 | 0.75167 | 0.757022 | 0.762057 | 0.766693 | 0.770622 | 0.77408 | 0.777578 | 0.780867 | 0.784019 | 0.7874 | 0.790904 | 0.794165 | 0.796962 | 0.799716 | 0.802414 |
| Western sub-Saharan Africa | 0.2737 | 0.277411 | 0.281025 | 0.284432 | 0.2877 | 0.290983 | 0.294618 | 0.298302 | 0.302026 | 0.30581 | 0.309781 | 0.314028 | 0.318881 | 0.324233 | 0.330228 | 0.33678 |
| Global | 0.525529 | 0.530272 | 0.534637 | 0.538598 | 0.542389 | 0.546328 | 0.550703 | 0.555139 | 0.559381 | 0.563738 | 0.568003 | 0.571778 | 0.575688 | 0.579907 | 0.584379 | 0.588979 |

# Table S3. The Socio-demographic index of 204 countries and territories from 2006 to 2021.

| **Location** | **2006** | **2007** | **2008** | **2009** | **2010** | **2011** | **2012** | **2013** | **2014** | **2015** | **2016** | **2017** | **2018** | **2019** | **2020** | **2021** |
| --- | --- | --- | --- | --- | --- | --- | --- | --- | --- | --- | --- | --- | --- | --- | --- | --- |
| Andean Latin America | 0.567987 | 0.572999 | 0.578564 | 0.583999 | 0.590201 | 0.596945 | 0.603839 | 0.610705 | 0.61706 | 0.622761 | 0.628161 | 0.633538 | 0.63886 | 0.643921 | 0.647807 | 0.651602 |
| Australasia | 0.792857 | 0.79395 | 0.796402 | 0.799932 | 0.804112 | 0.808161 | 0.812464 | 0.817349 | 0.821647 | 0.825611 | 0.829457 | 0.832924 | 0.836619 | 0.840582 | 0.843333 | 0.845514 |
| Caribbean | 0.590602 | 0.59471 | 0.598297 | 0.601655 | 0.60548 | 0.609525 | 0.613255 | 0.616877 | 0.620443 | 0.624075 | 0.627582 | 0.630788 | 0.633972 | 0.637321 | 0.639709 | 0.642003 |
| Central Asia | 0.613078 | 0.619105 | 0.62493 | 0.630103 | 0.635296 | 0.640314 | 0.644674 | 0.648888 | 0.653 | 0.656818 | 0.660312 | 0.663577 | 0.666666 | 0.669837 | 0.672487 | 0.675164 |
| Central Europe | 0.733476 | 0.738077 | 0.743148 | 0.748766 | 0.754919 | 0.760349 | 0.764803 | 0.768568 | 0.771755 | 0.774844 | 0.777817 | 0.781359 | 0.785476 | 0.789749 | 0.793111 | 0.796244 |
| Central Latin America | 0.565649 | 0.570707 | 0.575839 | 0.57994 | 0.584253 | 0.589308 | 0.594827 | 0.600731 | 0.606509 | 0.611815 | 0.617086 | 0.622442 | 0.627765 | 0.632847 | 0.636996 | 0.640685 |
| Central sub-Saharan Africa | 0.35146 | 0.359566 | 0.368488 | 0.376669 | 0.385381 | 0.394692 | 0.404234 | 0.413522 | 0.422579 | 0.430988 | 0.438746 | 0.446179 | 0.45325 | 0.46016 | 0.466456 | 0.472256 |
| East Asia | 0.607273 | 0.617524 | 0.626956 | 0.636271 | 0.647985 | 0.657502 | 0.66293 | 0.668682 | 0.674063 | 0.677307 | 0.681528 | 0.689748 | 0.698626 | 0.708328 | 0.717709 | 0.725705 |
| Eastern Europe | 0.732617 | 0.738787 | 0.74545 | 0.75107 | 0.756606 | 0.761025 | 0.765423 | 0.770338 | 0.775092 | 0.779663 | 0.784805 | 0.790015 | 0.794486 | 0.798138 | 0.800505 | 0.802851 |
| Eastern sub-Saharan Africa | 0.296455 | 0.303576 | 0.310918 | 0.318457 | 0.326296 | 0.334253 | 0.341628 | 0.349207 | 0.35701 | 0.364907 | 0.372759 | 0.3805 | 0.388355 | 0.396345 | 0.40348 | 0.409721 |
| High-income Asia Pacific | 0.833766 | 0.836546 | 0.83928 | 0.841771 | 0.844682 | 0.847722 | 0.850693 | 0.853671 | 0.856612 | 0.859603 | 0.862733 | 0.86587 | 0.869073 | 0.872219 | 0.874437 | 0.876767 |
| High-income North America | 0.809721 | 0.812486 | 0.817084 | 0.82247 | 0.827814 | 0.832061 | 0.835669 | 0.839059 | 0.842341 | 0.845877 | 0.849423 | 0.85296 | 0.856316 | 0.859749 | 0.861874 | 0.863465 |
| North Africa and Middle East | 0.560089 | 0.566516 | 0.572339 | 0.577062 | 0.581849 | 0.587593 | 0.594112 | 0.601127 | 0.608419 | 0.615777 | 0.623142 | 0.630561 | 0.637918 | 0.645095 | 0.651673 | 0.658225 |
| Oceania | 0.434584 | 0.436102 | 0.437392 | 0.438808 | 0.440752 | 0.442618 | 0.444524 | 0.446594 | 0.449364 | 0.452397 | 0.455454 | 0.458378 | 0.460818 | 0.463334 | 0.465535 | 0.467445 |
| South Asia | 0.421367 | 0.429262 | 0.437012 | 0.445197 | 0.454076 | 0.463339 | 0.473081 | 0.483434 | 0.494084 | 0.504891 | 0.515311 | 0.525128 | 0.534477 | 0.543246 | 0.550586 | 0.557865 |
| Southeast Asia | 0.562302 | 0.567654 | 0.573208 | 0.578497 | 0.584268 | 0.590415 | 0.59678 | 0.603145 | 0.609439 | 0.615689 | 0.62184 | 0.627978 | 0.634096 | 0.640107 | 0.64507 | 0.649777 |
| Southern Latin America | 0.664386 | 0.667393 | 0.670689 | 0.674152 | 0.678761 | 0.684134 | 0.688428 | 0.691716 | 0.69604 | 0.703287 | 0.710788 | 0.717548 | 0.724952 | 0.731134 | 0.733963 | 0.735985 |
| Southern sub-Saharan Africa | 0.580685 | 0.585673 | 0.590405 | 0.594585 | 0.598836 | 0.603347 | 0.608038 | 0.612844 | 0.617453 | 0.621838 | 0.625919 | 0.629762 | 0.633411 | 0.63694 | 0.639882 | 0.6422 |
| Tropical Latin America | 0.57472 | 0.580915 | 0.587354 | 0.593303 | 0.599646 | 0.605957 | 0.611787 | 0.617402 | 0.622646 | 0.6274 | 0.631533 | 0.635765 | 0.64018 | 0.644694 | 0.648603 | 0.652442 |
| Western Europe | 0.805067 | 0.807723 | 0.810484 | 0.81311 | 0.816142 | 0.819375 | 0.822634 | 0.825787 | 0.82861 | 0.831322 | 0.834216 | 0.837522 | 0.840834 | 0.844212 | 0.84655 | 0.848726 |
| Western sub-Saharan Africa | 0.343248 | 0.35004 | 0.356716 | 0.363492 | 0.370609 | 0.377609 | 0.384753 | 0.39189 | 0.399162 | 0.406238 | 0.412927 | 0.419615 | 0.426437 | 0.433384 | 0.439822 | 0.446023 |
| Global | 0.593757 | 0.598641 | 0.60337 | 0.607759 | 0.612952 | 0.617759 | 0.621845 | 0.626343 | 0.630982 | 0.635441 | 0.640154 | 0.645564 | 0.651048 | 0.656576 | 0.661344 | 0.665821 |

## **Table S4.** AAPC of ASIR of early-onset gastrointestinal cancers from 1990 to 2021.

| **Location** | **Esophageal cancer (95%CI)** | **Stomach cancer (95%CI)** | **Colorectal cancer (95%CI)** | **Liver cancer (95%CI)** | **Pancreatic cancer ( 95%CI)** | **Gallbladder and biliary tract cancer (95%CI)** |
| --- | --- | --- | --- | --- | --- | --- |
| **Global** | -1.90 (-2.09,-1.72) | -2.32 (-2.41,-2.22) | 0.37 (0.24,0.50) | -0.61 (-0.68,-0.54) | -0.35 (-0.49,-0.21) | -0.39 (-0.57,-0.22) |
| **SDI regions** |  |  |  |  |  |  |
| High SDI | -0.92 (-1.13,-0.70) | -3.01 (-3.17,-2.85) | 0.40 (0.20,0.61) | -0.63 (-0.71,-0.56) | -0.36 (-0.55,-0.17) | -0.88 (-1.16,-0.59) |
| High-middle SDI | -1.96 (-2.33,-1.60) | -2.17 (-2.47,-1.87) | 0.96 (0.66,1.26) | -0.63 (-0.76,-0.49) | -0.27 (-0.56,0.03) | -0.16 (-0.5,0.18) |
| Middle SDI | -2.82 (-2.95,-2.69) | -2.33 (-2.50,-2.17) | 0.89 (0.69,1.10) | -0.75 (-0.85,-0.66) | 0.13 (0.01,0.26) | -0.14 (-0.35,0.07) |
| Low-middle SDI | -0.57 (-0.69,-0.45) | -1.30 (-1.50,-1.10) | 0.73 (0.63,0.82) | -0.66 (-0.74,-0.59) | 1.16 (1.09,1.23) | 0.21 (0.02,0.39) |
| Low SDI | -0.73 (-0.81,-0.65) | -1.41 (-1.57,-1.25) | -0.08 (-0.23,0.06) | 0.05 (-0.02,0.11) | 0.53 (0.43,0.62) | -0.08 (-0.37,0.20) |
| **Regions** |  |  |  |  |  |  |
| Andean Latin America | -1.42 (-1.94,-0.90) | -1.23 (-1.89,-0.57) | 1.32 (0.66,1.97) | -0.96 (-1.23,-0.68) | 0.06 (-0.61,0.74) | -1.02 (-1.63,-0.41) |
| Australasia | 0.31 (-0.12,0.74) | -1.23 (-2.02,-0.44) | 0.52 (-0.15,1.20) | 2.97 (2.87,3.07) | 0.56 (-0.25,1.39) | 0.95 (-0.38,2.30) |
| Caribbean | -0.02 (-0.46,0.43) | -0.83 (-1.12,-0.55) | 0.82 (0.48,1.17) | -0.29 (-0.53,-0.04) | 0.92 (0.67,1.17) | -1.42 (-1.63,-1.21) |
| Central Asia | -3.51 (-4.01,-3.01) | -3.06 (-3.27,-2.84) | -1.10 (-1.60,-0.59) | -0.98 (-1.11,-0.85) | 0.66 (0.04,1.28) | -1.51 (-2.39,-0.63) |
| Central Europe | -1.52 (-1.84,-1.20) | -2.73 (-3.09,-2.37) | 0.23 (-0.30,0.75) | -1.20 (-1.30,-1.10) | -0.85 (-1.01,-0.69) | -1.75 (-1.99,-1.52) |
| Central Latin America | -1.28 (-1.69,-0.86) | -0.70 (-0.87,-0.53) | 2.56 (2.28,2.85) | -0.17 (-0.22,-0.12) | 0.11 (-0.46,0.69) | -2.25 (-2.72,-1.78) |
| Central Sub-Saharan Africa | -1.14 (-1.25,-1.03) | -1.09 (-1.25,-0.94) | 0.29 (0.12,0.47) | -1.31 (-1.39,-1.23) | 0.14 (0.00,0.27) | -0.04 (-0.19,0.10) |
| East Asia | -2.52 (-2.75,-2.29) | -1.91 (-2.16,-1.66) | 1.44 (1.17,1.72) | -0.45 (-0.55,-0.34) | 0.24 (0.09,0.38) | 0.68 (0.45,0.91) |
| Eastern Europe | -1.04 (-1.82,-0.26) | -3.14 (-3.69,-2.58) | 0.07 (-0.66,0.81) | 0.65 (0.33,0.97) | -0.18 (-0.54,0.19) | 0.10 (-1.18,1.39) |
| Eastern Sub-Saharan Africa | -0.89 (-0.99,-0.78) | -1.86 (-1.97,-1.75) | -0.22 (-0.34,-0.10) | -0.38 (-0.43,-0.32) | 0.59 (0.51,0.67) | -0.78 (-0.84,-0.71) |
| High-income Asia Pacific | -1.58 (-1.88,-1.27) | -3.91 (-4.20,-3.62) | -0.01 (-0.38,0.37) | -2.08 (-2.19,-1.96) | -0.64 (-1.06,-0.21) | -2.35 (-2.63,-2.08) |
| High-income North America | -0.16 (-0.35,0.04) | -0.43 (-0.76,-0.10) | 0.94 (0.59,1.28) | 1.69 (1.51,1.88) | -0.17 (-0.46,0.12) | 0.22 (-0.6,1.05) |
| North Africa and Middle East | -1.29 (-1.37,-1.21) | -1.90 (-2.00,-1.79) | 0.69 (0.58,0.81) | -0.36 (-0.4,-0.32) | 0.49 (0.31,0.67) | -0.31 (-0.40,-0.22) |
| Oceania | -0.62 (-0.66,-0.58) | -0.77 (-0.86,-0.69) | -0.28 (-0.43,-0.13) | -0.98 (-1.03,-0.93) | 0.63 (0.53,0.73) | -0.94 (-1.16,-0.71) |
| South Asia | -0.67 (-0.77,-0.56) | -1.51 (-1.93,-1.08) | 0.43 (0.16,0.69) | 0.75 (0.67,0.83) | 0.85 (0.62,1.07) | 0.63 (0.46,0.80) |
| Southeast Asia | -0.36 (-0.51,-0.2) | -1.40 (-1.51,-1.30) | 1.14 (1.00,1.28) | -0.47 (-0.51,-0.43) | 1.06 (0.91,1.21) | -0.14 (-0.33,0.04) |
| Southern Latin America | -2.45 (-2.83,-2.08) | -1.68 (-2.06,-1.29) | 1.06 (0.60,1.51) | 1.92 (1.83,2.00) | -0.33 (-0.82,0.16) | -2.13 (-2.82,-1.44) |
| Southern Sub-Saharan Africa | -1.51 (-1.85,-1.18) | -1.21 (-1.82,-0.60) | 0.50 (-0.21,1.22) | 0.50 (0.17,0.84) | 0.34 (-0.03,0.72) | -0.11 (-0.74,0.53) |
| Tropical Latin America | -1.08 (-1.49,-0.67) | -1.54 (-1.80,-1.28) | 1.77 (1.67,1.88) | -0.56 (-0.65,-0.47) | 0.31 (0.21,0.41) | -0.86 (-1.15,-0.56) |
| Western Europe | -1.66 (-1.97,-1.34) | -2.36 (-2.73,-1.99) | -0.03 (-0.42,0.36) | 1.34 (1.28,1.41) | -0.3 (-0.76,0.17) | -0.86 (-1.28,-0.45) |
| Western Sub-Saharan Africa | 1.38 (1.21,1.55) | -0.97 (-1.07,-0.86) | 0.47 (0.34,0.61) | -1.15 (-1.22,-1.09) | 1.87 (1.72,2.01) | -0.06 (-0.16,0.04) |

AAPC, average annual percentage changes. ASIR, age-standardized incidence rate. SDI, socio-demographic index. CI, confidence interval.

# Table S5. AAPC of ASMR of early-onset gastrointestinal cancers from 1990 to 2021.

| **Location** | **Esophageal cancer (95%CI)** | **Stomach cancer (95%CI)** | **Colorectal cancer (95%CI)** | **Liver cancer (95%CI)** | **Pancreatic cancer (95%CI)** | **Gallbladder and biliary tract cancer (95%CI)** |
| --- | --- | --- | --- | --- | --- | --- |
| **Global** | -2.32 (-2.55,-2.10) | -2.87 (-3.04,-2.71) | -0.86 (-1.00,-0.72) | -0.97 (-1.16,-0.79) | -0.44 (-0.59,-0.28) | -1.05 (-1.10,-1.00) |
| **SDI regions** |  |  |  |  |  |  |
| High SDI | -1.52 (-1.7,-1.34) | -3.55 (-3.70,-3.39) | -0.99 (-1.11,-0.88) | -1.50 (-1.99,-1.00) | -0.64 (-0.83,-0.44) | -2.19 (-2.35,-2.03) |
| High-middle SDI | -2.79 (-3.16,-2.42) | -3.26 (-3.56,-2.95) | -0.98 (-1.26,-0.70) | -1.21 (-1.53,-0.89) | -0.43 (-0.71,-0.14) | -1.56 (-1.77,-1.34) |
| Middle SDI | -3.24 (-3.46,-3.02) | -3.11 (-3.32,-2.91) | -0.68 (-0.84,-0.52) | -1.25 (-1.47,-1.02) | 0.01 (-0.11,0.14) | -0.92 (-1.08,-0.76) |
| Low-middle SDI | -0.63 (-0.75,-0.52) | -1.47 (-1.67,-1.26) | 0.08 (-0.07,0.23) | 0.02 (-0.08,0.12) | 1.11 (1.04,1.18) | 0.10 (-0.11,0.30) |
| Low SDI | -0.76 (-0.84,-0.68) | -1.49 (-1.65,-1.33) | -0.50 (-0.65,-0.35) | -0.68 (-0.84,-0.52) | 0.50 (0.41,0.60) | -0.17 (-0.45,0.11) |
| **Regions** |  |  |  |  |  |  |
| Andean Latin America | -1.61 (-2.03,-1.18) | -1.60 (-2.24,-0.95) | 0.08 (-0.59,0.75) | -0.97 (-1.90,-0.03) | -0.03 (-0.71,0.65) | -1.44 (-2.05,-0.83) |
| Australasia | -0.04 (-0.48,0.39) | -1.74 (-2.61,-0.87) | -1.21 (-1.69,-0.73) | 2.29 (1.74,2.84) | 0.44 (0.34,0.54) | -0.90 (-2.21,0.42) |
| Caribbean | -0.09 (-0.54,0.36) | -0.88 (-1.15,-0.60) | 0.13 (-0.11,0.37) | -0.27 (-0.97,0.44) | 0.87 (0.63,1.12) | -1.52 (-1.73,-1.31) |
| Central Asia | -3.55 (-4.05,-3.05) | -3.19 (-3.49,-2.88) | -1.65 (-2.13,-1.17) | -0.99 (-1.37,-0.61) | 0.64 (0.02,1.27) | -1.59 (-2.45,-0.72) |
| Central Europe | -1.65 (-1.97,-1.34) | -3.05 (-3.40,-2.70) | -1.06 (-1.52,-0.6) | -1.42 (-1.97,-0.87) | -0.91 (-1.07,-0.75) | -2.25 (-2.48,-2.03) |
| Central Latin America | -1.42 (-1.79,-1.06) | -0.94 (-1.14,-0.74) | 1.39 (1.07,1.72) | -0.51 (-0.78,-0.24) | 0.04 (-0.53,0.61) | -2.58 (-3.05,-2.11) |
| Central Sub-Saharan Africa | -1.17 (-1.28,-1.06) | -1.16 (-1.32,-1.00) | -0.05 (-0.20,0.10) | -1.33 (-1.57,-1.09) | 0.12 (-0.01,0.26) | -0.10 (-0.24,0.05) |
| East Asia | -3.28 (-3.73,-2.84) | -3.20 (-3.41,-2.98) | -0.92 (-1.14,-0.71) | -1.15 (-1.59,-0.70) | 0.04 (-0.10,0.18) | -1.08 (-1.24,-0.93) |
| Eastern Europe | -1.31 (-1.89,-0.74) | -3.65 (-3.92,-3.38) | -1.00 (-1.74,-0.26) | 0.68 (-0.38,1.74) | -0.25 (-0.61,0.12) | -0.98 (-2.21,0.26) |
| Eastern Sub-Saharan Africa | -0.92 (-1.03,-0.82) | -1.95 (-2.06,-1.84) | -0.60 (-0.73,-0.48) | -0.41 (-0.50,-0.32) | 0.55 (0.48,0.63) | -0.85 (-0.91,-0.79) |
| High-income Asia Pacific | -3.05 (-3.39,-2.71) | -4.85 (-5.09,-4.61) | -1.66 (-1.93,-1.38) | -3.30 (-3.76,-2.85) | -0.90 (-1.33,-0.46) | -3.24 (-3.50,-2.99) |
| High-income North America | -0.51 (-1.04,0.02) | -1.05 (-1.32,-0.78) | -0.04 (-0.33,0.25) | 1.04 (0.56,1.52) | -0.44 (-0.75,-0.14) | -0.95 (-1.73,-0.16) |
| North Africa and Middle East | -1.48 (-1.57,-1.4) | -2.20 (-2.38,-2.02) | -0.82 (-0.96,-0.68) | -0.49 (-0.75,-0.24) | 0.34 (0.17,0.51) | -0.83 (-0.91,-0.75) |
| Oceania | -0.66 (-0.7,-0.62) | -0.86 (-0.95,-0.78) | -0.45 (-0.64,-0.25) | -1.05 (-1.21,-0.89) | 0.61 (0.51,0.70) | -0.98 (-1.19,-0.77) |
| South Asia | -0.75 (-0.86,-0.65) | -1.66 (-2.08,-1.25) | -0.17 (-0.43,0.09) | 0.69 (0.57,0.82) | 0.79 (0.57,1.02) | 0.45 (0.38,0.53) |
| Southeast Asia | -0.64 (-0.82,-0.45) | -1.79 (-1.88,-1.70) | 0.30 (0.19,0.42) | -0.65 (-0.85,-0.46) | 0.98 (0.83,1.12) | -0.75 (-0.92,-0.57) |
| Southern Latin America | -2.66 (-3.09,-2.23) | -2.06 (-2.45,-1.66) | -0.07 (-0.54,0.40) | 1.80 (1.10,2.51) | -0.45 (-0.94,0.05) | -2.92 (-3.5,-2.33) |
| Southern Sub-Saharan Africa | -1.54 (-1.87,-1.22) | -1.34 (-1.79,-0.89) | 0.13 (-0.61,0.87) | 0.50 (0.09,0.92) | 0.33 (-0.04,0.70) | -0.16 (-0.77,0.44) |
| Tropical Latin America | -1.19 (-1.6,-0.78) | -1.79 (-2.05,-1.53) | 0.88 (0.77,0.98) | -0.61 (-0.92,-0.30) | 0.25 (0.15,0.35) | -1.08 (-1.62,-0.54) |
| Western Europe | -2.42 (-2.71,-2.12) | -3.01 (-3.06,-2.97) | -1.67 (-1.88,-1.46) | 0.47 (0.09,0.85) | -0.70 (-1.15,-0.25) | -2.56 (-2.76,-2.36) |
| Western Sub-Saharan Africa | 1.36 (1.20,1.53) | -1.04 (-1.14,-0.93) | 0.15 (0.03,0.27) | -1.17 (-1.41,-0.93) | 1.84 (1.69,1.98) | -0.12 (-0.23,-0.02) |

AAPC, average annual percentage changes. ASMR, age-standardized mortality rate. SDI, socio-demographic index. CI, confidence interval.

# Table S6. AAPC of ASDR of early-onset gastrointestinal cancers from 1990 to 2021.

| **Location** | **Esophageal cancer (95%CI)** | **Stomach cancer (95%CI)** | **Colorectal cancer (95%CI)** | **Liver cancer (95%CI)** | **Pancreatic cancer (95%CI)** | **Gallbladder and biliary tract cancer (95%CI)** |
| --- | --- | --- | --- | --- | --- | --- |
| **Global** | -2.31 (-2.53,-2.09) | -2.87 (-3.06,-2.68) | -0.84 (-0.98,-0.70) | -1.01 (-1.19,-0.82) | -0.44 (-0.60,-0.28) | -1.03 (-1.08,-0.98) |
| **SDI regions** |  |  |  |  |  |  |
| High SDI | -1.48 (-1.65,-1.30) | -3.51 (-3.64,-3.38) | -0.93 (-1.05,-0.81) | -1.41 (-1.71,-1.11) | -0.63 (-0.81,-0.44) | -2.14 (-2.28,-2.00) |
| High-middle SDI | -2.81 (-3.02,-2.60) | -3.22 (-3.52,-2.92) | -0.92 (-1.20,-0.64) | -1.25 (-1.56,-0.93) | -0.43 (-0.71,-0.14) | -1.52 (-1.73,-1.31) |
| Middle SDI | -3.24 (-3.44,-3.03) | -3.11 (-3.29,-2.93) | -0.68 (-0.83,-0.52) | -1.28 (-1.52,-1.03) | 0.01 (-0.13,0.14) | -0.93 (-1.1,-0.76) |
| Low-middle SDI | -0.64 (-0.76,-0.52) | -1.48 (-1.68,-1.28) | 0.08 (-0.08,0.24) | 0.03 (-0.07,0.13) | 1.1 (1.03,1.17) | 0.10 (-0.11,0.32) |
| Low SDI | -0.77 (-0.88,-0.66) | -1.49 (-1.66,-1.33) | -0.45 (-0.54,-0.37) | -0.65 (-0.81,-0.49) | 0.49 (0.4,0.59) | -0.15 (-0.44,0.14) |
| **Regions** |  |  |  |  |  |  |
| Andean Latin America | -1.60 (-2.04,-1.17) | -1.60 (-2.24,-0.95) | 0.09 (-0.55,0.75) | -0.91 (-1.74,-0.08) | -0.03 (-0.69,0.64) | -1.45 (-2.05,-0.84) |
| Australasia | -0.06 (-0.49,0.38) | -1.77 (-2.63,-0.90) | -1.08 (-1.57,-0.60) | 2.27 (1.75,2.80) | 0.40 (0.31,0.50) | -0.85 (-2.15,0.47) |
| Caribbean | -0.12 (-0.56,0.32) | -0.86 (-1.15,-0.57) | 0.10 (-0.16,0.36) | -0.27 (-0.99,0.45) | 0.84 (0.60,1.08) | -1.52 (-1.72,-1.31) |
| Central Asia | -3.52 (-4.02,-3.02) | -3.18 (-3.46,-2.90) | -1.67 (-2.11,-1.22) | -1.00 (-1.36,-0.64) | 0.63 (0.00,1.26) | -1.6 (-2.46,-0.73) |
| Central Europe | -1.66 (-1.98,-1.34) | -3.07 (-3.43,-2.72) | -1.04 (-1.59,-0.49) | -1.34 (-1.89,-0.77) | -0.93 (-1.09,-0.77) | -2.24 (-2.46,-2.01) |
| Central Latin America | -1.42 (-1.77,-1.06) | -0.95 (-1.14,-0.75) | 1.39 (1.07,1.72) | -0.46 (-0.73,-0.19) | 0.02 (-0.52,0.56) | -2.57 (-3.03,-2.11) |
| Central Sub-Saharan Africa | -1.17 (-1.27,-1.06) | -1.15 (-1.31,-0.99) | -0.04 (-0.19,0.11) | -1.31 (-1.53,-1.09) | 0.12 (-0.01,0.26) | -0.10 (-0.24,0.05) |
| East Asia | -3.27 (-3.71,-2.83) | -3.14 (-3.35,-2.93) | -0.86 (-1.08,-0.63) | -1.05 (-1.38,-0.72) | 0.04 (-0.14,0.22) | -1.05 (-1.33,-0.76) |
| Eastern Europe | -1.26 (-1.84,-0.67) | -3.63 (-4.30,-2.95) | -0.97 (-1.64,-0.31) | 0.64 (-0.38,1.67) | -0.27 (-0.64,0.09) | -0.97 (-2.21,0.28) |
| Eastern Sub-Saharan Africa | -0.91 (-1.00,-0.82) | -1.94 (-2.04,-1.83) | -0.56 (-0.69,-0.44) | -0.4 (-0.49,-0.31) | 0.56 (0.49,0.63) | -0.83 (-0.92,-0.74) |
| High-income Asia Pacific | -3.01 (-3.36,-2.67) | -4.85 (-5.08,-4.62) | -1.57 (-1.81,-1.34) | -3.31 (-3.77,-2.84) | -0.90 (-1.29,-0.52) | -3.21 (-3.46,-2.97) |
| High-income North America | -0.44 (-0.98,0.11) | -0.99 (-1.27,-0.72) | -0.01 (-0.39,0.38) | 1.07 (0.63,1.50) | -0.42 (-0.69,-0.14) | -0.88 (-1.58,-0.17) |
| North Africa and Middle East | -1.46 (-1.55,-1.38) | -2.18 (-2.33,-2.03) | -0.78 (-0.89,-0.67) | -0.45 (-0.69,-0.20) | 0.33 (0.17,0.50) | -0.83 (-0.94,-0.72) |
| Oceania | -0.65 (-0.69,-0.6) | -0.82 (-0.91,-0.73) | -0.42 (-0.63,-0.22) | -1.01 (-1.13,-0.89) | 0.61 (0.52,0.71) | -0.96 (-1.18,-0.75) |
| South Asia | -0.75 (-0.87,-0.64) | -1.67 (-2.11,-1.24) | -0.17 (-0.44,0.09) | 0.69 (0.56,0.81) | 0.79 (0.48,1.11) | 0.46 (0.32,0.62) |
| Southeast Asia | -0.67 (-0.84,-0.49) | -1.82 (-1.91,-1.72) | 0.30 (0.18,0.41) | -0.68 (-0.87,-0.48) | 0.97 (0.82,1.12) | -0.75 (-0.93,-0.57) |
| Southern Latin America | -2.62 (-3.03,-2.2) | -2.04 (-2.43,-1.65) | -0.02 (-0.46,0.43) | 1.83 (1.15,2.51) | -0.41 (-0.9,0.08) | -2.92 (-3.49,-2.34) |
| Southern Sub-Saharan Africa | -1.67 (-2.07,-1.26) | -1.26 (-1.88,-0.64) | 0.21 (-0.44,0.87) | 0.49 (-0.04,1.02) | 0.30 (-0.09,0.69) | -0.25 (-0.92,0.42) |
| Tropical Latin America | -1.18 (-1.58,-0.78) | -1.75 (-2.01,-1.49) | 0.86 (0.75,0.96) | -0.6 (-0.91,-0.29) | 0.36 (0.16,0.57) | -1.08 (-1.61,-0.55) |
| Western Europe | -2.36 (-2.66,-2.06) | -3.04 (-3.09,-2.99) | -1.58 (-1.79,-1.37) | 0.47 (0.09,0.84) | -0.71 (-1.16,-0.25) | -2.54 (-2.74,-2.33) |
| Western Sub-Saharan Africa | 1.38 (1.21,1.55) | -1.02 (-1.13,-0.91) | 0.17 (0.05,0.29) | -1.14 (-1.37,-0.9) | 1.85 (1.69,2.01) | -0.14 (-0.24,-0.03) |

AAPC, average annual percentage changes. ASDR, age-standardized disability-adjusted life years rate. SDI, socio-demographic index. CI, confidence interval.

## **Table S7.** ASIR of early-onset gastrointestinal cancers in both sexes in 204 countries and territories in 2021.

| **Location** | **Esophageal cancer (95%UI)** | **Stomach cancer (95%UI)** | **Colorectal cancer (95%UI)** | **Liver cancer (95%UI)** | **Pancreatic cancer (95%UI)** | **Gallbladder and biliary tract cancer (95%UI)** |
| --- | --- | --- | --- | --- | --- | --- |
| Afghanistan | 1.52 (0.64,2.64) | 9.85 (4.81,15.50) | 4.53 (1.64,8.01) | 1.53 (0.88,2.45) | 0.51 (0.23,0.97) | 0.33 (0.11,0.63) |
| Albania | 0.26 (0.18,0.39) | 1.94 (1.39,2.66) | 2.10 (1.48,3.00) | 1.23 (0.71,1.96) | 0.73 (0.50,1.01) | 0.12 (0.07,0.19) |
| Algeria | 0.1 (0.07,0.15) | 0.82 (0.57,1.12) | 1.69 (1.14,2.47) | 0.41 (0.25,0.65) | 0.24 (0.16,0.33) | 0.49 (0.32,0.76) |
| American Samoa | 0.33 (0.2,0.51) | 6.51 (4.40,9.16) | 5.18 (3.53,7.24) | 2.15 (1.31,3.36) | 1.00 (0.66,1.44) | 0.13 (0.08,0.20) |
| Andorra | 0.26 (0.14,0.43) | 1.61 (0.96,2.42) | 7.44 (4.26,11.38) | 1.94 (1.00,3.20) | 1.36 (0.79,2.06) | 0.36 (0.20,0.56) |
| Angola | 1.30 (0.77,2.00) | 1.84 (1.18,2.68) | 1.82 (1.14,2.75) | 2.04 (0.43,5.56) | 0.41 (0.22,0.70) | 0.05 (0.03,0.08) |
| Antigua and Barbuda | 0.33 (0.28,0.38) | 1.35 (1.17,1.57) | 4.38 (3.70,5.22) | 0.38 (0.32,0.45) | 0.57 (0.49,0.67) | 0.11 (0.10,0.13) |
| Argentina | 0.46 (0.38,0.57) | 1.84 (1.55,2.17) | 5.65 (4.60,6.76) | 0.22 (0.17,0.28) | 1.11 (0.94,1.3) | 0.55 (0.46,0.66) |
| Armenia | 0.10 (0.09,0.11) | 2.05 (1.8,2.32) | 3.47 (2.97,4.01) | 1.00 (0.81,1.21) | 1.54 (1.25,1.86) | 0.13 (0.11,0.16) |
| Australia | 0.56 (0.46,0.70) | 1.28 (1.07,1.52) | 10.14 (8.06,12.52) | 1.19 (0.91,1.54) | 1.02 (0.86,1.20) | 0.55 (0.47,0.62) |
| Austria | 0.38 (0.31,0.48) | 1.24 (1.03,1.49) | 5.21 (4.18,6.42) | 0.81 (0.62,1.04) | 1.02 (0.86,1.20) | 0.29 (0.23,0.36) |
| Azerbaijan | 0.66 (0.49,0.85) | 2.73 (2.06,3.77) | 2.56 (1.86,3.40) | 1.34 (0.70,2.40) | 0.72 (0.47,1.09) | 0.07 (0.04,0.11) |
| Bahamas | 1.33 (1.00,1.76) | 2.54 (1.94,3.31) | 9.82 (7.25,13.08) | 0.81 (0.62,1.06) | 0.98 (0.73,1.27) | 0.33 (0.25,0.42) |
| Bahrain | 0.21 (0.14,0.30) | 0.87 (0.60,1.21) | 3.50 (2.40,5.13) | 0.50 (0.29,0.81) | 0.63 (0.41,0.95) | 0.09 (0.05,0.15) |
| Bangladesh | 0.65 (0.39,1.04) | 1.22 (0.79,1.76) | 1.12 (0.71,1.85) | 0.48 (0.26,0.88) | 0.17 (0.10,0.31) | 0.28 (0.16,0.49) |
| Barbados | 0.86 (0.63,1.16) | 1.88 (1.40,2.48) | 8.67 (6.41,11.68) | 0.43 (0.32,0.58) | 1.02 (0.76,1.35) | 0.18 (0.14,0.24) |
| Belarus | 0.63 (0.46,0.85) | 4.47 (3.34,5.67) | 6.41 (4.61,8.55) | 0.6 (0.42,0.82) | 1.41 (1.05,1.82) | 0.17 (0.13,0.23) |
| Belgium | 0.72 (0.58,0.89) | 1.11 (0.93,1.33) | 6.18 (4.90,7.65) | 0.64 (0.50,0.82) | 1.13 (0.96,1.33) | 0.19 (0.16,0.24) |
| Belize | 0.46 (0.38,0.55) | 2.26 (1.90,2.69) | 3.81 (3.15,4.55) | 0.58 (0.48,0.68) | 0.96 (0.8,1.14) | 0.19 (0.16,0.22) |
| Benin | 0.76 (0.45,1.16) | 1.60 (1.02,2.28) | 0.96 (0.60,1.48) | 4.13 (2.19,7.07) | 0.36 (0.20,0.56) | 0.01 (0.00,0.01) |
| Bermuda | 0.75 (0.54,1.01) | 0.99 (0.75,1.27) | 13.35 (9.63,17.66) | 0.30 (0.23,0.37) | 1.17 (0.91,1.45) | 0.11 (0.09,0.15) |
| Bhutan | 0.67 (0.41,1.04) | 1.22 (0.75,1.90) | 1.28 (0.79,1.91) | 0.87 (0.44,1.50) | 0.21 (0.10,0.35) | 0.30 (0.18,0.50) |
| Bolivia (Plurinational State of) | 0.26 (0.16,0.39) | 5.16 (3.38,7.53) | 2.73 (1.71,4.13) | 0.52 (0.29,0.86) | 0.79 (0.51,1.18) | 0.64 (0.40,0.99) |
| Bosnia and Herzegovina | 0.35 (0.23,0.47) | 1.41 (0.95,1.85) | 4.39 (2.95,5.83) | 0.61 (0.38,0.86) | 1.01 (0.69,1.34) | 0.26 (0.16,0.45) |
| Botswana | 1.44 (0.87,2.19) | 1.06 (0.67,1.56) | 2.05 (1.23,3.47) | 1.90 (0.75,4.76) | 0.68 (0.38,1.21) | 0.09 (0.05,0.16) |
| Brazil | 1.06 (0.98,1.14) | 2.54 (2.39,2.70) | 4.56 (4.23,4.93) | 0.34 (0.31,0.36) | 0.93 (0.88,0.99) | 0.41 (0.38,0.43) |
| Brunei Darussalam | 0.30 (0.21,0.42) | 3.57 (2.60,4.70) | 6.78 (5.02,8.99) | 1.98 (1.21,3.14) | 0.77 (0.57,1.01) | 0.25 (0.16,0.35) |
| Bulgaria | 0.54 (0.44,0.66) | 2.35 (1.84,2.92) | 8.58 (6.63,10.86) | 0.74 (0.52,1.03) | 1.64 (1.29,2.04) | 0.21 (0.16,0.26) |
| Burkina Faso | 0.83 (0.49,1.29) | 1.98 (1.26,2.81) | 1.03 (0.66,1.53) | 6.37 (2.47,13.02) | 0.31 (0.19,0.48) | 0.01 (0.00,0.01) |
| Burundi | 1.76 (1.12,2.63) | 1.80 (1.13,2.62) | 1.61 (0.99,2.58) | 0.69 (0.35,1.38) | 0.26 (0.16,0.46) | 0.10 (0.05,0.17) |
| Cabo Verde | 2.65 (1.59,4.13) | 2.97 (1.88,5.15) | 1.46 (0.99,2.14) | 4.21 (2.29,7.30) | 1.12 (0.74,1.67) | 0.02 (0.01,0.04) |
| Cambodia | 0.58 (0.37,0.88) | 2.60 (1.69,3.82) | 4.23 (2.71,6.37) | 1.64 (0.72,3.44) | 0.61 (0.39,0.89) | 0.17 (0.10,0.30) |
| Cameroon | 1.09 (0.61,1.77) | 1.88 (1.07,2.84) | 1.44 (0.86,2.24) | 4.30 (2.00,8.55) | 0.58 (0.34,0.9) | 0.01 (0.00,0.01) |
| Canada | 0.71 (0.58,0.88) | 1.61 (1.32,1.95) | 10.71 (8.7,13.14) | 0.85 (0.66,1.09) | 0.92 (0.79,1.07) | 0.31 (0.25,0.39) |
| Central African Republic | 2.06 (1.15,3.30) | 3.22 (1.99,4.96) | 2.06 (1.13,3.34) | 1.96 (0.71,4.35) | 0.39 (0.23,0.61) | 0.05 (0.02,0.09) |
| Chad | 0.81 (0.49,1.26) | 2.29 (1.50,3.30) | 1.08 (0.69,1.60) | 4.21 (1.99,8.09) | 0.28 (0.18,0.41) | 0.01 (0.00,0.01) |
| Chile | 0.35 (0.29,0.43) | 3.13 (2.58,3.75) | 5.04 (4.17,6.08) | 0.35 (0.27,0.43) | 0.77 (0.65,0.90) | 1.23 (1.02,1.48) |
| China | 2.18 (1.69,2.76) | 7.00 (5.43,8.95) | 9.55 (7.58,11.79) | 4.60 (3.50,6.00) | 1.22 (0.95,1.54) | 0.47 (0.29,0.62) |
| Colombia | 0.27 (0.20,0.35) | 4.24 (3.31,5.28) | 5.39 (4.05,7.03) | 0.31 (0.23,0.40) | 0.67 (0.52,0.84) | 0.37 (0.29,0.47) |
| Comoros | 2.07 (1.33,3.12) | 1.65 (1.09,2.41) | 2.12 (1.33,3.28) | 1.16 (0.63,1.99) | 0.44 (0.27,0.70) | 0.11 (0.06,0.18) |
| Congo | 1.82 (1.07,3.00) | 2.01 (1.25,3.02) | 2.38 (1.49,3.64) | 1.88 (0.72,4.01) | 0.59 (0.33,0.97) | 0.06 (0.03,0.09) |
| Cook Islands | 0.48 (0.29,0.77) | 2.36 (1.54,3.51) | 2.39 (1.58,3.56) | 3.55 (1.99,5.82) | 0.69 (0.44,1.03) | 0.10 (0.05,0.18) |
| Costa Rica | 0.26 (0.20,0.32) | 4.71 (3.86,5.70) | 8.84 (6.92,11.16) | 0.83 (0.63,1.07) | 0.81 (0.66,0.98) | 0.32 (0.26,0.40) |
| Croatia | 0.47 (0.36,0.62) | 1.67 (1.33,2.06) | 7.43 (5.77,9.26) | 0.31 (0.22,0.44) | 0.99 (0.79,1.20) | 0.27 (0.21,0.35) |
| Cuba | 0.91 (0.69,1.18) | 1.08 (0.86,1.35) | 8.79 (6.62,11.31) | 0.32 (0.24,0.42) | 0.81 (0.64,1.02) | 0.13 (0.10,0.17) |
| Cyprus | 0.20 (0.13,0.29) | 0.88 (0.63,1.19) | 3.94 (2.85,5.43) | 0.37 (0.23,0.61) | 0.64 (0.45,0.92) | 0.18 (0.11,0.29) |
| Czechia | 0.49 (0.38,0.63) | 1.18 (0.91,1.5) | 6.74 (5.16,8.55) | 0.28 (0.20,0.39) | 1.29 (1.01,1.60) | 0.37 (0.29,0.47) |
| Cote d'lovire | 0.21 (0.13,0.32) | 0.71 (0.43,1.07) | 0.82 (0.50,1.30) | 1.09 (0.54,2.04) | 0.25 (0.15,0.41) | 0.01 (0.00,0.01) |
| Democratic People's Republic of Korea | 1.99 (1.13,3.24) | 7.54 (4.75,11.4) | 5.59 (3.19,9.47) | 3.41 (1.70,6.35) | 1.01 (0.57,1.60) | 0.35 (0.19,0.58) |
| Democratic Republic of the Congo | 1.24 (0.75,1.93) | 1.77 (1.15,2.57) | 1.30 (0.78,2.15) | 0.78 (0.32,1.91) | 0.30 (0.17,0.50) | 0.04 (0.02,0.06) |
| Denmark | 0.62 (0.50,0.76) | 0.95 (0.78,1.14) | 4.99 (3.99,6.17) | 0.3 (0.23,0.38) | 0.82 (0.68,0.97) | 0.16 (0.12,0.19) |
| Djibouti | 1.91 (1.13,3.06) | 1.58 (0.97,2.41) | 2.47 (1.46,4.00) | 1.09 (0.54,1.94) | 0.42 (0.22,0.73) | 0.10 (0.05,0.19) |
| Dominica | 0.82 (0.54,1.22) | 3.51 (2.44,4.89) | 4.36 (3.02,6.22) | 0.56 (0.33,0.90) | 1.22 (0.84,1.74) | 0.23 (0.15,0.34) |
| Dominican Republic | 0.51 (0.33,0.75) | 1.87 (1.31,2.58) | 4.01 (2.76,5.58) | 0.51 (0.30,0.80) | 1.12 (0.78,1.54) | 0.15 (0.10,0.22) |
| Ecuador | 0.17 (0.12,0.23) | 4.57 (3.4,6.05) | 3.08 (2.19,4.19) | 0.50 (0.35,0.68) | 0.63 (0.46,0.84) | 0.39 (0.28,0.51) |
| Egypt | 0.14 (0.10,0.21) | 1.46 (0.76,2.03) | 3.53 (2.47,4.88) | 2.43 (1.60,3.44) | 0.69 (0.49,0.94) | 0.16 (0.10,0.23) |
| El Salvador | 0.35 (0.26,0.48) | 5.21 (3.89,6.97) | 4.54 (3.31,6.15) | 0.35 (0.25,0.48) | 0.85 (0.63,1.12) | 0.44 (0.31,0.63) |
| Equatorial Guinea | 1.45 (0.81,2.4) | 1.36 (0.77,2.20) | 2.53 (1.41,4.11) | 1.31 (0.6,2.55) | 0.69 (0.36,1.16) | 0.05 (0.03,0.09) |
| Eritrea | 2.69 (1.55,4.38) | 2.71 (1.66,4.09) | 2.70 (1.69,4.27) | 0.97 (0.5,1.86) | 0.41 (0.21,0.66) | 0.14 (0.08,0.23) |
| Estonia | 0.35 (0.27,0.45) | 2.46 (1.95,3.01) | 5.71 (4.27,7.24) | 0.55 (0.42,0.71) | 1.32 (1.03,1.64) | 0.14 (0.11,0.17) |
| Eswatini | 4.08 (2.34,6.42) | 2.57 (1.47,4.02) | 4.2 (2.24,6.69) | 8.54 (2.29,22.86) | 1.42 (0.71,2.47) | 0.16 (0.08,0.26) |
| Ethiopia | 0.74 (0.55,1.03) | 1.74 (1.27,2.36) | 2.56 (2.00,3.36) | 0.55 (0.36,0.88) | 0.16 (0.10,0.26) | 0.16 (0.11,0.26) |
| Fiji | 0.44 (0.26,0.69) | 2.32 (1.04,3.53) | 2.46 (1.58,3.60) | 1.39 (0.75,2.36) | 0.54 (0.36,0.79) | 0.17 (0.10,0.27) |
| Finland | 0.58 (0.51,0.67) | 1.06 (0.87,1.27) | 4.38 (3.46,5.48) | 0.71 (0.54,0.93) | 1.04 (0.87,1.23) | 0.27 (0.22,0.33) |
| France | 0.82 (0.65,1.04) | 1.39 (1.15,1.68) | 8.11 (6.45,10.02) | 1.01 (0.76,1.30) | 1.52 (1.25,1.81) | 0.22 (0.18,0.28) |
| Gabon | 1.72 (0.99,2.78) | 1.62 (1.00,2.48) | 3.20 (1.97,4.92) | 2.45 (1.07,4.81) | 0.80 (0.49,1.24) | 0.05 (0.03,0.09) |
| Gambia | 0.31 (0.20,0.45) | 0.74 (0.47,1.10) | 0.63 (0.40,0.95) | 12.11 (5.92,22.51) | 0.30 (0.19,0.44) | 0.00 (0.00,0.00) |
| Georgia | 0.15 (0.13,0.18) | 2.35 (2.01,2.70) | 3.93 (3.32,4.60) | 0.78 (0.66,0.93) | 1.23 (1.04,1.46) | 0.15 (0.11,0.19) |
| Germany | 0.74 (0.60,0.91) | 1.98 (1.67,2.34) | 6.77 (5.41,8.35) | 0.65 (0.51,0.81) | 1.20 (1.01,1.39) | 0.36 (0.29,0.44) |
| Ghana | 0.54 (0.33,0.80) | 1.33 (0.88,1.91) | 1.29 (0.83,1.92) | 2.83 (1.41,5.22) | 0.55 (0.36,0.82) | 0.01 (0.00,0.01) |
| Greece | 0.36 (0.31,0.41) | 1.70 (1.51,1.91) | 5.04 (4.35,5.84) | 1.00 (0.87,1.14) | 1.21 (1.08,1.36) | 0.27 (0.23,0.31) |
| Greenland | 1.66 (1.08,2.39) | 2.53 (1.71,3.54) | 7.94 (5.36,10.91) | 0.92 (0.51,1.52) | 2.30 (1.57,3.19) | 0.18 (0.10,0.40) |
| Grenada | 0.82 (0.60,1.09) | 1.66 (1.30,2.09) | 5.85 (4.38,7.59) | 0.67 (0.48,0.89) | 1.47 (1.18,1.79) | 0.21 (0.16,0.26) |
| Guam | 0.63 (0.47,0.83) | 3.04 (2.34,3.83) | 6.78 (5.34,8.56) | 2.27 (1.65,3.07) | 1.19 (0.95,1.49) | 0.08 (0.06,0.11) |
| Guatemala | 0.29 (0.23,0.35) | 6.49 (5.35,7.74) | 2.78 (2.27,3.42) | 0.94 (0.78,1.12) | 0.55 (0.46,0.67) | 0.31 (0.26,0.38) |
| Guinea | 0.21 (0.13,0.32) | 2.24 (1.42,3.33) | 0.92 (0.57,1.39) | 6.48 (3.53,11.23) | 0.23 (0.14,0.37) | 0.01 (0.00,0.01) |
| Guinea-Bissau | 1.34 (0.82,2.07) | 3.4 (2.21,4.98) | 1.78 (1.11,2.68) | 7.26 (3.61,13.22) | 0.51 (0.33,0.78) | 0.01 (0.00,0.02) |
| Guyana | 0.5 (0.34,0.71) | 2.17 (1.53,2.98) | 5.02 (3.46,6.92) | 0.62 (0.45,0.84) | 1.08 (0.78,1.46) | 0.23 (0.16,0.31) |
| Haiti | 0.73 (0.44,1.12) | 3.96 (2.42,5.82) | 3.32 (1.97,5.25) | 0.28 (0.13,0.59) | 0.65 (0.40,0.99) | 0.24 (0.13,0.39) |
| Honduras | 0.15 (0.09,0.24) | 3.31 (2.04,5.10) | 1.56 (0.90,2.50) | 0.43 (0.23,0.72) | 0.65 (0.38,1.07) | 0.44 (0.25,0.71) |
| Hungary | 0.47 (0.37,0.60) | 1.37 (1.10,1.66) | 8.47 (6.65,10.59) | 0.25 (0.19,0.34) | 1.32 (1.05,1.61) | 0.32 (0.25,0.39) |
| Iceland | 0.98 (0.76,1.23) | 1.19 (0.97,1.43) | 6.17 (4.86,7.72) | 0.56 (0.42,0.74) | 1.26 (1.02,1.52) | 0.23 (0.18,0.29) |
| India | 0.72 (0.62,0.85) | 1.49 (1.27,1.86) | 1.68 (1.43,2.02) | 0.68 (0.57,0.83) | 0.24 (0.20,0.28) | 0.35 (0.24,0.43) |
| Indonesia | 0.30 (0.23,0.40) | 1.77 (1.38,2.35) | 3.62 (2.55,4.89) | 1.08 (0.68,1.72) | 0.69 (0.51,0.92) | 0.12 (0.08,0.20) |
| Iran (Islamic Republic of) | 0.55 (0.49,0.63) | 2.48 (1.94,2.86) | 3.92 (3.26,4.69) | 0.55 (0.47,0.64) | 0.53 (0.46,0.59) | 0.15 (0.06,0.18) |
| Iraq | 0.19 (0.13,0.29) | 1.27 (0.85,1.89) | 2.8 (1.85,4.25) | 0.85 (0.52,1.36) | 0.73 (0.48,1.07) | 0.16 (0.10,0.26) |
| Ireland | 0.91 (0.72,1.13) | 0.93 (0.76,1.13) | 6.39 (5.08,7.9) | 0.45 (0.34,0.57) | 0.86 (0.72,1.02) | 0.13 (0.11,0.17) |
| Israel | 0.23 (0.18,0.28) | 0.89 (0.75,1.05) | 5.15 (4.24,6.23) | 0.35 (0.27,0.45) | 0.88 (0.75,1.03) | 0.11 (0.09,0.13) |
| Italy | 0.29 (0.27,0.31) | 1.87 (1.68,2.08) | 6.69 (5.90,7.54) | 0.85 (0.74,0.97) | 1.01 (0.96,1.08) | 0.34 (0.30,0.39) |
| Jamaica | 0.50 (0.32,0.72) | 1.88 (1.33,2.68) | 6.21 (4.29,8.95) | 0.33 (0.22,0.48) | 0.65 (0.45,0.93) | 0.16 (0.11,0.23) |
| Japan | 0.58 (0.54,0.62) | 4.47 (4.14,4.74) | 8.85 (7.94,9.85) | 0.94 (0.84,1.03) | 1.06 (1.02,1.10) | 0.39 (0.36,0.43) |
| Jordan | 0.14 (0.09,0.20) | 0.87 (0.59,1.24) | 4.22 (2.81,6.36) | 0.25 (0.15,0.39) | 0.49 (0.33,0.73) | 0.19 (0.12,0.28) |
| Kazakhstan | 0.67 (0.59,0.76) | 2.65 (2.32,3.00) | 3.79 (3.27,4.34) | 0.9 (0.74,1.09) | 1.05 (0.86,1.27) | 0.18 (0.15,0.22) |
| Kenya | 1.74 (1.26,2.46) | 1.74 (1.31,2.29) | 1.64 (1.26,2.19) | 1.04 (0.74,1.48) | 0.44 (0.33,0.62) | 0.15 (0.10,0.22) |
| Kiribati | 1.36 (0.77,2.19) | 8.41 (5.31,12.52) | 2.63 (1.68,3.93) | 2.17 (1.22,3.58) | 0.23 (0.14,0.36) | 0.15 (0.05,0.26) |
| Kuwait | 0.12 (0.09,0.16) | 0.48 (0.36,0.63) | 4.93 (3.73,6.41) | 0.14 (0.10,0.19) | 0.56 (0.43,0.73) | 0.19 (0.15,0.25) |
| Kyrgyzstan | 0.39 (0.29,0.50) | 3.89 (3.08,4.78) | 3.01 (2.27,3.87) | 0.48 (0.32,0.67) | 1.09 (0.83,1.41) | 0.14 (0.10,0.18) |
| Lao People's Democratic Republic | 0.51 (0.31,0.79) | 2.15 (1.37,3.18) | 3.80 (2.36,5.69) | 1.91 (0.99,3.40) | 0.56 (0.36,0.83) | 0.14 (0.09,0.26) |
| Latvia | 0.45 (0.34,0.58) | 2.97 (2.38,3.62) | 5.20 (3.96,6.54) | 0.58 (0.43,0.77) | 1.79 (1.41,2.25) | 0.15 (0.11,0.18) |
| Lebanon | 0.13 (0.08,0.18) | 1.07 (0.78,1.44) | 4.20 (2.91,6.00) | 0.46 (0.29,0.71) | 0.54 (0.35,0.78) | 0.19 (0.13,0.29) |
| Lesotho | 3.51 (2.11,5.29) | 3.09 (1.85,4.67) | 3.09 (1.84,4.90) | 6.44 (1.92,19.17) | 1.01 (0.57,1.6) | 0.15 (0.08,0.23) |
| Liberia | 0.94 (0.55,1.5) | 1.85 (1.12,2.75) | 1.08 (0.59,1.85) | 5.94 (2.92,10.55) | 0.38 (0.18,0.65) | 0.01 (0.00,0.01) |
| Libya | 0.3 (0.19,0.46) | 1.69 (1.11,2.45) | 5.98 (3.90,8.78) | 1.81 (1.05,2.97) | 1.29 (0.81,1.98) | 0.58 (0.36,0.91) |
| Lithuania | 0.65 (0.5,0.84) | 3.28 (2.66,3.96) | 5.40 (4.14,6.87) | 0.69 (0.53,0.90) | 1.77 (1.43,2.15) | 0.25 (0.20,0.30) |
| Luxembourg | 0.52 (0.43,0.62) | 0.69 (0.59,0.81) | 4.97 (4.13,5.91) | 0.50 (0.42,0.59) | 0.78 (0.67,0.91) | 0.13 (0.11,0.15) |
| Madagascar | 1.92 (1.17,2.98) | 1.71 (1.09,2.49) | 1.84 (1.2,2.69) | 0.76 (0.41,1.33) | 0.28 (0.17,0.45) | 0.10 (0.05,0.16) |
| Malawi | 5.22 (3.49,7.85) | 0.80 (0.53,1.15) | 1.02 (0.65,1.66) | 1.41 (0.74,2.51) | 0.16 (0.10,0.25) | 0.03 (0.02,0.06) |
| Malaysia | 0.44 (0.31,0.63) | 1.58 (1.17,2.09) | 5.88 (4.29,7.85) | 1.43 (0.82,2.31) | 0.41 (0.30,0.55) | 0.16 (0.09,0.23) |
| Maldives | 0.22 (0.14,0.32) | 0.67 (0.46,0.97) | 1.42 (0.95,2.04) | 0.65 (0.33,1.16) | 0.26 (0.17,0.37) | 0.06 (0.04,0.12) |
| Mali | 0.37 (0.24,0.57) | 3.73 (2.45,5.28) | 1.36 (0.89,2.00) | 7.98 (4.49,13.28) | 0.35 (0.23,0.53) | 0.03 (0.02,0.05) |
| Malta | 0.49 (0.38,0.62) | 1.03 (0.85,1.24) | 5.54 (4.37,6.93) | 0.41 (0.31,0.54) | 1.24 (1.01,1.51) | 0.14 (0.11,0.18) |
| Marshall Islands | 0.58 (0.34,0.93) | 7.09 (4.68,10.12) | 3.80 (2.28,5.63) | 1.40 (0.69,2.72) | 0.79 (0.48,1.26) | 0.14 (0.07,0.25) |
| Mauritania | 0.68 (0.40,1.10) | 1.33 (0.87,1.94) | 1.23 (0.8,1.88) | 5.61 (2.20,10.97) | 0.41 (0.25,0.67) | 0.01 (0.00,0.01) |
| Mauritius | 0.94 (0.81,1.08) | 3.61 (3.13,4.10) | 6.52 (5.53,7.54) | 0.16 (0.14,0.18) | 1.15 (0.99,1.31) | 0.11 (0.1,0.13) |
| Mexico | 0.29 (0.25,0.33) | 2.74 (2.41,3.08) | 4.37 (3.83,4.94) | 0.43 (0.38,0.49) | 0.82 (0.72,0.92) | 0.33 (0.29,0.38) |
| Micronesia (Federated States of) | 0.64 (0.37,1.01) | 6.65 (4.28,9.72) | 3.87 (2.33,5.95) | 1.92 (0.96,3.62) | 0.88 (0.52,1.34) | 0.13 (0.07,0.22) |
| Monaco | 1.36 (0.82,2.02) | 2.08 (1.29,3.17) | 14.81 (9.05,22.49) | 1.67 (0.89,2.88) | 1.85 (0.91,3.13) | 0.15 (0.09,0.25) |
| Mongolia | 2.11 (1.50,2.86) | 8.48 (6.34,11.70) | 2.88 (2.14,3.77) | 12.74 (8.31,18.94) | 1.55 (1.09,2.13) | 0.43 (0.28,0.61) |
| Montenegro | 0.43 (0.32,0.56) | 1.34 (1.05,1.72) | 4.98 (3.78,6.52) | 0.97 (0.70,1.33) | 1.32 (1.00,1.70) | 0.13 (0.09,0.19) |
| Morocco | 0.12 (0.07,0.18) | 0.47 (0.31,0.74) | 3.20 (1.88,5.16) | 0.11 (0.06,0.19) | 0.21 (0.14,0.33) | 0.09 (0.05,0.15) |
| Mozambique | 1.07 (0.64,1.67) | 1.22 (0.77,1.86) | 0.48 (0.30,0.72) | 4.18 (1.68,9.94) | 0.08 (0.05,0.12) | 0.13 (0.06,0.23) |
| Myanmar | 0.46 (0.29,0.69) | 1.91 (1.26,2.77) | 3.56 (2.31,5.19) | 0.89 (0.40,1.93) | 0.54 (0.35,0.79) | 0.12 (0.07,0.21) |
| Namibia | 0.49 (0.29,0.80) | 0.69 (0.42,1.05) | 1.77 (1.06,2.69) | 0.88 (0.47,1.56) | 0.27 (0.16,0.42) | 0.07 (0.04,0.12) |
| Nauru | 0.84 (0.48,1.33) | 8.65 (5.63,12.32) | 5.72 (2.82,8.65) | 2.89 (1.38,5.20) | 1.22 (0.59,1.93) | 0.20 (0.09,0.35) |
| Nepal | 0.79 (0.49,1.22) | 1.48 (0.95,2.19) | 1.32 (0.87,1.95) | 0.69 (0.37,1.15) | 0.19 (0.10,0.31) | 0.33 (0.21,0.53) |
| Netherlands | 0.70 (0.57,0.85) | 0.62 (0.53,0.71) | 11.92 (9.7,14.51) | 0.34 (0.26,0.42) | 0.83 (0.70,0.96) | 0.29 (0.23,0.36) |
| New Zealand | 0.47 (0.39,0.56) | 1.80 (1.53,2.10) | 9.30 (7.75,11.06) | 1.65 (1.32,2.09) | 0.95 (0.83,1.10) | 0.58 (0.47,0.70) |
| Nicaragua | 0.13 (0.09,0.19) | 2.37 (1.76,3.21) | 2.44 (1.71,3.34) | 0.51 (0.31,0.77) | 0.46 (0.32,0.62) | 0.31 (0.21,0.49) |
| Niger | 0.54 (0.33,0.86) | 1.78 (1.12,2.64) | 0.79 (0.49,1.22) | 2.60 (1.22,5.24) | 0.20 (0.11,0.33) | 0.01 (0.00,0.01) |
| Nigeria | 0.62 (0.38,0.88) | 0.48 (0.34,0.66) | 0.99 (0.63,1.40) | 0.99 (0.59,1.59) | 0.12 (0.08,0.17) | 0.01 (0.00,0.01) |
| Niue | 0.43 (0.26,0.70) | 3.73 (2.48,5.55) | 3.81 (2.50,5.61) | 1.53 (0.76,2.89) | 0.87 (0.55,1.34) | 0.11 (0.06,0.17) |
| North Macedonia | 0.21 (0.15,0.29) | 1.88 (1.41,2.45) | 3.75 (2.75,5.03) | 0.94 (0.61,1.34) | 0.96 (0.71,1.33) | 0.16 (0.11,0.24) |
| Northern Mariana Islands | 0.42 (0.27,0.63) | 4.11 (2.94,5.62) | 4.98 (3.57,6.83) | 1.34 (0.76,2.20) | 1.15 (0.84,1.56) | 0.07 (0.04,0.13) |
| Norway | 0.35 (0.32,0.39) | 0.78 (0.70,0.87) | 6.36 (5.57,7.22) | 0.89 (0.78,1.01) | 0.87 (0.81,0.93) | 0.30 (0.25,0.35) |
| Oman | 0.17 (0.11,0.25) | 0.77 (0.52,1.10) | 0.98 (0.63,1.53) | 0.62 (0.35,1.05) | 0.20 (0.12,0.32) | 0.07 (0.04,0.12) |
| Pakistan | 1.23 (0.90,1.69) | 1.31 (0.94,1.78) | 1.75 (1.28,2.39) | 0.96 (0.65,1.39) | 0.23 (0.17,0.33) | 0.53 (0.36,0.80) |
| Palau | 0.70 (0.44,1.07) | 8.11 (5.4,11.65) | 3.42 (2.29,4.91) | 3.82 (1.88,6.88) | 1.16 (0.78,1.70) | 0.05 (0.03,0.08) |
| Palestine | 0.11 (0.07,0.15) | 1.00 (0.72,1.34) | 5.30 (3.80,7.25) | 0.76 (0.49,1.14) | 0.62 (0.43,0.85) | 0.15 (0.09,0.22) |
| Panama | 0.18 (0.14,0.23) | 2.54 (1.97,3.16) | 7.85 (5.98,10.00) | 0.49 (0.38,0.61) | 0.50 (0.39,0.62) | 0.19 (0.14,0.24) |
| Papua New Guinea | 0.28 (0.17,0.44) | 4.27 (2.80,6.33) | 1.20 (0.80,1.70) | 0.74 (0.26,2.02) | 0.32 (0.20,0.54) | 0.09 (0.05,0.15) |
| Paraguay | 0.67 (0.42,1.01) | 1.64 (1.11,2.36) | 2.97 (2.06,4.18) | 0.56 (0.33,0.87) | 0.68 (0.46,0.97) | 0.26 (0.16,0.38) |
| Peru | 0.18 (0.11,0.26) | 4.63 (3.12,6.43) | 3.01 (2.04,4.24) | 0.47 (0.27,0.73) | 0.71 (0.48,1.02) | 0.56 (0.36,0.86) |
| Philippines | 0.33 (0.27,0.40) | 1.36 (1.09,1.71) | 5.19 (4.30,6.14) | 2.07 (1.66,2.57) | 0.76 (0.62,0.90) | 0.12 (0.07,0.15) |
| Poland | 0.49 (0.44,0.54) | 1.52 (1.38,1.66) | 5.39 (4.76,6.06) | 0.30 (0.26,0.33) | 1.09 (0.99,1.20) | 0.29 (0.27,0.32) |
| Portugal | 0.68 (0.55,0.85) | 2.96 (2.45,3.52) | 9.84 (7.90,12.24) | 0.76 (0.59,0.99) | 0.91 (0.75,1.08) | 0.28 (0.22,0.35) |
| Puerto Rico | 0.58 (0.43,0.78) | 1.17 (0.90,1.48) | 13.04 (9.71,17.23) | 0.74 (0.53,1.00) | 1.24 (0.96,1.59) | 0.17 (0.13,0.22) |
| Qatar | 0.23 (0.14,0.35) | 0.73 (0.47,1.11) | 3.45 (2.25,5.09) | 1.10 (0.62,1.84) | 0.53 (0.33,0.81) | 0.14 (0.08,0.23) |
| Republic of Korea | 0.34 (0.24,0.47) | 6.98 (5.30,9.23) | 7.00 (5.35,9.11) | 4.70 (3.18,6.74) | 0.86 (0.65,1.10) | 0.58 (0.41,0.85) |
| Republic of Moldova | 0.26 (0.22,0.30) | 2.12 (1.82,2.47) | 4.94 (4.08,5.94) | 0.62 (0.53,0.72) | 1.51 (1.28,1.78) | 0.09 (0.08,0.11) |
| Romania | 0.54 (0.41,0.70) | 2.27 (1.80,2.80) | 6.71 (5.36,8.31) | 0.43 (0.31,0.56) | 1.50 (1.21,1.84) | 0.18 (0.14,0.22) |
| Russian Federation | 0.72 (0.65,0.78) | 3.82 (3.49,4.13) | 5.98 (5.43,6.51) | 0.60 (0.54,0.65) | 1.56 (1.41,1.69) | 0.31 (0.28,0.34) |
| Rwanda | 1.75 (1.09,2.65) | 1.53 (0.97,2.26) | 1.90 (1.15,2.95) | 1.00 (0.55,1.74) | 0.36 (0.22,0.59) | 0.12 (0.07,0.19) |
| Saint Kitts and Nevis | 0.53 (0.39,0.70) | 1.23 (0.89,1.67) | 4.30 (3.08,5.91) | 0.44 (0.32,0.58) | 0.81 (0.59,1.08) | 0.11 (0.08,0.15) |
| Saint Lucia | 0.92 (0.71,1.17) | 2.53 (2.00,3.18) | 4.83 (3.77,6.13) | 0.42 (0.33,0.52) | 1.17 (0.92,1.48) | 0.16 (0.13,0.20) |
| Saint Vincent and the Grenadines | 0.61 (0.50,0.74) | 2.61 (2.18,3.13) | 5.81 (4.64,7.18) | 0.74 (0.61,0.89) | 1.20 (0.99,1.44) | 0.18 (0.15,0.22) |
| Samoa | 0.19 (0.12,0.29) | 3.02 (1.94,4.5) | 2.63 (1.70,3.80) | 1.25 (0.65,2.21) | 0.65 (0.42,0.98) | 0.13 (0.07,0.20) |
| San Marino | 0.17 (0.08,0.30) | 2.06 (1.09,3.36) | 5.09 (2.61,8.24) | 0.42 (0.19,0.78) | 0.70 (0.36,1.16) | 0.25 (0.12,0.44) |
| Sao Tome and Principe | 0.66 (0.39,1.08) | 1.88 (1.19,2.89) | 1.64 (0.95,2.65) | 0.83 (0.37,1.79) | 0.14 (0.08,0.23) | 0.14 (0.08,0.24) |
| Saudi Arabia | 0.30 (0.19,0.46) | 0.92 (0.57,1.63) | 4.38 (2.80,6.47) | 0.70 (0.40,1.15) | 0.52 (0.34,0.75) | 0.29 (0.18,0.47) |
| Senegal | 0.75 (0.47,1.15) | 1.77 (1.17,2.53) | 1.21 (0.80,1.89) | 2.83 (1.58,4.71) | 0.40 (0.25,0.64) | 0.01 (0.00,0.01) |
| Serbia | 0.35 (0.24,0.52) | 1.42 (1.01,1.94) | 5.74 (4.14,7.73) | 0.67 (0.43,1.02) | 1.01 (0.71,1.36) | 0.17 (0.11,0.27) |
| Seychelles | 1.19 (0.81,1.73) | 1.94 (1.41,2.62) | 7.69 (5.57,10.27) | 1.13 (0.65,1.82) | 1.12 (0.78,1.56) | 0.13 (0.08,0.20) |
| Sierra Leone | 0.76 (0.46,1.20) | 1.87 (1.19,2.75) | 0.98 (0.60,1.55) | 3.05 (1.57,5.52) | 0.30 (0.18,0.48) | 0.01 (0.00,0.01) |
| Singapore | 0.26 (0.21,0.33) | 1.52 (1.24,1.82) | 5.67 (4.61,6.89) | 0.84 (0.64,1.08) | 0.46 (0.38,0.54) | 0.08 (0.06,0.09) |
| Slovakia | 0.64 (0.41,0.96) | 1.71 (1.23,2.34) | 7.53 (5.34,10.16) | 0.60 (0.36,1.00) | 1.27 (0.87,1.79) | 0.54 (0.35,0.82) |
| Slovenia | 0.40 (0.30,0.52) | 1.40 (1.08,1.78) | 5.80 (4.33,7.70) | 0.41 (0.29,0.56) | 0.83 (0.64,1.03) | 0.23 (0.17,0.30) |
| Solomon Islands | 0.60 (0.34,0.96) | 7.91 (5.08,11.61) | 3.11 (1.93,4.71) | 2.05 (1.00,3.81) | 0.64 (0.39,0.97) | 0.14 (0.07,0.24) |
| Somalia | 2.54 (1.51,4.04) | 2.95 (1.77,4.44) | 1.99 (1.09,3.37) | 2.19 (0.85,4.66) | 0.23 (0.10,0.42) | 0.10 (0.05,0.19) |
| South Africa | 2.07 (1.76,2.45) | 1.50 (1.28,1.90) | 3.21 (2.74,3.90) | 2.32 (1.88,2.86) | 0.91 (0.78,1.06) | 0.13 (0.09,0.17) |
| South Sudan | 2.51 (1.54,3.79) | 2.21 (1.42,3.33) | 2.30 (1.43,3.48) | 1.56 (0.82,2.72) | 0.38 (0.20,0.62) | 0.11 (0.06,0.18) |
| Spain | 0.49 (0.38,0.61) | 1.97 (1.64,2.36) | 6.55 (5.35,7.92) | 0.94 (0.72,1.23) | 0.95 (0.80,1.11) | 0.30 (0.23,0.37) |
| Sri Lanka | 0.82 (0.46,1.26) | 1.09 (0.64,1.65) | 1.95 (1.13,3.02) | 0.33 (0.17,0.57) | 0.26 (0.14,0.42) | 0.13 (0.06,0.22) |
| Sudan | 0.74 (0.36,1.22) | 3.49 (1.62,5.50) | 2.87 (1.56,4.65) | 0.73 (0.38,1.25) | 0.42 (0.22,0.72) | 0.17 (0.09,0.28) |
| Suriname | 0.35 (0.22,0.52) | 1.76 (1.21,2.45) | 5.31 (3.64,7.36) | 0.62 (0.33,1.06) | 1.18 (0.76,1.74) | 0.17 (0.11,0.25) |
| Sweden | 0.29 (0.24,0.35) | 0.73 (0.60,0.88) | 5.08 (4.21,6.09) | 0.48 (0.37,0.60) | 0.69 (0.57,0.82) | 0.27 (0.22,0.33) |
| Switzerland | 0.49 (0.38,0.61) | 0.94 (0.78,1.13) | 3.97 (3.25,4.86) | 0.45 (0.34,0.60) | 0.67 (0.55,0.81) | 0.17 (0.13,0.21) |
| Syrian Arab Republic | 0.14 (0.10,0.19) | 1.28 (0.85,1.82) | 3.35 (2.21,4.96) | 0.87 (0.55,1.29) | 0.55 (0.36,0.8) | 0.01 (0.01,0.02) |
| Taiwan (Province of China) | 3.55 (3.02,4.12) | 2.64 (2.15,3.17) | 14.12 (11.44,17.08) | 3.16 (2.42,4.01) | 1.38 (1.14,1.64) | 0.67 (0.54,0.83) |
| Tajikistan | 0.84 (0.57,1.22) | 3.51 (2.49,5.02) | 1.72 (1.12,2.81) | 0.84 (0.47,1.39) | 0.29 (0.19,0.42) | 0.01 (0.01,0.02) |
| Thailand | 1.69 (1.08,2.53) | 3.76 (1.94,5.51) | 9.32 (6.31,13.36) | 3.89 (2.33,6.22) | 1.19 (0.81,1.67) | 1.39 (0.68,2.09) |
| Timor-Leste | 0.38 (0.24,0.57) | 1.67 (1.05,2.43) | 2.38 (1.54,3.50) | 0.69 (0.34,1.31) | 0.35 (0.23,0.53) | 0.10 (0.06,0.17) |
| Togo | 0.97 (0.58,1.52) | 2.12 (1.29,3.14) | 1.18 (0.69,1.84) | 2.48 (1.27,4.71) | 0.39 (0.23,0.61) | 0.01 (0.00,0.01) |
| Tokelau | 0.35 (0.21,0.56) | 3.96 (2.71,5.75) | 3.48 (2.31,5.12) | 1.63 (0.79,3.06) | 0.70 (0.41,1.16) | 0.12 (0.07,0.20) |
| Tonga | 0.34 (0.20,0.54) | 5.31 (3.41,8.01) | 1.88 (1.23,2.82) | 7.39 (3.92,12.81) | 1.05 (0.64,1.63) | 0.08 (0.04,0.13) |
| Trinidad and Tobago | 0.43 (0.31,0.58) | 1.43 (1.05,1.90) | 8.32 (6.05,11.09) | 0.53 (0.39,0.68) | 1.01 (0.71,1.36) | 0.19 (0.14,0.26) |
| Tunisia | 0.09 (0.06,0.15) | 1.03 (0.66,1.51) | 3.33 (2.12,4.91) | 0.40 (0.22,0.68) | 0.40 (0.25,0.62) | 0.31 (0.19,0.49) |
| Turkey | 0.31 (0.20,0.43) | 2.67 (1.67,3.74) | 6.16 (4.37,8.38) | 0.46 (0.29,0.69) | 1.10 (0.77,1.52) | 0.20 (0.13,0.29) |
| Turkmenistan | 1.48 (1.11,1.94) | 2.82 (2.13,3.71) | 2.29 (1.72,3.02) | 1.24 (0.90,1.68) | 0.62 (0.46,0.84) | 0.11 (0.08,0.15) |
| Tuvalu | 0.48 (0.29,0.76) | 5.51 (3.73,7.86) | 3.33 (2.23,4.86) | 1.67 (0.90,3.00) | 0.72 (0.47,1.07) | 0.12 (0.07,0.19) |
| Uganda | 2.55 (1.57,3.85) | 1.51 (0.99,2.19) | 2.44 (1.54,3.67) | 2.07 (1.18,3.50) | 0.61 (0.37,0.94) | 0.10 (0.06,0.17) |
| Ukraine | 0.68 (0.42,1.01) | 3.35 (2.24,4.63) | 5.07 (3.39,7.06) | 0.39 (0.26,0.56) | 1.81 (1.20,2.51) | 0.18 (0.12,0.26) |
| United Arab Emirates | 0.16 (0.10,0.24) | 0.72 (0.47,1.07) | 2.49 (1.57,3.90) | 1.20 (0.69,1.94) | 0.76 (0.49,1.11) | 0.25 (0.15,0.40) |
| United Kingdom | 0.98 (0.94,1.01) | 1.06 (1.02,1.11) | 7.54 (7.22,7.89) | 1.31 (1.25,1.37) | 0.97 (0.94,1.00) | 0.40 (0.38,0.42) |
| United Republic of Tanzania | 1.84 (1.16,2.76) | 1.41 (0.93,1.99) | 2.00 (1.25,3.01) | 1.19 (0.65,2.00) | 0.38 (0.23,0.65) | 0.11 (0.06,0.18) |
| United States of America | 0.67 (0.64,0.70) | 1.57 (1.51,1.64) | 10.22 (9.73,10.71) | 0.98 (0.94,1.04) | 1.20 (1.16,1.25) | 0.40 (0.38,0.43) |
| United States Virgin Islands | 0.68 (0.42,1.06) | 1.89 (1.24,2.79) | 10.47 (6.76,16.12) | 0.53 (0.28,0.94) | 1.29 (0.82,1.95) | 0.26 (0.15,0.42) |
| Uruguay | 0.70 (0.57,0.87) | 2.49 (2.09,2.96) | 8.21 (6.58,10.07) | 0.38 (0.28,0.50) | 1.58 (1.31,1.87) | 0.63 (0.52,0.75) |
| Uzbekistan | 0.71 (0.54,0.93) | 2.29 (1.85,2.80) | 2.07 (1.60,2.63) | 0.97 (0.69,1.29) | 0.51 (0.39,0.66) | 0.05 (0.04,0.06) |
| Vanuatu | 0.49 (0.29,0.76) | 6.04 (3.98,8.65) | 3.05 (1.85,4.51) | 1.37 (0.69,2.52) | 0.56 (0.37,0.82) | 0.10 (0.05,0.17) |
| Venezuela (Bolivarian Republic of) | 0.28 (0.19,0.38) | 3.05 (2.26,4.04) | 4.49 (3.20,6.11) | 0.54 (0.40,0.70) | 0.70 (0.47,0.96) | 0.27 (0.18,0.37) |
| Viet Nam | 0.42 (0.25,0.68) | 2.33 (1.57,3.41) | 4.13 (2.68,6.19) | 3.49 (1.86,6.28) | 0.32 (0.20,0.48) | 0.16 (0.09,0.27) |
| Yemen | 0.63 (0.29,1.04) | 3.88 (1.65,6.17) | 2.20 (1.28,3.57) | 0.43 (0.20,0.84) | 0.29 (0.16,0.46) | 0.16 (0.08,0.26) |
| Zambia | 3.04 (1.70,5.38) | 1.93 (1.17,3.09) | 3.37 (1.74,8.03) | 1.18 (0.37,3.49) | 0.55 (0.33,0.85) | 0.13 (0.08,0.20) |
| Zimbabwe | 3.43 (2.10,5.22) | 3.63 (2.26,5.42) | 3.58 (2.28,5.41) | 4.26 (2.28,7.26) | 1.38 (0.89,2.10) | 0.20 (0.12,0.31) |

ASIR, age-standardized incidence rate. UI, uncertainty interval.

## **Table S8.** ASMR of early-onset gastrointestinal cancers in both sexes in 204 countries and territories in 2021.

| **Location** | **Esophageal cancer (95%UI)** | **Stomach cancer (95%UI)** | **Colorectal cancer (95%UI)** | **Liver cancer (95%UI)** | **Pancreatic cancer (95%UI)** | **Gallbladder and biliary tract cancer (95%UI)** |
| --- | --- | --- | --- | --- | --- | --- |
| Afghanistan | 1.40 (0.58,2.42) | 8.90 (4.30,13.90) | 2.98 (1.11,5.21) | 1.42 (0.81,2.28) | 0.46 (0.21,0.89) | 0.30 (0.10,0.57) |
| Albania | 0.23 (0.15,0.34) | 1.51 (1.09,2.07) | 0.83 (0.59,1.19) | 1.11 (0.63,1.78) | 0.65 (0.45,0.91) | 0.01 (0.06,0.16) |
| Algeria | 0.09 (0.06,0.12) | 0.65 (0.46,0.90) | 0.64 (0.44,0.91) | 0.36 (0.22,0.57) | 0.20 (0.14,0.29) | 0.38 (0.25,0.56) |
| American Samoa | 0.29 (0.17,0.44) | 4.97 (3.36,6.93) | 3.03 (2.09,4.27) | 1.92 (1.16,3.00) | 0.90 (0.59,1.29) | 0.10 (0.07,0.17) |
| Andorra | 0.17 (0.10,0.28) | 0.90 (0.53,1.35) | 1.55 (0.91,2.35) | 1.28 (0.68,2.12) | 1.10 (0.65,1.69) | 0.14 (0.08,0.21) |
| Angola | 1.19 (0.70,1.83) | 1.60 (1.03,2.32) | 1.36 (0.84,2.07) | 1.91 (0.40,5.31) | 0.38 (0.20,0.65) | 0.04 (0.03,0.07) |
| Antigua and Barbuda | 0.28 (0.24,0.33) | 1.03 (0.89,1.19) | 1.37 (1.19,1.57) | 0.33 (0.28,0.39) | 0.51 (0.44,0.60) | 0.09 (0.08,0.10) |
| Argentina | 0.39 (0.32,0.48) | 1.35 (1.14,1.57) | 2.66 (2.23,3.17) | 0.19 (0.15,0.24) | 0.98 (0.82,1.15) | 0.40 (0.34,0.48) |
| Armenia | 0.09 (0.08,0.10) | 1.65 (1.45,1.86) | 1.60 (1.39,1.82) | 0.92 (0.74,1.11) | 1.40 (1.12,1.68) | 0.11 (0.09,0.13) |
| Australia | 0.41 (0.33,0.50) | 0.60 (0.52,0.69) | 1.98 (1.63,2.35) | 0.78 (0.61,1.00) | 0.75 (0.64,0.88) | 0.10 (0.09,0.11) |
| Austria | 0.25 (0.20,0.32) | 0.61 (0.52,0.71) | 1.14 (0.95,1.36) | 0.47 (0.37,0.59) | 0.80 (0.67,0.93) | 0.12 (0.10,0.14) |
| Azerbaijan | 0.60 (0.45,0.77) | 2.28 (1.71,3.19) | 1.42 (1.04,1.90) | 1.24 (0.65,2.21) | 0.66 (0.43,1.00) | 0.06 (0.03,0.10) |
| Bahamas | 1.18 (0.89,1.57) | 2.00 (1.53,2.63) | 3.63 (2.72,4.82) | 0.73 (0.56,0.95) | 0.88 (0.66,1.14) | 0.26 (0.20,0.34) |
| Bahrain | 0.17 (0.11,0.25) | 0.60 (0.42,0.81) | 1.14 (0.77,1.73) | 0.42 (0.24,0.68) | 0.53 (0.35,0.8) | 0.06 (0.03,0.10) |
| Bangladesh | 0.58 (0.35,0.94) | 1.00 (0.65,1.45) | 0.70 (0.43,1.16) | 0.43 (0.23,0.80) | 0.15 (0.09,0.28) | 0.24 (0.14,0.43) |
| Barbados | 0.74 (0.54,0.99) | 1.41 (1.04,1.86) | 2.62 (1.95,3.47) | 0.38 (0.28,0.50) | 0.9 (0.68,1.20) | 0.14 (0.10,0.18) |
| Belarus | 0.51 (0.37,0.68) | 2.88 (2.18,3.65) | 2.09 (1.50,2.76) | 0.54 (0.37,0.76) | 1.22 (0.92,1.58) | 0.13 (0.10,0.17) |
| Belgium | 0.48 (0.38,0.59) | 0.66 (0.56,0.77) | 1.39 (1.14,1.67) | 0.44 (0.34,0.56) | 0.92 (0.78,1.08) | 0.08 (0.07,0.10) |
| Belize | 0.41 (0.34,0.49) | 1.82 (1.54,2.16) | 1.54 (1.30,1.82) | 0.52 (0.44,0.61) | 0.87 (0.72,1.03) | 0.16 (0.13,0.18) |
| Benin | 0.70 (0.42,1.07) | 1.38 (0.88,1.97) | 0.71 (0.44,1.10) | 3.82 (2.02,6.57) | 0.33 (0.19,0.53) | 0.01 (0.00,0.01) |
| Bermuda | 0.57 (0.41,0.77) | 0.62 (0.47,0.79) | 2.14 (1.53,2.82) | 0.23 (0.18,0.28) | 0.99 (0.78,1.23) | 0.06 (0.05,0.08) |
| Bhutan | 0.6 (0.37,0.94) | 1.01 (0.62,1.56) | 0.82 (0.52,1.23) | 0.79 (0.40,1.38) | 0.19 (0.09,0.31) | 0.25 (0.15,0.44) |
| Bolivia (Plurinational State of) | 0.23 (0.14,0.36) | 4.32 (2.83,6.31) | 1.79 (1.12,2.73) | 0.48 (0.27,0.79) | 0.72 (0.47,1.09) | 0.56 (0.35,0.86) |
| Bosnia and Herzegovina | 0.31 (0.20,0.42) | 1.11 (0.73,1.44) | 1.76 (1.17,2.32) | 0.55 (0.34,0.77) | 0.91 (0.63,1.22) | 0.21 (0.13,0.36) |
| Botswana | 1.31 (0.80,1.97) | 0.90 (0.57,1.32) | 1.42 (0.85,2.44) | 1.75 (0.68,4.47) | 0.62 (0.35,1.10) | 0.08 (0.04,0.14) |
| Brazil | 0.93 (0.86,1.00) | 1.99 (1.88,2.11) | 2.38 (2.21,2.55) | 0.30 (0.28,0.32) | 0.84 (0.79,0.89) | 0.33 (0.31,0.35) |
| Brunei Darussalam | 0.23 (0.16,0.32) | 2.07 (1.51,2.70) | 3.38 (2.53,4.45) | 1.65 (1.01,2.64) | 0.68 (0.51,0.90) | 0.21 (0.14,0.29) |
| Bulgaria | 0.47 (0.38,0.57) | 1.86 (1.48,2.30) | 3.42 (2.69,4.23) | 0.68 (0.48,0.94) | 1.47 (1.16,1.81) | 0.13 (0.10,0.16) |
| Burkina Faso | 0.76 (0.45,1.19) | 1.73 (1.11,2.49) | 0.78 (0.5,1.15) | 5.91 (2.26,12.07) | 0.29 (0.17,0.45) | 0.01 (0.00,0.01) |
| Burundi | 1.62 (1.04,2.47) | 1.58 (1.01,2.34) | 1.26 (0.78,2.04) | 0.64 (0.33,1.29) | 0.25 (0.14,0.43) | 0.09 (0.05,0.16) |
| Cabo Verde | 2.37 (1.46,3.68) | 2.38 (1.50,4.11) | 0.83 (0.56,1.20) | 3.79 (2.04,6.54) | 1.01 (0.66,1.53) | 0.02 (0.01,0.03) |
| Cambodia | 0.52 (0.33,0.78) | 2.10 (1.37,3.09) | 2.76 (1.79,4.12) | 1.50 (0.65,3.19) | 0.55 (0.35,0.81) | 0.15 (0.09,0.26) |
| Cameroon | 1.01 (0.56,1.66) | 1.61 (0.94,2.45) | 1.04 (0.62,1.62) | 3.96 (1.84,7.88) | 0.53 (0.31,0.84) | 0.01 (0.00,0.01) |
| Canada | 0.43 (0.35,0.53) | 0.63 (0.54,0.73) | 2.12 (1.78,2.53) | 0.51 (0.41,0.64) | 0.71 (0.61,0.82) | 0.10 (0.08,0.12) |
| Central African Republic | 1.91 (1.08,3.04) | 2.89 (1.79,4.36) | 1.72 (0.95,2.78) | 1.83 (0.66,4.04) | 0.36 (0.21,0.56) | 0.04 (0.02,0.08) |
| Chad | 0.75 (0.46,1.17) | 2.02 (1.32,2.90) | 0.85 (0.55,1.26) | 3.91 (1.87,7.49) | 0.26 (0.17,0.38) | 0.01 (0.00,0.01) |
| Chile | 0.27 (0.22,0.33) | 2.05 (1.70,2.43) | 1.79 (1.50,2.13) | 0.28 (0.22,0.35) | 0.66 (0.55,0.77) | 0.74 (0.62,0.86) |
| China | 1.49 (1.15,1.92) | 3.64 (2.82,4.66) | 2.96 (2.34,3.69) | 3.36 (2.56,4.39) | 1.05 (0.81,1.32) | 0.23 (0.15,0.30) |
| Colombia | 0.23 (0.17,0.29) | 3.11 (2.45,3.87) | 2.11 (1.62,2.72) | 0.27 (0.20,0.34) | 0.59 (0.46,0.74) | 0.28 (0.22,0.35) |
| Comoros | 1.90 (1.23,2.86) | 1.42 (0.95,2.08) | 1.56 (0.98,2.43) | 1.08 (0.58,1.86) | 0.40 (0.24,0.65) | 0.10 (0.06,0.17) |
| Congo | 1.67 (0.98,2.74) | 1.73 (1.06,2.62) | 1.72 (1.09,2.69) | 1.74 (0.67,3.72) | 0.54 (0.30,0.88) | 0.05 (0.03,0.08) |
| Cook Islands | 0.37 (0.22,0.60) | 1.51 (0.99,2.21) | 0.94 (0.63,1.40) | 2.96 (1.65,4.88) | 0.59 (0.39,0.87) | 0.07 (0.03,0.12) |
| Costa Rica | 0.21 (0.16,0.27) | 3.36 (2.76,4.03) | 3.06 (2.43,3.78) | 0.71 (0.54,0.91) | 0.71 (0.58,0.86) | 0.23 (0.18,0.28) |
| Croatia | 0.40 (0.30,0.52) | 0.96 (0.77,1.17) | 2.18 (1.73,2.71) | 0.24 (0.17,0.34) | 0.76 (0.61,0.93) | 0.13 (0.11,0.17) |
| Cuba | 0.76 (0.57,1.00) | 0.77 (0.61,0.95) | 2.11 (1.63,2.66) | 0.27 (0.21,0.36) | 0.71 (0.56,0.91) | 0.09 (0.07,0.11) |
| Cyprus | 0.13 (0.09,0.20) | 0.52 (0.38,0.69) | 0.84 (0.61,1.16) | 0.26 (0.16,0.41) | 0.53 (0.36,0.76) | 0.07 (0.05,0.12) |
| Czechia | 0.39 (0.30,0.50) | 0.68 (0.54,0.85) | 2.04 (1.60,2.57) | 0.25 (0.18,0.34) | 1.11 (0.86,1.38) | 0.22 (0.17,0.27) |
| C么te d'Ivoire | 0.19 (0.12,0.30) | 0.61 (0.36,0.93) | 0.59 (0.36,0.94) | 1.00 (0.50,1.90) | 0.23 (0.14,0.37) | 0.01 (0.00,0.01) |
| Democratic People's Republic of Korea | 1.66 (0.94,2.71) | 5.57 (3.54,8.35) | 2.94 (1.69,5.07) | 3.02 (1.49,5.64) | 0.89 (0.51,1.43) | 0.27 (0.15,0.44) |
| Democratic Republic of the Congo | 1.15 (0.68,1.77) | 1.55 (1.01,2.27) | 0.99 (0.58,1.68) | 0.73 (0.30,1.82) | 0.28 (0.15,0.47) | 0.03 (0.02,0.06) |
| Denmark | 0.43 (0.34,0.53) | 0.49 (0.41,0.59) | 1.24 (1.02,1.50) | 0.18 (0.14,0.22) | 0.65 (0.54,0.77) | 0.05 (0.04,0.06) |
| Djibouti | 1.74 (1.03,2.81) | 1.36 (0.82,2.07) | 1.79 (1.05,2.88) | 1.00 (0.50,1.80) | 0.38 (0.2,0.67) | 0.09 (0.05,0.17) |
| Dominica | 0.74 (0.49,1.11) | 2.87 (2.00,4.02) | 1.90 (1.33,2.68) | 0.51 (0.3,0.82) | 1.11 (0.76,1.58) | 0.20 (0.13,0.29) |
| Dominican Republic | 0.46 (0.30,0.66) | 1.52 (1.07,2.11) | 1.73 (1.20,2.38) | 0.46 (0.28,0.73) | 1.01 (0.71,1.4) | 0.13 (0.09,0.19) |
| Ecuador | 0.15 (0.11,0.20) | 3.57 (2.66,4.76) | 1.63 (1.17,2.20) | 0.45 (0.31,0.61) | 0.56 (0.42,0.75) | 0.31 (0.23,0.41) |
| Egypt | 0.13 (0.09,0.18) | 1.18 (0.61,1.64) | 1.61 (1.13,2.20) | 2.18 (1.43,3.10) | 0.62 (0.43,0.84) | 0.13 (0.08,0.19) |
| El Salvador | 0.31 (0.22,0.41) | 4.03 (3.01,5.39) | 2.03 (1.50,2.71) | 0.31 (0.22,0.42) | 0.76 (0.56,1.01) | 0.35 (0.24,0.49) |
| Equatorial Guinea | 1.31 (0.71,2.20) | 1.13 (0.64,1.84) | 1.64 (0.90,2.67) | 1.20 (0.55,2.34) | 0.63 (0.32,1.06) | 0.04 (0.02,0.08) |
| Eritrea | 2.48 (1.43,4.04) | 2.39 (1.46,3.59) | 2.11 (1.33,3.35) | 0.91 (0.46,1.72) | 0.38 (0.20,0.60) | 0.13 (0.07,0.21) |
| Estonia | 0.28 (0.21,0.36) | 1.27 (1.04,1.51) | 1.55 (1.18,1.91) | 0.50 (0.38,0.64) | 1.02 (0.79,1.28) | 0.10 (0.08,0.13) |
| Eswatini | 3.75 (2.07,5.96) | 2.22 (1.28,3.49) | 3.06 (1.63,4.83) | 7.88 (2.09,21.06) | 1.30 (0.65,2.26) | 0.14 (0.07,0.23) |
| Ethiopia | 0.68 (0.51,0.93) | 1.50 (1.10,2.03) | 1.88 (1.46,2.45) | 0.51 (0.33,0.82) | 0.15 (0.09,0.24) | 0.15 (0.10,0.24) |
| Fiji | 0.39 (0.23,0.62) | 1.87 (0.83,2.83) | 1.60 (1.02,2.33) | 1.26 (0.67,2.13) | 0.49 (0.32,0.70) | 0.15 (0.08,0.23) |
| Finland | 0.32 (0.28,0.36) | 0.51 (0.43,0.59) | 0.98 (0.80,1.19) | 0.37 (0.28,0.47) | 0.78 (0.66,0.92) | 0.12 (0.10,0.14) |
| France | 0.42 (0.33,0.52) | 0.79 (0.66,0.93) | 1.55 (1.26,1.88) | 0.63 (0.49,0.81) | 0.94 (0.78,1.12) | 0.09 (0.07,0.11) |
| Gabon | 1.56 (0.88,2.51) | 1.37 (0.85,2.09) | 2.16 (1.32,3.29) | 2.24 (0.98,4.38) | 0.73 (0.44,1.14) | 0.05 (0.03,0.08) |
| Gambia | 0.28 (0.18,0.42) | 0.64 (0.41,0.95) | 0.45 (0.29,0.68) | 11.15 (5.48,20.73) | 0.27 (0.17,0.42) | 0.00 (0.00,0.00) |
| Georgia | 0.14 (0.12,0.16) | 1.94 (1.68,2.23) | 2.06 (1.77,2.39) | 0.72 (0.60,0.86) | 1.13 (0.95,1.32) | 0.13 (0.10,0.16) |
| Germany | 0.43 (0.35,0.53) | 0.93 (0.80,1.07) | 1.61 (1.31,1.94) | 0.41 (0.32,0.50) | 0.87 (0.74,1.01) | 0.15 (0.12,0.18) |
| Ghana | 0.49 (0.30,0.74) | 1.13 (0.75,1.64) | 0.90 (0.58,1.36) | 2.60 (1.28,4.77) | 0.51 (0.33,0.76) | 0.01 (0.00,0.01) |
| Greece | 0.25 (0.22,0.29) | 1.04 (0.94,1.14) | 1.25 (1.13,1.39) | 0.71 (0.63,0.80) | 1.00 (0.89,1.12) | 0.12 (0.11,0.13) |
| Greenland | 1.39 (0.90,2.01) | 1.82 (1.25,2.56) | 3.67 (2.50,5.00) | 0.79 (0.44,1.31) | 2.02 (1.39,2.83) | 0.13 (0.07,0.28) |
| Grenada | 0.73 (0.54,0.98) | 1.33 (1.04,1.67) | 2.28 (1.74,2.91) | 0.60 (0.43,0.80) | 1.32 (1.06,1.62) | 0.17 (0.13,0.22) |
| Guam | 0.52 (0.38,0.68) | 2.12 (1.62,2.64) | 3.20 (2.53,4.04) | 1.97 (1.43,2.68) | 1.04 (0.83,1.30) | 0.06 (0.04,0.08) |
| Guatemala | 0.26 (0.21,0.31) | 5.35 (4.42,6.37) | 1.61 (1.33,1.95) | 0.86 (0.71,1.02) | 0.50 (0.41,0.61) | 0.27 (0.22,0.33) |
| Guinea | 0.19 (0.12,0.30) | 1.96 (1.23,2.92) | 0.70 (0.44,1.07) | 6.00 (3.27,10.34) | 0.21 (0.13,0.34) | 0.01 (0.00,0.01) |
| Guinea-Bissau | 1.25 (0.76,1.92) | 3.00 (1.94,4.36) | 1.39 (0.87,2.10) | 6.74 (3.33,12.33) | 0.47 (0.30,0.72) | 0.01 (0.00,0.02) |
| Guyana | 0.45 (0.31,0.65) | 1.83 (1.29,2.51) | 2.62 (1.81,3.60) | 0.57 (0.41,0.77) | 0.98 (0.71,1.32) | 0.2 (0.15,0.27) |
| Haiti | 0.67 (0.41,1.04) | 3.49 (2.14,5.13) | 2.20 (1.31,3.45) | 0.26 (0.12,0.56) | 0.60 (0.37,0.91) | 0.22 (0.11,0.36) |
| Honduras | 0.14 (0.08,0.21) | 2.80 (1.72,4.27) | 0.99 (0.58,1.58) | 0.39 (0.21,0.66) | 0.59 (0.34,0.98) | 0.39 (0.22,0.62) |
| Hungary | 0.41 (0.32,0.52) | 1.03 (0.84,1.24) | 2.90 (2.30,3.58) | 0.22 (0.16,0.30) | 1.18 (0.94,1.44) | 0.24 (0.19,0.30) |
| Iceland | 0.59 (0.46,0.74) | 0.63 (0.52,0.74) | 1.16 (0.94,1.42) | 0.34 (0.26,0.45) | 0.99 (0.81,1.20) | 0.08 (0.06,0.09) |
| India | 0.64 (0.55,0.76) | 1.24 (1.06,1.55) | 1.09 (0.93,1.32) | 0.62 (0.52,0.76) | 0.22 (0.19,0.26) | 0.31 (0.20,0.38) |
| Indonesia | 0.26 (0.20,0.35) | 1.41 (1.10,1.87) | 2.26 (1.60,3.05) | 0.98 (0.61,1.56) | 0.62 (0.46,0.83) | 0.10 (0.07,0.17) |
| Iran (Islamic Republic of) | 0.46 (0.40,0.52) | 1.81 (1.46,2.05) | 1.29 (1.11,1.49) | 0.47 (0.40,0.55) | 0.44 (0.39,0.50) | 0.10 (0.05,0.13) |
| Iraq | 0.16 (0.11,0.25) | 1.02 (0.69,1.49) | 1.13 (0.76,1.67) | 0.74 (0.46,1.19) | 0.64 (0.42,0.92) | 0.12 (0.08,0.19) |
| Ireland | 0.56 (0.44,0.69) | 0.52 (0.43,0.61) | 1.28 (1.03,1.54) | 0.29 (0.22,0.37) | 0.69 (0.57,0.81) | 0.05 (0.04,0.06) |
| Israel | 0.16 (0.13,0.19) | 0.55 (0.47,0.65) | 1.31 (1.12,1.53) | 0.25 (0.2,0.32) | 0.73 (0.62,0.86) | 0.05 (0.04,0.06) |
| Italy | 0.20 (0.19,0.21) | 0.89 (0.84,0.94) | 1.41 (1.32,1.51) | 0.43 (0.4,0.47) | 0.79 (0.74,0.84) | 0.15 (0.14,0.15) |
| Jamaica | 0.43 (0.29,0.62) | 1.46 (1.03,2.05) | 2.14 (1.50,2.97) | 0.30 (0.2,0.43) | 0.59 (0.41,0.83) | 0.13 (0.09,0.18) |
| Japan | 0.24 (0.23,0.25) | 1.44 (1.40,1.48) | 1.90 (1.84,1.96) | 0.49 (0.47,0.5) | 0.85 (0.82,0.87) | 0.21 (0.2,0.21) |
| Jordan | 0.11 (0.07,0.16) | 0.64 (0.44,0.90) | 1.44 (0.96,2.12) | 0.21 (0.13,0.33) | 0.42 (0.28,0.63) | 0.13 (0.08,0.20) |
| Kazakhstan | 0.60 (0.52,0.68) | 2.15 (1.88,2.42) | 1.87 (1.63,2.11) | 0.83 (0.67,1.00) | 0.95 (0.78,1.15) | 0.15 (0.13,0.18) |
| Kenya | 1.58 (1.14,2.23) | 1.49 (1.12,1.94) | 1.14 (0.88,1.53) | 0.96 (0.68,1.36) | 0.41 (0.3,0.57) | 0.13 (0.09,0.2) |
| Kiribati | 1.24 (0.71,1.99) | 7.17 (4.54,10.59) | 2.01 (1.29,2.99) | 2.00 (1.12,3.31) | 0.21 (0.13,0.32) | 0.14 (0.05,0.24) |
| Kuwait | 0.09 (0.07,0.12) | 0.30 (0.24,0.39) | 1.19 (0.91,1.56) | 0.11 (0.08,0.15) | 0.46 (0.35,0.6) | 0.10 (0.08,0.13) |
| Kyrgyzstan | 0.35 (0.26,0.45) | 3.25 (2.57,3.98) | 1.65 (1.25,2.09) | 0.44 (0.30,0.62) | 1.00 (0.75,1.28) | 0.12 (0.09,0.16) |
| Lao People's Democratic Republic | 0.45 (0.28,0.71) | 1.79 (1.14,2.67) | 2.64 (1.62,3.96) | 1.75 (0.90,3.15) | 0.51 (0.32,0.75) | 0.13 (0.08,0.23) |
| Latvia | 0.39 (0.3,0.51) | 1.96 (1.58,2.37) | 1.89 (1.46,2.34) | 0.51 (0.38,0.68) | 1.54 (1.23,1.95) | 0.12 (0.09,0.14) |
| Lebanon | 0.10 (0.07,0.15) | 0.79 (0.57,1.06) | 1.24 (0.87,1.72) | 0.38 (0.24,0.57) | 0.46 (0.3,0.66) | 0.12 (0.08,0.19) |
| Lesotho | 3.24 (1.94,4.9) | 2.73 (1.64,4.13) | 2.40 (1.43,3.86) | 5.98 (1.75,17.64) | 0.93 (0.53,1.47) | 0.13 (0.07,0.21) |
| Liberia | 0.86 (0.50,1.39) | 1.58 (0.96,2.35) | 0.77 (0.42,1.33) | 5.47 (2.72,9.73) | 0.35 (0.17,0.6) | 0.01 (0.00,0.01) |
| Libya | 0.26 (0.16,0.40) | 1.35 (0.89,1.99) | 2.44 (1.57,3.57) | 1.60 (0.93,2.62) | 1.13 (0.72,1.75) | 0.45 (0.28,0.72) |
| Lithuania | 0.55 (0.43,0.71) | 2.22 (1.78,2.65) | 1.97 (1.54,2.46) | 0.57 (0.43,0.73) | 1.56 (1.26,1.89) | 0.17 (0.14,0.20) |
| Luxembourg | 0.34 (0.28,0.40) | 0.40 (0.35,0.46) | 1.09 (0.94,1.26) | 0.34 (0.29,0.39) | 0.63 (0.54,0.75) | 0.05 (0.05,0.06) |
| Madagascar | 1.76 (1.07,2.75) | 1.49 (0.95,2.17) | 1.39 (0.91,2.03) | 0.70 (0.38,1.24) | 0.26 (0.16,0.41) | 0.09 (0.05,0.14) |
| Malawi | 4.80 (3.21,7.03) | 0.70 (0.46,1.00) | 0.76 (0.48,1.24) | 1.30 (0.68,2.28) | 0.14 (0.09,0.23) | 0.03 (0.02,0.05) |
| Malaysia | 0.37 (0.25,0.51) | 1.14 (0.85,1.49) | 2.83 (2.08,3.76) | 1.25 (0.71,2.03) | 0.36 (0.27,0.48) | 0.12 (0.07,0.17) |
| Maldives | 0.17 (0.11,0.25) | 0.45 (0.3,0.64) | 0.61 (0.41,0.87) | 0.56 (0.29,1.00) | 0.22 (0.15,0.33) | 0.04 (0.03,0.08) |
| Mali | 0.34 (0.22,0.51) | 3.24 (2.14,4.6) | 1.02 (0.67,1.49) | 7.38 (4.16,12.23) | 0.33 (0.21,0.49) | 0.03 (0.02,0.04) |
| Malta | 0.33 (0.26,0.42) | 0.62 (0.51,0.74) | 1.30 (1.06,1.57) | 0.29 (0.22,0.37) | 1.02 (0.82,1.23) | 0.06 (0.05,0.07) |
| Marshall Islands | 0.52 (0.31,0.82) | 5.87 (3.9,8.19) | 2.69 (1.63,4.01) | 1.29 (0.63,2.52) | 0.72 (0.44,1.15) | 0.13 (0.06,0.23) |
| Mauritania | 0.62 (0.36,0.99) | 1.11 (0.72,1.64) | 0.81 (0.52,1.25) | 5.12 (2.01,9.75) | 0.38 (0.23,0.61) | 0.01 (0.00,0.01) |
| Mauritius | 0.77 (0.66,0.88) | 2.55 (2.23,2.86) | 3.10 (2.67,3.53) | 0.14 (0.12,0.16) | 1.01 (0.87,1.15) | 0.08 (0.07,0.09) |
| Mexico | 0.25 (0.22,0.29) | 2.14 (1.88,2.4) | 2.03 (1.79,2.28) | 0.39 (0.34,0.44) | 0.73 (0.65,0.83) | 0.27 (0.23,0.31) |
| Micronesia (Federated States of) | 0.57 (0.34,0.92) | 5.41 (3.49,7.89) | 2.63 (1.58,4.12) | 1.75 (0.87,3.28) | 0.80 (0.48,1.21) | 0.12 (0.06,0.19) |
| Monaco | 0.86 (0.52,1.29) | 1.17 (0.73,1.80) | 3.17 (2.00,4.64) | 1.12 (0.59,1.92) | 1.49 (0.73,2.50) | 0.06 (0.03,0.09) |
| Mongolia | 1.94 (1.37,2.60) | 7.28 (5.46,10.02) | 1.80 (1.34,2.36) | 11.84 (7.74,17.48) | 1.43 (1.01,1.95) | 0.38 (0.25,0.55) |
| Montenegro | 0.37 (0.28,0.48) | 1.00 (0.79,1.29) | 1.69 (1.29,2.20) | 0.85 (0.62,1.18) | 1.18 (0.89,1.52) | 0.10 (0.07,0.14) |
| Morocco | 0.10 (0.06,0.16) | 0.41 (0.26,0.63) | 1.47 (0.91,2.34) | 0.10 (0.05,0.17) | 0.19 (0.12,0.308) | 0.08 (0.04,0.12) |
| Mozambique | 0.99 (0.60,1.55) | 1.08 (0.68,1.69) | 0.38 (0.23,0.57) | 3.88 (1.54,9.09) | 0.07 (0.05,0.12) | 0.12 (0.06,0.21) |
| Myanmar | 0.41 (0.26,0.62) | 1.54 (1.02,2.24) | 2.32 (1.51,3.42) | 0.81 (0.37,1.78) | 0.49 (0.32,0.72) | 0.10 (0.06,0.18) |
| Namibia | 0.45 (0.27,0.74) | 0.59 (0.35,0.90) | 1.19 (0.71,1.80) | 0.80 (0.43,1.43) | 0.25 (0.14,0.38) | 0.06 (0.03,0.10) |
| Nauru | 0.74 (0.44,1.19) | 7.01 (4.56,9.94) | 3.84 (1.86,5.85) | 2.64 (1.26,4.78) | 1.11 (0.54,1.74) | 0.18 (0.08,0.30) |
| Nepal | 0.71 (0.44,1.11) | 1.24 (0.80,1.83) | 0.88 (0.59,1.30) | 0.64 (0.34,1.07) | 0.17 (0.09,0.28) | 0.29 (0.18,0.47) |
| Netherlands | 0.45 (0.37,0.55) | 0.54 (0.46,0.62) | 1.75 (1.45,2.09) | 0.28 (0.23,0.35) | 0.73 (0.62,0.85) | 0.09 (0.08,0.11) |
| New Zealand | 0.27 (0.23,0.32) | 0.85 (0.73,0.98) | 2.20 (1.89,2.54) | 0.89 (0.72,1.12) | 0.73 (0.63,0.84) | 0.16 (0.14,0.19) |
| Nicaragua | 0.11 (0.08,0.16) | 1.86 (1.37,2.51) | 1.17 (0.83,1.59) | 0.45 (0.27,0.69) | 0.41 (0.29,0.55) | 0.25 (0.17,0.39) |
| Niger | 0.50 (0.31,0.80) | 1.56 (0.99,2.32) | 0.61 (0.38,0.95) | 2.41 (1.14,4.85) | 0.18 (0.10,0.31) | 0.01 (0.00,0.01) |
| Nigeria | 0.57 (0.35,0.81) | 0.41 (0.30,0.56) | 0.71 (0.46,0.99) | 0.91 (0.55,1.46) | 0.11 (0.07,0.16) | 0.01 (0.00,0.01) |
| Niue | 0.37 (0.22,0.61) | 2.78 (1.86,4.14) | 2.11 (1.39,3.09) | 1.36 (0.68,2.56) | 0.77 (0.49,1.19) | 0.09 (0.05,0.14) |
| North Macedonia | 0.19 (0.13,0.26) | 1.49 (1.11,1.94) | 1.55 (1.16,2.05) | 0.85 (0.56,1.21) | 0.87 (0.64,1.20) | 0.13 (0.09,0.20) |
| Northern Mariana Islands | 0.35 (0.23,0.52) | 2.90 (2.10,3.92) | 2.39 (1.71,3.26) | 1.18 (0.67,1.94) | 1.01 (0.73,1.37) | 0.05 (0.03,0.10) |
| Norway | 0.23 (0.21,0.25) | 0.37 (0.35,0.40) | 1.43 (1.32,1.55) | 0.55 (0.50,0.61) | 0.66 (0.62,0.70) | 0.08 (0.08,0.09) |
| Oman | 0.14 (0.09,0.20) | 0.53 (0.37,0.75) | 0.34 (0.22,0.54) | 0.52 (0.29,0.88) | 0.17 (0.11,0.27) | 0.05 (0.03,0.08) |
| Pakistan | 1.12 (0.83,1.55) | 1.13 (0.81,1.52) | 1.26 (0.92,1.73) | 0.89 (0.60,1.28) | 0.22 (0.15,0.30) | 0.48 (0.32,0.73) |
| Palau | 0.60 (0.37,0.92) | 5.97 (3.97,8.67) | 1.89 (1.27,2.71) | 3.43 (1.70,6.17) | 1.03 (0.69,1.51) | 0.04 (0.02,0.06) |
| Palestine | 0.09 (0.06,0.12) | 0.78 (0.56,1.03) | 2.08 (1.52,2.80) | 0.67 (0.43,1.01) | 0.54 (0.38,0.73) | 0.12 (0.07,0.17) |
| Panama | 0.15 (0.12,0.20) | 1.88 (1.45,2.34) | 2.11 (1.64,2.64) | 0.43 (0.33,0.53) | 0.44 (0.34,0.55) | 0.14 (0.11,0.18) |
| Papua New Guinea | 0.25 (0.15,0.40) | 3.56 (2.34,5.25) | 0.86 (0.57,1.23) | 0.68 (0.24,1.85) | 0.30 (0.18,0.50) | 0.08 (0.04,0.13) |
| Paraguay | 0.59 (0.37,0.89) | 1.30 (0.88,1.88) | 1.60 (1.11,2.21) | 0.51 (0.30,0.77) | 0.61 (0.41,0.87) | 0.21 (0.13,0.31) |
| Peru | 0.15 (0.10,0.22) | 3.44 (2.33,4.78) | 1.38 (0.95,1.92) | 0.41 (0.24,0.65) | 0.63 (0.43,0.9) | 0.43 (0.28,0.66) |
| Philippines | 0.29 (0.24,0.35) | 1.07 (0.87,1.36) | 3.22 (2.67,3.80) | 1.88 (1.5,2.34) | 0.68 (0.56,0.81) | 0.10 (0.06,0.12) |
| Poland | 0.44 (0.40,0.48) | 1.29 (1.18,1.41) | 2.25 (2.05,2.47) | 0.27 (0.24,0.3) | 1.01 (0.91,1.10) | 0.25 (0.23,0.27) |
| Portugal | 0.52 (0.41,0.65) | 1.51 (1.29,1.76) | 2.09 (1.72,2.54) | 0.60 (0.46,0.77) | 0.78 (0.65,0.93) | 0.11 (0.09,0.14) |
| Puerto Rico | 0.47 (0.34,0.63) | 0.77 (0.60,0.97) | 2.56 (1.96,3.25) | 0.60 (0.43,0.82) | 1.07 (0.83,1.38) | 0.10 (0.08,0.13) |
| Qatar | 0.18 (0.11,0.27) | 0.44 (0.29,0.66) | 0.91 (0.6,1.36) | 0.87 (0.5,1.43) | 0.43 (0.27,0.68) | 0.07 (0.04,0.13) |
| Republic of Korea | 0.15 (0.11,0.20) | 2.31 (1.79,3.03) | 1.56 (1.23,1.99) | 2.57 (1.75,3.68) | 0.7 (0.53,0.89) | 0.32 (0.23,0.46) |
| Republic of Moldova | 0.22 (0.19,0.26) | 1.52 (1.31,1.76) | 2.08 (1.73,2.48) | 0.57 (0.49,0.67) | 1.34 (1.14,1.58) | 0.08 (0.06,0.09) |
| Romania | 0.48 (0.37,0.62) | 1.75 (1.39,2.15) | 2.65 (2.17,3.23) | 0.38 (0.28,0.51) | 1.36 (1.1,1.67) | 0.14 (0.12,0.18) |
| Russian Federation | 0.58 (0.52,0.63) | 2.42 (2.21,2.62) | 2.36 (2.15,2.57) | 0.53 (0.48,0.58) | 1.38 (1.26,1.51) | 0.14 (0.13,0.15) |
| Rwanda | 1.61 (0.99,2.43) | 1.32 (0.84,1.97) | 1.40 (0.84,2.18) | 0.93 (0.50,1.63) | 0.33 (0.2,0.56) | 0.10 (0.06,0.17) |
| Saint Kitts and Nevis | 0.47 (0.35,0.63) | 0.98 (0.72,1.32) | 1.62 (1.20,2.18) | 0.40 (0.29,0.53) | 0.73 (0.54,0.97) | 0.09 (0.07,0.12) |
| Saint Lucia | 0.81 (0.63,1.02) | 2.00 (1.59,2.50) | 1.77 (1.40,2.21) | 0.38 (0.30,0.47) | 1.05 (0.83,1.33) | 0.13 (0.10,0.16) |
| Saint Vincent and the Grenadines | 0.55 (0.45,0.66) | 2.13 (1.78,2.53) | 2.41 (1.99,2.94) | 0.67 (0.56,0.80) | 1.08 (0.90,1.31) | 0.15 (0.13,0.18) |
| Samoa | 0.16 (0.10,0.25) | 2.39 (1.53,3.52) | 1.58 (1.03,2.27) | 1.13 (0.59,2.03) | 0.58 (0.38,0.88) | 0.11 (0.06,0.17) |
| San Marino | 0.11 (0.05,0.19) | 1.16 (0.62,1.86) | 1.07 (0.55,1.67) | 0.28 (0.13,0.52) | 0.57 (0.29,0.95) | 0.10 (0.04,0.16) |
| Sao Tome and Principe | 0.60 (0.36,0.98) | 1.57 (1.00,2.41) | 1.07 (0.62,1.75) | 0.76 (0.33,1.64) | 0.12 (0.07,0.20) | 0.12 (0.07,0.21) |
| Saudi Arabia | 0.25 (0.16,0.38) | 0.68 (0.43,1.15) | 1.52 (0.98,2.26) | 0.60 (0.35,1.00) | 0.44 (0.29,0.65) | 0.20 (0.13,0.33) |
| Senegal | 0.69 (0.43,1.06) | 1.52 (1.00,2.18) | 0.87 (0.57,1.37) | 2.61 (1.45,4.37) | 0.37 (0.22,0.59) | 0.01 (0.00,0.01) |
| Serbia | 0.30 (0.20,0.43) | 1.00 (0.71,1.34) | 2.10 (1.52,2.84) | 0.59 (0.37,0.89) | 0.91 (0.64,1.24) | 0.13 (0.08,0.20) |
| Seychelles | 1.01 (0.68,1.46) | 1.42 (1.04,1.91) | 3.90 (2.87,5.17) | 1.00 (0.58,1.62) | 0.99 (0.68,1.39) | 0.10 (0.07,0.15) |
| Sierra Leone | 0.70 (0.42,1.11) | 1.62 (1.02,2.38) | 0.73 (0.44,1.15) | 2.82 (1.43,5.16) | 0.28 (0.16,0.44) | 0.01 (0.00,0.01) |
| Singapore | 0.12 (0.10,0.14) | 0.51 (0.43,0.59) | 1.33 (1.12,1.57) | 0.47 (0.37,0.60) | 0.37 (0.31,0.44) | 0.04 (0.04,0.05) |
| Slovakia | 0.53 (0.34,0.81) | 1.14 (0.82,1.56) | 2.41 (1.72,3.25) | 0.52 (0.31,0.86) | 1.12 (0.76,1.59) | 0.25 (0.17,0.37) |
| Slovenia | 0.31 (0.23,0.40) | 0.66 (0.52,0.82) | 1.37 (1.04,1.79) | 0.33 (0.23,0.45) | 0.69 (0.53,0.86) | 0.11 (0.08,0.14) |
| Solomon Islands | 0.54 (0.32,0.85) | 6.62 (4.26,9.69) | 2.26 (1.40,3.46) | 1.88 (0.92,3.49) | 0.58 (0.36,0.88) | 0.12 (0.06,0.21) |
| Somalia | 2.35 (1.41,3.78) | 2.64 (1.59,3.96) | 1.63 (0.90,2.76) | 2.03 (0.8,4.35) | 0.21 (0.09,0.39) | 0.10 (0.05,0.17) |
| South Africa | 1.86 (1.58,2.20) | 1.24 (1.06,1.57) | 2.01 (1.71,2.44) | 2.10 (1.70,2.59) | 0.83 (0.71,0.96) | 0.11 (0.08,0.14) |
| South Sudan | 2.31 (1.41,3.48) | 1.93 (1.23,2.89) | 1.75 (1.10,2.68) | 1.45 (0.76,2.55) | 0.35 (0.18,0.58) | 0.10 (0.05,0.17) |
| Spain | 0.30 (0.24,0.37) | 0.85 (0.73,1.00) | 1.41 (1.19,1.66) | 0.54 (0.42,0.69) | 0.74 (0.63,0.86) | 0.08 (0.07,0.10) |
| Sri Lanka | 0.64 (0.36,0.98) | 0.73 (0.43,1.10) | 0.81 (0.47,1.24) | 0.28 (0.14,0.49) | 0.23 (0.12,0.37) | 0.08 (0.04,0.15) |
| Sudan | 0.66 (0.32,1.09) | 2.98 (1.38,4.70) | 1.50 (0.82,2.44) | 0.67 (0.35,1.12) | 0.38 (0.20,0.65) | 0.14 (0.08,0.24) |
| Suriname | 0.31 (0.20,0.47) | 1.46 (1.00,2.03) | 2.48 (1.71,3.42) | 0.56 (0.30,0.96) | 1.07 (0.68,1.60) | 0.15 (0.10,0.21) |
| Sweden | 0.19 (0.16,0.24) | 0.35 (0.30,0.42) | 1.29 (1.09,1.53) | 0.36 (0.28,0.46) | 0.60 (0.49,0.70) | 0.19 (0.16,0.23) |
| Switzerland | 0.29 (0.23,0.36) | 0.46 (0.39,0.54) | 0.82 (0.70,0.98) | 0.28 (0.21,0.36) | 0.48 (0.40,0.58) | 0.05 (0.04,0.06) |
| Syrian Arab Republic | 0.11 (0.08,0.16) | 1.00 (0.66,1.44) | 1.18 (0.80,1.72) | 0.76 (0.49,1.12) | 0.47 (0.32,0.69) | 0.01 (0.01,0.02) |
| Taiwan (Province of China) | 2.28 (1.97,2.63) | 1.40 (1.18,1.64) | 3.65 (3.05,4.33) | 2.25 (1.73,2.87) | 1.12 (0.92,1.34) | 0.28 (0.23,0.34) |
| Tajikistan | 0.77 (0.53,1.12) | 3.04 (2.15,4.33) | 1.14 (0.74,1.87) | 0.78 (0.43,1.28) | 0.26 (0.17,0.38) | 0.01 (0.01,0.02) |
| Thailand | 1.32 (0.85,1.95) | 2.44 (1.26,3.54) | 3.73 (2.54,5.26) | 3.27 (1.95,5.23) | 1.02 (0.70,1.43) | 0.89 (0.44,1.34) |
| Timor-Leste | 0.34 (0.21,0.51) | 1.38 (0.88,2.01) | 1.63 (1.05,2.41) | 0.63 (0.31,1.20) | 0.32 (0.21,0.49) | 0.09 (0.05,0.15) |
| Togo | 0.89 (0.52,1.39) | 1.83 (1.11,2.72) | 0.86 (0.50,1.35) | 2.28 (1.17,4.39) | 0.36 (0.21,0.56) | 0.01 (0.00,0.01) |
| Tokelau | 0.30 (0.18,0.48) | 3.00 (2.03,4.36) | 2.00 (1.34,2.95) | 1.46 (0.71,2.77) | 0.62 (0.37,1.02) | 0.10 (0.06,0.16) |
| Tonga | 0.29 (0.18,0.46) | 4.04 (2.58,6.06) | 1.09 (0.71,1.62) | 6.65 (3.53,11.59) | 0.94 (0.58,1.45) | 0.07 (0.04,0.11) |
| Trinidad and Tobago | 0.38 (0.27,0.52) | 1.12 (0.82,1.49) | 2.70 (1.99,3.53) | 0.47 (0.34,0.62) | 0.91 (0.64,1.22) | 0.16 (0.11,0.21) |
| Tunisia | 0.08 (0.05,0.12) | 0.78 (0.50,1.14) | 1.10 (0.72,1.60) | 0.34 (0.19,0.58) | 0.35 (0.22,0.53) | 0.22 (0.14,0.34) |
| Turkey | 0.25 (0.16,0.35) | 1.93 (1.24,2.70) | 2.04 (1.44,2.79) | 0.39 (0.25,0.59) | 0.94 (0.66,1.29) | 0.14 (0.09,0.20) |
| Turkmenistan | 1.35 (1.02,1.78) | 2.39 (1.82,3.16) | 1.37 (1.04,1.81) | 1.15 (0.83,1.55) | 0.57 (0.42,0.78) | 0.10 (0.07,0.14) |
| Tuvalu | 0.43 (0.26,0.68) | 4.43 (2.99,6.28) | 2.19 (1.48,3.23) | 1.52 (0.81,2.78) | 0.65 (0.43,0.97) | 0.10 (0.06,0.16) |
| Uganda | 2.34 (1.44,3.55) | 1.30 (0.84,1.88) | 1.77 (1.12,2.70) | 1.92 (1.07,3.28) | 0.56 (0.34,0.87) | 0.09 (0.05,0.15) |
| Ukraine | 0.52 (0.32,0.78) | 2.61 (1.74,3.62) | 2.49 (1.68,3.43) | 0.35 (0.23,0.50) | 1.60 (1.06,2.22) | 0.14 (0.09,0.19) |
| United Arab Emirates | 0.14 (0.09,0.21) | 0.53 (0.36,0.79) | 1.00 (0.63,1.61) | 1.06 (0.61,1.71) | 0.66 (0.43,0.97) | 0.19 (0.11,0.30) |
| United Kingdom | 0.69 (0.66,0.71) | 0.55 (0.54,0.57) | 1.90 (1.84,1.96) | 0.75 (0.72,0.78) | 0.78 (0.75,0.80) | 0.09 (0.09,0.1) |
| United Republic of Tanzania | 1.68 (1.07,2.52) | 1.21 (0.80,1.71) | 1.45 (0.91,2.21) | 1.10 (0.60,1.88) | 0.35 (0.21,0.60) | 0.10 (0.05,0.16) |
| United States of America | 0.44 (0.42,0.46) | 0.66 (0.64,0.69) | 2.40 (2.31,2.50) | 0.53 (0.51,0.55) | 0.88 (0.85,0.91) | 0.10 (0.09,0.10) |
| United States Virgin Islands | 0.59 (0.37,0.91) | 1.42 (0.93,2.11) | 3.25 (2.14,5.01) | 0.47 (0.25,0.83) | 1.15 (0.73,1.77) | 0.20 (0.12,0.32) |
| Uruguay | 0.58 (0.47,0.71) | 1.75 (1.48,2.05) | 3.47 (2.84,4.17) | 0.32 (0.24,0.42) | 1.38 (1.15,1.63) | 0.42 (0.35,0.51) |
| Uzbekistan | 0.64 (0.49,0.84) | 1.94 (1.56,2.37) | 1.23 (0.96,1.55) | 0.90 (0.64,1.20) | 0.47 (0.35,0.61) | 0.04 (0.03,0.06) |
| Vanuatu | 0.44 (0.26,0.69) | 5.03 (3.33,7.29) | 2.20 (1.35,3.26) | 1.26 (0.63,2.34) | 0.51 (0.33,0.74) | 0.09 (0.05,0.16) |
| Venezuela (Bolivarian Republic of) | 0.24 (0.17,0.34) | 2.38 (1.75,3.17) | 2.12 (1.51,2.87) | 0.48 (0.35,0.63) | 0.63 (0.42,0.87) | 0.22 (0.15,0.30) |
| Viet Nam | 0.35 (0.21,0.56) | 1.66 (1.11,2.39) | 1.91 (1.26,2.82) | 3.03 (1.60,5.39) | 0.28 (0.18,0.43) | 0.12 (0.07,0.20) |
| Yemen | 0.57 (0.26,0.94) | 3.36 (1.38,5.42) | 1.30 (0.76,2.07) | 0.39 (0.18,0.78) | 0.26 (0.15,0.41) | 0.14 (0.07,0.23) |
| Zambia | 2.79 (1.55,4.99) | 1.67 (1.01,2.68) | 2.48 (1.28,5.97) | 1.09 (0.34,3.28) | 0.50 (0.31,0.78) | 0.12 (0.07,0.19) |
| Zimbabwe | 3.16 (1.93,4.72) | 3.17 (1.97,4.74) | 2.72 (1.73,4.12) | 3.94 (2.12,6.67) | 1.27 (0.83,1.93) | 0.18 (0.11,0.28) |

ASMR, age-standardized mortality rate. UI, uncertainty interval.

## **Table S9.** ASDR of early-onset gastrointestinal cancers in both sexes in 204 countries and territories in 2021.

| **Location** | **Esophageal cancer (95%UI)** | **Stomach cancer (95%UI)** | **Colorectal cancer (95%UI)** | **Liver cancer (95%UI)** | **Pancreatic cancer (95%UI)** | **Gallbladder and biliary tract cancer (95%UI)** |
| --- | --- | --- | --- | --- | --- | --- |
| Afghanistan | 66.59 (27.73,115.29) | 437.21 (210.54,683.55) | 147.26 (54.66,257.19) | 70.21 (40.15,112.47) | 22.02 (10.05,42.31) | 14.63 (4.74,27.15) |
| Albania | 10.91 (7.17,16.15) | 73.54 (52.80,100.6) | 41.59 (29.51,59.58) | 54.33 (31.43,87.32) | 31.14 (21.42,43.62) | 4.59 (2.72,7.41) |
| Algeria | 4.13 (2.76,5.84) | 32.08 (22.42,44.01) | 32.10 (21.93,45.88) | 17.86 (10.90,28.24) | 9.81 (6.77,13.87) | 18.04 (11.89,27.12) |
| American Samoa | 14.07 (8.36,21.41) | 250.85 (168.75,349.87) | 152.5 (104.93,214.93) | 94.53 (57.06,147.47) | 42.45 (27.77,61.34) | 4.93 (3.09,7.96) |
| Andorra | 7.96 (4.47,13.08) | 43.64 (26.07,65.66) | 78.80 (46.64,119.57) | 62.47 (32.95,103.67) | 51.47 (30.42,79.29) | 6.58 (3.73,9.94) |
| Angola | 55.90 (32.72,86.34) | 77.93 (50.15,113.35) | 66.75 (41.24,102.13) | 93.29 (19.64,262.09) | 18.01 (9.64,30.56) | 2.13 (1.27,3.35) |
| Antigua and Barbuda | 13.17 (11.21,15.26) | 49.94 (43.40,58.03) | 68.13 (59.00,78.19) | 16.20 (13.62,19.15) | 24.08 (20.53,28.14) | 4.17 (3.59,4.82) |
| Argentina | 18.41 (14.96,22.56) | 65.79 (55.75,76.92) | 133.12 (110.46,158.87) | 9.37 (7.37,11.88) | 46.35 (39.07,54.55) | 19.05 (16.06,22.56) |
| Armenia | 4.20 (3.68,4.78) | 79.45 (69.9,89.84) | 80.49 (70.10,91.76) | 44.19 (35.66,53.34) | 66.25 (53.25,79.68) | 5.15 (4.17,6.17) |
| Australia | 19.04 (15.46,23.59) | 29.35 (25.28,33.70) | 102.05 (84.00,121.80) | 37.87 (29.49,48.46) | 35.32 (29.89,41.42) | 4.74 (4.18,5.29) |
| Austria | 11.87 (9.40,14.82) | 29.92 (25.44,35.00) | 58.17 (48.48,69.52) | 22.89 (17.9,28.83) | 37.36 (31.34,43.87) | 5.70 (4.79,6.78) |
| Azerbaijan | 28.40 (21.24,36.41) | 111.25 (83.41,155.67) | 71.46 (52.37,95.77) | 60.17 (31.43,107.13) | 31.49 (20.41,47.88) | 2.74 (1.48,4.69) |
| Bahamas | 55.70 (41.89,73.79) | 98.71 (75.8,129.96) | 184.14 (138.32,245.36) | 36.26 (27.82,47.09) | 42.00 (31.76,54.45) | 12.74 (9.66,16.59) |
| Bahrain | 8.20 (5.44,12.21) | 29.78 (21.15,40.62) | 59.35 (40.09,89.56) | 20.6 (12.00,33.31) | 25.56 (16.71,38.45) | 2.91 (1.64,4.68) |
| Bangladesh | 27.71 (16.57,45.36) | 48.97 (31.86,71.15) | 34.36 (21.29,57.48) | 21.51 (11.51,39.69) | 7.38 (4.10,13.64) | 11.47 (6.53,20.61) |
| Barbados | 34.81 (25.59,46.34) | 69.37 (51.41,91.42) | 132.92 (98.98,176.04) | 18.64 (13.94,24.7) | 42.96 (32.28,57.05) | 6.69 (5.01,8.82) |
| Belarus | 23.35 (16.95,31.27) | 139.57 (105.61,177.09) | 103.82 (74.74,136.65) | 26.17 (18.10,36.35) | 57.53 (43.38,74.39) | 6.25 (4.67,8.16) |
| Belgium | 22.40 (17.90,27.58) | 31.61 (26.9,37.13) | 70.60 (58.13,85.12) | 21.31 (16.57,26.92) | 43.14 (36.58,50.78) | 3.88 (3.23,4.61) |
| Belize | 19.25 (15.81,23.07) | 89.49 (75.66,105.82) | 77.30 (65.00,91.27) | 25.81 (21.73,30.27) | 41.19 (34.46,48.77) | 7.46 (6.21,8.78) |
| Benin | 32.88 (19.52,50.21) | 67.53 (43.08,96.90) | 34.96 (21.82,54.57) | 191.01 (100.88,327.65) | 15.79 (9.13,25.37) | 0.28 (0.09,0.46) |
| Bermuda | 26.30 (18.91,35.76) | 30.21 (22.95,38.61) | 110.91 (79.89,146.31) | 11.11 (8.70,13.81) | 46.86 (36.67,58.20) | 2.85 (2.16,3.73) |
| Bhutan | 28.77 (17.64,44.97) | 49.24 (30.39,76.48) | 40.34 (25.20,60.73) | 38.91 (19.62,68.06) | 9.15 (4.41,15.09) | 12.23 (7.21,21.31) |
| Bolivia (Plurinational State of) | 11.15 (6.85,17.17) | 212.78 (139.21,310.13) | 88.76 (55.54,135.76) | 23.65 (13.42,39.75) | 34.31 (22.44,52.04) | 26.5 (16.44,40.81) |
| Bosnia and Herzegovina | 14.31 (9.37,19.38) | 53.33 (35.44,69.18) | 86.55 (57.66,113.95) | 26.63 (16.72,37.41) | 42.97 (29.44,57.58) | 9.72 (5.96,17.08) |
| Botswana | 60.98 (37.28,91.62) | 43.72 (27.52,64.05) | 69.80 (41.63,121.13) | 87.35 (33.86,223.74) | 29.09 (16.24,52) | 3.86 (2.05,6.94) |
| Brazil | 43.83 (40.54,47.19) | 97.53 (91.97,103.63) | 118.29 (109.96,127.2) | 14.89 (13.85,15.96) | 39.99 (37.5,42.52) | 15.67 (14.79,16.62) |
| Brunei Darussalam | 10.56 (7.47,14.81) | 101.74 (74.27,132.63) | 166.18 (124.03,219.61) | 78.68 (48.35,125.3) | 32.10 (23.97,42.33) | 9.81 (6.53,13.67) |
| Bulgaria | 21.76 (17.64,26.28) | 90.03 (71.14,111.38) | 168.33 (133.2,208.77) | 32.39 (22.8,44.96) | 69.24 (54.59,85.44) | 6.16 (4.87,7.63) |
| Burkina Faso | 35.66 (20.90,55.57) | 84.60 (53.95,121.77) | 38.16 (24.46,56.63) | 293.17 (112.45,599.21) | 13.83 (8.28,21.66) | 0.34 (0.10,0.55) |
| Burundi | 76.69 (48.82,116.98) | 77.70 (49.64,114.57) | 62.58 (38.64,100.97) | 31.98 (16.35,63.94) | 11.74 (6.81,20.73) | 4.35 (2.27,7.45) |
| Cabo Verde | 110.72 (67.86,171.57) | 113.96 (71.75,196.88) | 40.75 (27.24,59.36) | 189.9 (102.38,327.15) | 48.72 (31.94,74.02) | 0.92 (0.46,1.49) |
| Cambodia | 24.80 (15.82,37.72) | 103.35 (67.09,152.13) | 137.44 (89.02,205.41) | 73.11 (31.77,155.51) | 26.31 (17,38.91) | 7.14 (4.26,12.34) |
| Cameroon | 47.03 (26.32,77.73) | 78.95 (45.71,120.27) | 51.14 (30.46,80.17) | 197.98 (92.41,393.12) | 25.58 (14.81,40.47) | 0.40 (0.11,0.67) |
| Canada | 20.61 (16.62,25.44) | 31.76 (26.89,37.03) | 109.17 (91.6,131.27) | 25.49 (20.33,31.66) | 33.53 (28.68,38.9) | 4.85 (4.05,5.80) |
| Central African Republic | 89.01 (50.21,141.97) | 139.19 (86.26,209.6) | 83.35 (46.07,134.90) | 88.83 (32.16,196.81) | 16.85 (9.91,26.44) | 2.06 (1.00,3.80) |
| Chad | 34.85 (21.43,54.38) | 98.38 (64.18,141.37) | 41.41 (26.86,61.38) | 194.64 (92.90,373.18) | 12.44 (8.04,18.30) | 0.34 (0.12,0.54) |
| Chile | 12.98 (10.71,15.8) | 99.34 (82.47,118.01) | 89.73 (74.85,107.08) | 13.33 (10.59,16.68) | 31.42 (26.48,36.80) | 34.86 (29.37,40.6) |
| China | 70.61 (54.32,90.48) | 178.7 (138.69,228.55) | 152.77 (120.98,189.65) | 165.19 (126.03,215.96) | 50.9 (39.43,63.84) | 11.25 (7.31,14.81) |
| Colombia | 11.02 (8.31,14.33) | 154.27 (121.77,191.75) | 108.27 (83.13,138.8) | 13.54 (10.18,17.31) | 28.34 (22.15,35.71) | 13.09 (10.27,16.56) |
| Comoros | 89.71 (58.17,135.37) | 70.26 (46.75,102.70) | 77.58 (48.19,120.62) | 53.69 (28.84,92.32) | 19.42 (11.67,31.14) | 4.72 (2.66,8.03) |
| Congo | 78.28 (45.87,128.17) | 84.31 (51.82,127.95) | 84.70 (53.36,131.74) | 85.1 (32.35,183.17) | 25.64 (14.25,41.36) | 2.52 (1.37,4.00) |
| Cook Islands | 18.08 (10.68,29.03) | 75.58 (49.49,110.75) | 47.33 (31.66,70.22) | 141.57 (79.08,233.63) | 27.88 (18.06,41.19) | 3.11 (1.66,5.53) |
| Costa Rica | 10.17 (7.94,12.87) | 165.27 (135.98,198.72) | 155.72 (123.82,192.28) | 34.94 (26.76,44.89) | 33.96 (27.49,40.97) | 10.82 (8.70,13.13) |
| Croatia | 18.25 (13.91,23.90) | 46.39 (37.25,56.96) | 108.05 (85.65,134.18) | 11.68 (8.12,16.30) | 35.87 (28.93,43.72) | 6.29 (4.97,7.78) |
| Cuba | 34.57 (26.03,45.55) | 37.20 (29.59,45.79) | 105.90 (82.43,133.73) | 13.25 (9.97,17.18) | 33.37 (26.18,42.31) | 4.33 (3.41,5.37) |
| Cyprus | 6.23 (4.15,9.18) | 24.97 (18.11,33.40) | 42.55 (30.91,58.35) | 12.51 (7.63,20.12) | 24.50 (16.95,35.38) | 3.54 (2.35,5.77) |
| Czechia | 18.28 (14.01,23.32) | 33.22 (26.09,41.53) | 102.49 (80.25,128.89) | 12.11 (8.73,16.52) | 52.24 (40.92,65.08) | 10.47 (8.31,12.98) |
| C么te d'Ivoire | 8.98 (5.40,14.00) | 30.19 (17.83,45.89) | 29.17 (17.66,46.49) | 49.44 (24.70,93.12) | 11.11 (6.51,17.96) | 0.36 (0.10,0.63) |
| Democratic People's Republic of Korea | 77.95 (44.00,127.76) | 269.61 (171.14,405.26) | 147.15 (84.71,254.36) | 147.57 (72.62,276.91) | 42.76 (24.50,68.91) | 12.84 (7.22,21.36) |
| Democratic Republic of the Congo | 53.63 (31.86,82.82) | 75.24 (48.95,109.67) | 48.46 (28.64,82.16) | 35.66 (14.73,88.86) | 13.07 (7.19,22.13) | 1.59 (0.90,2.67) |
| Denmark | 19.89 (16.00,24.46) | 23.73 (19.85,28.27) | 61.97 (50.81,74.44) | 8.51 (6.63,10.76) | 30.20 (25.15,35.9) | 2.41 (1.97,2.87) |
| Djibouti | 81.61 (47.84,131.85) | 65.87 (39.92,100.65) | 87.61 (51.53,141.03) | 49.08 (24.20,88.49) | 18.12 (9.61,31.87) | 4.31 (2.18,8.10) |
| Dominica | 34.26 (22.82,51.60) | 140.14 (97.54,196.34) | 94.54 (65.77,133.23) | 24.80 (14.49,39.91) | 52.44 (36.07,74.79) | 9.39 (6.23,13.87) |
| Dominican Republic | 21.45 (14.05,31.19) | 74.95 (52.78,103.71) | 86.75 (60.32,119.96) | 22.93 (13.71,36.17) | 48.35 (33.6,66.56) | 6.28 (4.18,9.02) |
| Ecuador | 7.47 (5.31,9.95) | 177.59 (132.73,236.74) | 82.63 (59.6,111.44) | 22.82 (16.11,31.26) | 27.23 (20.22,36.43) | 15.05 (11.12,19.95) |
| Egypt | 6.05 (4.08,8.62) | 58.11 (30.19,81.29) | 81.71 (57.58,111.94) | 105.63 (69.75,150.43) | 29.12 (20.59,39.75) | 6.38 (3.89,9.02) |
| El Salvador | 14.89 (10.83,19.86) | 197.96 (148.23,263.9) | 102.6 (75.59,136.73) | 15.26 (11.00,20.80) | 36.3 (27.02,48.46) | 16.55 (11.59,23.21) |
| Equatorial Guinea | 61.51 (33.16,103.27) | 55.03 (31.11,89.88) | 80.82 (44.25,132.19) | 59.11 (26.96,115.39) | 29.64 (15.31,50.28) | 2.07 (1.03,3.73) |
| Eritrea | 116.12 (66.92,189.47) | 115.67 (70.58,174.41) | 103.36 (64.74,163.78) | 44.64 (22.69,84.89) | 17.81 (9.23,28.42) | 5.98 (3.29,9.84) |
| Estonia | 12.59 (9.38,16.21) | 61.14 (49.64,72.82) | 76.94 (58.63,94.60) | 24.19 (18.45,31.02) | 48.49 (37.58,60.56) | 4.76 (3.81,5.89) |
| Eswatini | 173.63 (95.82,275.32) | 106.68 (61.06,167.65) | 149.34 (79.67,235.29) | 389.6 (103.71,1047.32) | 61.12 (30.76,105.89) | 6.73 (3.41,11.20) |
| Ethiopia | 32.23 (23.97,44.27) | 74.16 (54.27,100.6) | 93.93 (73.08,122.36) | 25.34 (16.47,40.60) | 7.18 (4.25,11.25) | 7.08 (4.61,11.35) |
| Fiji | 18.79 (11.24,29.97) | 93.67 (41.92,141.6) | 79.72 (50.90,116.08) | 61.05 (32.78,102.90) | 23.07 (15.26,33.28) | 6.92 (3.84,11.00) |
| Finland | 14.96 (13.12,16.94) | 24.84 (20.98,29.26) | 49.66 (40.12,60.80) | 18.15 (13.96,22.97) | 37.10 (31.30,43.60) | 5.70 (4.74,6.86) |
| France | 19.85 (15.72,24.91) | 38.23 (32.07,45.52) | 80.15 (65.33,97.52) | 30.87 (23.70,39.53) | 44.48 (36.85,52.75) | 4.24 (3.48,5.10) |
| Gabon | 72.81 (41.04,117.46) | 65.92 (40.78,101.05) | 105.44 (64.1,161.90) | 109.07 (47.57,213.33) | 34.16 (20.49,53.51) | 2.27 (1.24,3.71) |
| Gambia | 13.15 (8.30,19.82) | 31.14 (20.01,46.61) | 22.17 (14.01,33.64) | 546.18 (269.19,1016.57) | 13.05 (8.34,19.96) | 0.00 (0.00,0.00) |
| Georgia | 6.30 (5.42,7.32) | 93.17 (80.54,107.27) | 103.06 (88.54,119.18) | 33.87 (28.36,40.55) | 53.8 (45.14,63.25) | 6.10 (4.64,7.72) |
| Germany | 20.14 (16.26,24.96) | 45.15 (38.81,51.83) | 80.79 (65.93,97.43) | 19.91 (15.79,24.59) | 40.51 (34.28,47.21) | 7.03 (5.89,8.34) |
| Ghana | 23.42 (14.31,35.00) | 55.55 (36.6,80.60) | 44.60 (28.64,67.34) | 135.64 (67.11,249.02) | 24.52 (15.73,37.04) | 0.37 (0.12,0.60) |
| Greece | 11.71 (10.23,13.39) | 50.20 (45.42,55.19) | 62.44 (56.31,69.76) | 34.99 (31.05,39.23) | 46.92 (41.87,52.54) | 5.80 (5.19,6.44) |
| Greenland | 64.43 (41.56,93.55) | 88.74 (60.24,124.97) | 178.92 (120.97,243.41) | 38.18 (21.18,63.31) | 93.84 (64.02,131.26) | 6.06 (3.46,13.14) |
| Grenada | 34.26 (25.26,45.5) | 65.01 (50.78,81.76) | 114.66 (87.65,146.7) | 29.44 (21.31,39.28) | 62.86 (50.75,77.04) | 8.15 (6.27,10.41) |
| Guam | 24.98 (18.47,32.73) | 105.1 (80.54,131.02) | 159.49 (125.97,201.23) | 96.56 (69.58,131.00) | 48.6 (38.74,60.87) | 2.85 (2.05,3.87) |
| Guatemala | 12.52 (10.23,14.98) | 266.12 (220.48,316.19) | 80.77 (67.13,97.63) | 42.73 (35.41,50.43) | 24.02 (19.85,29.03) | 12.96 (10.56,15.59) |
| Guinea | 9.00 (5.56,13.98) | 96.51 (60.41,144.13) | 34.65 (21.79,52.80) | 296.10 (161.36,509.48) | 10.38 (6.24,16.52) | 0.33 (0.11,0.57) |
| Guinea-Bissau | 58.26 (35.43,89.56) | 146.11 (94.39,212.99) | 68.28 (42.98,103.37) | 336.19 (166.66,612.21) | 22.85 (14.6,34.85) | 0.51 (0.19,0.89) |
| Guyana | 21.34 (14.52,30.35) | 90.77 (64.06,124.03) | 130.91 (90.75,179.9) | 28.36 (20.12,38.14) | 46.89 (33.76,62.99) | 9.57 (6.97,12.91) |
| Haiti | 31.56 (19.02,48.56) | 171.96 (105.04,252.19) | 108.79 (64.2,170.84) | 12.92 (5.8,27.41.00) | 28.35 (17.41,43.03) | 10.36 (5.43,17.15) |
| Honduras | 6.41 (3.73,10.20) | 135.33 (82.52,207.52) | 48.49 (28.11,78.64) | 18.79 (10.19,31.65) | 27.96 (16.09,46.63) | 18.03 (10.41,29.1) |
| Hungary | 19.12 (15.02,23.99) | 49.49 (40.27,59.81) | 143.69 (114.08,178.25) | 10.93 (8.01,14.59) | 55.41 (44.32,67.76) | 11.29 (8.96,13.93) |
| Iceland | 27.86 (21.91,34.91) | 30.9 (25.66,36.60) | 59.85 (48.56,73.37) | 16.91 (12.82,22.00) | 47.13 (38.66,56.89) | 3.74 (3.03,4.56) |
| India | 30.46 (26.28,36.36) | 60.49 (51.60,75.50) | 53.27 (45.20,64.83) | 30.51 (25.53,37.17) | 10.38 (8.80,12.17) | 14.58 (9.69,17.95) |
| Indonesia | 12.67 (9.53,16.79) | 69.41 (54.01,92.07) | 112.79 (79.67,152.8) | 47.87 (29.9,76.02) | 30.11 (22.28,40.16) | 4.77 (3.43,8.01) |
| Iran (Islamic Republic of) | 22.21 (19.6,25.11) | 89.75 (72.54,101.86) | 66.60 (56.82,76.63) | 23.68 (20.23,27.53) | 21.26 (18.54,23.92) | 5.08 (2.24,6.21) |
| Iraq | 7.71 (5.07,11.68) | 49.38 (33.12,71.99) | 55.67 (37.12,82.64) | 36.20 (22.40,57.76) | 29.89 (19.70,43.28) | 5.85 (3.76,9.17) |
| Ireland | 26.22 (20.90,32.68) | 24.98 (20.87,29.57) | 65.37 (52.9,78.78) | 14.01 (10.84,17.65) | 32.27 (26.97,38.32) | 2.37 (1.96,2.86) |
| Israel | 7.44 (5.94,9.16) | 26.55 (22.51,30.97) | 65.73 (55.89,77.33) | 12.15 (9.48,15.59) | 34.43 (29.23,40.29) | 2.42 (1.98,2.92) |
| Italy | 9.36 (8.73,10.03) | 43.14 (40.76,45.62) | 71.85 (66.84,76.86) | 20.96 (19.22,22.84) | 37.04 (34.88,39.44) | 6.91 (6.48,7.36) |
| Jamaica | 20.22 (13.39,28.75) | 71.58 (50.36,100.00) | 106.9 (75.07,148.9) | 14.48 (9.71,20.81) | 27.66 (19.34,39.01) | 6.17 (4.19,8.71) |
| Japan | 11.12 (10.73,11.54) | 72.00 (69.91,74.15) | 96.75 (93.04,100.48) | 23.32 (22.49,24.21) | 39.90 (38.74,41.12) | 10.02 (9.76,10.29) |
| Jordan | 5.30 (3.45,7.80) | 31.28 (21.59,43.83) | 72.71 (48.43,107.19) | 10.37 (6.33,16.20) | 20.05 (13.26,29.93) | 6.18 (3.92,9.5) |
| Kazakhstan | 28.58 (24.87,32.26) | 105.47 (92.52,118.1) | 93.67 (82.11,105.37) | 40.5 (32.92,48.93) | 45.79 (37.5,55.40) | 7.32 (6.08,8.75) |
| Kenya | 74.27 (53.68,105.88) | 72.29 (54.54,94.76) | 55.81 (43.01,75.45) | 46.82 (33.29,66.98) | 19.38 (14.29,27.18) | 6.17 (4.28,9.54) |
| Kiribati | 60.50 (34.7,97.49) | 363.05 (228.87,536.62) | 101.31 (64.51,150.96) | 99.17 (55.53,163.98) | 10.05 (6.08,15.65) | 6.64 (2.28,11.28) |
| Kuwait | 4.48 (3.32,5.96) | 14.94 (11.73,19.13) | 62.03 (47.48,81.11) | 5.46 (3.99,7.30) | 21.81 (16.86,28.61) | 5.06 (3.90,6.47) |
| Kyrgyzstan | 16.78 (12.42,21.57) | 158.14 (125.26,194.05) | 83.70 (63.89,106.31) | 21.24 (14.45,29.87) | 48.08 (36.36,61.68) | 5.75 (4.31,7.40) |
| Lao People's Democratic Republic | 21.99 (13.65,34.36) | 88.46 (56.41,132.47) | 132.50 (81.04,199.03) | 86.01 (44.39,154.10) | 24.66 (15.68,36.36) | 6.08 (3.62,10.77) |
| Latvia | 17.97 (13.57,23.3) | 93.10 (74.89,112.55) | 92.82 (72.27,115.03) | 24.91 (18.49,33.25) | 72.86 (57.97,91.81) | 5.40 (4.27,6.73) |
| Lebanon | 4.78 (3.16,6.84) | 38.33 (27.79,51.41) | 62.54 (43.68,86.87) | 18.50 (11.49,27.69) | 21.61 (14.01,31.60) | 5.86 (3.91,8.96) |
| Lesotho | 149.8 (89.86,226.53) | 130.46 (78.52,197.09) | 116.27 (69.31,186.8) | 294.16 (85.54,875.95) | 43.62 (24.8,68.64) | 6.27 (3.39,10.14) |
| Liberia | 40.34 (23.26,65.13) | 77.63 (46.9,115.65) | 38.05 (20.63,65.75) | 271.97 (135.37,483.15) | 16.90 (8.03,28.95) | 0.33 (0.10,0.58) |
| Libya | 12.31 (7.64,18.77) | 66.02 (43.73,96.85) | 120.96 (77.87,177.73) | 78.00 (45.25,127.5) | 53.77 (34.13,82.56) | 21.51 (13.45,34.29) |
| Lithuania | 24.90 (19.25,32.38) | 106.95 (85.8,127.59) | 97.57 (76.02,121.80) | 27.29 (20.82,35.39) | 73.22 (59.35,88.83) | 7.80 (6.36,9.5) |
| Luxembourg | 15.68 (13.19,18.65) | 19.24 (16.6,22.24) | 54.80 (46.86,63.37) | 16.4 (13.93,19.11) | 29.45 (25.29,34.58) | 2.47 (2.13,2.84) |
| Madagascar | 83.56 (50.72,130.3) | 73.60 (46.8,107.58) | 69.22 (45.07,100.92) | 35.10 (18.81,61.92) | 12.53 (7.54,19.90) | 4.30 (2.41,6.92) |
| Malawi | 225.62 (150.64,332.48) | 34.05 (22.49,48.82) | 37.66 (23.56,61.71) | 63.84 (33.51,111.41) | 6.88 (4.20,10.93) | 1.41 (0.76,2.43) |
| Malaysia | 17.02 (11.85,23.99) | 55.42 (41.11,72.29) | 139.75 (102.75,185.77) | 60.12 (34.40,97.95) | 17.21 (12.69,22.74) | 5.61 (3.22,8.08) |
| Maldives | 8.22 (5.30,12.10) | 22.27 (15.21,31.94) | 30.99 (20.75,44.76) | 27.68 (14.10,49.48) | 10.80 (7.09,15.84) | 1.99 (1.19,3.92) |
| Mali | 16.04 (10.21,24.14) | 158.92 (104.91,226.09) | 50.05 (32.72,73.54) | 361.33 (203.87,597.5) | 15.53 (10.02,23.38) | 1.30 (0.76,2.13) |
| Malta | 15.30 (11.93,19.31) | 29.78 (24.68,35.54) | 65.08 (52.59,78.73) | 14.14 (10.87,18.21) | 47.42 (38.47,57.5) | 2.81 (2.25,3.44) |
| Marshall Islands | 25.33 (15.00,40.15) | 295.13 (196.02,412.28) | 134.45 (81.19,200.08) | 62.84 (31.04,122.65) | 34.20 (20.79,54.71) | 6.06 (2.84,10.69) |
| Mauritania | 28.74 (16.95,46.22) | 53.87 (35.11,79.56) | 39.45 (25.58,61.21) | 255.68 (100.95,486.57) | 18.12 (11.07,29.48) | 0.27 (0.09,0.48) |
| Mauritius | 36.56 (31.22,41.80) | 125.71 (110.35,141.18) | 155.94 (134.22,177.26) | 6.59 (5.69,7.55) | 48.53 (41.78,55.33) | 3.97 (3.46,4.47) |
| Mexico | 12.28 (10.76,13.96) | 106.20 (93.71,119.10) | 101.88 (89.92,114.57) | 18.93 (16.58,21.37) | 35.24 (31.07,39.67) | 12.9 (11.19,14.76) |
| Micronesia (Federated States of) | 27.66 (16.39,44.65) | 271.09 (174.56,395.73) | 131.20 (78.97,205.10) | 84.99 (42.29,158.90) | 37.8 (22.66,57.40) | 5.56 (3.05,8.92) |
| Monaco | 40.88 (24.5,61.37) | 57.77 (35.68,88.98) | 163.74 (102.09,242.2) | 55.06 (29.3,94.64) | 71.07 (34.3,119.68) | 2.82 (1.66,4.46) |
| Mongolia | 91.80 (65.04,123.51) | 351.97 (264.11,484.87) | 89.54 (66.61,117.88) | 569.50 (372.17,841.8) | 68.23 (48.05,93.24) | 18.04 (11.75,25.88) |
| Montenegro | 17.12 (12.85,22.23) | 48.48 (37.96,62.24) | 83.85 (64.26,109.77) | 41.42 (29.9,57.15) | 55.82 (42.08,71.67) | 4.74 (3.43,6.63) |
| Morocco | 4.75 (2.98,7.57) | 19.49 (12.66,30.30) | 72.61 (44.97,115.82) | 4.80 (2.64,8.36) | 8.85 (5.84,14.05) | 3.54 (2.10,5.88) |
| Mozambique | 45.79 (27.68,72.11) | 51.96 (32.49,81.53) | 18.37 (11.29,27.68) | 185.76 (73.74,435.24) | 3.47 (2.17,5.47) | 5.75 (2.84,10.03) |
| Myanmar | 19.61 (12.36,30.12) | 76.51 (50.71,111.00) | 116.6 (75.28,171.78) | 39.60 (17.97,87.15) | 23.77 (15.34,34.76) | 4.91 (2.92,8.52) |
| Namibia | 20.85 (12.29,34.24) | 28.29 (17.02,43.5) | 57.97 (34.76,88.07) | 39.27 (20.8,70.38) | 11.78 (6.84,17.94) | 3.02 (1.62,4.94) |
| Nauru | 36.01 (21.08,57.68) | 350.59 (228.32,497.2) | 190.99 (92.9,291.38) | 127.59 (60.69,231.22) | 52.54 (25.36,82.72) | 8.46 (3.73,14.37) |
| Nepal | 33.69 (21.04,52.77) | 60.64 (39.13,89.54) | 43.31 (28.68,63.71) | 31.25 (16.85,52.57) | 8.30 (4.50,13.64) | 13.99 (8.62,22.53) |
| Netherlands | 21.12 (17.22,25.83) | 26.06 (22.22,30.2) | 91.00 (75.11,109.00) | 13.73 (10.89,16.94) | 34.41 (29.16,40.06) | 4.47 (3.77,5.29) |
| New Zealand | 12.86 (10.78,15.27) | 42.13 (36.29,48.6) | 111.67 (95.2,130.16) | 43.11 (34.91,54.02) | 34.24 (29.84,39.36) | 7.96 (6.87,9.17) |
| Nicaragua | 5.49 (3.78,7.83) | 91.8 (67.31,123.70) | 59.12 (42.06,80.35) | 22.35 (13.52,34.01) | 19.64 (13.99,26.55) | 11.85 (7.96,18.72) |
| Niger | 23.55 (14.29,37.65) | 76.35 (48.24,113.49) | 30.01 (18.3,46.63) | 120.63 (57.29,241.48) | 8.90 (4.65,14.85) | 0.25 (0.08,0.43) |
| Nigeria | 26.51 (16.39,38.14) | 20.24 (14.45,27.55) | 35.05 (22.69,48.91) | 44.94 (26.88,72.05) | 5.18 (3.51,7.58) | 0.26 (0.10,0.40) |
| Niue | 18.12 (10.74,29.56) | 141.61 (95.17,210.21) | 107.12 (71.04,156.72) | 65.83 (33.05,123.18) | 37.14 (23.62,57.20) | 4.23 (2.51,6.55) |
| North Macedonia | 8.67 (6.16,11.89) | 71.38 (53.62,93.19) | 76.17 (56.86,100.54) | 40.97 (26.94,58.58) | 40.93 (30.15,56.20) | 6.32 (4.23,9.24) |
| Northern Mariana Islands | 16.86 (11.03,25.39) | 145.29 (104.92,196.63) | 120.03 (85.2,163.77) | 55.16 (31.32,90.49) | 47.77 (34.48,65.20) | 2.44 (1.51,4.58) |
| Norway | 10.72 (9.82,11.65) | 18.10 (17.00,19.29) | 72.32 (66.65,78.53) | 27.25 (24.67,30.15) | 30.5 (28.59,32.45) | 4.07 (3.78,4.37) |
| Oman | 6.38 (4.13,9.59) | 26.01 (17.98,36.51) | 17.24 (11.03,27.00) | 25.77 (14.37,43.39) | 7.92 (5.05,12.84) | 2.20 (1.34,3.76) |
| Pakistan | 53.51 (39.22,74.02) | 55.90 (39.96,75.58) | 62.78 (45.75,86.12) | 44.55 (30.18,63.85) | 10.43 (7.35,14.78) | 23.26 (15.51,35.36) |
| Palau | 29.62 (18.3,45.43) | 305.85 (203.93,443.98) | 95.53 (64.05,137.6) | 167.41 (83.07,301.77) | 50.06 (33.08,73.8) | 1.99 (1.20,3.06) |
| Palestine | 4.32 (3.04,6.00) | 38.45 (27.72,50.78) | 105.52 (77.53,142.41) | 32.68 (20.95,49.05) | 25.64 (18.05,34.98) | 5.68 (3.23,8.13) |
| Panama | 7.51 (5.66,9.52) | 93.38 (72.18,115.63) | 109.64 (85.25,136.17) | 21.09 (16.39,26.3) | 21.46 (16.47,26.7) | 6.76 (5.20,8.46) |
| Papua New Guinea | 12.18 (7.42,19.50) | 180.36 (118.5,265.88) | 43.87 (28.97,62.49) | 33.27 (11.85,89.37) | 14.09 (8.46,23.62) | 3.78 (2.05,6.30) |
| Paraguay | 27.48 (17.04,41.40) | 63.26 (42.62,90.94) | 79.27 (54.94,109.73) | 24.94 (14.63,37.85) | 28.84 (19.58,41.12) | 10.01 (6.41,14.77) |
| Peru | 7.21 (4.63,10.66) | 170.97 (115.93,237.7) | 69.41 (48,96.56.00) | 20.87 (12.15,32.94) | 30.39 (20.48,43.15) | 20.33 (13.14,31.09) |
| Philippines | 14.15 (11.63,17.04) | 53.13 (42.83,66.75) | 161.8 (134.06,190.55) | 92.25 (73.9,114.47) | 32.92 (27.26,39.48) | 4.73 (2.95,6.00) |
| Poland | 20.48 (18.35,22.47) | 62.25 (56.70,67.91) | 109.99 (100.19,120.39) | 12.94 (11.53,14.45) | 47.43 (43.15,51.95) | 11.71 (10.62,12.79) |
| Portugal | 24.06 (19.01,30.03) | 72.92 (62.37,85.22) | 105.65 (86.29,128.37) | 28.20 (21.83,36.31) | 36.47 (30.41,43.04) | 5.36 (4.40,6.41) |
| Puerto Rico | 21.97 (16.09,29.52) | 38.32 (29.72,48.23) | 132.2 (101.13,167.67) | 29.98 (21.40,40.87) | 51.33 (39.81,65.83) | 4.92 (3.71,6.33) |
| Qatar | 8.51 (5.24,13.04) | 21.36 (14.32,32.28) | 47.04 (30.77,69.96) | 43.13 (24.76,71.04) | 20.53 (13.04,32.17) | 3.62 (2.09,6.06) |
| Republic of Korea | 6.99 (5.03,9.64) | 115.24 (88.96,151.24) | 79.98 (63.05,102.05) | 121.52 (82.63,174.25) | 32.86 (24.88,42.26) | 15.15 (10.78,21.88) |
| Republic of Moldova | 10.13 (8.57,11.83) | 72.44 (62.36,83.93) | 100.87 (84.53,120.10) | 27.83 (23.74,32.48) | 62.99 (53.58,74.13) | 3.55 (3.02,4.17) |
| Romania | 22.08 (16.88,28.57) | 84.04 (66.56,102.87) | 129.85 (105.79,157.85) | 18.56 (13.60,24.59) | 63.47 (51.62,78.00) | 6.8 (5.42,8.36) |
| Russian Federation | 27.00 (24.42,29.42) | 117.70 (107.75,127.48) | 117.06 (107.01,127.33) | 25.89 (23.6,28.22) | 65.42 (59.69,71.18) | 6.64 (6.07,7.25) |
| Rwanda | 75.79 (46.7,115.12) | 64.67 (41.33,96.78) | 68.96 (41.31,107.99) | 46.2 (25.08,81.08) | 16.05 (9.57,26.77) | 5.02 (2.81,8.39) |
| Saint Kitts and Nevis | 21.69 (15.89,28.95) | 47.17 (34.25,63.67) | 79.56 (58.75,107.40) | 19.13 (13.88,25.47) | 34.09 (25.11,45.90) | 4.27 (3.10,5.79) |
| Saint Lucia | 38.16 (29.69,47.63) | 98.07 (78.16,122.69) | 88.95 (70.71,110.45) | 18.72 (14.71,23.20) | 50.17 (39.49,63.09) | 6.13 (4.89,7.61) |
| Saint Vincent and the Grenadines | 25.36 (20.79,30.68) | 102.89 (86.16,122.51) | 119.46 (98.9,145.94) | 32.90 (27.16,39.26) | 51.07 (42.41,61.91) | 7.16 (6.01,8.59) |
| Samoa | 7.75 (4.83,11.80) | 116.63 (74.61,172.61) | 77.43 (50.35,111.68) | 54.49 (28.41,97.26) | 27.69 (17.92,41.96) | 5.06 (2.89,8.06) |
| San Marino | 5.32 (2.58,9.10) | 56.74 (30.16,90.89) | 54.75 (28.41,85.61) | 13.80 (6.41,25.52) | 26.79 (13.65,44.64) | 4.61 (2.18,7.79) |
| Sao Tome and Principe | 27.82 (16.65,45.56) | 75.4 (47.73,116.77) | 53.22 (30.66,87.65) | 38.45 (16.87,83.32) | 6.09 (3.51,10.13) | 5.83 (3.08,9.99) |
| Saudi Arabia | 11.84 (7.48,18.21) | 33.39 (21.00,56.31) | 75.75 (48.82,113.03) | 29.56 (16.90,48.77) | 21.03 (13.76,31.05) | 9.76 (5.99,15.9) |
| Senegal | 32.42 (20.16,49.79) | 74.03 (48.97,106.07) | 42.82 (27.94,67.38) | 130.69 (72.59,218.29) | 17.71 (10.83,28.52) | 0.32 (0.11,0.56) |
| Serbia | 13.59 (9.14,19.81) | 47.47 (33.63,63.80) | 102.48 (74.3,138.22) | 28.22 (17.70,43.09) | 42.48 (29.99,57.66) | 6.28 (3.93,9.50) |
| Seychelles | 47.48 (31.89,68.94) | 69.45 (50.84,93.77) | 196.11 (144.11,260.82) | 47.75 (27.36,77.89) | 47.6 (32.92,67.13) | 4.58 (3.10,7.21) |
| Sierra Leone | 32.60 (19.6,52.05) | 79.26 (49.88,117.00) | 35.95 (21.81,57.21) | 141.16 (71.9,257.76) | 13.41 (7.82,21.38) | 0.32 (0.10,0.55) |
| Singapore | 5.53 (4.46,6.77) | 25.60 (21.72,30.02) | 67.46 (57.01,79.81) | 22.48 (17.42,28.71) | 17.43 (14.45,20.69) | 2.09 (1.75,2.45) |
| Slovakia | 24.56 (15.86,37.32) | 55.26 (39.80,75.22) | 119.94 (85.6,161.55) | 25.06 (14.97,41.62) | 52.84 (36.03,74.80) | 11.9 (7.88,17.58) |
| Slovenia | 14.43 (10.8,18.82) | 32.00 (25.10,39.93) | 69.43 (52.40,90.95) | 15.98 (11.30,22.01) | 32.41 (24.97,40.38) | 5.22 (3.95,6.71) |
| Solomon Islands | 25.99 (15.29,41.42) | 330.10 (212.27,483.55) | 112.12 (69.32,172) | 90.84 (44.54,167.49) | 27.43 (17.20,41.70) | 5.76 (3.01,10.09) |
| Somalia | 110.48 (66.16,177.26) | 128.37 (77.40,193.16) | 79.83 (44.13,134.84) | 98.91 (38.89,211.35) | 10.01 (4.37,18.51) | 4.53 (2.32,8.17) |
| South Africa | 87.16 (73.97,103.22) | 60.17 (51.38,76.52) | 99.72 (84.69,121.57) | 105.64 (85.5,129.95) | 38.9 (33.24,45.23) | 5.47 (3.87,6.80) |
| South Sudan | 108.67 (66.16,163.9) | 94.30 (60.10,141.33) | 85.89 (53.73,130.95) | 71.34 (37.39,125.19) | 16.53 (8.40,27.70) | 4.66 (2.6,7.92) |
| Spain | 14.39 (11.43,17.75) | 41.83 (35.65,48.98) | 71.72 (60.36,84.80) | 26.17 (20.30,33.09) | 34.78 (29.31,40.58) | 3.95 (3.28,4.68) |
| Sri Lanka | 30.50 (17.3,46.51) | 36.09 (21.33,54.05) | 40.92 (24.03,62.51) | 13.84 (7.08,23.71) | 10.94 (6.03,17.70) | 4.00 (2.11,6.99) |
| Sudan | 31.53 (15.34,52.19) | 147.14 (67.78,232.97) | 75.40 (40.85,122.64) | 33.06 (17.16,55.68) | 18.05 (9.41,31.20) | 7.02 (3.70,11.81) |
| Suriname | 14.58 (9.47,21.92) | 71.87 (49.41,99.83) | 124.44 (85.71,171.35) | 27.67 (14.83,47.05) | 51.25 (32.46,76.18) | 7.05 (4.66,10.19) |
| Sweden | 9.14 (7.42,11.24) | 17.50 (14.66,20.74) | 65.42 (55.11,77.51) | 18.15 (14.31,22.79) | 28.03 (23.04,33.12) | 9.23 (7.62,11.17) |
| Switzerland | 13.85 (10.82,17.21) | 22.20 (18.79,26.33) | 41.92 (35.34,49.55) | 13.35 (10.26,17.22) | 22.75 (19.1,27.55) | 2.27 (1.84,2.76) |
| Syrian Arab Republic | 5.48 (3.81,7.79) | 49.07 (32.60,70.45) | 59.90 (40.58,87.22) | 37.19 (23.88,54.95) | 22.40 (14.89,32.49) | 0.48 (0.24,0.73) |
| Taiwan (Province of China) | 105.38 (90.84,121.13) | 68.42 (57.34,80.18) | 184.25 (153.78,219.69) | 109.21 (84.16,139.37) | 53.25 (43.73,63.59) | 13.53 (11.15,16.28) |
| Tajikistan | 37.44 (25.72,54.08) | 150.67 (106.59,214.74) | 58.22 (37.52,96.43) | 38.87 (21.66,63.72) | 12.94 (8.49,18.90) | 0.60 (0.37,0.92) |
| Thailand | 62.98 (40.47,93.24) | 122.14 (62.85,176.82) | 190.37 (130.05,267.8) | 160.76 (95.83,257.82) | 49.91 (34.03,69.95) | 42.66 (20.96,63.68) |
| Timor-Leste | 16.37 (10.27,24.51) | 67.79 (43.24,99.17) | 81.30 (52.40,120.38) | 30.71 (15.01,58.47) | 15.47 (10.07,23.48) | 4.10 (2.47,7.28) |
| Togo | 41.32 (24.19,65.03) | 88.91 (54.06,132.62) | 42.01 (24.47,66.18) | 113.83 (58.7,217.67) | 17.24 (10.14,26.98) | 0.35 (0.11,0.62) |
| Tokelau | 15.02 (8.96,23.65) | 154.63 (105.41,223.17) | 102.22 (68.94,149.77) | 70.28 (34.65,132.86) | 30.23 (18.06,49.36) | 4.80 (2.74,7.64) |
| Tonga | 14.09 (8.45,22.18) | 203.03 (129.42,305.31) | 54.08 (35.49,80.78) | 318.74 (169.04,558.14) | 44.14 (27.41,68.19) | 3.11 (1.72,5.00) |
| Trinidad and Tobago | 17.96 (12.79,24.44) | 55.44 (40.43,73.42) | 136.97 (101.76,178.37) | 23.30 (17.13,30.56) | 43.40 (30.54,58.53) | 7.52 (5.25,10.14) |
| Tunisia | 3.68 (2.25,5.68) | 38.37 (24.66,55.99) | 55.36 (36.12,80.75) | 16.99 (9.30,28.43) | 16.50 (10.35,25.35) | 10.57 (6.58,16.58) |
| Turkey | 11.87 (7.84,16.80) | 94.41 (60.88,132.57) | 104.00 (73.83,142.19) | 18.99 (12.16,28.66) | 44.57 (31.11,61.26) | 6.75 (4.41,9.72) |
| Turkmenistan | 64.83 (49.13,85.47) | 118.37 (90.14,155.84) | 69.61 (52.93,91.67) | 56.03 (40.85,75.53) | 27.78 (20.56,37.59) | 4.75 (3.50,6.47) |
| Tuvalu | 20.88 (12.53,32.87) | 221.89 (150.06,314.41) | 109.46 (73.44,161.09) | 73.92 (39.33,135.21) | 30.69 (20.18,46.03) | 4.82 (2.79,7.65) |
| Uganda | 110.02 (67.79,167.13) | 63.91 (41.26,92.55) | 87.71 (55.31,133.71) | 94.88 (53.32,161.89) | 26.71 (16.33,41.98) | 4.36 (2.44,7.13) |
| Ukraine | 24.37 (15.12,36.24) | 127.39 (85.42,176.56) | 122.70 (82.5,169.01) | 17.15 (11.18,24.25) | 76.34 (50.86,106.16) | 6.52 (4.34,9.13) |
| United Arab Emirates | 7.01 (4.45,10.41) | 27.33 (18.35,40.25) | 53.22 (33.53,84.88) | 53.80 (30.86,86.75) | 32.30 (20.97,47.32) | 9.64 (5.74,14.96) |
| United Kingdom | 32.73 (31.58,34.00) | 26.85 (26.02,27.68) | 96.40 (93.12,100.06) | 37.25 (35.86,38.74) | 36.85 (35.56,37.99) | 4.56 (4.40,4.71) |
| United Republic of Tanzania | 79.35 (50.43,119.47) | 59.69 (39.24,84.44) | 71.99 (45.02,109.71) | 54.67 (29.85,92.95) | 16.76 (10.01,28.95) | 4.63 (2.61,7.73) |
| United States of America | 20.9 (20.05,21.79) | 32.99 (31.74,34.23) | 121.64 (116.3,127.01) | 26.28 (25.12,27.45) | 41.25 (39.73,42.92) | 4.67 (4.47,4.90) |
| United States Virgin Islands | 28.08 (17.43,42.93) | 71.27 (46.95,105.47) | 170.98 (111.81,265.4) | 22.47 (11.97,40.07) | 56.06 (35.44,86.30) | 10.12 (5.88,16.47) |
| Uruguay | 27.46 (22.19,33.90) | 85.31 (71.97,100.31) | 174.94 (142.57,211.85) | 15.46 (11.45,20.39) | 65.31 (54.32,77.37) | 20.21 (16.71,24.11) |
| Uzbekistan | 31.42 (23.85,40.91) | 96.25 (77.75,117.63) | 62.25 (48.49,78.53) | 44.28 (31.69,59.37) | 22.85 (17.28,29.74) | 2.03 (1.50,2.68) |
| Vanuatu | 21.52 (12.82,33.8) | 252.00 (166.49,365.05) | 109.46 (67.00,162.80) | 61.53 (30.69,114.08) | 24.38 (15.8,35.17) | 4.44 (2.30,7.39) |
| Venezuela (Bolivarian Republic of) | 11.89 (8.28,16.38) | 118.47 (87.26,157.45) | 109.01 (77.55,147.14) | 24.83 (18.35,32.18) | 30.14 (20.34,41.57) | 10.37 (7.13,14.36) |
| Viet Nam | 16.09 (9.75,25.67) | 79.69 (53.19,115.25) | 92.87 (60.73,137.73) | 145.27 (76.55,259.10) | 13.12 (8.38,20.21) | 5.52 (3.11,9.25) |
| Yemen | 26.96 (12.21,44.41) | 162.35 (66.13,262.12) | 63.63 (36.88,101.72) | 19.05 (8.81,38.16) | 12.32 (6.96,19.47) | 6.72 (3.49,10.96) |
| Zambia | 131.28 (72.65,237.45) | 81.93 (49.41,132.41) | 122.5 (62.93,294.93) | 54.28 (16.76,162.45) | 24.13 (14.55,37.26) | 5.78 (3.19,8.99) |
| Zimbabwe | 148.47 (90.65,222.03) | 155.47 (96.56,232.85) | 133.57 (85.17,202.66) | 197.16 (105.95,333.48) | 60.51 (39.5,92.42) | 8.82 (5.19,13.62) |

ASDR, age-standardized disability-adjusted life years rate. UI, uncertainty interval.

## **Table S10.** Percentage rates of incidence early-onset gastrointestinal cancers from 1990 to 2021.

| **Location** | **Esophageal cancer (95%UI)** | **Stomach cancer (95%UI)** | **Colorectal cancer (95%UI)** | **Liver cancer (95%UI)** | **Pancreatic cancer (95%UI)** | **Gallbladder and biliary tract cancer (95%UI)** |
| --- | --- | --- | --- | --- | --- | --- |
| Afghanistan | -0.36 (-0.57,0.07) | -0.30 (-0.54,0.04) | 0.04 (-0.32,0.98) | -0.18 (-0.49,0.32) | 0.24 (-0.23,1.26) | 0.06 (-0.38,1.14) |
| Albania | 0.19 (-0.28,0.94) | -0.17 (-0.41,0.24) | 0.92 (0.26,1.92) | -0.16 (-0.5,0.34) | 0.68 (0.11,1.53) | 0.18 (-0.26,0.75) |
| Algeria | 0.38 (-0.05,1.03) | -0.17 (-0.39,0.09) | 0.80 (0.29,1.60) | 0.74 (0.02,1.94) | 0.90 (0.33,1.76) | 0.28 (-0.1,0.85) |
| American Samoa | 0.97 (0.22,1.98) | 0.2 (-0.14,0.66) | 0.68 (0.15,1.45) | 1.23 (0.42,2.69) | 1.37 (0.53,2.43) | 0.36 (-0.15,1.03) |
| Andorra | 0.11 (-0.42,0.99) | -0.23 (-0.56,0.27) | 0.32 (-0.27,1.13) | 0.44 (-0.23,1.49) | 0.13 (-0.36,0.92) | 0.22 (-0.34,1.12) |
| Angola | -0.38 (-0.61,0.03) | -0.39 (-0.61,-0.11) | 0.18 (-0.23,0.94) | -0.39 (-0.63,0.27) | 0.19 (-0.34,1.14) | 0.11 (-0.34,0.98) |
| Antigua and Barbuda | -0.05 (-0.22,0.11) | -0.44 (-0.51,-0.34) | 0.51 (0.29,0.80) | -0.18 (-0.33,0) | 0.31 (0.12,0.56) | -0.5 (-0.58,-0.41) |
| Argentina | -0.51 (-0.59,-0.42) | -0.33 (-0.43,-0.21) | 0.38 (0.16,0.67) | 1.07 (0.63,1.68) | -0.10 (-0.23,0.06) | -0.28 (-0.37,-0.18) |
| Armenia | -0.65 (-0.7,-0.59) | -0.54 (-0.6,-0.48) | -0.13 (-0.26,0.00) | 0.1 (-0.2,0.44) | 0.67 (0.25,1.23) | 0.99 (0.53,1.60) |
| Australia | 0.35 (0.12,0.63) | -0.21 (-0.32,-0.09) | 0.38 (0.11,0.69) | 1.95 (1.45,2.52) | 0.42 (0.24,0.65) | 0.56 (0.37,0.75) |
| Austria | -0.3 (-0.43,-0.15) | -0.57 (-0.63,-0.51) | -0.18 (-0.32,0.00) | 0.69 (0.4,1.05) | -0.17 (-0.28,-0.05) | -0.32 (-0.43,-0.2) |
| Azerbaijan | -0.35 (-0.52,-0.13) | -0.45 (-0.59,-0.24) | -0.06 (-0.3,0.23) | 0.39 (-0.42,2.21) | 0.8 (-0.01,2.25) | -0.09 (-0.56,0.68) |
| Bahamas | 0.4 (0.06,0.88) | -0.07 (-0.3,0.21) | 0.91 (0.39,1.56) | 0.15 (-0.15,0.51) | 0.63 (0.20,1.23) | -0.11 (-0.34,0.19) |
| Bahrain | -0.04 (-0.34,0.36) | -0.14 (-0.35,0.13) | 1.10 (0.56,1.84) | -0.1 (-0.37,0.32) | 0.75 (0.23,1.48) | 0.48 (0.06,1.14) |
| Bangladesh | -0.21 (-0.47,0.23) | -0.41 (-0.59,-0.17) | 0.14 (-0.28,1.12) | 0.12 (-0.3,0.79) | 0.32 (-0.26,1.20) | 0.21 (-0.26,1.19) |
| Barbados | 0.16 (-0.18,0.59) | -0.28 (-0.47,-0.05) | 0.64 (0.21,1.23) | 0.2 (-0.11,0.61) | 0.25 (-0.07,0.72) | -0.15 (-0.36,0.13) |
| Belarus | 0.3 (-0.06,0.74) | -0.43 (-0.58,-0.28) | 0.48 (0.04,0.98) | 0.45 (0.03,1.00) | 0.95 (0.51,1.47) | 0.35 (0.00,0.79) |
| Belgium | -0.11 (-0.26,0.09) | -0.44 (-0.51,-0.34) | 0.01 (-0.19,0.25) | 0.76 (0.44,1.11) | 0.24 (0.07,0.41) | -0.1 (-0.24,0.05) |
| Belize | 0.97 (0.62,1.38) | 0.19 (0.03,0.4) | 1.61 (1.19,2.12) | 0.95 (0.56,1.35) | 1.23 (0.57,1.93) | -0.13 (-0.35,0.16) |
| Benin | 0.66 (0.08,1.64) | -0.28 (-0.47,0.02) | 0.19 (-0.18,0.75) | -0.36 (-0.62,0.11) | 0.8 (0.15,1.74) | -0.14 (-0.41,0.26) |
| Bermuda | -0.06 (-0.31,0.27) | -0.44 (-0.56,-0.28) | 0.86 (0.28,1.58) | -0.29 (-0.45,-0.1) | 0.16 (-0.13,0.65) | -0.31 (-0.5,-0.07) |
| Bhutan | -0.11 (-0.49,0.53) | -0.29 (-0.55,0.15) | 0.38 (-0.19,1.43) | 0.33 (-0.27,1.60) | 0.85 (0.16,2.20) | 0.38 (-0.18,1.51) |
| Bolivia (Plurinational State of) | -0.26 (-0.52,0.16) | -0.37 (-0.56,-0.1) | 0.22 (-0.19,0.97) | -0.12 (-0.47,0.42) | 0.20 (-0.21,1.04) | -0.22 (-0.51,0.32) |
| Bosnia and Herzegovina | 0.1 (-0.25,0.49) | -0.31 (-0.52,-0.06) | 0.70 (0.12,1.28) | -0.28 (-0.54,0.08) | 0.32 (-0.08,0.75) | -0.22 (-0.53,0.40) |
| Botswana | -0.15 (-0.48,0.4) | -0.37 (-0.59,-0.01) | 0.35 (-0.17,1.22) | 0.44 (-0.42,2.91) | 0.65 (-0.10,1.90) | 0.14 (-0.38,0.99) |
| Brazil | 0 (-0.07,0.08) | -0.18 (-0.22,-0.13) | 1.30 (1.13,1.47) | 0.07 (0.00,0.14) | 0.56 (0.48,0.65) | 0.11 (0.05,0.18) |
| Brunei Darussalam | 0.37 (-0.01,0.93) | -0.33 (-0.5,-0.09) | 0.29 (-0.07,0.80) | 0.05 (-0.42,1.00) | 0.66 (0.18,1.32) | 0.00 (-0.26,0.39) |
| Bulgaria | 0.07 (-0.14,0.33) | -0.39 (-0.54,-0.19) | 0.45 (0.06,1.00) | -0.36 (-0.58,-0.07) | 0.25 (-0.06,0.66) | 0.12 (-0.17,0.55) |
| Burkina Faso | 0.63 (0.01,1.54) | -0.27 (-0.47,0.03) | 0.15 (-0.20,0.60) | -0.27 (-0.56,0.33) | 0.75 (0.26,1.46) | -0.1 (-0.41,0.3) |
| Burundi | -0.49 (-0.67,-0.2) | -0.42 (-0.6,-0.15) | -0.17 (-0.46,0.43) | -0.44 (-0.68,0.01) | -0.24 (-0.56,0.37) | -0.3 (-0.59,0.42) |
| Cabo Verde | 1.47 (0.64,2.76) | -0.33 (-0.54,0.01) | 1.43 (0.68,2.57) | 0.37 (-0.22,1.45) | 11.61 (8.1,17.33) | 1.47 (-0.02,3.2) |
| Cambodia | -0.25 (-0.52,0.18) | -0.33 (-0.56,0.00) | 0.41 (-0.12,1.46) | -0.17 (-0.52,0.44) | 0.55 (-0.07,1.66) | 0.14 (-0.42,1.09) |
| Cameroon | 0.93 (0.17,2.03) | -0.22 (-0.49,0.12) | 0.24 (-0.20,0.88) | -0.30 (-0.59,0.17) | 0.84 (0.19,1.66) | -0.05 (-0.38,0.36) |
| Canada | 0.64 (0.39,0.93) | -0.12 (-0.24,0.00) | 0.54 (0.29,0.84) | 1.03 (0.68,1.45) | -0.01 (-0.13,0.14) | 0.07 (-0.10,0.28) |
| Central African Republic | -0.19 (-0.49,0.32) | -0.19 (-0.46,0.19) | 0.09 (-0.31,0.67) | -0.36 (-0.65,0.08) | 0.04 (-0.33,0.66) | -0.05 (-0.4,0.49) |
| Chad | 1.02 (0.30,2.04) | -0.03 (-0.31,0.39) | 0.38 (-0.09,0.94) | -0.23 (-0.55,0.42) | 1.01 (0.37,1.99) | 0.08 (-0.28,0.53) |
| Chile | -0.45 (-0.52,-0.36) | -0.33 (-0.44,-0.2) | 1.27 (0.93,1.65) | 0.97 (0.56,1.55) | 0.80 (0.55,1.07) | -0.52 (-0.58,-0.45) |
| China | -0.25 (-0.46,0.02) | -0.17 (-0.35,0.1) | 1.26 (0.69,1.98) | 0.28 (-0.08,0.74) | 0.66 (0.19,1.26) | 0.92 (0.40,1.70) |
| Colombia | -0.44 (-0.54,-0.29) | -0.14 (-0.31,0.05) | 1.52 (0.98,2.13) | -0.25 (-0.41,-0.05) | 0.04 (-0.15,0.26) | -0.28 (-0.42,-0.12) |
| Comoros | -0.13 (-0.47,0.54) | -0.2 (-0.48,0.4) | 0.28 (-0.17,1.16) | 0.03 (-0.38,0.83) | 0.31 (-0.25,1.3) | 0.14 (-0.30,0.80) |
| Congo | -0.23 (-0.54,0.3) | -0.28 (-0.54,0.14) | 0.32 (-0.20,1.48) | -0.31 (-0.63,0.19) | 0.28 (-0.3,1.73) | 0.34 (-0.20,1.56) |
| Cook Islands | 0.29 (-0.22,1.07) | -0.15 (-0.45,0.3) | 0.39 (-0.11,1.24) | 0.54 (-0.09,1.72) | 0.59 (-0.02,1.47) | -0.18 (-0.65,1.29) |
| Costa Rica | 0.03 (-0.17,0.23) | -0.13 (-0.25,0.03) | 2.85 (2.06,3.86) | 0.27 (0.06,0.50) | 0.81 (0.51,1.13) | -0.11 (-0.26,0.08) |
| Croatia | -0.31 (-0.5,-0.07) | -0.58 (-0.67,-0.44) | 0.40 (0.09,0.82) | -0.37 (-0.58,-0.04) | -0.14 (-0.31,0.06) | -0.24 (-0.43,-0.01) |
| Cuba | 1.22 (0.7,1.81) | -0.17 (-0.31,0.01) | 1.01 (0.60,1.57) | -0.08 (-0.29,0.16) | 0.66 (0.34,1.07) | -0.35 (-0.47,-0.18) |
| Cyprus | 0.93 (0.29,1.85) | -0.25 (-0.43,0.00) | 0.55 (0.13,1.20) | 0.06 (-0.29,0.59) | 0.62 (0.06,1.52) | 0.06 (-0.23,0.55) |
| Czechia | -0.11 (-0.3,0.13) | -0.57 (-0.67,-0.44) | 0.06 (-0.20,0.42) | -0.58 (-0.7,-0.44) | -0.12 (-0.3,0.1) | -0.36 (-0.49,-0.2) |
| C么te d'Ivoire | 0.24 (-0.22,0.95) | -0.09 (-0.39,0.35) | 0.29 (-0.16,0.92) | -0.37 (-0.68,0.36) | 0.55 (-0.02,1.28) | 0.08 (-0.24,0.62) |
| Democratic People's Republic of Korea | 0.12 (-0.36,0.85) | 0.07 (-0.32,0.67) | 0.40 (-0.10,1.24) | -0.18 (-0.60,0.95) | 0.33 (-0.21,1.20) | 0.15 (-0.33,0.82) |
| Democratic Republic of the Congo | -0.17 (-0.47,0.29) | -0.16 (-0.43,0.22) | 0.14 (-0.28,0.76) | -0.22 (-0.53,0.23) | 0.06 (-0.42,0.81) | 0.05 (-0.34,0.74) |
| Denmark | -0.20 (-0.33,-0.04) | -0.54 (-0.62,-0.46) | -0.17 (-0.34,0.04) | -0.18 (-0.33,0.01) | -0.07 (-0.22,0.09) | -0.29 (-0.41,-0.14) |
| Djibouti | 0.01 (-0.40,0.69) | 0.02 (-0.37,0.65) | 0.68 (0.07,1.76) | 0.41 (-0.22,1.32) | 0.62 (-0.01,1.5) | 0.36 (-0.14,1.23) |
| Dominica | 0.56 (0.05,1.35) | 0.02 (-0.24,0.38) | 1.01 (0.45,1.76) | 0.57 (-0.14,1.65) | 1.07 (0.44,2.04) | 0.21 (-0.18,0.66) |
| Dominican Republic | 0.70 (0.09,1.60) | 0.11 (-0.18,0.52) | 1.16 (0.55,1.94) | 0.81 (0.08,1.81) | 1.32 (0.64,2.33) | -0.01 (-0.33,0.67) |
| Ecuador | -0.30 (-0.48,-0.07) | -0.09 (-0.32,0.16) | 1.46 (0.82,2.24) | -0.39 (-0.54,-0.17) | 0.72 (0.26,1.25) | -0.24 (-0.44,-0.02) |
| Egypt | -0.36 (-0.56,-0.07) | 0.23 (-0.35,0.69) | 0.86 (0.43,1.38) | 0.13 (-0.33,0.84) | 0.91 (0.39,1.54) | -0.01 (-0.26,0.30) |
| El Salvador | 0.13 (-0.15,0.47) | 0.13 (-0.14,0.52) | 1.73 (1.02,2.71) | 0.19 (-0.11,0.61) | 0.91 (0.45,1.43) | 0.06 (-0.21,0.53) |
| Equatorial Guinea | -0.52 (-0.72,-0.15) | -0.66 (-0.79,-0.42) | 0.28 (-0.28,1.41) | 0.24 (-0.37,1.36) | 0.48 (-0.23,2.00) | -0.12 (-0.59,0.76) |
| Eritrea | -0.32 (-0.57,0.09) | -0.26 (-0.51,0.10) | 0.24 (-0.24,1.02) | -0.04 (-0.44,0.59) | 0.14 (-0.34,1) | 0.06 (-0.34,0.86) |
| Estonia | -0.39 (-0.52,-0.22) | -0.64 (-0.71,-0.58) | 0.13 (-0.17,0.53) | -0.16 (-0.36,0.08) | 0.03 (-0.19,0.28) | -0.42 (-0.53,-0.29) |
| Eswatini | 0.46 (-0.15,1.48) | 0.25 (-0.24,0.94) | 1.02 (0.21,2.27) | 2.36 (-0.31,15.06) | 1.26 (0.27,2.9) | 0.64 (-0.03,1.54) |
| Ethiopia | -0.56 (-0.70,-0.28) | -0.65 (-0.75,-0.53) | -0.31 (-0.53,0.4) | -0.41 (-0.63,0.05) | -0.12 (-0.49,0.65) | -0.44 (-0.66,0.35) |
| Fiji | 0.27 (-0.17,0.91) | -0.19 (-0.46,0.16) | 0.20 (-0.20,0.78) | 0.26 (-0.32,1.41) | 0.52 (0.04,1.17) | 0.14 (-0.27,0.76) |
| Finland | -0.23 (-0.33,-0.12) | -0.68 (-0.73,-0.62) | -0.15 (-0.30,0.04) | -0.09 (-0.25,0.11) | -0.25 (-0.36,-0.13) | -0.33 (-0.43,-0.19) |
| France | -0.51 (-0.59,-0.4) | -0.23 (-0.37,-0.05) | 0.48 (0.18,0.84) | 0.66 (0.36,1.03) | 0.48 (0.27,0.7) | -0.19 (-0.31,-0.04) |
| Gabon | -0.09 (-0.46,0.52) | -0.29 (-0.53,0.11) | 0.28 (-0.23,1.23) | 0.03 (-0.48,0.99) | 0.61 (-0.07,1.73) | 0.15 (-0.3,0.99) |
| Gambia | 0.39 (-0.15,1.18) | -0.12 (-0.41,0.30) | 0.34 (-0.13,1.05) | 0.18 (-0.35,1.22) | 0.63 (0.01,1.6) | 0.17 (-0.21,0.71) |
| Georgia | -0.47 (-0.57,-0.37) | -0.46 (-0.55,-0.35) | 0.02 (-0.17,0.23) | -0.03 (-0.25,0.25) | 3.56 (2.54,4.8) | -0.38 (-0.58,-0.09) |
| Germany | -0.26 (-0.39,-0.09) | -0.36 (-0.44,-0.28) | 0.05 (-0.15,0.27) | 0.89 (0.44,1.42) | 0.04 (-0.09,0.21) | -0.22 (-0.34,-0.1) |
| Ghana | 0.37 (-0.10,1.03) | -0.30 (-0.52,0.03) | 0.31 (-0.16,1.13) | -0.34 (-0.67,0.41) | 1.66 (0.87,3.03) | 0.06 (-0.3,0.58) |
| Greece | 0.62 (0.43,0.87) | -0.31 (-0.38,-0.24) | 0.45 (0.25,0.70) | 1.57 (1.29,1.89) | 0.61 (0.45,0.78) | 0.18 (0.03,0.35) |
| Greenland | -0.42 (-0.64,-0.07) | -0.54 (-0.68,-0.36) | -0.23 (-0.47,0.07) | -0.27 (-0.61,0.34) | -0.21 (-0.45,0.11) | -0.57 (-0.74,0.00) |
| Grenada | -0.04 (-0.29,0.26) | -0.37 (-0.51,-0.19) | 0.79 (0.34,1.40) | 0.75 (0.25,1.48) | 0.68 (0.33,1.19) | -0.12 (-0.32,0.21) |
| Guam | 1.20 (0.72,1.81) | 0.57 (0.28,0.92) | 1.00 (0.64,1.48) | 2.18 (1.45,3.02) | 2.12 (1.5,2.77) | 0.65 (0.26,1.11) |
| Guatemala | -0.4 (-0.49,-0.28) | -0.07 (-0.2,0.09) | 1.07 (0.72,1.51) | -0.26 (-0.37,-0.13) | 0.26 (0.07,0.51) | -0.57 (-0.65,-0.49) |
| Guinea | -0.01 (-0.41,0.65) | -0.21 (-0.45,0.18) | 0.10 (-0.30,0.79) | -0.28 (-0.59,0.27) | 0.58 (0,1.36) | -0.05 (-0.38,0.43) |
| Guinea-Bissau | 0.59 (-0.01,1.56) | -0.22 (-0.47,0.17) | 0.19 (-0.23,0.9) | -0.4 (-0.66,0.39) | 0.69 (0.1,1.66) | -0.03 (-0.37,0.56) |
| Guyana | 0.73 (0.18,1.44) | -0.02 (-0.31,0.37) | 1.31 (0.60,2.22) | 0.39 (-0.05,0.94) | 1.26 (0.59,2.05) | 0.03 (-0.28,0.38) |
| Haiti | -0.19 (-0.47,0.25) | -0.33 (-0.54,-0.02) | 0.09 (-0.31,0.81) | -0.18 (-0.52,0.44) | 0.04 (-0.36,0.75) | -0.23 (-0.49,0.22) |
| Honduras | -0.14 (-0.48,0.33) | -0.31 (-0.56,0.03) | 0.09 (-0.32,0.68) | 0.14 (-0.33,0.94) | 0.31 (-0.2,1.1) | 0.04 (-0.35,0.58) |
| Hungary | -0.62 (-0.69,-0.51) | -0.60 (-0.68,-0.51) | 0.28 (-0.03,0.65) | -0.58 (-0.71,-0.4) | -0.19 (-0.36,0.00) | -0.46 (-0.57,-0.34) |
| Iceland | 0.70 (0.39,1.07) | -0.51 (-0.59,-0.42) | 0.28 (0.03,0.60) | 0.55 (0.22,0.94) | 0.50 (0.27,0.79) | 0.14 (-0.08,0.40) |
| India | -0.10 (-0.23,0.08) | -0.30 (-0.40,-0.17) | 0.27 (0.03,0.65) | 0.43 (0.19,0.69) | 0.44 (0.07,0.88) | 0.39 (0.02,0.74) |
| Indonesia | -0.06 (-0.29,0.34) | -0.21 (-0.38,0.07) | 0.59 (0.14,1.32) | 0.41 (0.02,0.97) | 0.93 (0.40,1.60) | 0.12 (-0.22,0.77) |
| Iran (Islamic Republic of) | 0.07 (-0.11,0.38) | -0.14 (-0.27,0.02) | 1.25 (0.78,2.15) | 0.78 (0.4,1.15) | 1.68 (1.10,2.42) | 1.58 (0.45,2.66) |
| Iraq | 0.04 (-0.31,0.63) | -0.23 (-0.47,0.16) | 0.67 (0.11,1.67) | 0.13 (-0.29,0.86) | 0.58 (-0.04,1.72) | 0.06 (-0.29,0.67) |
| Ireland | 0.19 (-0.02,0.47) | -0.45 (-0.52,-0.37) | 0.22 (-0.01,0.49) | 1.13 (0.74,1.57) | 0.09 (-0.07,0.26) | 0.02 (-0.14,0.23) |
| Israel | 0.11 (-0.08,0.34) | -0.45 (-0.55,-0.34) | 0.24 (0.03,0.49) | 0.76 (0.34,1.27) | 0.11 (-0.03,0.28) | -0.2 (-0.33,-0.06) |
| Italy | -0.38 (-0.43,-0.33) | -0.45 (-0.48,-0.4) | 0.21 (0.09,0.35) | 0.19 (0.05,0.33) | 0.09 (0.02,0.15) | -0.09 (-0.17,0.02) |
| Jamaica | 0.78 (0.22,1.57) | 0.14 (-0.19,0.57) | 1.94 (1.01,3.20) | 1.28 (0.50,2.26) | 0.87 (0.16,1.9) | 0.15 (-0.28,0.80) |
| Japan | -0.09 (-0.16,-0.02) | -0.70 (-0.71,-0.68) | 0.16 (0.07,0.26) | -0.51 (-0.55,-0.47) | 0.08 (0.05,0.12) | -0.47 (-0.51,-0.43) |
| Jordan | 0.06 (-0.32,0.63) | -0.35 (-0.54,-0.10) | 0.51 (-0.01,1.36) | -0.09 (-0.45,0.59) | 0.76 (0.13,1.72) | 0.01 (-0.37,0.58) |
| Kazakhstan | -0.69 (-0.73,-0.65) | -0.63 (-0.68,-0.58) | -0.14 (-0.26,-0.01) | -0.52 (-0.61,-0.40) | 0.29 (-0.22,0.86) | 0.17 (-0.19,0.77) |
| Kenya | 0.48 (0.12,1.02) | 0.11 (-0.15,0.49) | 0.66 (0.28,1.26) | 0.87 (0.41,1.48) | 1.30 (0.75,2.21) | 0.39 (0.04,0.88) |
| Kiribati | 0.13 (-0.29,0.80) | 0.04 (-0.29,0.60) | 0.20 (-0.20,0.78) | 0.01 (-0.44,0.84) | 0.39 (-0.16,1.29) | 0.15 (-0.26,0.93) |
| Kuwait | -0.16 (-0.36,0.08) | -0.12 (-0.31,0.14) | 2.71 (1.81,4.04) | -0.73 (-0.80,-0.64) | 1.26 (0.79,2.00) | 0.45 (0.12,0.81) |
| Kyrgyzstan | -0.69 (-0.78,-0.57) | -0.56 (-0.66,-0.43) | -0.09 (-0.32,0.22) | -0.67 (-0.80,-0.46) | 0.97 (0.46,1.75) | 0.07 (-0.22,0.45) |
| Lao People's Democratic Republic | -0.44 (-0.66,-0.06) | -0.53 (-0.69,-0.28) | 0.13 (-0.29,0.99) | -0.32 (-0.61,0.18) | 0.27 (-0.29,1.20) | -0.20 (-0.57,0.60) |
| Latvia | -0.22 (-0.39,0.01) | -0.55 (-0.64,-0.45) | 0.09 (-0.17,0.40) | 0.00 (-0.29,0.38) | 0.49 (0.16,0.86) | -0.07 (-0.24,0.16) |
| Lebanon | -0.28 (-0.5,0.11) | -0.51 (-0.65,-0.33) | 0.34 (-0.13,1.63) | -0.23 (-0.51,0.23) | 0.15 (-0.36,1.41) | -0.15 (-0.48,0.71) |
| Lesotho | 0.90 (0.20,2.07) | 0.75 (0.07,1.85) | 1.60 (0.53,3.30) | 2.7 (-0.04,17.26) | 1.77 (0.47,3.66) | 1.01 (0.09,2.47) |
| Liberia | 1.54 (0.60,3.00) | 0.08 (-0.29,0.65) | 0.55 (-0.11,1.50) | -0.01 (-0.43,0.7) | 1.07 (0.16,2.30) | 0.07 (-0.32,0.77) |
| Libya | 1.14 (0.20,2.86) | 0.35 (-0.12,1.19) | 1.21 (0.42,2.54) | 1.16 (0.14,2.83) | 1.45 (0.38,3.21) | 0.82 (0.18,1.76) |
| Lithuania | 0.06 (-0.18,0.34) | -0.50 (-0.59,-0.39) | 0.15 (-0.11,0.49) | 0.40 (0.07,0.81) | 0.34 (0.09,0.61) | -0.01 (-0.20,0.17) |
| Luxembourg | -0.42 (-0.51,-0.30) | -0.73 (-0.77,-0.70) | -0.27 (-0.38,-0.14) | 0.09 (-0.07,0.27) | -0.31 (-0.40,-0.19) | -0.43 (-0.52,-0.34) |
| Madagascar | -0.23 (-0.50,0.13) | -0.23 (-0.45,0.11) | 0.06 (-0.24,0.53) | -0.23 (-0.52,0.23) | 0.01 (-0.37,0.62) | 0.00 (-0.33,0.49) |
| Malawi | 0.18 (-0.23,0.79) | -0.19 (-0.42,0.09) | 0.22 (-0.16,0.75) | 0.42 (-0.2,1.55) | 0.36 (-0.12,1.12) | -0.06 (-0.4,0.53) |
| Malaysia | 0.45 (0.07,0.96) | -0.01 (-0.20,0.22) | 0.77 (0.39,1.26) | 0.69 (0.05,1.55) | 0.95 (0.42,1.65) | 0.25 (-0.11,0.71) |
| Maldives | -0.53 (-0.7,-0.12) | -0.61 (-0.72,-0.42) | 0.09 (-0.32,2.12) | -0.26 (-0.58,0.51) | 0.16 (-0.37,1.99) | -0.33 (-0.66,1.28) |
| Mali | -0.15 (-0.44,0.27) | -0.38 (-0.55,-0.14) | -0.09 (-0.38,0.29) | -0.10 (-0.4,0.38) | 0.11 (-0.28,0.64) | -0.19 (-0.47,0.22) |
| Malta | 0.49 (0.20,0.83) | -0.42 (-0.51,-0.31) | 0.47 (0.18,0.82) | 1.16 (0.75,1.71) | 0.71 (0.43,1.07) | 0.12 (-0.07,0.35) |
| Marshall Islands | 0.50 (-0.07,1.34) | 0.15 (-0.17,0.64) | 0.64 (0.12,1.29) | 0.74 (-0.02,1.88) | 1.45 (0.67,2.63) | 0.38 (-0.10,0.99) |
| Mauritania | 0.39 (-0.10,1.08) | -0.36 (-0.55,-0.09) | 0.23 (-0.19,1.07) | -0.55 (-0.73,0.36) | 0.67 (0.06,1.82) | -0.18 (-0.47,0.35) |
| Mauritius | 0.81 (0.57,1.03) | 0.17 (0.01,0.34) | 2.25 (1.79,2.71) | -0.77 (-0.79,-0.74) | 0.88 (0.66,1.11) | -0.23 (-0.32,-0.14) |
| Mexico | 0.10 (-0.04,0.26) | 0.17 (0.03,0.31) | 2.24 (1.85,2.65) | 0.77 (0.56,0.99) | 0.36 (0.20,0.54) | -0.29 (-0.39,-0.19) |
| Micronesia (Federated States of) | 0.08 (-0.37,0.90) | -0.07 (-0.37,0.43) | 0.35 (-0.12,1.10) | 0.24 (-0.33,1.26) | 0.87 (0.20,1.93) | 0.01 (-0.38,0.67) |
| Monaco | 0.43 (-0.11,1.26) | -0.31 (-0.59,0.10) | 0.50 (-0.06,1.29) | 1.45 (0.47,3.19) | 0.14 (-0.32,0.82) | 0.18 (-0.32,0.90) |
| Mongolia | -0.05 (-0.38,0.42) | 0.06 (-0.27,0.46) | 0.88 (0.36,1.57) | 0.43 (-0.23,1.47) | 10.08 (6.64,15.11) | -0.13 (-0.51,0.96) |
| Montenegro | 0.17 (-0.17,0.69) | -0.2 (-0.38,0.02) | 0.29 (-0.05,0.73) | 0.13 (-0.22,0.64) | 0.24 (-0.13,0.84) | -0.05 (-0.29,0.25) |
| Morocco | 0.17 (-0.24,0.85) | -0.18 (-0.45,0.23) | 1.10 (0.40,2.42) | 0.50 (-0.11,1.64) | 0.86 (0.23,1.94) | 0.32 (-0.13,1.19) |
| Mozambique | 0.32 (-0.25,1.09) | -0.02 (-0.36,0.47) | 0.36 (-0.11,1.03) | 0.29 (-0.5,3.16) | 0.57 (-0.02,1.53) | 0.12 (-0.28,0.72) |
| Myanmar | -0.38 (-0.61,0.03) | -0.51 (-0.68,-0.26) | 0.23 (-0.25,1.03) | 0.05 (-0.42,1.09) | 0.45 (-0.18,1.6) | -0.16 (-0.5,0.52) |
| Namibia | 0.34 (-0.23,1.4) | -0.03 (-0.39,0.50) | 0.51 (-0.08,1.31) | 0.92 (-0.05,2.82) | 0.79 (0.09,1.85) | 0.28 (-0.24,1.05) |
| Nauru | -0.06 (-0.42,0.45) | -0.13 (-0.38,0.30) | 0.06 (-0.30,0.59) | -0.08 (-0.5,0.72) | 0.33 (-0.11,1.02) | -0.14 (-0.45,0.35) |
| Nepal | -0.23 (-0.5,0.19) | -0.39 (-0.59,-0.12) | 0.16 (-0.25,0.95) | 0.71 (0.03,1.79) | 0.44 (-0.08,1.3) | 0.23 (-0.22,1.00) |
| Netherlands | -0.04 (-0.2,0.15) | -0.62 (-0.67,-0.58) | 0.12 (-0.08,0.36) | 0.69 (0.38,1.06) | -0.06 (-0.18,0.06) | -0.06 (-0.2,0.12) |
| New Zealand | 0.04 (-0.11,0.21) | -0.33 (-0.43,-0.21) | 0.06 (-0.12,0.27) | 1.35 (0.94,1.89) | 0.18 (0.03,0.35) | 1.02 (0.75,1.35) |
| Nicaragua | -0.02 (-0.29,0.38) | -0.13 (-0.32,0.11) | 0.95 (0.42,1.60) | 0.16 (-0.24,0.77) | 0.62 (0.2,1.15) | -0.21 (-0.45,0.26) |
| Niger | 0.12 (-0.3,0.72) | -0.36 (-0.55,-0.09) | -0.09 (-0.38,0.36) | -0.61 (-0.76,-0.3) | 0.18 (-0.24,0.85) | -0.27 (-0.51,0.05) |
| Nigeria | 0.48 (0.00,1.13) | -0.35 (-0.57,0.02) | 0.11 (-0.27,0.77) | -0.28 (-0.53,0.43) | 0.53 (0.00,1.37) | 0.03 (-0.37,0.62) |
| Niue | 0.15 (-0.31,0.94) | -0.07 (-0.39,0.45) | 0.46 (-0.05,1.30) | 0.27 (-0.34,1.39) | 0.93 (0.18,2.14) | 0.00 (-0.37,0.56) |
| North Macedonia | -0.05 (-0.35,0.37) | -0.44 (-0.57,-0.26) | 0.30 (-0.04,0.75) | -0.26 (-0.51,0.08) | 0.13 (-0.18,0.6) | -0.23 (-0.43,0.11) |
| Northern Mariana Islands | 1.23 (0.50,2.45) | -0.07 (-0.37,0.40) | 0.34 (-0.07,1.03) | 0.25 (-0.37,1.33) | 1.65 (0.75,3.07) | 0.29 (-0.17,1.01) |
| Norway | 0.15 (0.06,0.25) | -0.63 (-0.65,-0.59) | 0.02 (-0.09,0.15) | 1.62 (1.38,1.91) | 0 (-0.06,0.07) | 0.37 (0.19,0.57) |
| Oman | -0.07 (-0.39,0.48) | -0.5 (-0.66,-0.21) | 0.39 (-0.19,1.34) | 0.47 (-0.18,1.77) | 0.66 (0.01,1.59) | -0.02 (-0.43,0.57) |
| Pakistan | 0.1 (-0.23,0.52) | -0.01 (-0.27,0.36) | 0.48 (0.10,1.00) | 0.36 (-0.02,0.98) | 0.7 (0.21,1.43) | 0.24 (-0.11,0.74) |
| Palau | 0.78 (0.15,1.81) | 0.31 (-0.08,0.88) | 0.41 (-0.04,1.18) | 1.06 (0.07,2.98) | 0.61 (0.03,1.56) | 0.57 (0.04,1.33) |
| Palestine | -0.3 (-0.56,0.05) | -0.40 (-0.58,-0.13) | 0.2 (-0.19,0.94) | -0.14 (-0.43,0.28) | 0.29 (-0.23,1.15) | -0.06 (-0.38,0.48) |
| Panama | -0.11 (-0.31,0.12) | 0.03 (-0.21,0.27) | 1.93 (1.23,2.65) | 0.22 (-0.03,0.49) | 0.65 (0.28,1.04) | -0.29 (-0.44,-0.1) |
| Papua New Guinea | -0.12 (-0.49,0.55) | -0.17 (-0.46,0.26) | 0.04 (-0.32,0.71) | -0.25 (-0.6,0.45) | 0.44 (-0.11,1.5) | -0.16 (-0.5,0.47) |
| Paraguay | 0.38 (-0.15,1.19) | -0.14 (-0.37,0.21) | 1.04 (0.42,1.91) | 0.66 (0.01,1.52) | 1.02 (0.34,1.89) | 0.02 (-0.31,0.46) |
| Peru | -0.17 (-0.44,0.21) | -0.13 (-0.38,0.23) | 0.83 (0.32,1.53) | 0.14 (-0.29,0.68) | 0.19 (-0.19,0.75) | 0.07 (-0.32,0.53) |
| Philippines | -0.05 (-0.23,0.17) | -0.22 (-0.37,-0.04) | 0.39 (0.14,0.67) | -0.25 (-0.45,0.21) | 0.42 (0.13,0.78) | 0.13 (-0.08,0.44) |
| Poland | -0.21 (-0.3,-0.13) | -0.50 (-0.55,-0.45) | 0.40 (0.24,0.57) | 2.43 (1.91,2.99) | -0.20 (-0.28,-0.12) | -0.21 (-0.28,-0.13) |
| Portugal | 0.00 (-0.17,0.22) | -0.49 (-0.56,-0.40) | 0.86 (0.54,1.25) | 1.40 (0.85,2.18) | 0.25 (0.06,0.43) | 0.18 (-0.01,0.42) |
| Puerto Rico | -0.37 (-0.53,-0.18) | -0.47 (-0.60,-0.32) | 1.13 (0.67,1.74) | 0.35 (0.02,0.82) | 1.93 (1.27,2.71) | -0.23 (-0.4,-0.01) |
| Qatar | -0.26 (-0.53,0.16) | -0.42 (-0.61,-0.19) | 0.62 (0.14,1.25) | 0.24 (-0.27,0.96) | 0.25 (-0.14,0.8) | 0.07 (-0.29,0.54) |
| Republic of Korea | -0.34 (-0.55,-0.03) | -0.52 (-0.62,-0.26) | 1.18 (0.68,2.00) | -0.24 (-0.52,0.24) | 0.02 (-0.21,0.34) | -0.16 (-0.45,0.74) |
| Republic of Moldova | -0.25 (-0.37,-0.12) | -0.54 (-0.60,-0.46) | 0.24 (0.03,0.52) | 0.22 (0.05,0.40) | 0.32 (0.14,0.51) | -0.21 (-0.33,-0.07) |
| Romania | 0.75 (0.36,1.25) | -0.20 (-0.38,0.02) | 1.12 (0.68,1.68) | 0.95 (0.46,1.57) | 0.54 (0.26,0.89) | -0.23 (-0.36,-0.08) |
| Russian Federation | 0.02 (-0.09,0.11) | -0.49 (-0.54,-0.45) | 0.48 (0.34,0.62) | 0.88 (0.71,1.05) | 0.06 (-0.03,0.14) | 0.52 (0.38,0.65) |
| Rwanda | -0.51 (-0.71,-0.16) | -0.54 (-0.7,-0.31) | -0.15 (-0.44,0.42) | -0.39 (-0.64,0.02) | -0.18 (-0.54,0.62) | -0.28 (-0.58,0.46) |
| Saint Kitts and Nevis | 0.09 (-0.18,0.45) | -0.48 (-0.6,-0.32) | 0.45 (0.11,0.92) | -0.2 (-0.42,0.08) | 0.29 (-0.07,1.02) | -0.39 (-0.56,-0.09) |
| Saint Lucia | 0.45 (0.15,0.86) | -0.18 (-0.34,0.02) | 1.03 (0.62,1.54) | 0.18 (-0.1,0.53) | 0.99 (0.59,1.51) | -0.10 (-0.28,0.17) |
| Saint Vincent and the Grenadines | 1.08 (0.71,1.50) | 0.00 (-0.18,0.22) | 1.42 (0.94,2.03) | 0.38 (0.11,0.67) | 1.00 (0.65,1.43) | -0.01 (-0.16,0.19) |
| Samoa | 0.37 (-0.15,1.11) | 0.27 (-0.20,0.96) | 0.64 (0.04,1.60) | 0.28 (-0.4,1.54) | 0.64 (0.03,1.64) | 0.34 (-0.19,1.17) |
| San Marino | -0.15 (-0.61,0.6) | -0.47 (-0.74,-0.14) | -0.08 (-0.52,0.48) | 0.84 (-0.12,2.43) | -0.01 (-0.49,0.66) | -0.14 (-0.57,0.43) |
| Sao Tome and Principe | 1.42 (0.5,2.96) | -0.01 (-0.38,0.50) | 0.70 (0.07,1.67) | 0.00 (-0.53,1.29) | 0.97 (0.16,2.32) | 0.43 (-0.12,1.26) |
| Saudi Arabia | 0.50 (-0.1,1.48) | 0.07 (-0.33,0.72) | 2.24 (1.05,4.41) | -0.2 (-0.62,0.59) | 2.05 (0.94,3.9) | 0.96 (0.26,2.15) |
| Senegal | 0.68 (0.14,1.55) | -0.23 (-0.45,0.09) | 0.29 (-0.08,0.87) | -0.37 (-0.6,0.15) | 0.88 (0.23,1.84) | -0.07 (-0.36,0.42) |
| Serbia | -0.11 (-0.46,0.51) | -0.42 (-0.58,-0.23) | 0.17 (-0.18,0.68) | -0.12 (-0.43,0.36) | 0.01 (-0.31,0.4) | -0.22 (-0.5,0.16) |
| Seychelles | 0.45 (0.03,1.01) | -0.13 (-0.3,0.08) | 1.09 (0.6,1.61) | -0.26 (-0.51,0.04) | 0.59 (0.21,1.09) | -0.10 (-0.37,0.50) |
| Sierra Leone | 0.83 (0.14,2.05) | -0.06 (-0.34,0.34) | 0.42 (-0.03,1.15) | -0.48 (-0.72,0.5) | 0.82 (0.22,1.70) | 0.02 (-0.30,0.51) |
| Singapore | -0.06 (-0.24,0.14) | -0.53 (-0.6,-0.46) | 0.29 (0.09,0.55) | -0.33 (-0.44,-0.2) | 0.09 (-0.07,0.28) | -0.37 (-0.47,-0.26) |
| Slovakia | -0.28 (-0.59,0.21) | -0.48 (-0.62,-0.33) | 0.2 (-0.14,0.61) | -0.31 (-0.61,0.17) | -0.01 (-0.32,0.45) | -0.06 (-0.39,0.48) |
| Slovenia | -0.27 (-0.42,-0.08) | -0.6 (-0.69,-0.49) | 0.16 (-0.16,0.61) | -0.50 (-0.65,-0.30) | -0.07 (-0.27,0.15) | -0.41 (-0.56,-0.22) |
| Solomon Islands | 0.29 (-0.27,1.57) | 0.12 (-0.27,0.96) | 0.55 (0.02,1.75) | 0.11 (-0.43,2.34) | 1.17 (0.36,3.22) | 0.33 (-0.20,1.98) |
| Somalia | -0.38 (-0.60,0.00) | -0.24 (-0.51,0.17) | -0.10 (-0.44,0.43) | -0.13 (-0.56,0.63) | -0.26 (-0.54,0.22) | -0.12 (-0.43,0.45) |
| South Africa | -0.35 (-0.46,-0.20) | -0.36 (-0.44,-0.24) | 0.31 (0.12,0.55) | 0.25 (-0.22,1.16) | 0.23 (0.03,0.47) | 0.08 (-0.09,0.31) |
| South Sudan | 0.10 (-0.27,0.66) | 0.16 (-0.26,0.73) | 0.53 (-0.03,1.56) | 0.36 (-0.19,1.28) | 0.37 (-0.2,1.31) | 0.37 (-0.12,1.15) |
| Spain | -0.37 (-0.48,-0.23) | -0.42 (-0.51,-0.32) | 0.22 (0.01,0.5) | 0.86 (0.49,1.28) | 0.21 (0.04,0.4) | 0.01 (-0.16,0.19) |
| Sri Lanka | -0.13 (-0.49,0.34) | -0.45 (-0.67,-0.21) | 0.55 (-0.07,1.42) | -0.12 (-0.48,0.41) | 0.22 (-0.33,0.96) | -0.53 (-0.77,0.5) |
| Sudan | -0.21 (-0.53,0.3) | -0.34 (-0.6,0) | 0.42 (-0.15,1.55) | -0.02 (-0.49,0.7) | 0.78 (0.06,2.04) | 0.13 (-0.50,1.34) |
| Suriname | 0.43 (-0.09,1.17) | -0.07 (-0.3,0.29) | 0.85 (0.33,1.61) | 0.6 (-0.06,1.82) | 0.8 (0.19,1.73) | 0.05 (-0.28,0.57) |
| Sweden | -0.16 (-0.3,0.01) | -0.64 (-0.7,-0.57) | -0.09 (-0.24,0.09) | 0.02 (-0.16,0.24) | -0.38 (-0.47,-0.28) | -0.56 (-0.63,-0.49) |
| Switzerland | -0.39 (-0.5,-0.26) | -0.59 (-0.64,-0.52) | -0.19 (-0.30,-0.04) | -0.37 (-0.51,-0.2) | -0.15 (-0.28,-0.01) | -0.31 (-0.44,-0.16) |
| Syrian Arab Republic | 0.26 (-0.18,0.98) | -0.12 (-0.41,0.33) | 0.62 (0.01,1.71) | -0.14 (-0.49,0.42) | 0.99 (0.18,2.31) | 0.55 (-0.05,1.74) |
| Taiwan (Province of China) | 1.72 (1.32,2.14) | -0.28 (-0.38,-0.15) | 1.42 (1.13,1.81) | 0.23 (0.01,0.45) | 1.19 (0.88,1.57) | 0.43 (0.21,0.69) |
| Tajikistan | -0.43 (-0.63,-0.14) | -0.42 (-0.6,-0.18) | -0.28 (-0.52,0.14) | -0.21 (-0.61,0.47) | 0.09 (-0.43,1.09) | -0.19 (-0.58,0.58) |
| Thailand | 1.85 (0.75,3.56) | 0.64 (0.14,1.37) | 2.42 (1.49,3.84) | 0.45 (-0.09,1.31) | 1.73 (0.86,2.93) | 1.30 (0.57,2.34) |
| Timor-Leste | -0.25 (-0.57,0.21) | -0.37 (-0.57,-0.06) | 0.16 (-0.26,0.91) | -0.2 (-0.57,0.42) | 0.27 (-0.25,1.24) | -0.15 (-0.48,0.41) |
| Togo | 1.52 (0.64,3.08) | 0.05 (-0.29,0.55) | 0.59 (0.07,1.37) | -0.03 (-0.41,0.58) | 1.37 (0.53,2.72) | 0.14 (-0.29,0.76) |
| Tokelau | 0.26 (-0.25,1.18) | -0.05 (-0.34,0.44) | 0.59 (0.08,1.36) | 0.70 (0.03,1.95) | 1.19 (0.38,2.65) | -0.04 (-0.37,0.56) |
| Tonga | 0.29 (-0.20,1.10) | 0.07 (-0.30,0.63) | 0.37 (-0.13,1.06) | 0.29 (-0.28,1.26) | 0.76 (0.16,1.75) | -0.04 (-0.4,0.46) |
| Trinidad and Tobago | 0.52 (0.10,1.02) | -0.05 (-0.3,0.25) | 1.26 (0.65,1.99) | 0.39 (0.03,0.81) | 0.67 (0.19,1.22) | -0.15 (-0.41,0.16) |
| Tunisia | 0.67 (0.01,1.63) | 0.08 (-0.27,0.63) | 1.39 (0.56,2.53) | 0.84 (0.00,2.13) | 1.39 (0.50,2.89) | 0.64 (0.08,1.45) |
| Turkey | -0.30 (-0.52,0.02) | -0.51 (-0.64,-0.35) | 0.31 (-0.08,0.91) | 0.10 (-0.3,0.65) | 0.25 (-0.19,0.9) | -0.19 (-0.45,0.32) |
| Turkmenistan | -0.52 (-0.64,-0.35) | -0.29 (-0.47,-0.04) | 0.29 (-0.03,0.71) | 0.39 (-0.05,0.96) | 23.19 (16.26,32.27) | -0.56 (-0.69,-0.40) |
| Tuvalu | -0.06 (-0.36,0.4) | -0.28 (-0.47,0.00) | 0.12 (-0.21,0.61) | 0.15 (-0.29,0.91) | 0.64 (0.12,1.43) | -0.37 (-0.59,0.00) |
| Uganda | 0.16 (-0.26,0.81) | -0.16 (-0.45,0.27) | 0.37 (-0.13,1.17) | 0.11 (-0.35,0.9) | 0.78 (0.09,2.02) | 0.22 (-0.24,1.00) |
| Ukraine | -0.10 (-0.42,0.34) | -0.55 (-0.69,-0.39) | -0.15 (-0.42,0.17) | 0.06 (-0.3,0.53) | 0.67 (-0.08,1.7) | 0.26 (-0.23,1.14) |
| United Arab Emirates | 0.05 (-0.28,0.55) | -0.42 (-0.58,-0.23) | 0.01 (-0.36,1.01) | 0.76 (0.10,1.74) | 0.48 (-0.14,1.38) | -0.03 (-0.33,0.54) |
| United Kingdom | 0.15 (0.11,0.19) | -0.40 (-0.42,-0.38) | 0.28 (0.23,0.33) | 2.67 (2.54,2.81) | 0.06 (0.02,0.1) | 0.79 (0.70,0.88) |
| United Republic of Tanzania | -0.26 (-0.52,0.15) | -0.29 (-0.53,0.01) | 0.22 (-0.20,0.76) | -0.15 (-0.49,0.42) | 0.2 (-0.25,0.74) | 0.07 (-0.30,0.58) |
| United States of America | 0.00 (-0.04,0.04) | -0.07 (-0.11,-0.03) | 0.44 (0.37,0.50) | 0.80 (0.72,0.88) | 0.07 (0.04,0.11) | 0.20 (0.15,0.27) |
| United States Virgin Islands | 0.20 (-0.26,0.83) | -0.25 (-0.47,0.09) | 0.43 (-0.01,1.01) | 0.55 (-0.17,1.93) | 0.9 (0.21,1.89) | -0.27 (-0.54,0.18) |
| Uruguay | -0.24 (-0.37,-0.10) | -0.18 (-0.28,-0.05) | 0.51 (0.23,0.86) | 1.46 (0.70,2.48) | 0.3 (0.11,0.53) | -0.21 (-0.31,-0.07) |
| Uzbekistan | -0.53 (-0.65,-0.38) | -0.45 (-0.55,-0.33) | 0.08 (-0.14,0.35) | 0.55 (-0.01,1.45) | 1.35 (0.59,2.42) | 1.43 (0.59,2.64) |
| Vanuatu | 0.11 (-0.39,0.90) | -0.03 (-0.35,0.50) | 0.28 (-0.18,0.96) | 0.03 (-0.45,1.14) | 0.81 (0.14,1.83) | 0.11 (-0.34,0.73) |
| Venezuela (Bolivarian Republic of) | 0.24 (-0.13,0.69) | 0.18 (-0.13,0.57) | 1.42 (0.75,2.27) | 0.06 (-0.22,0.37) | 2.35 (1.25,3.53) | -0.11 (-0.39,0.24) |
| Viet Nam | 1.30 (0.40,2.94) | 0.03 (-0.28,0.53) | 1.82 (0.85,3.44) | 0.32 (-0.2,1.31) | 1.72 (0.69,3.28) | 1.02 (0.15,2.46) |
| Yemen | -0.26 (-0.54,0.24) | -0.28 (-0.52,0.14) | 0.28 (-0.19,1.27) | -0.29 (-0.58,0.37) | 0.45 (-0.14,1.72) | 0.26 (-0.31,1.59) |
| Zambia | -0.07 (-0.46,0.64) | -0.21 (-0.48,0.29) | 0.66 (-0.11,3.12) | -0.53 (-0.83,0.10) | 0.32 (-0.22,1.1) | 0.09 (-0.3,0.63) |
| Zimbabwe | 1.25 (0.47,2.60) | 0.96 (0.24,1.91) | 1.31 (0.53,2.39) | 0.78 (-0.03,2.39) | 1.73 (0.81,3.27) | 0.99 (0.24,2.14) |

UI, uncertainty interval.

## **Table S11.** Percentage rates of death of early-onset gastrointestinal cancers from 1990 to 2021.

| **Location** | **Esophageal cancer** **(95%UI)** | **Stomach cancer (95%UI)** | **Colorectal cancer (95%UI)** | **Liver cancer (95%UI)** | **Pancreatic cancer (95%UI)** | **Gallbladder and biliary tract cancer (95%UI)** |
| --- | --- | --- | --- | --- | --- | --- |
| Afghanistan | -0.37 (-0.57,0.08) | -0.31 (-0.55,0.04) | -0.11 (-0.41,0.69) | -0.18 (-0.5,0.32) | 0.23 (-0.23,1.24) | 0.05 (-0.38,1.06) |
| Albania | 0.14 (-0.31,0.86) | -0.25 (-0.47,0.12) | 0.21 (-0.21,0.82) | -0.19 (-0.52,0.31) | 0.65 (0.10,1.47) | 0.07 (-0.33,0.58) |
| Algeria | 0.29 (-0.10,0.89) | -0.24 (-0.44,0.01) | 0.13 (-0.18,0.61) | 0.64 (-0.03,1.77) | 0.82 (0.27,1.65) | 0.10 (-0.22,0.61) |
| American Samoa | 0.92 (0.17,1.94) | 0.15 (-0.18,0.6) | 0.52 (0.04,1.21) | 1.17 (0.37,2.56) | 1.34 (0.5,2.41) | 0.31 (-0.17,0.95) |
| Andorra | -0.09 (-0.51,0.63) | -0.31 (-0.6,0.14) | -0.13 (-0.49,0.41) | 0.23 (-0.35,1.09) | 0.07 (-0.39,0.84) | -0.14 (-0.53,0.45) |
| Angola | -0.38 (-0.61,0.03) | -0.41 (-0.62,-0.14) | 0.05 (-0.32,0.74) | -0.39 (-0.64,0.25) | 0.19 (-0.35,1.04) | 0.08 (-0.36,0.96) |
| Antigua and Barbuda | -0.08 (-0.25,0.08) | -0.46 (-0.53,-0.37) | 0.09 (-0.07,0.27) | -0.2 (-0.35,-0.02) | 0.29 (0.10,0.54) | -0.54 (-0.61,-0.46) |
| Argentina | -0.54 (-0.61,-0.45) | -0.38 (-0.47,-0.27) | 0.04 (-0.14,0.24) | 0.98 (0.55,1.56) | -0.13 (-0.25,0.03) | -0.37 (-0.45,-0.28) |
| Armenia | -0.65 (-0.70,-0.60) | -0.57 (-0.62,-0.51) | -0.33 (-0.42,-0.23) | 0.08 (-0.21,0.42) | 0.66 (0.24,1.20) | 0.90 (0.45,1.49) |
| Australia | 0.22 (0.03,0.45) | -0.31 (-0.4,-0.22) | -0.21 (-0.35,-0.03) | 1.34 (0.98,1.77) | 0.28 (0.11,0.48) | -0.18 (-0.26,-0.09) |
| Austria | -0.42 (-0.52,-0.29) | -0.66 (-0.7,-0.62) | -0.5 (-0.58,-0.40) | 0.30 (0.08,0.57) | -0.23 (-0.34,-0.11) | -0.57 (-0.62,-0.5) |
| Azerbaijan | -0.36 (-0.53,-0.15) | -0.47 (-0.61,-0.27) | -0.24 (-0.44,0.00) | 0.37 (-0.42,2.16) | 0.78 (-0.02,2.2) | -0.12 (-0.57,0.66) |
| Bahamas | 0.37 (0.03,0.86) | -0.1 (-0.32,0.18) | 0.48 (0.08,0.94) | 0.13 (-0.17,0.49) | 0.61 (0.18,1.2) | -0.16 (-0.38,0.12) |
| Bahrain | -0.13 (-0.41,0.25) | -0.28 (-0.46,-0.07) | 0.23 (-0.11,0.67) | -0.17 (-0.44,0.22) | 0.65 (0.15,1.38) | 0.13 (-0.18,0.65) |
| Bangladesh | -0.24 (-0.50,0.19) | -0.45 (-0.62,-0.21) | -0.12 (-0.45,0.67) | 0.09 (-0.32,0.77) | 0.3 (-0.27,1.17) | 0.13 (-0.31,1.08) |
| Barbados | 0.12 (-0.20,0.53) | -0.31 (-0.5,-0.08) | 0.19 (-0.14,0.58) | 0.17 (-0.14,0.56) | 0.23 (-0.09,0.69) | -0.21 (-0.41,0.04) |
| Belarus | 0.18 (-0.13,0.59) | -0.52 (-0.64,-0.38) | -0.04 (-0.32,0.29) | 0.43 (0.01,0.97) | 0.88 (0.46,1.37) | 0.22 (-0.09,0.62) |
| Belgium | -0.26 (-0.38,-0.11) | -0.50 (-0.57,-0.42) | -0.35 (-0.47,-0.22) | 0.50 (0.22,0.78) | 0.17 (0.01,0.33) | -0.38 (-0.47,-0.29) |
| Belize | 0.93 (0.58,1.34) | 0.16 (0.00,0.37) | 1.05 (0.72,1.44) | 0.91 (0.52,1.31) | 1.20 (0.54,1.89) | -0.17 (-0.38,0.11) |
| Benin | 0.65 (0.07,1.65) | -0.30 (-0.50,0.01) | 0.09 (-0.25,0.63) | -0.37 (-0.62,0.12) | 0.79 (0.13,1.74) | -0.15 (-0.42,0.25) |
| Bermuda | -0.18 (-0.39,0.11) | -0.53 (-0.64,-0.39) | -0.11 (-0.39,0.24) | -0.38 (-0.52,-0.22) | 0.10 (-0.17,0.57) | -0.53 (-0.65,-0.36) |
| Bhutan | -0.14 (-0.5,0.47) | -0.34 (-0.58,0.06) | 0.09 (-0.35,0.93) | 0.30 (-0.30,1.51) | 0.81 (0.13,2.10) | 0.30 (-0.23,1.40) |
| Bolivia (Plurinational State of) | -0.27 (-0.53,0.13) | -0.4 (-0.59,-0.15) | -0.01 (-0.34,0.62) | -0.13 (-0.47,0.39) | 0.18 (-0.22,1.03) | -0.25 (-0.53,0.24) |
| Bosnia and Herzegovina | 0.07 (-0.26,0.46) | -0.36 (-0.55,-0.13) | 0.17 (-0.23,0.54) | -0.3 (-0.56,0.05) | 0.31 (-0.09,0.77) | -0.28 (-0.56,0.25) |
| Botswana | -0.16 (-0.49,0.38) | -0.38 (-0.61,-0.04) | 0.20 (-0.26,0.98) | 0.43 (-0.44,2.85) | 0.63 (-0.11,1.83) | 0.11 (-0.39,0.94) |
| Brazil | -0.03 (-0.1,0.04) | -0.23 (-0.27,-0.19) | 0.75 (0.63,0.88) | 0.04 (-0.03,0.11) | 0.53 (0.45,0.62) | 0.02 (-0.04,0.07) |
| Brunei Darussalam | 0.23 (-0.11,0.75) | -0.42 (-0.57,-0.22) | -0.01 (-0.28,0.37) | -0.02 (-0.46,0.85) | 0.62 (0.15,1.25) | -0.05 (-0.31,0.31) |
| Bulgaria | 0.03 (-0.17,0.28) | -0.43 (-0.58,-0.24) | 0.07 (-0.21,0.48) | -0.37 (-0.59,-0.08) | 0.23 (-0.07,0.61) | -0.02 (-0.27,0.32) |
| Burkina Faso | 0.63 (0.00,1.59) | -0.28 (-0.48,0.04) | 0.08 (-0.25,0.51) | -0.28 (-0.57,0.35) | 0.74 (0.24,1.45) | -0.11 (-0.42,0.29) |
| Burundi | -0.50 (-0.67,-0.20) | -0.43 (-0.61,-0.17) | -0.22 (-0.50,0.37) | -0.44 (-0.69,0.00) | -0.25 (-0.57,0.37) | -0.31 (-0.60,0.43) |
| Cabo Verde | 1.41 (0.62,2.63) | -0.36 (-0.57,-0.04) | 0.94 (0.34,1.84) | 0.33 (-0.25,1.40) | 11.38 (7.81,17.09) | 1.31 (-0.07,2.96) |
| Cambodia | -0.28 (-0.54,0.12) | -0.39 (-0.6,-0.09) | 0.15 (-0.29,1.01) | -0.19 (-0.53,0.41) | 0.51 (-0.10,1.59) | 0.07 (-0.46,0.97) |
| Cameroon | 0.92 (0.18,2.11) | -0.24 (-0.51,0.10) | 0.13 (-0.27,0.75) | -0.30 (-0.59,0.16) | 0.82 (0.17,1.68) | -0.07 (-0.4,0.36) |
| Canada | 0.39 (0.18,0.63) | -0.32 (-0.4,-0.22) | 0.04 (-0.11,0.23) | 0.67 (0.39,1.01) | -0.09 (-0.2,0.05) | -0.3 (-0.38,-0.20) |
| Central African Republic | -0.19 (-0.49,0.33) | -0.19 (-0.47,0.20) | 0.07 (-0.31,0.66) | -0.36 (-0.64,0.08) | 0.03 (-0.33,0.65) | -0.05 (-0.41,0.50) |
| Chad | 1.02 (0.31,2.05) | -0.04 (-0.33,0.38) | 0.33 (-0.13,0.86) | -0.24 (-0.54,0.43) | 1.00 (0.36,1.95) | 0.08 (-0.29,0.55) |
| Chile | -0.52 (-0.59,-0.44) | -0.43 (-0.52,-0.32) | 0.34 (0.15,0.56) | 0.76 (0.37,1.27) | 0.70 (0.46,0.95) | -0.65 (-0.69,-0.60) |
| China | -0.42 (-0.58,-0.21) | -0.44 (-0.56,-0.25) | 0.09 (-0.19,0.45) | 0.03 (-0.26,0.41) | 0.57 (0.13,1.15) | 0.13 (-0.17,0.60) |
| Colombia | -0.48 (-0.58,-0.34) | -0.24 (-0.39,-0.07) | 0.58 (0.26,0.99) | -0.29 (-0.45,-0.1) | 0.00 (-0.18,0.22) | -0.39 (-0.51,-0.26) |
| Comoros | -0.14 (-0.48,0.52) | -0.22 (-0.49,0.33) | 0.17 (-0.25,1.04) | 0.02 (-0.39,0.83) | 0.30 (-0.26,1.31) | 0.12 (-0.31,0.77) |
| Congo | -0.23 (-0.55,0.3) | -0.30 (-0.56,0.10) | 0.17 (-0.30,1.18) | -0.32 (-0.63,0.19) | 0.27 (-0.30,1.67) | 0.31 (-0.21,1.47) |
| Cook Islands | 0.15 (-0.32,0.82) | -0.30 (-0.55,0.10) | -0.07 (-0.4,0.48) | 0.40 (-0.17,1.46) | 0.53 (-0.06,1.37) | -0.35 (-0.72,0.83) |
| Costa Rica | -0.03 (-0.21,0.16) | -0.20 (-0.31,-0.05) | 1.71 (1.20,2.38) | 0.20 (0.00,0.43) | 0.76 (0.48,1.08) | -0.22 (-0.35,-0.08) |
| Croatia | -0.35 (-0.53,-0.12) | -0.65 (-0.73,-0.54) | -0.09 (-0.3,0.18) | -0.44 (-0.63,-0.15) | -0.20 (-0.36,0.00) | -0.48 (-0.60,-0.31) |
| Cuba | 1.13 (0.63,1.71) | -0.21 (-0.35,-0.04) | 0.33 (0.07,0.67) | -0.13 (-0.33,0.1) | 0.63 (0.30,1.04) | -0.43 (-0.55,-0.29) |
| Cyprus | 0.52 (0.03,1.24) | -0.41 (-0.55,-0.21) | -0.23 (-0.44,0.09) | -0.17 (-0.44,0.24) | 0.49 (-0.03,1.31) | -0.39 (-0.56,-0.11) |
| Czechia | -0.2 (-0.38,0.01) | -0.66 (-0.74,-0.55) | -0.36 (-0.52,-0.15) | -0.60 (-0.71,-0.46) | -0.15 (-0.32,0.06) | -0.53 (-0.62,-0.42) |
| C么te d'Ivoire | 0.23 (-0.22,0.96) | -0.11 (-0.41,0.34) | 0.16 (-0.25,0.74) | -0.37 (-0.68,0.36) | 0.53 (-0.03,1.26) | 0.06 (-0.28,0.57) |
| Democratic People's Republic of Korea | 0.05 (-0.41,0.74) | -0.04 (-0.41,0.50) | 0.11 (-0.29,0.78) | -0.22 (-0.62,0.86) | 0.30 (-0.24,1.16) | 0.03 (-0.40,0.63) |
| Democratic Republic of the Congo | -0.18 (-0.48,0.3) | -0.17 (-0.44,0.20) | 0.06 (-0.34,0.66) | -0.22 (-0.54,0.24) | 0.05 (-0.42,0.84) | 0.04 (-0.34,0.75) |
| Denmark | -0.34 (-0.45,-0.22) | -0.63 (-0.69,-0.57) | -0.52 (-0.62,-0.41) | -0.25 (-0.38,-0.08) | -0.14 (-0.27,0.01) | -0.60 (-0.66,-0.54) |
| Djibouti | 0.00 (-0.41,0.68) | 0.00 (-0.39,0.62) | 0.53 (-0.04,1.52) | 0.39 (-0.22,1.31) | 0.60 (-0.02,1.47) | 0.33 (-0.19,1.22) |
| Dominica | 0.55 (0.05,1.32) | -0.01 (-0.26,0.35) | 0.69 (0.22,1.30) | 0.55 (-0.15,1.62) | 1.05 (0.45,2.03) | 0.17 (-0.20,0.60) |
| Dominican Republic | 0.67 (0.08,1.56) | 0.07 (-0.21,0.45) | 0.60 (0.14,1.16) | 0.77 (0.06,1.74) | 1.29 (0.62,2.29) | -0.06 (-0.37,0.61) |
| Ecuador | -0.33 (-0.51,-0.12) | -0.17 (-0.38,0.08) | 0.79 (0.33,1.37) | -0.41 (-0.56,-0.2) | 0.68 (0.24,1.22) | -0.32 (-0.50,-0.11) |
| Egypt | -0.39 (-0.59,-0.12) | 0.14 (-0.38,0.55) | 0.24 (-0.04,0.59) | 0.09 (-0.35,0.78) | 0.85 (0.36,1.49) | -0.11 (-0.33,0.17) |
| El Salvador | 0.08 (-0.19,0.4) | 0.02 (-0.23,0.38) | 0.80 (0.34,1.39) | 0.14 (-0.15,0.55) | 0.86 (0.42,1.40) | -0.06 (-0.31,0.35) |
| Equatorial Guinea | -0.53 (-0.73,-0.19) | -0.69 (-0.81,-0.47) | -0.02 (-0.45,0.84) | 0.21 (-0.40,1.32) | 0.45 (-0.26,1.91) | -0.18 (-0.61,0.66) |
| Eritrea | -0.32 (-0.57,0.1) | -0.28 (-0.53,0.08) | 0.16 (-0.28,0.9) | -0.05 (-0.44,0.58) | 0.13 (-0.35,0.96) | 0.04 (-0.35,0.81) |
| Estonia | -0.46 (-0.58,-0.31) | -0.73 (-0.78,-0.67) | -0.33 (-0.5,-0.12) | -0.18 (-0.37,0.06) | -0.06 (-0.26,0.17) | -0.50 (-0.59,-0.38) |
| Eswatini | 0.46 (-0.15,1.49) | 0.23 (-0.26,0.91) | 0.90 (0.14,2.08) | 2.33 (-0.33,15.02) | 1.24 (0.26,2.86) | 0.62 (-0.06,1.52) |
| Ethiopia | -0.57 (-0.71,-0.29) | -0.67 (-0.76,-0.55) | -0.41 (-0.59,0.22) | -0.42 (-0.64,0.04) | -0.13 (-0.49,0.62) | -0.45 (-0.67,0.32) |
| Fiji | 0.25 (-0.19,0.89) | -0.22 (-0.49,0.10) | 0.10 (-0.27,0.65) | 0.23 (-0.34,1.35) | 0.51 (0.03,1.14) | 0.11 (-0.28,0.73) |
| Finland | -0.41 (-0.48,-0.34) | -0.75 (-0.79,-0.70) | -0.47 (-0.55,-0.36) | -0.32 (-0.44,-0.19) | -0.32 (-0.42,-0.21) | -0.55 (-0.62,-0.48) |
| France | -0.68 (-0.74,-0.61) | -0.38 (-0.49,-0.23) | -0.22 (-0.37,-0.06) | 0.27 (0.03,0.54) | 0.11 (-0.05,0.29) | -0.50 (-0.57,-0.43) |
| Gabon | -0.10 (-0.47,0.49) | -0.32 (-0.55,0.08) | 0.10 (-0.34,0.90) | 0.02 (-0.48,0.98) | 0.59 (-0.09,1.70) | 0.11 (-0.33,0.92) |
| Gambia | 0.38 (-0.17,1.17) | -0.14 (-0.44,0.30) | 0.23 (-0.21,0.90) | 0.17 (-0.35,1.17) | 0.62 (0.00,1.58) | 0.14 (-0.24,0.69) |
| Georgia | -0.47 (-0.56,-0.37) | -0.47 (-0.55,-0.36) | -0.07 (-0.23,0.12) | -0.03 (-0.25,0.25) | 3.56 (2.53,4.78) | -0.38 (-0.58,-0.09) |
| Germany | -0.44 (-0.54,-0.32) | -0.53 (-0.58,-0.47) | -0.37 (-0.48,-0.24) | 0.47 (0.15,0.87) | -0.09 (-0.2,0.06) | -0.53 (-0.58,-0.47) |
| Ghana | 0.35 (-0.12,1.04) | -0.32 (-0.53,0.02) | 0.17 (-0.26,0.93) | -0.35 (-0.67,0.42) | 1.64 (0.82,3.07) | 0.02 (-0.32,0.55) |
| Greece | 0.44 (0.27,0.66) | -0.34 (-0.41,-0.28) | 0.14 (0.02,0.28) | 1.33 (1.10,1.59) | 0.55 (0.40,0.73) | -0.05 (-0.13,0.05) |
| Greenland | -0.45 (-0.65,-0.13) | -0.58 (-0.71,-0.42) | -0.43 (-0.6,-0.21) | -0.31 (-0.63,0.26) | -0.23 (-0.47,0.09) | -0.63 (-0.78,-0.14) |
| Grenada | -0.06 (-0.31,0.25) | -0.39 (-0.53,-0.22) | 0.3 (-0.02,0.74) | 0.72 (0.21,1.44) | 0.66 (0.31,1.17) | -0.17 (-0.36,0.14) |
| Guam | 1.13 (0.67,1.72) | 0.49 (0.22,0.83) | 0.79 (0.49,1.19) | 2.05 (1.34,2.87) | 2.09 (1.48,2.74) | 0.57 (0.21,1.00) |
| Guatemala | -0.41 (-0.51,-0.3) | -0.12 (-0.26,0.03) | 0.59 (0.32,0.90) | -0.27 (-0.38,-0.15) | 0.24 (0.05,0.48) | -0.6 (-0.67,-0.52) |
| Guinea | -0.01 (-0.41,0.65) | -0.23 (-0.46,0.15) | 0.03 (-0.35,0.68) | -0.28 (-0.59,0.26) | 0.57 (-0.01,1.35) | -0.06 (-0.4,0.44) |
| Guinea-Bissau | 0.58 (-0.04,1.58) | -0.24 (-0.49,0.15) | 0.11 (-0.28,0.76) | -0.4 (-0.66,0.39) | 0.67 (0.07,1.63) | -0.05 (-0.39,0.56) |
| Guyana | 0.72 (0.16,1.39) | -0.04 (-0.33,0.32) | 0.88 (0.29,1.63) | 0.38 (-0.07,0.92) | 1.24 (0.56,2.03) | 0.01 (-0.29,0.34) |
| Haiti | -0.19 (-0.47,0.24) | -0.34 (-0.55,-0.04) | -0.04 (-0.38,0.59) | -0.18 (-0.53,0.42) | 0.04 (-0.37,0.74) | -0.24 (-0.5,0.22) |
| Honduras | -0.15 (-0.5,0.33) | -0.33 (-0.58,0.00) | -0.06 (-0.42,0.42) | 0.13 (-0.34,0.94) | 0.3 (-0.2,1.08) | 0.01 (-0.38,0.53) |
| Hungary | -0.63 (-0.71,-0.53) | -0.64 (-0.71,-0.56) | -0.18 (-0.37,0.05) | -0.59 (-0.72,-0.42) | -0.2 (-0.36,-0.01) | -0.52 (-0.61,-0.41) |
| Iceland | 0.37 (0.14,0.64) | -0.59 (-0.66,-0.51) | -0.18 (-0.34,0.01) | 0.24 (-0.03,0.54) | 0.4 (0.18,0.68) | -0.29 (-0.4,-0.16) |
| India | -0.13 (-0.26,0.05) | -0.33 (-0.43,-0.21) | 0.04 (-0.16,0.36) | 0.40 (0.16,0.66) | 0.42 (0.06,0.86) | 0.31 (-0.05,0.65) |
| Indonesia | -0.09 (-0.32,0.28) | -0.27 (-0.43,-0.01) | 0.33 (-0.05,0.93) | 0.38 (-0.01,0.93) | 0.89 (0.38,1.56) | 0.05 (-0.28,0.66) |
| Iran (Islamic Republic of) | -0.01 (-0.18,0.25) | -0.27 (-0.38,-0.14) | 0.33 (0.06,0.86) | 0.65 (0.3,0.98) | 1.52 (0.96,2.21) | 1.09 (0.17,1.93) |
| Iraq | -0.02 (-0.36,0.54) | -0.29 (-0.52,0.09) | 0.09 (-0.26,0.72) | 0.08 (-0.33,0.76) | 0.52 (-0.08,1.65) | -0.08 (-0.38,0.42) |
| Ireland | -0.09 (-0.26,0.12) | -0.55 (-0.61,-0.49) | -0.34 (-0.46,-0.21) | 0.67 (0.38,1.01) | 0.01 (-0.15,0.17) | -0.41 (-0.49,-0.31) |
| Israel | -0.07 (-0.23,0.12) | -0.52 (-0.6,-0.42) | -0.24 (-0.36,-0.1) | 0.5 (0.15,0.93) | 0.05 (-0.08,0.21) | -0.46 (-0.53,-0.36) |
| Italy | -0.48 (-0.52,-0.45) | -0.56 (-0.59,-0.54) | -0.25 (-0.3,-0.2) | -0.15 (-0.22,-0.08) | 0.00 (-0.06,0.05) | -0.39 (-0.42,-0.36) |
| Jamaica | 0.74 (0.20,1.52) | 0.10 (-0.22,0.50) | 1.29 (0.58,2.15) | 1.22 (0.45,2.20) | 0.85 (0.16,1.81) | 0.08 (-0.33,0.69) |
| Japan | -0.39 (-0.41,-0.36) | -0.73 (-0.74,-0.73) | -0.24 (-0.27,-0.21) | -0.63 (-0.65,-0.62) | 0.03 (0.01,0.06) | -0.56 (-0.57,-0.55) |
| Jordan | -0.03 (-0.37,0.52) | -0.45 (-0.61,-0.22) | -0.08 (-0.39,0.44) | -0.15 (-0.49,0.47) | 0.67 (0.07,1.6) | -0.19 (-0.49,0.26) |
| Kazakhstan | -0.7 (-0.74,-0.66) | -0.65 (-0.7,-0.6) | -0.34 (-0.42,-0.24) | -0.53 (-0.62,-0.41) | 0.28 (-0.22,0.84) | 0.13 (-0.22,0.71) |
| Kenya | 0.46 (0.11,1.00) | 0.09 (-0.16,0.47) | 0.52 (0.17,1.08) | 0.85 (0.40,1.44) | 1.28 (0.74,2.16) | 0.36 (0.01,0.83) |
| Kiribati | 0.11 (-0.3,0.79) | 0.00 (-0.32,0.56) | 0.13 (-0.26,0.65) | 0.00 (-0.44,0.83) | 0.37 (-0.16,1.25) | 0.13 (-0.29,0.92) |
| Kuwait | -0.26 (-0.43,-0.02) | -0.27 (-0.42,-0.08) | 1.27 (0.73,2.05) | -0.76 (-0.82,-0.68) | 1.12 (0.69,1.81) | 0.06 (-0.15,0.33) |
| Kyrgyzstan | -0.7 (-0.78,-0.58) | -0.58 (-0.67,-0.46) | -0.26 (-0.45,-0.01) | -0.67 (-0.81,-0.47) | 0.96 (0.46,1.77) | 0.04 (-0.24,0.42) |
| Lao People's Democratic Republic | -0.45 (-0.67,-0.07) | -0.56 (-0.71,-0.34) | -0.05 (-0.40,0.70) | -0.33 (-0.62,0.18) | 0.25 (-0.31,1.18) | -0.24 (-0.59,0.53) |
| Latvia | -0.25 (-0.42,-0.02) | -0.59 (-0.67,-0.49) | -0.21 (-0.39,0.02) | -0.03 (-0.32,0.33) | 0.45 (0.13,0.82) | -0.13 (-0.29,0.08) |
| Lebanon | -0.36 (-0.55,-0.02) | -0.58 (-0.7,-0.43) | -0.26 (-0.51,0.44) | -0.3 (-0.56,0.13) | 0.07 (-0.39,1.23) | -0.36 (-0.61,0.28) |
| Lesotho | 0.90 (0.18,2.10) | 0.75 (0.06,1.84) | 1.55 (0.50,3.20) | 2.68 (-0.06,17.21) | 1.76 (0.47,3.63) | 1.00 (0.08,2.51) |
| Liberia | 1.52 (0.59,2.99) | 0.04 (-0.32,0.61) | 0.35 (-0.22,1.21) | -0.02 (-0.44,0.7) | 1.05 (0.14,2.24) | 0.05 (-0.34,0.73) |
| Libya | 1.07 (0.15,2.81) | 0.28 (-0.18,1.07) | 0.61 (0.00,1.63) | 1.08 (0.09,2.7) | 1.38 (0.34,3.22) | 0.66 (0.08,1.53) |
| Lithuania | 0.02 (-0.21,0.3) | -0.54 (-0.63,-0.44) | -0.12 (-0.33,0.13) | 0.34 (0.02,0.74) | 0.32 (0.07,0.58) | -0.13 (-0.29,0.03) |
| Luxembourg | -0.54 (-0.61,-0.45) | -0.78 (-0.8,-0.75) | -0.59 (-0.64,-0.52) | -0.12 (-0.24,0.02) | -0.35 (-0.45,-0.24) | -0.65 (-0.70,-0.60) |
| Madagascar | -0.24 (-0.51,0.13) | -0.24 (-0.48,0.07) | 0.00 (-0.30,0.42) | -0.23 (-0.52,0.22) | 0.00 (-0.37,0.63) | -0.01 (-0.34,0.48) |
| Malawi | 0.17 (-0.23,0.75) | -0.21 (-0.43,0.06) | 0.11 (-0.23,0.61) | 0.41 (-0.2,1.47) | 0.35 (-0.13,1.11) | -0.07 (-0.41,0.51) |
| Malaysia | 0.33 (-0.02,0.80) | -0.14 (-0.3,0.07) | 0.28 (0.01,0.64) | 0.59 (-0.01,1.42) | 0.89 (0.37,1.57) | 0.07 (-0.23,0.45) |
| Maldives | -0.59 (-0.74,-0.22) | -0.7 (-0.79,-0.54) | -0.37 (-0.6,0.84) | -0.31 (-0.62,0.41) | 0.09 (-0.41,1.85) | -0.50 (-0.75,0.71) |
| Mali | -0.16 (-0.44,0.27) | -0.4 (-0.56,-0.15) | -0.16 (-0.43,0.19) | -0.11 (-0.4,0.37) | 0.10 (-0.28,0.63) | -0.21 (-0.48,0.22) |
| Malta | 0.23 (0.00,0.48) | -0.5 (-0.57,-0.41) | -0.10 (-0.28,0.09) | 0.80 (0.47,1.28) | 0.61 (0.34,0.95) | -0.28 (-0.39,-0.13) |
| Marshall Islands | 0.47 (-0.09,1.30) | 0.11 (-0.19,0.56) | 0.51 (0.04,1.08) | 0.72 (-0.04,1.87) | 1.42 (0.66,2.62) | 0.36 (-0.12,0.97) |
| Mauritania | 0.36 (-0.12,1.05) | -0.4 (-0.58,-0.13) | 0.00 (-0.35,0.72) | -0.55 (-0.74,0.34) | 0.64 (0.04,1.78) | -0.22 (-0.51,0.30) |
| Mauritius | 0.70 (0.48,0.91) | 0.05 (-0.09,0.20) | 1.56 (1.25,1.87) | -0.78 (-0.8,-0.75) | 0.84 (0.63,1.07) | -0.32 (-0.4,-0.24) |
| Mexico | 0.06 (-0.08,0.21) | 0.09 (-0.04,0.22) | 1.35 (1.08,1.65) | 0.71 (0.51,0.94) | 0.33 (0.18,0.51) | -0.35 (-0.44,-0.26) |
| Micronesia (Federated States of) | 0.06 (-0.39,0.88) | -0.12 (-0.42,0.34) | 0.19 (-0.23,0.88) | 0.23 (-0.35,1.24) | 0.85 (0.18,1.92) | -0.03 (-0.41,0.64) |
| Monaco | 0.21 (-0.23,0.93) | -0.39 (-0.63,-0.01) | 0.08 (-0.31,0.64) | 1.13 (0.27,2.61) | 0.09 (-0.34,0.74) | -0.13 (-0.48,0.37) |
| Mongolia | -0.06 (-0.39,0.4) | 0.01 (-0.3,0.39) | 0.51 (0.09,1.07) | 0.42 (-0.24,1.43) | 9.96 (6.56,14.73) | -0.16 (-0.53,0.92) |
| Montenegro | 0.15 (-0.18,0.67) | -0.24 (-0.4,-0.04) | 0.05 (-0.22,0.4) | 0.11 (-0.24,0.61) | 0.23 (-0.14,0.84) | -0.09 (-0.32,0.20) |
| Morocco | 0.13 (-0.27,0.78) | -0.21 (-0.47,0.18) | 0.47 (-0.04,1.33) | 0.46 (-0.14,1.55) | 0.82 (0.2,1.92) | 0.23 (-0.19,1.00) |
| Mozambique | 0.31 (-0.25,1.12) | -0.03 (-0.38,0.48) | 0.28 (-0.17,0.93) | 0.28 (-0.51,3.14) | 0.56 (-0.03,1.56) | 0.11 (-0.29,0.73) |
| Myanmar | -0.4 (-0.63,0.00) | -0.55 (-0.71,-0.32) | 0.01 (-0.39,0.68) | 0.03 (-0.44,1.05) | 0.42 (-0.19,1.57) | -0.21 (-0.54,0.41) |
| Namibia | 0.32 (-0.24,1.37) | -0.06 (-0.42,0.46) | 0.31 (-0.2,1.00) | 0.89 (-0.07,2.79) | 0.76 (0.09,1.73) | 0.23 (-0.27,0.96) |
| Nauru | -0.08 (-0.42,0.44) | -0.17 (-0.41,0.24) | -0.04 (-0.37,0.44) | -0.09 (-0.51,0.7) | 0.31 (-0.12,1.00) | -0.16 (-0.47,0.34) |
| Nepal | -0.25 (-0.52,0.16) | -0.42 (-0.62,-0.15) | -0.05 (-0.37,0.62) | 0.68 (0.00,1.78) | 0.42 (-0.09,1.28) | 0.18 (-0.26,0.92) |
| Netherlands | -0.23 (-0.36,-0.08) | -0.62 (-0.67,-0.57) | -0.22 (-0.37,-0.06) | 0.60 (0.31,0.94) | -0.08 (-0.20,0.04) | -0.41 (-0.49,-0.32) |
| New Zealand | -0.16 (-0.28,-0.02) | -0.45 (-0.54,-0.36) | -0.32 (-0.44,-0.20) | 0.91 (0.59,1.33) | 0.12 (-0.02,0.28) | 0.17 (0.04,0.32) |
| Nicaragua | -0.05 (-0.31,0.33) | -0.19 (-0.37,0.04) | 0.42 (0.06,0.88) | 0.13 (-0.26,0.69) | 0.58 (0.17,1.10) | -0.27 (-0.50,0.16) |
| Niger | 0.11 (-0.31,0.73) | -0.37 (-0.56,-0.10) | -0.15 (-0.42,0.27) | -0.62 (-0.76,-0.29) | 0.18 (-0.25,0.84) | -0.27 (-0.53,0.03) |
| Nigeria | 0.46 (-0.01,1.12) | -0.37 (-0.59,-0.01) | -0.01 (-0.34,0.57) | -0.29 (-0.53,0.41) | 0.51 (-0.02,1.35) | 0.01 (-0.38,0.58) |
| Niue | 0.11 (-0.33,0.86) | -0.15 (-0.45,0.32) | 0.21 (-0.22,0.96) | 0.23 (-0.37,1.34) | 0.90 (0.16,2.07) | -0.07 (-0.42,0.45) |
| North Macedonia | -0.08 (-0.37,0.33) | -0.48 (-0.60,-0.31) | -0.10 (-0.32,0.20) | -0.28 (-0.52,0.05) | 0.12 (-0.19,0.56) | -0.28 (-0.47,0.03) |
| Northern Mariana Islands | 1.15 (0.44,2.29) | -0.13 (-0.40,0.31) | 0.17 (-0.19,0.80) | 0.21 (-0.39,1.28) | 1.61 (0.70,3.03) | 0.24 (-0.20,0.93) |
| Norway | -0.06 (-0.12,0.01) | -0.71 (-0.73,-0.69) | -0.4 (-0.44,-0.34) | 1.07 (0.90,1.27) | -0.08 (-0.14,-0.03) | -0.29 (-0.34,-0.25) |
| Oman | -0.15 (-0.45,0.35) | -0.58 (-0.73,-0.34) | -0.19 (-0.53,0.39) | 0.34 (-0.24,1.51) | 0.55 (-0.06,1.43) | -0.25 (-0.56,0.19) |
| Pakistan | 0.08 (-0.23,0.51) | -0.04 (-0.3,0.33) | 0.34 (0.00,0.80) | 0.35 (-0.03,0.95) | 0.68 (0.19,1.40) | 0.21 (-0.13,0.69) |
| Palau | 0.73 (0.11,1.71) | 0.22 (-0.14,0.76) | 0.21 (-0.18,0.87) | 1.01 (0.03,2.90) | 0.59 (0.00,1.54) | 0.47 (-0.02,1.19) |
| Palestine | -0.34 (-0.58,0.00) | -0.46 (-0.62,-0.22) | -0.18 (-0.45,0.27) | -0.18 (-0.46,0.21) | 0.24 (-0.26,1.06) | -0.18 (-0.46,0.31) |
| Panama | -0.15 (-0.36,0.07) | -0.06 (-0.29,0.16) | 0.75 (0.34,1.18) | 0.17 (-0.09,0.43) | 0.61 (0.24,0.99) | -0.37 (-0.51,-0.21) |
| Papua New Guinea | -0.13 (-0.5,0.52) | -0.20 (-0.48,0.23) | -0.01 (-0.36,0.65) | -0.26 (-0.61,0.46) | 0.43 (-0.13,1.49) | -0.17 (-0.52,0.51) |
| Paraguay | 0.34 (-0.18,1.12) | -0.18 (-0.41,0.16) | 0.64 (0.14,1.32) | 0.62 (-0.02,1.47) | 0.99 (0.32,1.87) | -0.05 (-0.37,0.36) |
| Peru | -0.23 (-0.48,0.13) | -0.24 (-0.47,0.08) | 0.16 (-0.17,0.59) | 0.08 (-0.33,0.62) | 0.15 (-0.22,0.7) | -0.10 (-0.41,0.29) |
| Philippines | -0.07 (-0.25,0.16) | -0.26 (-0.40,-0.08) | 0.25 (0.02,0.50) | -0.26 (-0.46,0.19) | 0.40 (0.12,0.76) | 0.09 (-0.11,0.39) |
| Poland | -0.22 (-0.31,-0.15) | -0.53 (-0.57,-0.48) | -0.07 (-0.16,0.02) | 2.35 (1.84,2.89) | -0.20 (-0.28,-0.13) | -0.27 (-0.33,-0.20) |
| Portugal | -0.13 (-0.28,0.06) | -0.61 (-0.66,-0.55) | -0.05 (-0.2,0.13) | 1.12 (0.62,1.83) | 0.20 (0.02,0.39) | -0.32 (-0.42,-0.20) |
| Puerto Rico | -0.42 (-0.57,-0.24) | -0.53 (-0.65,-0.41) | 0.20 (-0.05,0.53) | 0.23 (-0.08,0.66) | 1.83 (1.17,2.59) | -0.40 (-0.54,-0.25) |
| Qatar | -0.35 (-0.6,0.01) | -0.56 (-0.71,-0.39) | -0.15 (-0.4,0.21) | 0.08 (-0.36,0.72) | 0.14 (-0.22,0.67) | -0.30 (-0.53,0.06) |
| Republic of Korea | -0.64 (-0.75,-0.47) | -0.73 (-0.79,-0.59) | -0.14 (-0.34,0.15) | -0.52 (-0.7,-0.19) | -0.08 (-0.29,0.22) | -0.46 (-0.64,0.12) |
| Republic of Moldova | -0.3 (-0.4,-0.18) | -0.58 (-0.64,-0.51) | -0.09 (-0.25,0.09) | 0.21 (0.03,0.38) | 0.29 (0.12,0.48) | -0.25 (-0.37,-0.11) |
| Romania | 0.71 (0.33,1.18) | -0.27 (-0.44,-0.08) | 0.38 (0.10,0.71) | 0.90 (0.42,1.52) | 0.52 (0.24,0.87) | -0.30 (-0.41,-0.16) |
| Russian Federation | -0.06 (-0.16,0.02) | -0.57 (-0.61,-0.53) | 0.05 (-0.06,0.14) | 0.82 (0.66,0.99) | 0.04 (-0.05,0.12) | 0.01 (-0.09,0.1) |
| Rwanda | -0.51 (-0.71,-0.16) | -0.56 (-0.71,-0.33) | -0.26 (-0.52,0.23) | -0.39 (-0.65,0.02) | -0.19 (-0.55,0.63) | -0.30 (-0.59,0.44) |
| Saint Kitts and Nevis | 0.07 (-0.20,0.43) | -0.51 (-0.63,-0.36) | -0.06 (-0.28,0.22) | -0.21 (-0.43,0.06) | 0.28 (-0.08,1.00) | -0.43 (-0.6,-0.16) |
| Saint Lucia | 0.41 (0.13,0.78) | -0.22 (-0.38,-0.03) | 0.42 (0.13,0.77) | 0.15 (-0.14,0.50) | 0.95 (0.56,1.49) | -0.17 (-0.33,0.07) |
| Saint Vincent and the Grenadines | 1.05 (0.70,1.48) | -0.02 (-0.19,0.20) | 0.93 (0.55,1.39) | 0.36 (0.09,0.65) | 0.98 (0.64,1.42) | -0.05 (-0.20,0.15) |
| Samoa | 0.32 (-0.18,1.05) | 0.19 (-0.26,0.82) | 0.41 (-0.1,1.21) | 0.25 (-0.41,1.50) | 0.61 (0.02,1.59) | 0.27 (-0.23,1.05) |
| San Marino | -0.26 (-0.67,0.4) | -0.5 (-0.75,-0.19) | -0.31 (-0.64,0.1) | 0.62 (-0.23,2.03) | -0.04 (-0.52,0.61) | -0.32 (-0.66,0.08) |
| Sao Tome and Principe | 1.39 (0.48,2.92) | -0.04 (-0.4,0.44) | 0.45 (-0.11,1.3) | -0.01 (-0.54,1.25) | 0.95 (0.14,2.28) | 0.37 (-0.15,1.13) |
| Saudi Arabia | 0.36 (-0.20,1.24) | -0.07 (-0.42,0.48) | 0.80 (0.01,2.02) | -0.26 (-0.65,0.48) | 1.87 (0.83,3.69) | 0.55 (0.01,1.49) |
| Senegal | 0.67 (0.12,1.55) | -0.24 (-0.47,0.07) | 0.16 (-0.18,0.7) | -0.38 (-0.61,0.15) | 0.86 (0.20,1.85) | -0.08 (-0.38,0.38) |
| Serbia | -0.17 (-0.5,0.41) | -0.5 (-0.64,-0.33) | -0.24 (-0.46,0.07) | -0.16 (-0.46,0.3) | -0.01 (-0.32,0.38) | -0.31 (-0.56,0.03) |
| Seychelles | 0.36 (-0.03,0.91) | -0.22 (-0.38,-0.03) | 0.63 (0.26,1.00) | -0.29 (-0.53,0.01) | 0.55 (0.17,1.07) | -0.20 (-0.45,0.32) |
| Sierra Leone | 0.82 (0.13,2.04) | -0.08 (-0.37,0.34) | 0.31 (-0.11,0.99) | -0.48 (-0.72,0.50) | 0.81 (0.20,1.71) | 0.01 (-0.31,0.51) |
| Singapore | -0.42 (-0.53,-0.3) | -0.70 (-0.74,-0.66) | -0.36 (-0.45,-0.24) | -0.54 (-0.62,-0.45) | 0.00 (-0.14,0.17) | -0.56 (-0.63,-0.49) |
| Slovakia | -0.31 (-0.60,0.15) | -0.53 (-0.66,-0.40) | -0.21 (-0.44,0.06) | -0.34 (-0.63,0.13) | -0.02 (-0.33,0.44) | -0.33 (-0.56,0.05) |
| Slovenia | -0.35 (-0.49,-0.20) | -0.71 (-0.78,-0.64) | -0.36 (-0.54,-0.12) | -0.54 (-0.68,-0.37) | -0.12 (-0.32,0.09) | -0.62 (-0.71,-0.50) |
| Solomon Islands | 0.27 (-0.26,1.51) | 0.08 (-0.30,0.86) | 0.43 (-0.08,1.59) | 0.10 (-0.45,2.38) | 1.15 (0.35,3.16) | 0.31 (-0.24,1.97) |
| Somalia | -0.38 (-0.6,0.00) | -0.25 (-0.51,0.16) | -0.12 (-0.44,0.40) | -0.13 (-0.56,0.63) | -0.26 (-0.55,0.23) | -0.12 (-0.43,0.45) |
| South Africa | -0.35 (-0.47,-0.21) | -0.37 (-0.45,-0.26) | 0.15 (-0.01,0.37) | 0.23 (-0.24,1.13) | 0.22 (0.02,0.46) | 0.04 (-0.13,0.26) |
| South Sudan | 0.09 (-0.27,0.66) | 0.14 (-0.27,0.70) | 0.44 (-0.09,1.41) | 0.36 (-0.19,1.28) | 0.36 (-0.22,1.33) | 0.36 (-0.14,1.16) |
| Spain | -0.5 (-0.59,-0.4) | -0.54 (-0.60,-0.47) | -0.27 (-0.39,-0.12) | 0.32 (0.08,0.59) | 0.12 (-0.02,0.31) | -0.42 (-0.49,-0.33) |
| Sri Lanka | -0.23 (-0.56,0.19) | -0.55 (-0.72,-0.35) | 0.00 (-0.39,0.54) | -0.19 (-0.52,0.29) | 0.17 (-0.36,0.89) | -0.64 (-0.82,0.16) |
| Sudan | -0.24 (-0.55,0.25) | -0.37 (-0.63,-0.03) | 0.04 (-0.38,0.86) | -0.04 (-0.51,0.65) | 0.74 (0.03,1.97) | 0.07 (-0.51,1.22) |
| Suriname | 0.41 (-0.10,1.15) | -0.09 (-0.33,0.24) | 0.50 (0.08,1.07) | 0.58 (-0.07,1.81) | 0.78 (0.16,1.73) | 0.01 (-0.30,0.47) |
| Sweden | -0.24 (-0.37,-0.09) | -0.72 (-0.77,-0.66) | -0.37 (-0.48,-0.25) | -0.12 (-0.28,0.08) | -0.39 (-0.49,-0.29) | -0.58 (-0.64,-0.50) |
| Switzerland | -0.52 (-0.60,-0.41) | -0.65 (-0.70,-0.60) | -0.48 (-0.55,-0.39) | -0.49 (-0.6,-0.35) | -0.22 (-0.33,-0.08) | -0.57 (-0.64,-0.48) |
| Syrian Arab Republic | 0.17 (-0.23,0.87) | -0.21 (-0.47,0.22) | -0.02 (-0.39,0.66) | -0.18 (-0.51,0.35) | 0.91 (0.14,2.17) | 0.32 (-0.19,1.29) |
| Taiwan (Province of China) | 1.17 (0.89,1.45) | -0.42 (-0.50,-0.31) | 0.41 (0.24,0.61) | -0.01 (-0.18,0.18) | 1.05 (0.76,1.40) | -0.11 (-0.24,0.03) |
| Tajikistan | -0.43 (-0.63,-0.15) | -0.44 (-0.61,-0.20) | -0.33 (-0.55,0.08) | -0.21 (-0.61,0.46) | 0.09 (-0.43,1.09) | -0.20 (-0.58,0.57) |
| Thailand | 1.51 (0.55,3.07) | 0.34 (-0.06,0.96) | 1.22 (0.59,2.18) | 0.33 (-0.17,1.10) | 1.61 (0.79,2.72) | 0.78 (0.19,1.56) |
| Timor-Leste | -0.27 (-0.58,0.20) | -0.41 (-0.60,-0.13) | 0.01 (-0.36,0.68) | -0.21 (-0.57,0.4) | 0.25 (-0.27,1.25) | -0.19 (-0.51,0.36) |
| Togo | 1.51 (0.62,3.05) | 0.03 (-0.31,0.52) | 0.46 (-0.03,1.21) | -0.04 (-0.42,0.58) | 1.35 (0.52,2.66) | 0.12 (-0.30,0.75) |
| Tokelau | 0.20 (-0.28,1.11) | -0.15 (-0.41,0.30) | 0.27 (-0.15,0.89) | 0.64 (-0.01,1.88) | 1.14 (0.34,2.51) | -0.12 (-0.43,0.42) |
| Tonga | 0.25 (-0.23,1.01) | -0.01 (-0.35,0.5) | 0.20 (-0.25,0.85) | 0.26 (-0.32,1.2) | 0.73 (0.13,1.72) | -0.09 (-0.42,0.38) |
| Trinidad and Tobago | 0.47 (0.06,0.97) | -0.10 (-0.34,0.19) | 0.59 (0.15,1.08) | 0.35 (-0.01,0.77) | 0.64 (0.17,1.19) | -0.22 (-0.45,0.07) |
| Tunisia | 0.55 (-0.06,1.47) | -0.03 (-0.34,0.46) | 0.49 (-0.01,1.16) | 0.72 (-0.08,1.92) | 1.28 (0.43,2.75) | 0.36 (-0.12,1.07) |
| Turkey | -0.37 (-0.57,-0.07) | -0.58 (-0.69,-0.45) | -0.3 (-0.51,0.04) | 0.01 (-0.35,0.54) | 0.18 (-0.24,0.81) | -0.37 (-0.58,0.00) |
| Turkmenistan | -0.52 (-0.64,-0.36) | -0.31 (-0.48,-0.08) | 0.11 (-0.17,0.46) | 0.37 (-0.06,0.96) | 23.09 (16.21,32.19) | -0.57 (-0.69,-0.40) |
| Tuvalu | -0.09 (-0.38,0.35) | -0.34 (-0.52,-0.08) | -0.05 (-0.33,0.38) | 0.13 (-0.31,0.89) | 0.61 (0.11,1.38) | -0.40 (-0.61,-0.03) |
| Uganda | 0.15 (-0.27,0.83) | -0.18 (-0.47,0.26) | 0.25 (-0.22,0.97) | 0.10 (-0.36,0.91) | 0.77 (0.08,2.03) | 0.20 (-0.26,0.99) |
| Ukraine | -0.15 (-0.45,0.27) | -0.57 (-0.7,-0.41) | -0.24 (-0.49,0.03) | 0.04 (-0.32,0.51) | 0.65 (-0.08,1.68) | 0.16 (-0.29,0.96) |
| United Arab Emirates | 0.00 (-0.32,0.48) | -0.48 (-0.62,-0.3) | -0.32 (-0.56,0.35) | 0.68 (0.06,1.65) | 0.42 (-0.16,1.3) | -0.16 (-0.42,0.33) |
| United Kingdom | -0.01 (-0.04,0.03) | -0.49 (-0.51,-0.48) | -0.19 (-0.22,-0.16) | 1.92 (1.83,2.03) | 0.01 (-0.03,0.04) | 0.09 (0.05,0.12) |
| United Republic of Tanzania | -0.27 (-0.53,0.15) | -0.31 (-0.54,0.00) | 0.12 (-0.27,0.61) | -0.16 (-0.49,0.42) | 0.19 (-0.25,0.74) | 0.05 (-0.31,0.57) |
| United States of America | -0.1 (-0.14,-0.06) | -0.21 (-0.24,-0.18) | 0.09 (0.04,0.13) | 0.49 (0.43,0.55) | 0.00 (-0.04,0.03) | -0.13 (-0.16,-0.09) |
| United States Virgin Islands | 0.17 (-0.27,0.79) | -0.29 (-0.51,0.02) | 0.04 (-0.28,0.47) | 0.51 (-0.21,1.87) | 0.88 (0.19,1.88) | -0.33 (-0.58,0.09) |
| Uruguay | -0.3 (-0.42,-0.16) | -0.24 (-0.34,-0.13) | 0.11 (-0.08,0.34) | 1.32 (0.61,2.30) | 0.26 (0.07,0.49) | -0.33 (-0.43,-0.22) |
| Uzbekistan | -0.54 (-0.65,-0.39) | -0.46 (-0.56,-0.35) | -0.04 (-0.23,0.18) | 0.54 (-0.02,1.44) | 1.33 (0.57,2.41) | 1.41 (0.58,2.59) |
| Vanuatu | 0.10 (-0.39,0.89) | -0.06 (-0.37,0.45) | 0.22 (-0.22,0.88) | 0.02 (-0.46,1.14) | 0.79 (0.14,1.79) | 0.09 (-0.35,0.72) |
| Venezuela (Bolivarian Republic of) | 0.20 (-0.17,0.64) | 0.11 (-0.18,0.50) | 0.83 (0.32,1.51) | 0.03 (-0.24,0.34) | 2.30 (1.21,3.48) | -0.18 (-0.44,0.15) |
| Viet Nam | 1.10 (0.26,2.59) | -0.12 (-0.39,0.32) | 0.91 (0.28,2.02) | 0.25 (-0.25,1.17) | 1.63 (0.63,3.15) | 0.66 (-0.05,1.83) |
| Yemen | -0.27 (-0.55,0.21) | -0.3 (-0.55,0.11) | 0.05 (-0.34,0.81) | -0.3 (-0.59,0.39) | 0.43 (-0.15,1.66) | 0.22 (-0.34,1.49) |
| Zambia | -0.08 (-0.47,0.64) | -0.23 (-0.5,0.25) | 0.49 (-0.21,2.79) | -0.53 (-0.83,0.11) | 0.31 (-0.22,1.06) | 0.07 (-0.30,0.61) |
| Zimbabwe | 1.26 (0.46,2.62) | 0.99 (0.25,1.96) | 1.35 (0.55,2.5) | 0.78 (-0.03,2.4) | 1.72 (0.82,3.27) | 1.01 (0.22,2.15) |

UI, uncertainty interval.

## **Table S12.** Percentage rates of DALYs of early-onset gastrointestinal cancers from 1990 to 2021.

| **Location** | **Esophageal cancer** **(95%UI)** | **Stomach cancer (95%UI)** | **Colorectal cancer (95%UI)** | **Liver cancer (95%UI)** | **Pancreatic cancer (95%UI)** | **Gallbladder and biliary tract cancer (95%UI)** |
| --- | --- | --- | --- | --- | --- | --- |
| Afghanistan | -0.36 (-0.56,0.08) | -0.3 (-0.54,0.05) | -0.09 (-0.40,0.74) | -0.17 (-0.48,0.31) | 0.25 (-0.21,1.25) | 0.07 (-0.37,1.09) |
| Albania | 0.10 (-0.33,0.80) | -0.28 (-0.49,0.07) | 0.17 (-0.24,0.78) | -0.23 (-0.54,0.21) | 0.59 (0.06,1.39) | 0.03 (-0.35,0.52) |
| Algeria | 0.27 (-0.11,0.84) | -0.27 (-0.46,-0.03) | 0.10 (-0.19,0.55) | 0.57 (-0.08,1.61) | 0.77 (0.23,1.53) | 0.08 (-0.23,0.58) |
| American Samoa | 0.88 (0.16,1.83) | 0.11 (-0.21,0.55) | 0.47 (-0.01,1.12) | 1.12 (0.34,2.52) | 1.30 (0.48,2.33) | 0.28 (-0.18,0.90) |
| Andorra | -0.11 (-0.51,0.59) | -0.34 (-0.62,0.10) | -0.14 (-0.50,0.40) | 0.17 (-0.37,0.99) | 0.04 (-0.4,0.77) | -0.17 (-0.54,0.41) |
| Angola | -0.38 (-0.61,0.02) | -0.41 (-0.61,-0.14) | 0.06 (-0.32,0.74) | -0.39 (-0.63,0.25) | 0.19 (-0.34,1.04) | 0.08 (-0.35,0.96) |
| Antigua and Barbuda | -0.12 (-0.27,0.04) | -0.48 (-0.55,-0.40) | 0.05 (-0.10,0.22) | -0.23 (-0.37,-0.06) | 0.24 (0.06,0.47) | -0.56 (-0.62,-0.48) |
| Argentina | -0.54 (-0.61,-0.45) | -0.38 (-0.47,-0.28) | 0.05 (-0.13,0.26) | 0.99 (0.57,1.58) | -0.13 (-0.25,0.02) | -0.37 (-0.45,-0.29) |
| Armenia | -0.67 (-0.71,-0.61) | -0.59 (-0.64,-0.53) | -0.36 (-0.44,-0.26) | 0.04 (-0.24,0.36) | 0.59 (0.19,1.10) | 0.85 (0.41,1.43) |
| Australia | 0.21 (0.02,0.43) | -0.32 (-0.41,-0.23) | -0.18 (-0.33,0.00) | 1.28 (0.93,1.68) | 0.25 (0.1,0.44) | -0.17 (-0.25,-0.09) |
| Austria | -0.42 (-0.51,-0.29) | -0.67 (-0.71,-0.62) | -0.49 (-0.57,-0.38) | 0.28 (0.08,0.54) | -0.23 (-0.33,-0.12) | -0.57 (-0.62,-0.50) |
| Azerbaijan | -0.38 (-0.55,-0.18) | -0.49 (-0.62,-0.29) | -0.27 (-0.46,-0.05) | 0.31 (-0.45,1.98) | 0.74 (-0.04,2.14) | -0.14 (-0.58,0.62) |
| Bahamas | 0.36 (0.02,0.85) | -0.12 (-0.33,0.15) | 0.46 (0.07,0.92) | 0.10 (-0.18,0.45) | 0.59 (0.17,1.15) | -0.17 (-0.39,0.09) |
| Bahrain | -0.14 (-0.42,0.21) | -0.30 (-0.47,-0.10) | 0.21 (-0.12,0.63) | -0.21 (-0.45,0.15) | 0.61 (0.13,1.28) | 0.10 (-0.21,0.57) |
| Bangladesh | -0.25 (-0.50,0.19) | -0.46 (-0.63,-0.22) | -0.14 (-0.46,0.65) | 0.07 (-0.33,0.71) | 0.28 (-0.28,1.12) | 0.12 (-0.33,1.04) |
| Barbados | 0.10 (-0.22,0.49) | -0.34 (-0.51,-0.11) | 0.16 (-0.15,0.55) | 0.12 (-0.17,0.49) | 0.19 (-0.11,0.62) | -0.24 (-0.43,0.00) |
| Belarus | 0.17 (-0.14,0.56) | -0.52 (-0.64,-0.39) | -0.04 (-0.32,0.28) | 0.39 (-0.01,0.92) | 0.86 (0.45,1.34) | 0.20 (-0.11,0.58) |
| Belgium | -0.27 (-0.38,-0.13) | -0.52 (-0.58,-0.45) | -0.35 (-0.47,-0.21) | 0.45 (0.19,0.71) | 0.15 (0.00,0.30) | -0.39 (-0.47,-0.3) |
| Belize | 0.90 (0.56,1.28) | 0.12 (-0.03,0.32) | 0.99 (0.68,1.36) | 0.85 (0.48,1.24) | 1.15 (0.51,1.82) | -0.19 (-0.39,0.1) |
| Benin | 0.66 (0.08,1.62) | -0.29 (-0.49,0.01) | 0.09 (-0.24,0.62) | -0.36 (-0.62,0.12) | 0.81 (0.17,1.71) | -0.16 (-0.42,0.22) |
| Bermuda | -0.19 (-0.40,0.08) | -0.54 (-0.65,-0.41) | -0.10 (-0.39,0.24) | -0.40 (-0.54,-0.25) | 0.07 (-0.19,0.54) | -0.54 (-0.66,-0.37) |
| Bhutan | -0.14 (-0.50,0.46) | -0.35 (-0.59,0.04) | 0.08 (-0.37,0.92) | 0.26 (-0.32,1.41) | 0.78 (0.10,2.09) | 0.28 (-0.24,1.38) |
| Bolivia (Plurinational State of) | -0.28 (-0.53,0.13) | -0.41 (-0.60,-0.16) | -0.02 (-0.35,0.59) | -0.15 (-0.48,0.36) | 0.17 (-0.23,1.02) | -0.26 (-0.54,0.23) |
| Bosnia and Herzegovina | 0.05 (-0.28,0.42) | -0.38 (-0.57,-0.16) | 0.14 (-0.25,0.49) | -0.33 (-0.58,-0.01) | 0.27 (-0.11,0.71) | -0.30 (-0.57,0.22) |
| Botswana | -0.16 (-0.5,0.36) | -0.39 (-0.61,-0.05) | 0.18 (-0.27,0.93) | 0.39 (-0.45,2.79) | 0.62 (-0.11,1.81) | 0.10 (-0.40,0.88) |
| Brazil | -0.04 (-0.11,0.03) | -0.24 (-0.28,-0.20) | 0.70 (0.58,0.81) | 0.01 (-0.05,0.08) | 0.50 (0.42,0.58) | 0.00 (-0.05,0.05) |
| Brunei Darussalam | 0.18 (-0.14,0.66) | -0.45 (-0.59,-0.26) | -0.05 (-0.31,0.29) | -0.07 (-0.48,0.71) | 0.56 (0.11,1.15) | -0.09 (-0.32,0.27) |
| Bulgaria | 0.02 (-0.18,0.26) | -0.44 (-0.58,-0.26) | 0.07 (-0.22,0.47) | -0.39 (-0.6,-0.12) | 0.22 (-0.08,0.59) | -0.04 (-0.28,0.29) |
| Burkina Faso | 0.64 (0.03,1.56) | -0.27 (-0.48,0.04) | 0.10 (-0.23,0.51) | -0.26 (-0.55,0.34) | 0.77 (0.28,1.48) | -0.11 (-0.41,0.29) |
| Burundi | -0.49 (-0.66,-0.20) | -0.42 (-0.60,-0.16) | -0.22 (-0.5,0.38) | -0.44 (-0.68,-0.01) | -0.24 (-0.56,0.36) | -0.31 (-0.59,0.43) |
| Cabo Verde | 1.26 (0.54,2.39) | -0.40 (-0.59,-0.10) | 0.86 (0.29,1.67) | 0.24 (-0.30,1.20) | 10.60 (7.32,15.84) | 1.22 (-0.10,2.72) |
| Cambodia | -0.29 (-0.55,0.11) | -0.40 (-0.60,-0.10) | 0.13 (-0.31,0.96) | -0.2 (-0.55,0.36) | 0.50 (-0.11,1.58) | 0.06 (-0.46,0.95) |
| Cameroon | 0.93 (0.20,2.12) | -0.24 (-0.50,0.10) | 0.14 (-0.26,0.75) | -0.3 (-0.58,0.17) | 0.84 (0.17,1.67) | -0.08 (-0.41,0.33) |
| Canada | 0.39 (0.19,0.62) | -0.31 (-0.39,-0.22) | 0.06 (-0.09,0.25) | 0.66 (0.39,0.99) | -0.09 (-0.19,0.04) | -0.29 (-0.37,-0.19) |
| Central African Republic | -0.19 (-0.49,0.31) | -0.20 (-0.47,0.18) | 0.06 (-0.31,0.63) | -0.36 (-0.64,0.04) | 0.03 (-0.32,0.63) | -0.06 (-0.41,0.49) |
| Chad | 1.03 (0.33,2.06) | -0.03 (-0.32,0.38) | 0.34 (-0.12,0.86) | -0.22 (-0.53,0.43) | 1.02 (0.39,1.98) | 0.07 (-0.30,0.52) |
| Chile | -0.52 (-0.58,-0.45) | -0.44 (-0.53,-0.34) | 0.33 (0.13,0.54) | 0.73 (0.37,1.20) | 0.72 (0.49,0.97) | -0.65 (-0.69,-0.61) |
| China | -0.43 (-0.59,-0.23) | -0.45 (-0.57,-0.27) | 0.06 (-0.21,0.41) | 0.00 (-0.29,0.35) | 0.52 (0.09,1.06) | 0.10 (-0.19,0.57) |
| Colombia | -0.49 (-0.58,-0.36) | -0.26 (-0.41,-0.09) | 0.55 (0.23,0.94) | -0.31 (-0.45,-0.13) | -0.02 (-0.2,0.18) | -0.4 (-0.52,-0.28) |
| Comoros | -0.15 (-0.48,0.53) | -0.23 (-0.49,0.33) | 0.16 (-0.26,1.03) | 0.00 (-0.39,0.83) | 0.28 (-0.26,1.29) | 0.11 (-0.32,0.76) |
| Congo | -0.24 (-0.55,0.27) | -0.31 (-0.56,0.09) | 0.15 (-0.30,1.14) | -0.33 (-0.64,0.16) | 0.26 (-0.31,1.66) | 0.30 (-0.21,1.40) |
| Cook Islands | 0.14 (-0.32,0.79) | -0.31 (-0.55,0.07) | -0.08 (-0.40,0.46) | 0.35 (-0.20,1.33) | 0.52 (-0.07,1.35) | -0.35 (-0.72,0.81) |
| Costa Rica | -0.03 (-0.21,0.15) | -0.2 (-0.31,-0.07) | 1.69 (1.18,2.35) | 0.17 (-0.02,0.4) | 0.72 (0.45,1.01) | -0.24 (-0.36,-0.10) |
| Croatia | -0.35 (-0.53,-0.13) | -0.65 (-0.73,-0.54) | -0.10 (-0.30,0.17) | -0.46 (-0.64,-0.18) | -0.21 (-0.36,-0.03) | -0.48 (-0.61,-0.33) |
| Cuba | 1.03 (0.56,1.56) | -0.25 (-0.38,-0.09) | 0.29 (0.03,0.60) | -0.17 (-0.36,0.04) | 0.54 (0.24,0.91) | -0.45 (-0.55,-0.32) |
| Cyprus | 0.52 (0.03,1.24) | -0.41 (-0.55,-0.22) | -0.21 (-0.43,0.11) | -0.17 (-0.44,0.22) | 0.47 (-0.03,1.26) | -0.39 (-0.55,-0.11) |
| Czechia | -0.21 (-0.38,0.01) | -0.66 (-0.74,-0.55) | -0.36 (-0.51,-0.13) | -0.61 (-0.71,-0.47) | -0.16 (-0.32,0.04) | -0.53 (-0.62,-0.42) |
| C么te d'Ivoire | 0.22 (-0.23,0.94) | -0.11 (-0.41,0.33) | 0.15 (-0.25,0.72) | -0.37 (-0.68,0.33) | 0.53 (-0.02,1.24) | 0.03 (-0.28,0.53) |
| Democratic People's Republic of Korea | 0.04 (-0.41,0.70) | -0.05 (-0.41,0.49) | 0.09 (-0.3,0.75) | -0.23 (-0.62,0.81) | 0.28 (-0.25,1.13) | 0.02 (-0.40,0.62) |
| Democratic Republic of the Congo | -0.18 (-0.48,0.30) | -0.18 (-0.44,0.18) | 0.05 (-0.33,0.63) | -0.23 (-0.54,0.22) | 0.05 (-0.42,0.81) | 0.03 (-0.34,0.71) |
| Denmark | -0.34 (-0.45,-0.23) | -0.64 (-0.69,-0.57) | -0.52 (-0.61,-0.4) | -0.26 (-0.39,-0.1) | -0.16 (-0.28,-0.01) | -0.6 (-0.66,-0.54) |
| Djibouti | -0.01 (-0.41,0.66) | -0.02 (-0.41,0.60) | 0.52 (-0.05,1.47) | 0.35 (-0.24,1.26) | 0.58 (-0.04,1.43) | 0.32 (-0.2,1.20) |
| Dominica | 0.53 (0.04,1.30) | -0.01 (-0.26,0.33) | 0.67 (0.22,1.25) | 0.52 (-0.17,1.52) | 1.02 (0.42,1.95) | 0.17 (-0.2,0.60) |
| Dominican Republic | 0.63 (0.06,1.48) | 0.03 (-0.23,0.39) | 0.56 (0.11,1.09) | 0.72 (0.03,1.64) | 1.23 (0.58,2.19) | -0.09 (-0.38,0.56) |
| Ecuador | -0.34 (-0.51,-0.13) | -0.18 (-0.38,0.06) | 0.76 (0.32,1.32) | -0.42 (-0.57,-0.23) | 0.67 (0.25,1.19) | -0.32 (-0.49,-0.11) |
| Egypt | -0.39 (-0.59,-0.12) | 0.14 (-0.39,0.55) | 0.24 (-0.04,0.57) | 0.09 (-0.35,0.73) | 0.83 (0.36,1.44) | -0.11 (-0.33,0.16) |
| El Salvador | 0.06 (-0.20,0.36) | -0.01 (-0.25,0.33) | 0.76 (0.32,1.33) | 0.10 (-0.17,0.50) | 0.82 (0.4,1.31) | -0.07 (-0.32,0.34) |
| Equatorial Guinea | -0.52 (-0.72,-0.16) | -0.68 (-0.80,-0.44) | 0.02 (-0.43,0.88) | 0.25 (-0.39,1.37) | 0.49 (-0.23,1.98) | -0.16 (-0.6,0.68) |
| Eritrea | -0.31 (-0.57,0.11) | -0.27 (-0.52,0.09) | 0.18 (-0.25,0.92) | -0.03 (-0.42,0.58) | 0.15 (-0.33,0.99) | 0.05 (-0.35,0.81) |
| Estonia | -0.47 (-0.58,-0.32) | -0.73 (-0.78,-0.68) | -0.33 (-0.5,-0.13) | -0.19 (-0.38,0.04) | -0.06 (-0.26,0.16) | -0.51 (-0.6,-0.4) |
| Eswatini | 0.46 (-0.14,1.48) | 0.23 (-0.25,0.92) | 0.90 (0.14,2.03) | 2.32 (-0.32,14.51) | 1.26 (0.26,2.89) | 0.61 (-0.06,1.48) |
| Ethiopia | -0.56 (-0.70,-0.27) | -0.66 (-0.75,-0.54) | -0.4 (-0.58,0.25) | -0.41 (-0.63,0.06) | -0.11 (-0.47,0.64) | -0.44 (-0.67,0.35) |
| Fiji | 0.24 (-0.19,0.86) | -0.23 (-0.48,0.09) | 0.09 (-0.26,0.61) | 0.21 (-0.34,1.27) | 0.50 (0.03,1.13) | 0.10 (-0.28,0.71) |
| Finland | -0.41 (-0.47,-0.34) | -0.75 (-0.79,-0.71) | -0.46 (-0.55,-0.35) | -0.32 (-0.43,-0.18) | -0.32 (-0.42,-0.22) | -0.55 (-0.62,-0.48) |
| France | -0.68 (-0.73,-0.61) | -0.39 (-0.5,-0.25) | -0.21 (-0.36,-0.03) | 0.23 (0.01,0.49) | 0.09 (-0.06,0.25) | -0.51 (-0.57,-0.44) |
| Gabon | -0.11 (-0.47,0.47) | -0.33 (-0.55,0.06) | 0.09 (-0.35,0.89) | 0.00 (-0.48,0.95) | 0.57 (-0.09,1.67) | 0.10 (-0.34,0.87) |
| Gambia | 0.38 (-0.17,1.17) | -0.14 (-0.43,0.30) | 0.23 (-0.20,0.90) | 0.18 (-0.35,1.16) | 0.63 (0.02,1.57) | 0.14 (-0.24,0.7) |
| Georgia | -0.50 (-0.58,-0.4)0 | -0.49 (-0.57,-0.38) | -0.10 (-0.25,0.08) | -0.08 (-0.29,0.18) | 3.53 (2.51,4.76) | -0.39 (-0.59,-0.11) |
| Germany | -0.43 (-0.53,-0.31) | -0.53 (-0.59,-0.47) | -0.35 (-0.46,-0.23) | 0.47 (0.16,0.83) | -0.09 (-0.20,0.06) | -0.52 (-0.58,-0.47) |
| Ghana | 0.34 (-0.12,1.01) | -0.33 (-0.54,0.01) | 0.16 (-0.26,0.91) | -0.35 (-0.67,0.42) | 1.63 (0.85,3.05) | 0.00 (-0.33,0.51) |
| Greece | 0.41 (0.25,0.62) | -0.36 (-0.42,-0.30) | 0.11 (0.00,0.25) | 1.21 (1.00,1.46) | 0.52 (0.38,0.68) | -0.07 (-0.14,0.03) |
| Greenland | -0.46 (-0.66,-0.14) | -0.58 (-0.71,-0.42) | -0.44 (-0.61,-0.22) | -0.33 (-0.63,0.22) | -0.25 (-0.48,0.06) | -0.64 (-0.78,-0.17) |
| Grenada | -0.09 (-0.33,0.2) | -0.42 (-0.55,-0.26) | 0.25 (-0.06,0.67) | 0.61 (0.16,1.26) | 0.60 (0.26,1.07) | -0.20 (-0.38,0.10) |
| Guam | 1.05 (0.60,1.59) | 0.41 (0.16,0.73) | 0.68 (0.41,1.06) | 1.96 (1.28,2.74) | 1.96 (1.39,2.59) | 0.52 (0.18,0.93) |
| Guatemala | -0.42 (-0.51,-0.31) | -0.14 (-0.27,0.00) | 0.53 (0.27,0.82) | -0.27 (-0.38,-0.15) | 0.20 (0.02,0.43) | -0.60 (-0.67,-0.53) |
| Guinea | 0.00 (-0.40,0.64) | -0.21 (-0.45,0.16) | 0.05 (-0.33,0.72) | -0.27 (-0.57,0.26) | 0.60 (0.02,1.40) | -0.06 (-0.39,0.45) |
| Guinea-Bissau | 0.59 (-0.02,1.58) | -0.23 (-0.48,0.13) | 0.12 (-0.26,0.74) | -0.39 (-0.65,0.4) | 0.70 (0.09,1.68) | -0.06 (-0.38,0.55) |
| Guyana | 0.7 (0.15,1.35) | -0.05 (-0.34,0.30) | 0.85 (0.27,1.58) | 0.36 (-0.07,0.89) | 1.19 (0.54,1.98) | -0.02 (-0.31,0.31) |
| Haiti | -0.19 (-0.48,0.23) | -0.34 (-0.55,-0.05) | -0.05 (-0.39,0.57) | -0.19 (-0.53,0.39) | 0.04 (-0.36,0.71) | -0.24 (-0.50,0.22) |
| Honduras | -0.18 (-0.52,0.27) | -0.36 (-0.6,-0.04) | -0.10 (-0.45,0.40) | 0.08 (-0.37,0.84) | 0.26 (-0.24,1.01) | -0.02 (-0.39,0.49) |
| Hungary | -0.64 (-0.71,-0.53) | -0.65 (-0.72,-0.57) | -0.19 (-0.38,0.04) | -0.6 (-0.73,-0.44) | -0.22 (-0.37,-0.04) | -0.53 (-0.62,-0.43) |
| Iceland | 0.37 (0.15,0.63) | -0.59 (-0.66,-0.51) | -0.17 (-0.32,0.02) | 0.23 (-0.03,0.53) | 0.39 (0.18,0.65) | -0.28 (-0.4,-0.16) |
| India | -0.14 (-0.26,0.04) | -0.34 (-0.43,-0.22) | 0.03 (-0.17,0.35) | 0.38 (0.14,0.63) | 0.40 (0.04,0.83) | 0.30 (-0.06,0.64) |
| Indonesia | -0.12 (-0.34,0.26) | -0.29 (-0.44,-0.05) | 0.28 (-0.08,0.89) | 0.33 (-0.04,0.87) | 0.84 (0.33,1.51) | 0.02 (-0.29,0.63) |
| Iran (Islamic Republic of) | -0.03 (-0.19,0.23) | -0.29 (-0.4,-0.16) | 0.3 (0.04,0.82) | 0.56 (0.24,0.87) | 1.45 (0.91,2.13) | 1.04 (0.14,1.88) |
| Iraq | -0.04 (-0.36,0.52) | -0.31 (-0.53,0.06) | 0.07 (-0.28,0.69) | 0.05 (-0.34,0.69) | 0.49 (-0.09,1.58) | -0.10 (-0.38,0.40) |
| Ireland | -0.10 (-0.26,0.10) | -0.56 (-0.61,-0.5) | -0.33 (-0.45,-0.2) | 0.61 (0.34,0.92) | 0.00 (-0.16,0.14) | -0.41 (-0.49,-0.32) |
| Israel | -0.09 (-0.24,0.08) | -0.54 (-0.62,-0.45) | -0.24 (-0.36,-0.10) | 0.45 (0.13,0.86) | 0.03 (-0.10,0.18) | -0.46 (-0.54,-0.38) |
| Italy | -0.49 (-0.52,-0.46) | -0.57 (-0.59,-0.55) | -0.25 (-0.3,-0.19) | -0.17 (-0.24,-0.11) | -0.03 (-0.08,0.02) | -0.40 (-0.43,-0.37) |
| Jamaica | 0.74 (0.21,1.51) | 0.09 (-0.22,0.48) | 1.28 (0.59,2.12) | 1.18 (0.42,2.09) | 0.82 (0.13,1.78) | 0.07 (-0.34,0.69) |
| Japan | -0.38 (-0.40,-0.35) | -0.73 (-0.74,-0.73) | -0.24 (-0.26,-0.21) | -0.63 (-0.65,-0.62) | 0.02 (-0.01,0.05) | -0.56 (-0.57,-0.55) |
| Jordan | -0.03 (-0.37,0.51) | -0.46 (-0.62,-0.24) | -0.08 (-0.4,0.43) | -0.18 (-0.50,0.42) | 0.63 (0.04,1.51) | -0.20 (-0.50,0.23) |
| Kazakhstan | -0.71 (-0.75,-0.67) | -0.66 (-0.7,-0.61) | -0.36 (-0.44,-0.27) | -0.54 (-0.63,-0.42) | 0.24 (-0.24,0.78) | 0.10 (-0.24,0.67) |
| Kenya | 0.44 (0.09,0.97) | 0.07 (-0.18,0.43) | 0.49 (0.14,1.04) | 0.79 (0.35,1.37) | 1.23 (0.70,2.09) | 0.34 (-0.01,0.81) |
| Kiribati | 0.11 (-0.31,0.78) | -0.01 (-0.32,0.52) | 0.12 (-0.26,0.65) | 0.00 (-0.44,0.80) | 0.36 (-0.17,1.22) | 0.13 (-0.29,0.91) |
| Kuwait | -0.24 (-0.42,-0.01) | -0.28 (-0.43,-0.09) | 1.31 (0.77,2.09) | -0.77 (-0.83,-0.69) | 1.09 (0.67,1.75) | 0.06 (-0.15,0.32) |
| Kyrgyzstan | -0.70 (-0.78,-0.59) | -0.59 (-0.68,-0.48) | -0.28 (-0.46,-0.04) | -0.68 (-0.81,-0.49) | 0.95 (0.45,1.77) | 0.01 (-0.26,0.38) |
| Lao People's Democratic Republic | -0.45 (-0.67,-0.06) | -0.56 (-0.71,-0.34) | -0.04 (-0.40,0.70) | -0.33 (-0.61,0.17) | 0.26 (-0.30,1.18) | -0.23 (-0.59,0.55) |
| Latvia | -0.25 (-0.42,-0.03) | -0.6 (-0.68,-0.51) | -0.21 (-0.39,0.00) | -0.04 (-0.32,0.32) | 0.44 (0.12,0.80) | -0.15 (-0.31,0.05) |
| Lebanon | -0.35 (-0.54,-0.01) | -0.58 (-0.7,-0.43) | -0.25 (-0.49,0.47) | -0.31 (-0.56,0.08) | 0.07 (-0.39,1.21) | -0.36 (-0.60,0.28) |
| Lesotho | 0.91 (0.19,2.07) | 0.75 (0.08,1.84) | 1.57 (0.53,3.18) | 2.67 (-0.07,16.89) | 1.78 (0.49,3.64) | 1.01 (0.09,2.48) |
| Liberia | 1.49 (0.58,2.92) | 0.03 (-0.33,0.57) | 0.33 (-0.23,1.17) | -0.02 (-0.44,0.68) | 1.03 (0.13,2.17) | 0.01 (-0.36,0.69) |
| Libya | 1.02 (0.14,2.69) | 0.23 (-0.20,0.99) | 0.56 (-0.02,1.54) | 0.97 (0.06,2.42) | 1.31 (0.30,2.97) | 0.62 (0.06,1.43) |
| Lithuania | -0.01 (-0.23,0.26) | -0.55 (-0.64,-0.45) | -0.13 (-0.33,0.12) | 0.31 (0.00,0.70) | 0.30 (0.07,0.56) | -0.15 (-0.30,0.00) |
| Luxembourg | -0.54 (-0.61,-0.46) | -0.78 (-0.81,-0.76) | -0.58 (-0.64,-0.52) | -0.13 (-0.24,0.00) | -0.37 (-0.45,-0.26) | -0.65 (-0.70,-0.61) |
| Madagascar | -0.24 (-0.5,0.14) | -0.25 (-0.48,0.06) | -0.01 (-0.3,0.39) | -0.24 (-0.52,0.17) | -0.01 (-0.37,0.60) | -0.02 (-0.33,0.46) |
| Malawi | 0.16 (-0.23,0.73) | -0.22 (-0.44,0.05) | 0.11 (-0.24,0.60) | 0.39 (-0.20,1.40) | 0.34 (-0.14,1.09) | -0.08 (-0.41,0.52) |
| Malaysia | 0.31 (-0.03,0.75) | -0.16 (-0.32,0.03) | 0.26 (-0.01,0.60) | 0.55 (-0.02,1.31) | 0.85 (0.36,1.49) | 0.05 (-0.23,0.41) |
| Maldives | -0.59 (-0.74,-0.21) | -0.7 (-0.79,-0.54) | -0.36 (-0.59,0.82) | -0.31 (-0.61,0.41) | 0.10 (-0.39,1.84) | -0.49 (-0.74,0.73) |
| Mali | -0.16 (-0.43,0.26) | -0.39 (-0.56,-0.15) | -0.15 (-0.41,0.20) | -0.09 (-0.38,0.39) | 0.11 (-0.26,0.64) | -0.20 (-0.47,0.22) |
| Malta | 0.21 (-0.01,0.47) | -0.51 (-0.58,-0.43) | -0.11 (-0.28,0.08) | 0.78 (0.47,1.23) | 0.58 (0.32,0.91) | -0.28 (-0.39,-0.14) |
| Marshall Islands | 0.44 (-0.10,1.22) | 0.08 (-0.22,0.5) | 0.45 (0.01,0.98) | 0.65 (-0.05,1.72) | 1.37 (0.63,2.49) | 0.33 (-0.12,0.91) |
| Mauritania | 0.36 (-0.11,1.03) | -0.4 (-0.58,-0.13) | 0.00 (-0.35,0.70) | -0.55 (-0.73,0.33) | 0.64 (0.05,1.76) | -0.24 (-0.52,0.29) |
| Mauritius | 0.65 (0.44,0.84) | 0.01 (-0.12,0.16) | 1.50 (1.20,1.81) | -0.78 (-0.81,-0.76) | 0.80 (0.6,1.01) | -0.33 (-0.41,-0.26) |
| Mexico | 0.03 (-0.10,0.17) | 0.05 (-0.07,0.18) | 1.25 (1.00,1.53) | 0.65 (0.46,0.86) | 0.30 (0.15,0.47) | -0.36 (-0.44,-0.27) |
| Micronesia (Federated States of) | 0.04 (-0.39,0.84) | -0.14 (-0.42,0.30) | 0.16 (-0.24,0.82) | 0.19 (-0.35,1.12) | 0.81 (0.17,1.84) | -0.04 (-0.42,0.61) |
| Monaco | 0.21 (-0.23,0.93) | -0.38 (-0.63,-0.01) | 0.10 (-0.3,0.65) | 1.12 (0.27,2.57) | 0.09 (-0.34,0.74) | -0.12 (-0.48,0.39) |
| Mongolia | -0.09 (-0.41,0.36) | -0.03 (-0.33,0.33) | 0.46 (0.05,1.00) | 0.34 (-0.25,1.22) | 9.85 (6.54,14.52) | -0.17 (-0.54,0.88) |
| Montenegro | 0.13 (-0.2,0.63) | -0.26 (-0.42,-0.07) | 0.03 (-0.24,0.37) | 0.06 (-0.27,0.54) | 0.20 (-0.16,0.79) | -0.11 (-0.34,0.17) |
| Morocco | 0.10 (-0.29,0.74) | -0.24 (-0.49,0.14) | 0.41 (-0.06,1.25) | 0.41 (-0.15,1.46) | 0.76 (0.16,1.84) | 0.19 (-0.22,0.99) |
| Mozambique | 0.33 (-0.25,1.14) | -0.03 (-0.37,0.50) | 0.30 (-0.15,0.94) | 0.30 (-0.49,3.10) | 0.58 (-0.01,1.57) | 0.11 (-0.29,0.72) |
| Myanmar | -0.42 (-0.64,-0.03) | -0.56 (-0.71,-0.35) | -0.03 (-0.41,0.63) | 0.00 (-0.45,0.97) | 0.38 (-0.21,1.48) | -0.23 (-0.55,0.39) |
| Namibia | 0.31 (-0.24,1.34) | -0.08 (-0.43,0.44) | 0.29 (-0.21,0.94) | 0.84 (-0.08,2.62) | 0.75 (0.07,1.69) | 0.22 (-0.27,0.95) |
| Nauru | -0.08 (-0.42,0.43) | -0.17 (-0.40,0.24) | -0.04 (-0.36,0.42) | -0.09 (-0.5,0.66) | 0.31 (-0.12,0.98) | -0.16 (-0.45,0.32) |
| Nepal | -0.25 (-0.51,0.15) | -0.43 (-0.62,-0.15) | -0.06 (-0.38,0.61) | 0.66 (0.02,1.67) | 0.41 (-0.09,1.29) | 0.17 (-0.26,0.92) |
| Netherlands | -0.23 (-0.36,-0.09) | -0.63 (-0.67,-0.58) | -0.21 (-0.36,-0.05) | 0.55 (0.27,0.87) | -0.09 (-0.2,0.02) | -0.42 (-0.49,-0.33) |
| New Zealand | -0.16 (-0.28,-0.03) | -0.46 (-0.55,-0.37) | -0.31 (-0.43,-0.18) | 0.88 (0.57,1.27) | 0.11 (-0.02,0.26) | 0.17 (0.04,0.32) |
| Nicaragua | -0.07 (-0.32,0.29) | -0.21 (-0.38,0.00) | 0.39 (0.03,0.82) | 0.09 (-0.26,0.58) | 0.54 (0.15,1.03) | -0.28 (-0.5,0.14) |
| Niger | 0.13 (-0.29,0.72) | -0.36 (-0.55,-0.09) | -0.13 (-0.41,0.28) | -0.6 (-0.76,-0.27) | 0.21 (-0.23,0.86) | -0.28 (-0.53,0.02) |
| Nigeria | 0.48 (0.01,1.14) | -0.36 (-0.58,0.00) | 0.00 (-0.34,0.59) | -0.28 (-0.53,0.41) | 0.52 (-0.02,1.38) | 0.01 (-0.38,0.58) |
| Niue | 0.10 (-0.33,0.83) | -0.14 (-0.44,0.32) | 0.22 (-0.20,0.95) | 0.21 (-0.37,1.26) | 0.91 (0.18,2.06) | -0.06 (-0.4,0.45) |
| North Macedonia | -0.10 (-0.37,0.3) | -0.49 (-0.61,-0.33) | -0.11 (-0.33,0.18) | -0.30 (-0.53,0.01) | 0.10 (-0.20,0.53) | -0.29 (-0.48,0.01) |
| Northern Mariana Islands | 1.10 (0.42,2.16) | -0.16 (-0.42,0.26) | 0.13 (-0.22,0.74) | 0.14 (-0.41,1.08) | 1.54 (0.66,2.94) | 0.19 (-0.23,0.85) |
| Norway | -0.06 (-0.13,0.00) | -0.71 (-0.73,-0.70) | -0.39 (-0.44,-0.33) | 1.07 (0.90,1.27) | -0.10 (-0.15,-0.05) | -0.29 (-0.33,-0.25) |
| Oman | -0.15 (-0.45,0.36) | -0.58 (-0.72,-0.35) | -0.18 (-0.51,0.42) | 0.34 (-0.23,1.47) | 0.55 (-0.05,1.44) | -0.25 (-0.55,0.2) |
| Pakistan | 0.09 (-0.22,0.53) | -0.03 (-0.30,0.35) | 0.35 (0.00,0.82) | 0.36 (-0.03,0.96) | 0.70 (0.20,1.42) | 0.23 (-0.11,0.72) |
| Palau | 0.67 (0.08,1.58) | 0.17 (-0.17,0.68) | 0.17 (-0.2,0.77) | 0.93 (0.00,2.73) | 0.52 (-0.02,1.39) | 0.42 (-0.05,1.15) |
| Palestine | -0.34 (-0.58,-0.01) | -0.47 (-0.63,-0.23) | -0.18 (-0.45,0.27) | -0.2 (-0.45,0.19) | 0.23 (-0.27,1.01) | -0.18 (-0.46,0.29) |
| Panama | -0.16 (-0.36,0.05) | -0.07 (-0.30,0.14) | 0.75 (0.35,1.17) | 0.15 (-0.09,0.40) | 0.58 (0.23,0.94) | -0.38 (-0.51,-0.22) |
| Papua New Guinea | -0.13 (-0.5,0.53) | -0.19 (-0.48,0.24) | -0.01 (-0.34,0.64) | -0.26 (-0.6,0.43) | 0.43 (-0.13,1.47) | -0.17 (-0.51,0.51) |
| Paraguay | 0.33 (-0.19,1.09) | -0.19 (-0.41,0.14) | 0.62 (0.13,1.28) | 0.60 (-0.02,1.39) | 0.96 (0.33,1.79) | -0.06 (-0.36,0.33) |
| Peru | -0.24 (-0.49,0.10) | -0.26 (-0.48,0.05) | 0.13 (-0.19,0.54) | 0.05 (-0.33,0.58) | 0.12 (-0.24,0.62) | -0.12 (-0.42,0.27) |
| Philippines | -0.10 (-0.27,0.12) | -0.28 (-0.42,-0.11) | 0.20 (-0.01,0.45) | -0.28 (-0.47,0.15) | 0.36 (0.09,0.71) | 0.06 (-0.13,0.36) |
| Poland | -0.23 (-0.32,-0.16) | -0.54 (-0.58,-0.49) | -0.08 (-0.17,0.00) | 2.21 (1.73,2.73) | -0.22 (-0.3,-0.15) | -0.28 (-0.34,-0.21) |
| Portugal | -0.15 (-0.30,0.02) | -0.63 (-0.68,-0.57) | -0.06 (-0.21,0.11) | 0.96 (0.51,1.59) | 0.16 (-0.02,0.33) | -0.34 (-0.44,-0.23) |
| Puerto Rico | -0.43 (-0.57,-0.26) | -0.54 (-0.65,-0.42) | 0.20 (-0.05,0.52) | 0.20 (-0.08,0.61) | 1.79 (1.16,2.51) | -0.41 (-0.54,-0.27) |
| Qatar | -0.36 (-0.59,-0.01) | -0.57 (-0.71,-0.40) | -0.15 (-0.40,0.21) | 0.07 (-0.36,0.65) | 0.13 (-0.23,0.62) | -0.31 (-0.53,0.03) |
| Republic of Korea | -0.65 (-0.75,-0.48) | -0.74 (-0.80,-0.60) | -0.16 (-0.35,0.12) | -0.53 (-0.7,-0.23) | -0.11 (-0.31,0.17) | -0.47 (-0.65,0.09) |
| Republic of Moldova | -0.30 (-0.40,-0.19) | -0.59 (-0.65,-0.52) | -0.11 (-0.26,0.07) | 0.17 (0.00,0.34) | 0.26 (0.09,0.44) | -0.26 (-0.38,-0.13) |
| Romania | 0.65 (0.29,1.10) | -0.30 (-0.45,-0.11) | 0.34 (0.07,0.64) | 0.77 (0.33,1.32) | 0.47 (0.21,0.80) | -0.31 (-0.42,-0.18) |
| Russian Federation | -0.06 (-0.16,0.02) | -0.57 (-0.61,-0.54) | 0.03 (-0.07,0.13) | 0.75 (0.6,0.91) | 0.01 (-0.08,0.09) | -0.01 (-0.10,0.08) |
| Rwanda | -0.52 (-0.71,-0.17) | -0.57 (-0.72,-0.35) | -0.27 (-0.53,0.22) | -0.4 (-0.65,0.02) | -0.2 (-0.55,0.61) | -0.31 (-0.59,0.41) |
| Saint Kitts and Nevis | 0.01 (-0.24,0.35) | -0.54 (-0.65,-0.40) | -0.12 (-0.32,0.16) | -0.27 (-0.47,-0.01) | 0.21 (-0.13,0.91) | -0.46 (-0.62,-0.19) |
| Saint Lucia | 0.39 (0.11,0.75) | -0.25 (-0.39,-0.06) | 0.38 (0.11,0.72) | 0.12 (-0.16,0.45) | 0.90 (0.52,1.40) | -0.19 (-0.35,0.03) |
| Saint Vincent and the Grenadines | 0.99 (0.65,1.39) | -0.07 (-0.23,0.13) | 0.84 (0.48,1.25) | 0.29 (0.05,0.57) | 0.89 (0.56,1.28) | -0.09 (-0.23,0.10) |
| Samoa | 0.31 (-0.18,1.03) | 0.18 (-0.27,0.80) | 0.40 (-0.11,1.18) | 0.22 (-0.41,1.35) | 0.59 (0.00,1.55) | 0.26 (-0.23,1.04) |
| San Marino | -0.27 (-0.67,0.36) | -0.52 (-0.75,-0.21) | -0.32 (-0.64,0.08) | 0.58 (-0.23,1.91) | -0.06 (-0.52,0.57) | -0.34 (-0.67,0.06) |
| Sao Tome and Principe | 1.36 (0.44,2.82) | -0.06 (-0.41,0.43) | 0.45 (-0.1,1.30) | -0.02 (-0.56,1.24) | 0.94 (0.15,2.27) | 0.36 (-0.16,1.1) |
| Saudi Arabia | 0.35 (-0.20,1.23) | -0.09 (-0.43,0.45) | 0.77 (0.09,2.01) | -0.27 (-0.65,0.44) | 1.82 (0.79,3.54) | 0.52 (-0.01,1.44) |
| Senegal | 0.67 (0.13,1.54) | -0.25 (-0.47,0.06) | 0.16 (-0.19,0.68) | -0.37 (-0.6,0.14) | 0.87 (0.22,1.84) | -0.1 (-0.39,0.36) |
| Serbia | -0.18 (-0.5,0.38) | -0.51 (-0.65,-0.35) | -0.25 (-0.46,0.07) | -0.18 (-0.48,0.25) | -0.03 (-0.34,0.35) | -0.33 (-0.57,0.01) |
| Seychelles | 0.33 (-0.05,0.84) | -0.25 (-0.39,-0.08) | 0.56 (0.21,0.94) | -0.32 (-0.54,-0.04) | 0.50 (0.14,0.96) | -0.22 (-0.46,0.29) |
| Sierra Leone | 0.83 (0.15,2.01) | -0.06 (-0.36,0.36) | 0.32 (-0.09,1.00) | -0.47 (-0.71,0.52) | 0.84 (0.24,1.77) | 0.00 (-0.33,0.51) |
| Singapore | -0.43 (-0.52,-0.31) | -0.71 (-0.74,-0.67) | -0.36 (-0.46,-0.25) | -0.56 (-0.63,-0.48) | -0.03 (-0.16,0.13) | -0.58 (-0.64,-0.51) |
| Slovakia | -0.31 (-0.60,0.14) | -0.54 (-0.66,-0.41) | -0.21 (-0.43,0.05) | -0.36 (-0.64,0.09) | -0.04 (-0.34,0.41) | -0.33 (-0.56,0.04) |
| Slovenia | -0.36 (-0.49,-0.2) | -0.72 (-0.78,-0.64) | -0.36 (-0.53,-0.11) | -0.56 (-0.68,-0.39) | -0.14 (-0.33,0.06) | -0.62 (-0.71,-0.51) |
| Solomon Islands | 0.27 (-0.26,1.49) | 0.07 (-0.31,0.82) | 0.42 (-0.09,1.54) | 0.08 (-0.45,2.34) | 1.14 (0.35,3.13) | 0.3 (-0.24,1.94) |
| Somalia | -0.38 (-0.59,0.00) | -0.24 (-0.50,0.17) | -0.11 (-0.43,0.41) | -0.12 (-0.54,0.62) | -0.25 (-0.54,0.23) | -0.12 (-0.43,0.46) |
| South Africa | -0.37 (-0.48,-0.23) | -0.39 (-0.47,-0.28) | 0.12 (-0.04,0.33) | 0.19 (-0.26,1.05) | 0.19 (-0.01,0.43) | 0.01 (-0.15,0.22) |
| South Sudan | 0.08 (-0.28,0.63) | 0.13 (-0.27,0.69) | 0.42 (-0.1,1.37) | 0.34 (-0.2,1.24) | 0.35 (-0.21,1.30) | 0.34 (-0.16,1.14) |
| Spain | -0.5 (-0.59,-0.4) | -0.56 (-0.61,-0.49) | -0.28 (-0.4,-0.13) | 0.24 (0.03,0.49) | 0.09 (-0.05,0.25) | -0.43 (-0.5,-0.35) |
| Sri Lanka | -0.26 (-0.58,0.13) | -0.56 (-0.73,-0.37) | -0.04 (-0.41,0.49) | -0.21 (-0.52,0.25) | 0.13 (-0.37,0.83) | -0.64 (-0.83,0.14) |
| Sudan | -0.23 (-0.55,0.25) | -0.37 (-0.63,-0.03) | 0.05 (-0.38,0.88) | -0.04 (-0.51,0.64) | 0.75 (0.03,1.99) | 0.08 (-0.51,1.27) |
| Suriname | 0.38 (-0.11,1.10) | -0.12 (-0.34,0.20) | 0.46 (0.06,1.00) | 0.52 (-0.1,1.68) | 0.75 (0.17,1.64) | 0.00 (-0.31,0.45) |
| Sweden | -0.24 (-0.36,-0.1) | -0.72 (-0.76,-0.66) | -0.36 (-0.47,-0.23) | -0.11 (-0.26,0.08) | -0.39 (-0.48,-0.29) | -0.57 (-0.63,-0.5) |
| Switzerland | -0.51 (-0.59,-0.42) | -0.66 (-0.70,-0.61) | -0.48 (-0.55,-0.39) | -0.5 (-0.61,-0.37) | -0.23 (-0.33,-0.1) | -0.57 (-0.64,-0.49) |
| Syrian Arab Republic | 0.11 (-0.27,0.78) | -0.25 (-0.50,0.14) | -0.08 (-0.42,0.57) | -0.23 (-0.54,0.26) | 0.81 (0.08,2.01) | 0.26 (-0.23,1.18) |
| Taiwan (Province of China) | 1.10 (0.84,1.36) | -0.45 (-0.52,-0.35) | 0.34 (0.18,0.52) | -0.07 (-0.22,0.11) | 0.96 (0.69,1.28) | -0.13 (-0.26,0.00) |
| Tajikistan | -0.43 (-0.64,-0.15) | -0.44 (-0.61,-0.20) | -0.34 (-0.56,0.06) | -0.24 (-0.61,0.39) | 0.08 (-0.44,1.09) | -0.2 (-0.59,0.56) |
| Thailand | 1.49 (0.57,2.97) | 0.31 (-0.08,0.91) | 1.17 (0.57,2.10) | 0.30 (-0.18,1.03) | 1.56 (0.76,2.64) | 0.74 (0.18,1.49) |
| Timor-Leste | -0.27 (-0.59,0.19) | -0.42 (-0.60,-0.14) | 0.00 (-0.36,0.66) | -0.22 (-0.57,0.36) | 0.23 (-0.27,1.22) | -0.19 (-0.51,0.35) |
| Togo | 1.46 (0.61,2.94) | 0.01 (-0.32,0.48) | 0.43 (-0.05,1.16) | -0.06 (-0.43,0.52) | 1.32 (0.51,2.58) | 0.09 (-0.32,0.69) |
| Tokelau | 0.20 (-0.29,1.08) | -0.14 (-0.4,0.31) | 0.28 (-0.14,0.9) | 0.57 (-0.04,1.71) | 1.15 (0.38,2.47) | -0.11 (-0.41,0.41) |
| Tonga | 0.25 (-0.23,1.02) | -0.01 (-0.35,0.5) | 0.20 (-0.24,0.85) | 0.25 (-0.31,1.20) | 0.73 (0.14,1.69) | -0.08 (-0.42,0.38) |
| Trinidad and Tobago | 0.47 (0.07,0.95) | -0.11 (-0.35,0.17) | 0.58 (0.15,1.06) | 0.33 (-0.03,0.74) | 0.63 (0.17,1.16) | -0.22 (-0.46,0.07) |
| Tunisia | 0.52 (-0.07,1.41) | -0.06 (-0.36,0.41) | 0.46 (-0.03,1.12) | 0.64 (-0.11,1.79) | 1.22 (0.40,2.64) | 0.33 (-0.13,0.99) |
| Turkey | -0.39 (-0.58,-0.1) | -0.60 (-0.70,-0.47) | -0.31 (-0.51,0.02) | -0.02 (-0.36,0.47) | 0.15 (-0.25,0.75) | -0.39 (-0.59,-0.03) |
| Turkmenistan | -0.53 (-0.65,-0.38) | -0.33 (-0.50,-0.11) | 0.06 (-0.20,0.40) | 0.29 (-0.11,0.82) | 22.26 (15.7,31.02) | -0.58 (-0.70,-0.42) |
| Tuvalu | -0.09 (-0.38,0.35) | -0.34 (-0.52,-0.09) | -0.05 (-0.33,0.36) | 0.12 (-0.31,0.84) | 0.61 (0.11,1.36) | -0.40 (-0.60,-0.03) |
| Uganda | 0.16 (-0.26,0.84) | -0.17 (-0.46,0.28) | 0.27 (-0.20,0.99) | 0.13 (-0.35,0.93) | 0.80 (0.12,2.04) | 0.22 (-0.25,1.03) |
| Ukraine | -0.14 (-0.45,0.28) | -0.57 (-0.7,-0.42) | -0.25 (-0.5,0.02) | 0.00 (-0.34,0.44) | 0.63 (-0.09,1.64) | 0.15 (-0.30,0.95) |
| United Arab Emirates | -0.03 (-0.33,0.42) | -0.49 (-0.63,-0.32) | -0.34 (-0.57,0.3) | 0.60 (0.02,1.53) | 0.38 (-0.18,1.19) | -0.19 (-0.43,0.28) |
| United Kingdom | 0.00 (-0.03,0.03) | -0.5 (-0.51,-0.48) | -0.17 (-0.2,-0.14) | 1.90 (1.81,2.00) | 0.02 (-0.02,0.05) | 0.10 (0.06,0.13) |
| United Republic of Tanzania | -0.27 (-0.53,0.15) | -0.31 (-0.54,0.00) | 0.12 (-0.27,0.6) | -0.16 (-0.49,0.39) | 0.19 (-0.25,0.74) | 0.04 (-0.31,0.55) |
| United States of America | -0.10 (-0.13,-0.06) | -0.21 (-0.24,-0.18) | 0.09 (0.04,0.13) | 0.48 (0.42,0.54) | -0.01 (-0.04,0.02) | -0.12 (-0.15,-0.08) |
| United States Virgin Islands | 0.16 (-0.27,0.77) | -0.29 (-0.5,0.02) | 0.07 (-0.25,0.51) | 0.46 (-0.22,1.71) | 0.89 (0.23,1.87) | -0.32 (-0.56,0.1) |
| Uruguay | -0.29 (-0.41,-0.16) | -0.24 (-0.33,-0.13) | 0.13 (-0.07,0.37) | 1.32 (0.63,2.25) | 0.26 (0.07,0.48) | -0.33 (-0.42,-0.22) |
| Uzbekistan | -0.54 (-0.65,-0.39) | -0.47 (-0.57,-0.36) | -0.08 (-0.26,0.13) | 0.47 (-0.06,1.31) | 1.26 (0.53,2.29) | 1.37 (0.56,2.52) |
| Vanuatu | 0.10 (-0.39,0.86) | -0.06 (-0.37,0.43) | 0.21 (-0.22,0.87) | 0.02 (-0.46,1.13) | 0.78 (0.14,1.75) | 0.09 (-0.35,0.71) |
| Venezuela (Bolivarian Republic of) | 0.17 (-0.18,0.60) | 0.09 (-0.20,0.47) | 0.79 (0.30,1.43) | 0.05 (-0.22,0.36) | 2.13 (1.1,3.23) | -0.19 (-0.44,0.13) |
| Viet Nam | 1.03 (0.24,2.41) | -0.16 (-0.41,0.25) | 0.82 (0.22,1.86) | 0.18 (-0.29,1.03) | 1.51 (0.57,2.95) | 0.59 (-0.09,1.74) |
| Yemen | -0.26 (-0.55,0.21) | -0.29 (-0.55,0.11) | 0.06 (-0.34,0.84) | -0.3 (-0.58,0.38) | 0.44 (-0.14,1.65) | 0.24 (-0.33,1.49) |
| Zambia | -0.09 (-0.47,0.65) | -0.24 (-0.5,0.25) | 0.48 (-0.21,2.75) | -0.53 (-0.83,0.09) | 0.29 (-0.23,1.05) | 0.06 (-0.31,0.6) |
| Zimbabwe | 1.26 (0.49,2.61) | 0.98 (0.25,1.92) | 1.33 (0.55,2.43) | 0.78 (-0.03,2.41) | 1.72 (0.81,3.23) | 1.00 (0.23,2.12) |

ASDR, age-standardized disability-adjusted life years rate. UI, uncertainty interval.

## **Table S13.** Region-specific proportion of ASIR, ASMR, and ASDR in 2021. ASIR: age-standardized incidence rate. ASMR, age-standardized mortality rate. ASDR, age-standardized disability-adjusted life years rate.

| **Location** | **Esophageal cancer** | | | **Stomach cancer** | | | **Colorectal cancer** | | | **Liver cancer** | | | **Pancreatic cancer** | | | | **Gallbladder and biliary tract cancer** | | |
| --- | --- | --- | --- | --- | --- | --- | --- | --- | --- | --- | --- | --- | --- | --- | --- | --- | --- | --- | --- |
|  | **ASIR** | **ASMR** | **ASDR** | **ASIR** | **ASMR** | **ASDR** | **ASIR** | **ASMR** | **ASDR** | **ASIR** | **ASMR** | **ASDR** | | **ASIR** | **ASMR** | **ASDR** | **ASIR** | **ASMR** | **ASDR** |
| **Global** | 8.48 | 11.44 | 11 | 25.07 | 27.61 | 27.56 | 42.44 | 27.86 | 28.64 | 15.01 | 20.62 | 20.68 | | 6.28 | 9.41 | 9.13 | 2.72 | 3.06 | 2.99 |
| **SDI Regions** | | | | | | | | | | | | | | | | | | | |
| High SDI | 5.04 | 8.59 | 8.22 | 16.13 | 19.03 | 19.01 | 59.73 | 38.97 | 40.23 | 8.43 | 13.79 | 13.69 | | 7.89 | 16.55 | 15.86 | 2.78 | 3.07 | 2.99 |
| High-middle SDI | 7.88 | 10.9 | 10.39 | 25.42 | 27.89 | 27.77 | 44.47 | 27.68 | 28.77 | 12.98 | 19.25 | 19.15 | | 6.94 | 11.91 | 11.59 | 2.31 | 2.37 | 2.32 |
| Middle SDI | 8.27 | 10.63 | 10.19 | 27.07 | 28.12 | 28.05 | 38.7 | 26.9 | 27.71 | 18.01 | 23.45 | 23.4 | | 5.51 | 8.1 | 7.93 | 2.45 | 2.8 | 2.72 |
| Low-middle SDI | 11.62 | 13.27 | 12.86 | 28.58 | 30.02 | 30.02 | 33.95 | 26.99 | 27.39 | 15.9 | 18.46 | 18.73 | | 5.6 | 6.44 | 6.3 | 4.35 | 4.81 | 4.7 |
| Low SDI | 15.6 | 16.74 | 16.12 | 30.32 | 30.83 | 30.88 | 25 | 21.04 | 21.31 | 22.6 | 24.5 | 24.95 | | 3.88 | 4.17 | 4.09 | 2.6 | 2.72 | 2.66 |
| **Regions** | | | | | | | | | | | | | | | | | | | |
| Andean Latin America | 1.94 | 2.39 | 2.32 | 49 | 53.33 | 53.34 | 31.14 | 22.41 | 22.8 | 5.19 | 6.55 | 6.78 | | 7.28 | 9.21 | 8.92 | 5.45 | 6.11 | 5.83 |
| Australasia | 3.69 | 8.14 | 7.69 | 9.27 | 13.63 | 13.48 | 67.81 | 42.84 | 44.51 | 8.68 | 17.2 | 17 | | 6.83 | 15.91 | 15.07 | 3.72 | 2.29 | 2.24 |
| Caribbean | 6.51 | 10.27 | 9.68 | 20.56 | 30.63 | 30.88 | 58.87 | 36.62 | 37.47 | 3.95 | 6.34 | 6.37 | | 8.45 | 13.71 | 13.23 | 1.65 | 2.44 | 2.37 |
| Central Asia | 8.53 | 9.97 | 9.71 | 33.61 | 36.3 | 36.3 | 31.26 | 22.26 | 22.9 | 16.42 | 19.56 | 19.44 | | 8.98 | 10.56 | 10.34 | 1.21 | 1.36 | 1.3 |
| Central Europe | 4.62 | 7.31 | 7 | 16.06 | 22.22 | 22.17 | 60.76 | 41.11 | 41.97 | 4.2 | 6.8 | 6.88 | | 11.73 | 19.09 | 18.61 | 2.63 | 3.47 | 3.38 |
| Central Latin America | 2.8 | 3.8 | 3.71 | 34.71 | 41.98 | 41.97 | 46.64 | 32.48 | 33.13 | 4.78 | 6.7 | 6.75 | | 7.66 | 10.74 | 10.35 | 3.42 | 4.29 | 4.08 |
| Central Sub-Saharan Africa | 20.83 | 22.09 | 21.33 | 29.25 | 29.43 | 29.58 | 24.52 | 21.28 | 21.68 | 19.14 | 20.56 | 20.94 | | 5.61 | 5.96 | 5.81 | 0.65 | 0.68 | 0.67 |
| East Asia | 8.74 | 11.78 | 11.22 | 27.79 | 28.49 | 28.31 | 38.5 | 23.51 | 24.58 | 18.23 | 26.18 | 26.05 | | 4.88 | 8.23 | 8.06 | 1.87 | 1.81 | 1.78 |
| Eastern Europe | 5.46 | 7.43 | 7.1 | 29.33 | 32.97 | 33.06 | 45.89 | 31.85 | 32.49 | 4.48 | 6.78 | 6.89 | | 12.71 | 19.12 | 18.66 | 2.14 | 1.85 | 1.8 |
| Eastern Sub-Saharan Africa | 24.84 | 26.58 | 25.66 | 23.05 | 23.33 | 23.52 | 28.2 | 24.29 | 24.79 | 17.9 | 19.38 | 19.71 | | 4.31 | 4.62 | 4.56 | 1.7 | 1.79 | 1.76 |
| High-income Asia Pacific | 2.85 | 3.54 | 3.36 | 29.63 | 28.8 | 29.22 | 47.41 | 30.8 | 31.95 | 11.97 | 19.29 | 18.65 | | 5.64 | 13.54 | 12.91 | 2.5 | 4.04 | 3.91 |
| High-income North America | 4.46 | 8.76 | 8.43 | 10.47 | 13.31 | 13.35 | 68.17 | 47.86 | 48.98 | 6.55 | 10.85 | 10.94 | | 7.75 | 17.3 | 16.41 | 2.6 | 1.93 | 1.89 |
| North Africa and Middle East | 4.29 | 5.95 | 5.75 | 25.93 | 33.45 | 33.41 | 48.29 | 30.54 | 31.29 | 11.23 | 16.12 | 16.13 | | 7.64 | 10.73 | 10.3 | 2.63 | 3.21 | 3.11 |
| Oceania | 4.14 | 4.53 | 4.4 | 55.87 | 56.59 | 57.18 | 20.81 | 17.49 | 17.65 | 12.9 | 14.46 | 14.22 | | 5.03 | 5.58 | 5.28 | 1.25 | 1.35 | 1.27 |
| South Asia | 14.8 | 16.52 | 16.11 | 28.07 | 29.15 | 29.22 | 31.84 | 25.99 | 26.28 | 13.7 | 15.59 | 15.87 | | 4.52 | 5.12 | 5.02 | 7.06 | 7.63 | 7.5 |
| Southeast Asia | 5.2 | 6.23 | 5.99 | 20.23 | 21.89 | 21.9 | 46.34 | 36.57 | 37.33 | 18.91 | 24.04 | 23.79 | | 6.64 | 8.52 | 8.35 | 2.67 | 2.76 | 2.65 |
| Southern Latin America | 4.25 | 6.01 | 5.82 | 21.58 | 25.9 | 25.79 | 54.49 | 41.11 | 42.15 | 2.57 | 3.71 | 3.71 | | 10.01 | 15.07 | 14.64 | 7.11 | 8.2 | 7.89 |
| Southern Sub-Saharan Africa | 20.19 | 22.13 | 21.21 | 16.51 | 16.85 | 16.85 | 28.76 | 22.85 | 23.21 | 24.63 | 27.29 | 28.17 | | 8.65 | 9.55 | 9.24 | 1.26 | 1.33 | 1.3 |
| Tropical Latin America | 10.67 | 13.65 | 13.08 | 25.74 | 29.3 | 29.42 | 46.43 | 35.22 | 35.96 | 3.58 | 4.66 | 4.76 | | 9.5 | 12.38 | 12.1 | 4.08 | 4.79 | 4.68 |
| Western Europe | 5.52 | 9.53 | 9.16 | 13.36 | 18.57 | 18.37 | 61.5 | 37.13 | 38.55 | 7.57 | 12.64 | 12.68 | | 9.41 | 19.5 | 18.68 | 2.63 | 2.63 | 2.56 |
| Western Sub-Saharan Africa | 10.84 | 11.4 | 10.8 | 20.57 | 20.33 | 20.23 | 18.33 | 15.2 | 15.22 | 45.67 | 48.26 | 49.04 | | 4.46 | 4.67 | 4.58 | 0.14 | 0.14 | 0.14 |

ASIR, age-standardized incidence rate. ASMR, age-standardized mortality rate. ASDR, age-standardized disability-adjusted life years rate. SDI, socio-demographic index.

## **Table S14.** Age-standardized rates and numbers of incidence, mortality, and DALYs of early-onset gastrointestinal cancers across five SDI regions in 2021.

|  | **Incidence** | | **Mortality** | | **DALYs** | |  |
| --- | --- | --- | --- | --- | --- | --- | --- |
|  | **Number (95%UI)** | **ASR (95%UI)** | **Number (95%UI)** | **ASR (95%UI)** | **Number (95%UI)** | **ASR (95%UI)** |  |
| **Esophageal cancer** |  |  |  |  |  |  |  |
| Global | 42698 (38138,47972) | 1.03 (0.91,1.16) | 32922 (29481,36953) | 0.79 (0.70,0.90) | 1551915 (1388751,1740151) | 37.52 (33.33,42.37) |  |
| High SDI | 4428 (4245,4627) | 0.71 (0.67,0.75) | 2784 (2663,2914) | 0.45 (0.42,0.47) | 130325 (124843,136304) | 21.11 (20.03,22.25) |  |
| High-middle SDI | 11556 (9321,14164) | 1.43 (1.15,1.81) | 8018 (6501,9801) | 0.99 (0.80,1.25) | 372792 (303062,456037) | 46.71 (37.49,58.63) |  |
| Middle SDI | 15321 (13092,17938) | 1.10 (0.93,1.30) | 11797 (10132,13749) | 0.84 (0.72,1.00) | 554399 (477354,643709) | 39.93 (34.02,46.93) |  |
| Low-middle SDI | 7198 (6406,8443) | 0.78 (0.69,0.92) | 6479 (5759,7594) | 0.7 (0.62,0.83) | 310182 (275335,365068) | 33.48 (29.54,39.83) |  |
| Low SDI | 4175 (3481,4921) | 1.02 (0.85,1.23) | 3828 (3184,4513) | 0.94 (0.78,1.13) | 183433 (152105,216066) | 44.37 (36.61,53.41) |  |
| **Stomach cancer** |  |  |  |  |  |  |  |
| Global | 125121 (107274,144783) | 3.04 (2.61,3.52) | 78871 (68704,90836) | 1.92 (1.66,2.20) | 3859036 (3356411,4428942) | 94.03 (81.42,107.52) |  |
| High SDI | 13627 (13035,14887) | 2.28 (2.14,2.47) | 5996 (5717,6573) | 0.99 (0.94,1.08) | 291911 (278437,319932) | 48.82 (46.06,53.37) |  |
| High-middle SDI | 35728 (29381,42856) | 4.63 (3.74,5.62) | 19859 (16404,23509) | 2.55 (2.09,3.04) | 960148 (793287,1130821) | 124.82 (102.43,148.52) |  |
| Middle SDI | 49237 (41551,59552) | 3.59 (3.01,4.34) | 30755 (26509,36336) | 2.24 (1.90,2.67) | 1500732 (1293397,1769054) | 109.86 (93.7,130.82) |  |
| Low-middle SDI | 17978 (15737,20489) | 1.92 (1.68,2.22) | 14853 (13028,16982) | 1.59 (1.39,1.84) | 734795 (641570,836869) | 78.16 (68.42,90.20) |  |
| Low SDI | 8479 (6631,9802) | 1.99 (1.55,2.34) | 7353 (5758,8510) | 1.73 (1.35,2.03) | 368693 (289794,426692) | 85.03 (66.42,99.78) |  |
| **Colorectal cancer** |  |  |  |  |  |  |  |
| Global | 211890 (193832,231272) | 5.15 (4.68,5.67) | 79504 (72699,86539) | 1.93 (1.76,2.11) | 4002756 (3666300,4350565) | 97.68 (88.78,106.85) |  |
| High SDI | 50985 (49340,52745) | 8.43 (8.08,8.79) | 12316 (11904,12747) | 2.03 (1.95,2.12) | 619204 (597718,644147) | 103.31 (98.97,108.23) |  |
| High-middle SDI | 62134 (54171,72026) | 8.1 (6.92,9.54) | 19428 (17107,22242) | 2.53 (2.20,2.93) | 977720 (860369,1115476) | 129.34 (112.40,150.01) |  |
| Low SDI | 7037 (6070,8090) | 1.64 (1.40,1.92) | 5058 (4355,5858) | 1.18 (1.01,1.39) | 257458 (221802,299099) | 58.68 (50.02,69.21) |  |
| Low-middle SDI | 21402 (18752,24495) | 2.28 (1.99,2.65) | 13388 (11782,15442) | 1.43 (1.25,1.67) | 672958 (590065,780314) | 71.31 (62.18,83.74) |  |
| Middle SDI | 70157 (61014,78922) | 5.13 (4.44,5.85) | 29246 (25664,32574) | 2.14 (1.87,2.41) | 1471968 (1287755,1632122) | 108.53 (95.12,122.16) |  |
| **Liver cancer** |  |  |  |  |  |  |  |
| Global | 74948 (65248,87630) | 1.82 (1.57,2.16) | 58825 (51340,68517) | 1.43 (1.23,1.69) | 2889492 (2528098,3356476) | 70.53 (60.87,83.05) |  |
| High SDI | 7188 (6677,7806) | 1.19 (1.09,1.31) | 4341 (4041,4694) | 0.72 (0.66,0.79) | 209167 (195748,225096) | 35.16 (32.26,38.52) |  |
| High-middle SDI | 18393 (14838,23253) | 2.36 (1.84,3.06) | 13661 (11050,17283) | 1.76 (1.37,2.26) | 659278 (532778,833335) | 86.10 (67.01,110.62) |  |
| Middle SDI | 32848 (27232,40731) | 2.39 (1.93,3.01) | 25623 (21354,31427) | 1.86 (1.52,2.32) | 1250555 (1047403,1528716) | 91.67 (75.10,113.85) |  |
| Low-middle SDI | 10050 (8682,11563) | 1.07 (0.91,1.26) | 9195 (7934,10582) | 0.98 (0.83,1.15) | 462526 (399447,533079) | 48.75 (41.19,57.62) |  |
| Low SDI | 6441 (4977,8558) | 1.48 (1.14,2.01) | 5980 (4607,7964) | 1.38 (1.05,1.86) | 306783 (236783,408649) | 68.69 (52.48,93.19) |  |
| **Pancreatic cancer** |  |  |  |  |  |  |  |
| Global | 31531 (28671,34517) | 0.76 (0.69,0.84) | 26996 (24493,29598) | 0.65 (0.59,0.72) | 1285174 (1164116,1407685) | 31.16 (28.24,34.29) |  |
| High SDI | 6887 (6647,7134) | 1.11 (1.07,1.16) | 5337 (5135,5542) | 0.86 (0.82,0.90) | 249984 (240784,259830) | 40.74 (38.98,42.68) |  |
| High-middle SDI | 9970 (8799,11315) | 1.26 (1.10,1.45) | 8596 (7584,9755) | 1.09 (0.94,1.25) | 406702 (358740,460247) | 52.10 (45.23,59.78) |  |
| Middle SDI | | 10093 (8732,11530) | 0.73 (0.63,0.84) | 8902 (7714,10154) | 0.64 (0.56,0.74) | 426614 (369820,485224) | 31.05 (26.79,35.44) |
| Low-middle SDI | 3490 (3143,3882) | 0.38 (0.34,0.42) | 3159 (2836,3523) | 0.34 (0.31,0.38) | 152893 (137067,170685) | 16.41 (14.66,18.45) |  |
| Low SDI | 1060 (847,1354) | 0.25 (0.20,0.33) | 975 (775,1257) | 0.23 (0.19,0.30) | 47742 (37982,61708) | 11.25 (8.90,14.61) |  |
| **Gallbladder and biliary tract cancer** |  |  |  |  |  |  |  |
| Global | 13612 (10670,15791) | 0.33 (0.26,0.38) | 8778 (6942,10235) | 0.21 (0.17,0.25) | 420527 (332554,490161) | 10.20 (8.04,11.95) |  |
| High SDI | 2397 (2127,2544) | 0.39 (0.35,0.42) | 985 (856,1055) | 0.16 (0.14,0.17) | 46883 (40622,50182) | 7.68 (6.67,8.35) |  |
| High-middle SDI | 3303 (2253,3993) | 0.42 (0.29,0.51) | 1713 (1195,2029) | 0.22 (0.15,0.26) | 81586 (56625,96516) | 10.45 (7.25,12.48) |  |
| Middle SDI | 4491 (3465,5436) | 0.32 (0.25,0.40) | 3086 (2407,3663) | 0.22 (0.17,0.27) | 147117 (114967,173945) | 10.67 (8.33,12.80) |  |
| Low-middle SDI | 2707 (2115,3334) | 0.29 (0.23,0.37) | 2356 (1828,2894) | 0.25 (0.20,0.32) | 113819 (88265,140561) | 12.23 (9.48,15.24) |  |
| Low SDI | 707 (497,923) | 0.17 (0.12,0.23) | 632 (442,824) | 0.15 (0.11,0.20) | 30863 (21516,40145) | 7.32 (5.18,9.76) |  |

ASR, age-standardized rate. DALYs, disability-adjusted life years. SDI, socio-demographic index. UI, uncertainty interval.

## **Table S15.** Age-standardized rates of early-onset gastrointestinal cancers projected until 2036 globally.

|  | **ASIR (95%UI)** | **ASMR (95%UI)** | **ASDR (95%UI)** |
| --- | --- | --- | --- |
| Esophageal cancer | 1.33 (0.94,1.72) | 0.98 (0.68,1.28) | 37.91 (26.16,49.66) |
| Stomach cancer | 2.50 (2.00,3.00) | 1.45 (1.12,1.78) | 71.82 (55.82,87.82) |
| Colorectal cancer | 5.53 (4.49,6.57) | 1.76 (1.41,2.11) | 91.18 (72.62,109.74) |
| Liver cancer | 1.75 (1.04,2.46) | 1.33 (0.67,1.99) | 66.85 (35.26,98.44) |
| Pancreatic cancer | 0.72 (0.59,0.85) | 0.62 (0.51,0.73) | 30.24 (24.96,35.52) |
| Gallbladder and biliary tract cancer | 0.32 (0.26,0.38) | 0.19 (0.16,0.22) | 11.04 (9.22,12.86) |

ASIR, age-standardized incidence rate; ASMR, age-standardized mortality rate; ASDR, age-standardized disability-adjusted life years rate. UI, uncertainty interval.

## **Table S16.** Comparison of ASIR and ASMR of early-onset esophageal, stomach, colorectal, and pancreatic cancers between GBD 2021 and GLOBOCAN 2022 globally.

|  | **ASIR** | | **ASMR** | |
| --- | --- | --- | --- | --- |
|  | **GBD 2021** | **GLOBOCAN 2022** | **GBD 2021** | **GLOBOCAN 2022** |
| **Esophageal cancer** |  |  |  |  |
| Both | 1.03 | 1.10 | 0.79 | 0.98 |
| Female | 0.48 | 0.74 | 0.37 | 0.63 |
| Male | 1.57 | 1.50 | 1.21 | 1.30 |
| **Stomach cancer** |  |  |  |  |
| Both | 3.04 | 2.00 | 1.92 | 1.30 |
| Female | 2.13 | 1.90 | 1.43 | 1.20 |
| Male | 3.94 | 2.20 | 2.40 | 1.40 |
| **Colorectal cancer** |  |  |  |  |
| Both | 5.15 | 4.60 | 1.93 | 1.60 |
| Female | 4.17 | 4.40 | 1.57 | 1.50 |
| Male | 6.11 | 4.80 | 2.29 | 1.70 |
| **Liver cancer** |  |  |  |  |
| Both | 1.82 | 2.70 | 1.43 | 2.20 |
| Female | 0.78 | 1.30 | 0.64 | 1.00 |
| Male | 2.84 | 4.00 | 2.21 | 3.40 |
| **Pancreatic cancer** |  |  |  |  |
| Both | 0.76 | 0.73 | 0.65 | 0.53 |
| Female | 0.50 | 0.60 | 0.43 | 0.40 |
| Male | 1.02 | 0.87 | 0.88 | 0.66 |

ASIR, age-standardized incidence rate; ASMR, age-standardized mortality rate

Data of GLOBOCAN 2022 available from <https://gco.iarc.fr/en>
